# RBL1/p107 Expression Levels Are Modulated by Multiple Signaling Pathways

**DOI:** 10.3390/cancers13195025

**Published:** 2021-10-08

**Authors:** Elisa Ventura, Carmelina Antonella Iannuzzi, Francesca Pentimalli, Antonio Giordano, Andrea Morrione

**Affiliations:** 1 Sbarro Institute for Cancer Research and Molecular Medicine, Center for Biotechnology, College of Science and Technology, Temple University, Philadelphia, PA 19122, USA; tuj82107@temple.edu (E.V.); antonio.giordano@temple.edu (A.G.); 2 Cell Biology and Biotherapy Unit, Istituto Nazionale Tumori, IRCCS, Fondazione G. Pascale, I-80131 Napoli, Italy; c.iannuzzi@istitutotumori.na.it (C.A.I.); f.pentimalli@istitutotumori.na.it (F.P.); 3 Department of Medical Biotechnologies, University of Siena, I-53100 Siena, Italy

**Keywords:** RBL1/p107, AKT, CaMK

## Abstract

**Simple Summary:**

The retinoblastoma-like (RBL)1/p107 protein is a member of the retinoblastoma (RB) family including the retinoblastoma (RB)1/p105 protein and RBL2/p130. They are key regulators of the cell cycle and play a central role in regulating cell proliferation. RB proteins act as tumor suppressors and their dysregulation is associated with tumor initiation. However, the mechanisms regulating RB protein actions are still very poorly characterized. We previously demonstrated that RBL2/p130 is a direct target of AKT and it is a key mediator of apoptosis associated with AKT inhibition. Here we demonstrated that RBL1/p107 levels are instead not directly modulated by AKT and discovered that RBL1/p107 levels are regulated by multiple pathways linked directly or indirectly to Ca^2+^-dependent signaling. These novel observations suggest a complex regulation of RBL1/p107 expression involving different components of signaling pathways controlled by Ca^2+^ levels, pointing out a significant difference with the mechanisms modulating the close family member RBL2/p130.

**Abstract:**

The members of the retinoblastoma (RB) protein family, RB1/p105, retinoblastoma-like (RBL)1/p107 and RBL2/p130 are critical modulators of the cell cycle and their dysregulation has been associated with tumor initiation and progression. The activity of RB proteins is regulated by numerous pathways including oncogenic signaling, but the molecular mechanisms of these functional interactions are not fully defined. We previously demonstrated that RBL2/p130 is a direct target of AKT and it is a key mediator of the apoptotic process induced by AKT inhibition. Here we demonstrated that RBL1/p107 levels are only minorly modulated by the AKT signaling pathway. In contrast, we discovered that RBL1/p107 levels are regulated by multiple pathways linked directly or indirectly to Ca^2+^-dependent signaling. Inhibition of the multifunctional calcium/calmodulin-dependent kinases (CaMKs) significantly reduced RBL1/p107 expression levels and phosphorylation, increased RBL1/p107 nuclear localization and led to cell cycle arrest in G0/G1. Targeting the Ca^2+^-dependent endopeptidase calpain stabilized RBL1/p107 levels and counteracted the reduction of RBL1/p107 levels associated with CaMKs inhibition. Thus, these novel observations suggest a complex regulation of RBL1/p107 expression involving different components of signaling pathways controlled by Ca^2+^ levels, including CaMKs and calpain, pointing out a significant difference with the mechanisms modulating the close family member RBL2/p130.

## 1. Introduction

The retinoblastoma-like (RBL)1/p107 protein is a member of the family of pocket proteins which includes the retinoblastoma (RB)1/p105 protein and RBL2/p130. RB proteins are key components of the cell cycle machinery and play a central role in regulating cell proliferation [1]. RB proteins restrain the cell cycle and act as tumor suppressors by interacting with the E2F transcription factors and, in the case of RBL2/p130 and RBL1/p107, by binding to the Multivulva class B (MuVB) core to form the transcriptional repressor DREAM (DP, Rb-like, E2F and MuvB) complex [2,3]. In addition, RB proteins antagonize the function of cycling-dependent kinases (CDK)s and regulate chromatin organization by interacting with histone-modifying enzymes and chromatin-regulating proteins [4]. RB proteins are often dysregulated in cancer and their dysregulation has been associated with tumor initiation and progression. However, deciphering the molecular mechanisms underlying the function of a specific RB protein has been complicated by their functional redundancy [5,6].

RBL1/p107 is structurally related to RBL2/p130 and has both overlapping and distinct functions with the other two RB protein family members [6]. Unlike RB1 and RBL2/p130, *RBL1* is considered a G1/S gene whose expression is low in quiescent cells and high during the G1-to-S phase transition [5,7]. However, in S phase, similarly to RB1/p105 and RBL2/p130, the RBL1/p107 protein is hyperphosphorylated and inactivated by cyclin-cyclin-dependent kinase (CDK) complexes [8,9]. Multiple studies support the role of RBL1/p107 as a tumor suppressor [10,11,12,13]. However, it is a weaker tumor suppressor when compared to RB1/p105, and its role as a tumor suppressor is often context-dependent [10]. In addition, it has been recently reported that variants mapping to RBL1 correlated with tumor T cell subset abundance [14]. Similarly to RBL2/p130, RBL1/p107 binds to the repressors E2F4 and E2F5 [15] and, in the absence of RBL2/p130, it is able to assemble into DREAM-like complexes but it is not clearly defined whether these repressor complexes are functional [2,16,17]. In addition, it has been demonstrated that upon DNA damage and p53 activation, RB1/p105 and RBL2/p130 repress the expression of G1/S genes whereas RBL2/p130 and RBL1/p107 repress G2/M genes [16]. However, in the absence of RB1/p105 and RBL2/p130, RBL1/p107 can also repress G1/S genes [16].

Our laboratories have recently demonstrated that RBL2/p130 is a direct AKT target and mediates apoptosis induced by AKT inhibition in lung cancer and mesothelioma cells [18]. These findings demonstrated that RB protein stability and activity are not exclusively controlled by CDKs and that oncogenic pathways might play a crucial role in modulating RB family member expression and action. Accordingly, additional reports indicated that the mitogen-activated protein kinase p38 phosphorylates RB1/p105 and modulates RB1/p105 stability [19].

Dysregulation of Ca^2+^ signaling has been implicated in tumor initiation and progression. Indeed, aberrant Ca^2+^ signaling is recognized as one of the hallmarks of cancer [20]. Calcium/calmodulin-stimulated protein kinases (CaMKs) are key mediators of Ca^2+^ action [21]. CaMKs are multifunctional serine/threonine protein kinases including CaMKI, CaMKII and CaMKIV. CaMKI and CaMKII constitute two different families each composed of four members encoded by four different genes, whereas CaMKIV is encoded by a single gene [21]. Alternative splicing modulates pre-mRNA processing for all CaMKs, further increasing the number of CaMKs proteins. All CaMKs require Ca^2+^ for their activity but they differ in the mechanisms of activation. CaMKII, upon Ca^2+^/calmodulin binding, undergoes autophosphorylation at Thr286, which renders CaMKII activity independent from Ca^2+^/calmodulin binding [22]. In contrast, CaMKI and CaMKIV activity is dependent on the phosphorylation status of their activation loop by Ca^2+^/calmodulin-dependent protein kinase kinases (CaMKK) [23,24].

Growing evidence suggests a role for CaMKs in tumorigenesis [25]. CaMKs are either upregulated or dysregulated in many tumor types and modulate key cellular process including proliferation, survival and cell motility [25]. Previously published data have reported that CaMKs play an important role in the regulation of the cell cycle, affecting every phase of the cell cycle [26].

Here, we investigated the mechanisms regulating RBL1/p107 protein levels and demonstrated that multiple pathways connected to Ca^2+^ signaling and CaMKs play a critical role in modulating RBL1/p107 protein levels and phosphorylation. Several studies demonstrated that CaMKs modulate the cells cycle by affecting cyclin D1 expression, CDK4 activation, p27 binding to CDK2 and RB1/p105 phosphorylation [27]. Here, we show that CaMK activity might also affect RBL1/p107. Indeed, CaMK inhibition modulated RBL1/p107 levels, phosphorylation status and subcellular distribution. In addition, we demonstrated that the inhibition of calpain, an endopeptidase whose activity relies on Ca^2+^ signaling, also affected RBL1/p107 levels and subcellular distribution. Notably, our data ruled out a direct major role of AKT in modulating RBL1/p107, pointing out a significant difference with the mechanisms modulating the close family member RBL2/p130.

## 2. Materials and Methods

### 2.1. Cell Culture and Reagents

A549 and MSTO-211H cells were provided by ATCC and cultured in DMEM and RPMI (Thermo Fisher Scientific, Waltham, MA, USA) supplemented with 10% FBS (R&D Systems, Minneapolis, MN, USA) and 1% L-glutamine (Thermo Fisher Scientific), respectively. Cells were treated with the AKT inhibitor AKTi VIII (Calbiochem, San Diego, CA, USA), epidermal growth factor (EGF) (Invitrogen, Carlsbad, CA, USA), insulin growth factor (IGF)2 (Peprotech, Rocky Hill, NJ, USA), the lysosome inhibitor bafilomycin A1 (Sigma-Aldrich, St. Louis, MO, USA), the CDK4/CDK6 inhibitor Abemaciclib (Selleckchem, Huston, TX, USA), and the following compounds from Cayman Chemicals (Ann Arbor, MI, USA): the MEK1/2 inhibitor U0126, the proteasomal inhibitor MG-132, the CaMK inhibitor KN-93, the KN-93 inactive derivative KN-92, the CaMKK inhibitor ST-609 and the calpain inhibitor 1/ALLN.

### 2.2. Immunoblot

Cells were lysed in RIPA buffer (Thermo Fisher Scientific) supplemented with the halt protease and phosphatase inhibitors cocktail (Thermo Fisher Scientific). Nuclear and cytoplasmic fractions were obtained using the NE-PER nuclear and cytoplasmic extraction reagents kit (Thermo Fisher Scientific). Immunoblot analysis was performed using the following primary antibodies: RBL1/p107 (13354-I-AP, Proteintech, Rosemont, IL, USA), RBL2/p130 (610262, BD, Franklin Lakes, NJ, USA), pRBL1/pp107 Ser975 (MBS9214765, Mybiosource, San Diego, CA, USA), HA (Rockland, Limerick, PA, USA), the antibodies from Santa Cruz Biotechnology (Santa Cruz, Dallas, TX, USA) GAPDH (sc-365062), beta-actin (sc-47778), alpha-tubulin (sc-398103), c-myc (sc-40), cyclin D3 (sc-182), and GFP (sc-9996), and the antibodies from Cell Signaling Technology (Danvers, MA, USA) pAKT Ser473 (4060), pan-AKT (4691), pERK1/2 (9160), ERK1/2 (9102), cyclin D1 (55506), lamin A/C (2032), CamKII (4436). The following secondary antibodies were used: mouse anti-rabbit IgG-HRP (sc-2357, Santa Cruz Biotechnology) and m-IgGk BP-HRP (sc-516102, Santa Cruz Biotechnology).

### 2.3. Gene Depletion and Expression

Transient gene depletion was performed by siRNAs using 25 nM ON-TARGET plus, small interfering RNA (siRNA) targeting AKT1 L-003000-00-0005 (Dharmacon, Lafayette, CO) and the Dharmafect transfection reagent according to the manufacturer’s instructions. A549 and MSTO-211H cells were transiently transfected using the Lipofectamine 3000 (Invitrogen) and the *Trans*IT-2020 (Mirus, Madison, WI, USA) reagents, respectively, according to the manufacturers’ recommendation.

To generate A549 cells with RBL1/p107 knock-out, we used CRISPR/Cas9 strategies. The sequence of the sgRNA GAGGACAAGCCCCACGCTGA specific for RBL1/p107 exon 1 [28] was inserted in the lentiviral vector lentiCRISPR v2-Blast, a gift from Mohan Babu (Addgene plasmid #83408, Addgene, Wartertown, MA, USA). Lentiviral particles were generated by transfecting HEK-293FT cells with the pMD2-G envelope plasmid and the psPAX2 packaging plasmid. HEK-293FT supernatants were used to transduce A549 cells in the presence of 8 µg/mL polybrene (Sigma-Aldrich).

To generate lentiviral particles for CDK6 and CDK6 S178P expression, HEK-293FT cells were transfected with the pMD2-G envelope plasmid, the psPAX2 packaging plasmid and the plasmid pHAGE/CDK6, a gift from Gordon Mills and Kenneth Scott (Addgene plasmid #116725) [29] or the following described plasmid pHAGE/CDK6 S178P. HEK-293FT supernatants were used to transduce A549 and MSTO-211H cells in the presence of 8 µg/mL polybrene (Sigma-Aldrich).

### 2.4. cDNA Constructs

pcDNA3 Myr HA Akt1 (Addgene plasmid #9008) and pcDNA3 Myr HA Akt2 (Addgene plasmid #9016) were a gift from William Sellers [30].

The following plasmids were used to obtain the cDNAs encoding CaMKIα, CaMKIIα and CaMKIV: pDONR223-CAMK1 (Addgene plasmid # 23544), pDONR223-CAMK2A (Addgene plasmid # 23408) and pDONR223-CAMK4 (Addgene plasmid # 23812). All plasmids were a gift from William Hahn and David Root [31]. cDNAs were obtained by PCR or by two-step PCR to insert desired mutations by using Phusion high-fidelity DNA polymerase (New England Biolabs, Ipswich, MA, USA) following the manufacturer’s instructions and primers reported in the Supplemental Table S1. All the cDNAs encoding CaMKs were digested with the restriction enzymes NcoI and NotI (New England Biolabs) and ligated into NcoI/NotI digested pENTR11 vector (Invitrogen), by using the T4 DNA ligase (Roche, Basel, Switzerland). In the case of CaMKI- and CaMKIV-based constructs, the cDNAs were fused to their 3′ terminus to the sequence encoding the c-myc tag, by ligating the cDNAs with a previously prepared NcoI/NotI-digested pENTR11 vector including the c-myc tag sequence. The cDNA encoding the various CaMKs was then cloned into the pLenti CMV Puro DEST vector, a gift from Eric Campeau and Paul Kaufman (Addgene plasmid # 17452) [32], by DNA recombination using the Gateway LR Clonase II (Invitrogen) according to the manufacturer’s instructions.

RcCMV/CycD1 and RcCMV/CycD3 were previously described. CDK6-HA (Addgene plasmid #1868) and CDK6-DN-HA (Addgene plasmid #1869) were a gift from Sander van den Heuvel [33]. Site-directed mutagenesis to generate pHAGE/CDK6 S178P was performed by two-step PCR, using the above mentioned plasmid pHAGE/CDK6 and the primers reported in Supplemental Table S1.

### 2.5. RT-PCR

RT-PCR was performed using the delta-CT method, RBL1/p107 forward and reverse primers and β-actin as a house-keeping gene. Primer sequences are reported in the Supplemental Table S1.

### 2.6. Cell Cycle Analysis

Cells were fixed with ice-cold 70% ethanol and stained with propidium iodide using the propidium iodide flow cytometry kit (Abcam, Cambridge, UK). Cell DNA content was determined by FACS analysis at the Wistar Institute Cytofluorimetry Core Facility.

### 2.7. Statistical Analysis

All experiments were performed at least twice. Data are shown as the mean ± the standard deviation.

## 3. Results

**Figure 1:**
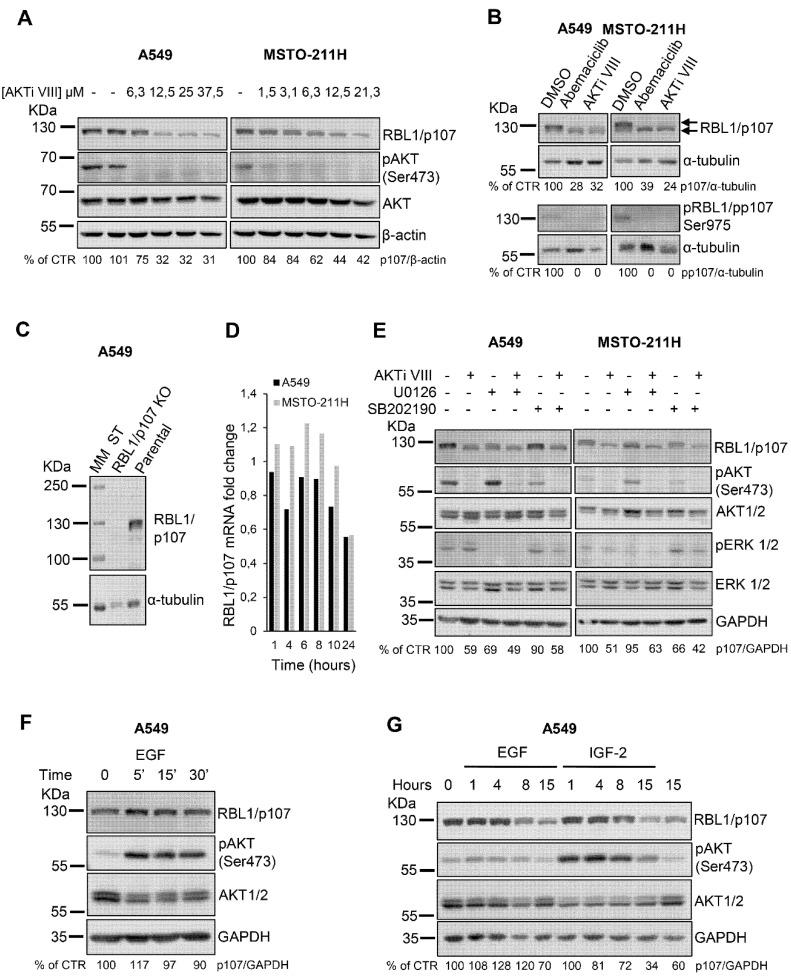
The AKT inhibitor AKTi VIII reduces RBL1/p107 levels in A549 and MSTO-211H cells. (**A**). A549 and MSTO-211H cells were treated with the indicated concentrations of the AKT inhibitor AKTi VIII for 16 h. RBL1/p107 levels were analyzed in cell lysates by immunoblot. (**B**). A549 and MSTO-211H cells were treated with 0.2 µM Abemaciclib or 12.5 µM AKTi VIII for 16 h. Levels of total and phosphorylated p107 (ser975) were analyzed by immunoblot. Arrows indicate the two major bands corresponding to hyper- and hypo-phosphorylated RBL1/p107. (**C**). RBL1/p107 levels were analyzed in cell lysates of parental and A549 cells with RBL1/p107 knock-out by CRISPR/Cas9. MM: molecular mass. (**D**). RBL1/p107 mRNA levels were determined by RT-PCR in A549 and MSTO-211H cells treated with 12.5 µM AKTi VIII for the indicated time points. (**E**). RBL1/p107 levels were determined by immunoblot in A549 and MSTO-211H cells preincubated with the MEK1/2 inhibitor U0126 or the p38 inhibitor SB202190, at the concentration of 10 and 20 µM, respectively, for 2 h and then treated with the same concentrations of U0126 or SB202190 alone or in combination with 12.5 µM AKTi VIII for 16 h (**F**). A549 cells were treated with 50 ng/mL EGF for the indicated time and RBL1/p107 protein levels analyzed by immunoblot. (**G**). RBL1/p107 levels were analyzed in cell lysates of A549 cells treated for the indicated time with 50 ng/mL EGF or 10 nM IGF-2. Western blot quantification was performed using the ImageJ program, normalized to β-actin, α-tubulin or GAPDH expression and reported as % of control (CTR). All experiments were performed three times.

We have recently demonstrated that in lung cancer and mesothelioma cells RBL2/p130 is a direct AKT target and mediates the apoptotic process induced by AKT inhibition [18]. However, whether AKT signaling might modulate the levels or activity of the related RBL1/p107 protein is not established. Thus, we first tested in A549 lung cancer and MSTO-211H mesothelioma cells whether AKT inhibition by the specific AKT inhibitor VIII (AKTi VIII) might affect also RBL1/p107 protein levels. In both cell lines, AKT inhibition determined a dose-dependent decrease in RBL1/p107 protein levels as well as RBL1/p107 dephosphorylation as suggested by a slight reduction in the molecular mass of RBL1/p107 detected in AKTi VIII-treated versus untreated cells (Figure 1A,B). Indeed, RBL1/p107 was detectable as two major bands (Figure 1B, arrows). Importantly, these bands are specific for RBL1/p107 forms, as in fact they are not detectable in A549 cells with RBL1/p107 knock-out by CRISPR/Cas9 strategies (Figure 1C). The slower migrating form was not detectable in cells exposed to the CDK4/CDK6 inhibitor Abemaciclib, suggesting that this form corresponds to hyperphosphorylated RBL1/p107 (Figure 1B), as in fact CDK4/CDK6, along with CDK2, are the main kinases responsible for RBL1/p107 phosphorylation [8,34]. In agreement, CDK4/CDK6 inhibition led to a reduction in pRBL1/pp107 (Ser975) levels (Figure 1B). Upon cell exposure to AKTi VIII, total levels of RBL1/p107 were reduced and the reduction of RBL1/p107 levels was more pronounced for the slower migrating, hyper-phosphorylated form of RBL1/p107. Accordingly, pRBL1/pp107 (Ser975) was undetectable in AKTi VIII-treated cells (Figure 1B). The effect of AKTi VIII on RBL1/p107 levels was post-translational, as in fact RBL1/p107 mRNA levels were only minimally affected by AKTi VIII treatment (Figure 1D). Importantly, pharmacological inhibition of MEK (U0126) or p38 (SB202190), alone or in combination with AKTi VIII, had no significant effect on RBL1/p107 protein levels (Figure 1E) indicating that MAPK and p38 do not directly. 

Contribute to the regulation of RBL1/p107 levels in this setting. Surprisingly, while pharmacological AKT inhibition decreased RBL1/p107 levels, AKT activation induced by growth factors did not affect RBL1/p107 levels, either after short EGF stimulation (Figure 1F) or chronic exposure to either EGF or IGF2 (Figure 1G).

**Figure 2:**
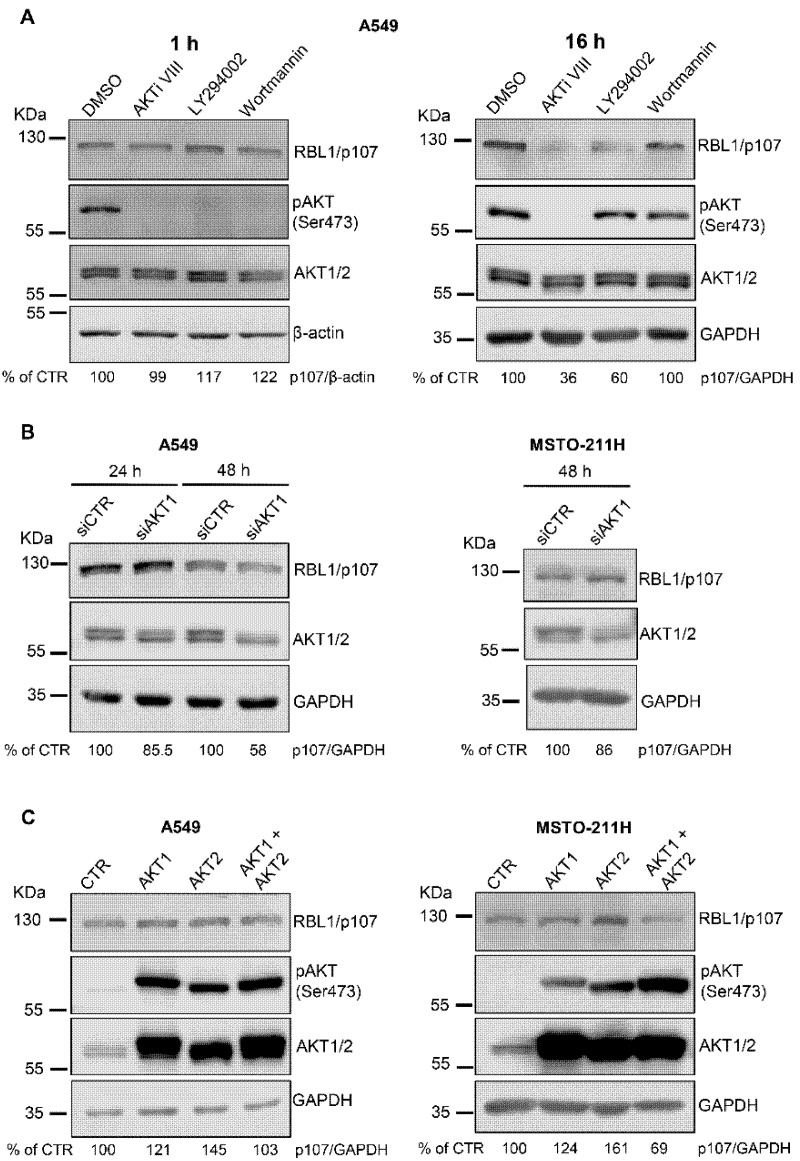
The modulation of AKT expression does not significantly affect RBL1/p107 levels. (**A**). A549 cells were treated with 12.5 µM AKTi VIII, 20 µM LY294002 or 1 µM Wortmannin for 1 h (left panel) or 16 h (right panel). RBL1/p107 levels were analyzed by immunoblot. (**B**). A549 and MSTO-211H cells were transfected with siRNA targeting AKT1 or non-targeting control siRNA and the levels of RBL1/p107 assessed by immunoblot. (**C**). RBL1/p107 levels as determined in A549 and MSTO-211H cells expressing myristoylated AKT1, AKT2, the combination of myristoylated AKT1 and AKT2 or an empty plasmid. AKT1 and AKT2 were detected using an anti-pan AKT antibody recognizing both AKT1 and AKT2. Quantification was performed by densitometric analysis using the ImageJ program and expressed as % of control. All experiments were performed three times.

To further define the role of AKT in modulating RBL1/p107 levels, we used two additional pharmacological inhibitors of the AKT signaling pathway, the PI3K inhibitors LY294002 and Wortmannin. All the compounds were effective in inhibiting the AKT pathway, as demonstrated by the reduction of AKT activation after 1 h of cell treatment with either AKTi VIII, LY294002 or Wortmannin (Figure 2A, left panel). However, in A549 cells treated with LY294002 and Wortmannin AKT activation was restored after 16 h of incubation with the inhibitors whereas AKT activity was still undetectable in AKTi VIII-treated cells, suggesting different time-dependency in the effects of the three compounds on the AKT signaling pathway (Figure 2A, right panel). Despite these differences, LY294002 induced a reduction of RBL1/p107 levels, which was instead not evident in Wortmannin-treated A549 cells (Figure 2A). To additionally confirm the role of AKT in modulating RBL1/p107 levels, we used siRNA approaches to transiently deplete AKT1 in A549 and MSTO-211H cells. Surprisingly, AKT1 depletion did not significantly affect RBL1/p107 levels in spite of a significant reduction of AKT1 protein (Figure 2B). The overexpression of constitutively active myristoylated AKT1 led to a minor increase in RBL1/p107 levels (Figure 2C). Accordingly, the overexpression of constitutively active myristoylated AKT2 led to an increase in RBL1/p107 levels (Figure 2C). However, when constitutively active AKT1 and AKT2 were expressed in combination, the levels of RBL1/p107 were not significantly affected and even showed a slight decrease in MSTO-211H cells (Figure 2C). All together, these results challenge the potential role of AKT in directly modulating RBL1/p107 levels, suggesting only a minor regulation of RBL1/p107 by AKT and the involvement of different pathways, which may be affected upon AKT inhibition.

**Figure 3:**
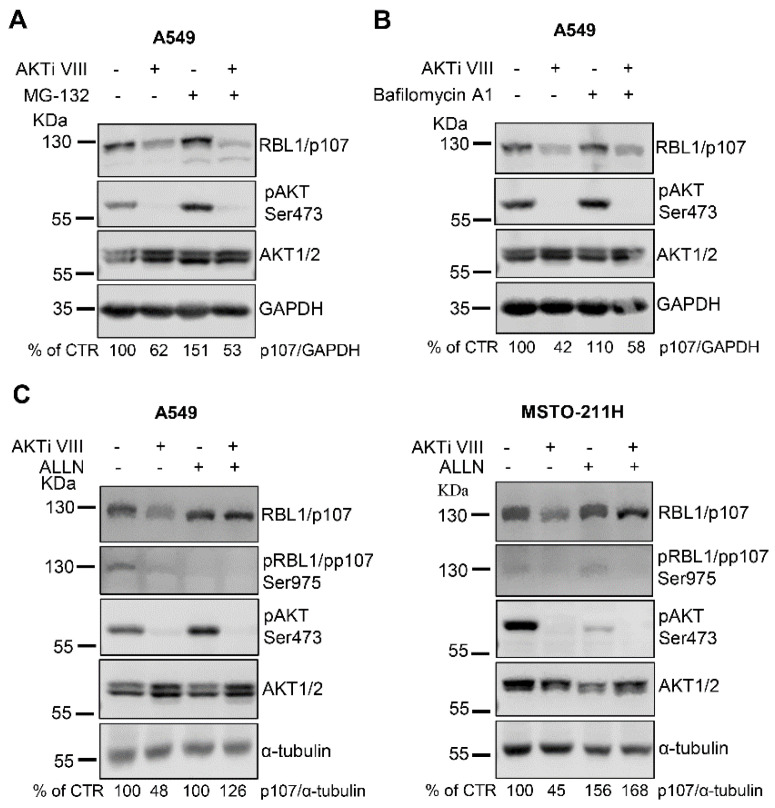
The inhibition of calpain stabilizes RBL1/p107 protein levels, which is not affected by either proteosomal or lysosomal inhibitors. (**A**–**C**). RBL1/p107 levels and RBL1/p107 phosphorylation at Ser975 (**C**) were determined by immunoblot in A549 cells and MSTO-221H cells (**C**) preincubated with the proteasome inhibitor MG-132 (1 µM) (**A**), the lysosome inhibitor bafilomycin A1 (0.1 µM) (**B**) or the calpain inhibitor ALLN (100 µM) (**C**), for 1 h and then treated for 16 h with 12.5 µM AKTi VIII alone or in combination with MG-132 (**A**), bafilomycin A1 (**B**), or ALLN (**C**). Quantification was performed by densitometric analysis using the ImageJ program and expressed as % of control. All experiments were performed three times.

Since short-term treatment with AKTi VIII did not affect RBL1/p107 levels (Figure 2A), we ruled out a direct effect of AKT on RBL1/p107 protein degradation. However, because AKT signaling might indirectly modulate the activity of the proteasome [35], we investigated whether RBL1/p107 downregulation associated with AKT inhibition was proteasome-dependent by exposing A549 cells to the proteasome inhibitor MG-132, alone or in combination with AKTi VIII. As shown in Figure 3A, the proteasomal inhibitor did not impact on AKTi VIII-induced RBL1/p107 downregulation, indicating that the proteasomal pathway is likely not involved in the process. Thus, we alternatively investigated a potential role of the lysosomes, since lysosomal-dependent protein degradation is in part controlled by AKT signaling [35]. However, cell exposure to the lysosome inhibitor bafilomycin A1 did not affect RBL1/p107 levels (Figure 3B) indicating that the lysosomal compartment does not clearly contribute to the AKTi VIII-induced regulation of RBL1/p107 protein levels.

Since it has been reported that the calcium-dependent endopeptidase calpain might regulate RBL1/p107 protein levels [36,37], we exposed A549 cells to the calpain inhibitor 1/ALLN, which did not change the total levels of RBL1/p107, but enhanced the relative levels of hypo-phosphorylated RBL1/p107 (Figure 3C, left panel). Importantly, in the context of calpain inhibition, AKTi VIII had no effect on RBL1/p107 levels (Figure 3C, left panel). In MSTO-211H cells, the calpain inhibitor did not alter RBL1/p107 phosphorylation, but increased the total levels of RBL1/p107, and, as in A549, counteracted the reduction of RBL1/p107 levels caused by AKTi VIII exposure (Figure 3C, right panel). Thus, these data suggest that the decrease of RBL1/p107 associated with AKT inhibition might be partially dependent on calpain activity.

**Figure 4:**
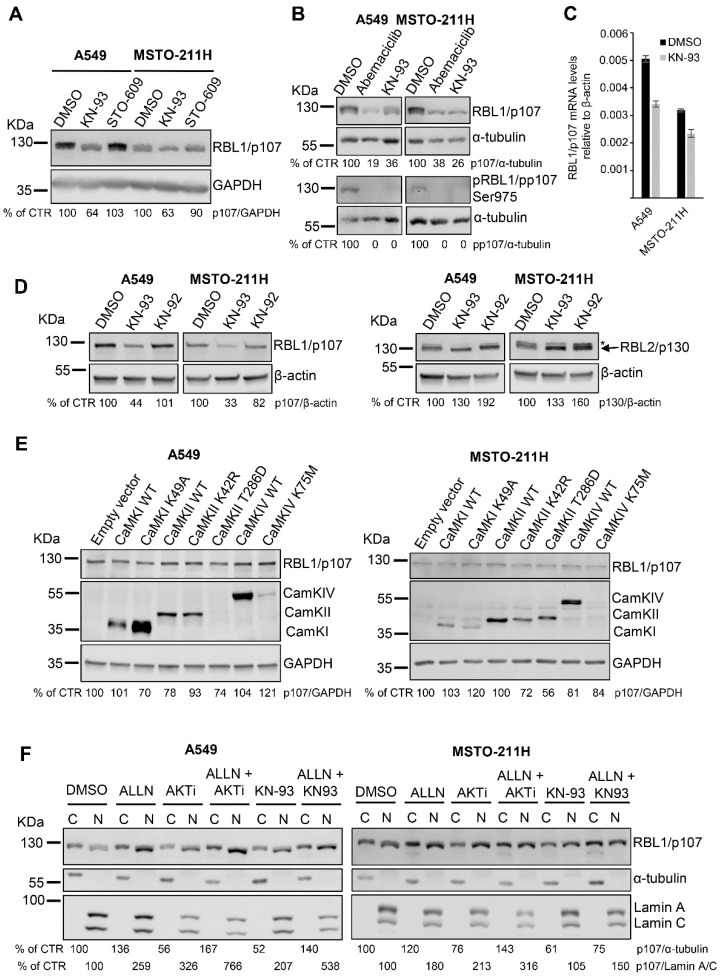
The CaMK inhibitor KN-93 reduces RBL1/p107 levels in A549 and MSTO-211H cells. (**A**). A549 and MSTO211H cells were treated for 16 h with the CaMK inhibitor KN-93 (20 µM) or the CaMKK inhibitor ST-609 (20 µM) and RBL1/p107 levels assessed by immunoblot. (**B**). A549 and MSTO-211H cells were treated with 0.2 µM Abemaciclib or 20 µM KN-93 for 16 h. Levels of total and phosphorylated RBL1/p107 (Ser975) were analyzed by immunoblot. (**C**). mRNA levels of RBL1/p107 relative to β-actin were assessed by qPCR in A549 and MSTO-211H treated with 20 µM KN-93 or control for 16 h. Mean ± SD. (**D**). Protein levels of RBL1/p107 and RBL2/p130 were analyzed in A549 and MSTO211H cells treated with the CaMK inhibitor KN-93 (20 µM) or with the inactive derivative of KN-93, KN-92 (20 µM), for 16 h. * Indicates an unspecific band recognized by the anti-RBL2/p130 antibody. (**E**). A549 and MSTO-211H cells were transiently transfected with plasmids encoding wild type (WT) CaMKI, CaMKI kinase-inactive mutant K49A, WT CaMKII, CaMKII kinase-inactive mutant K42R, CaMKII constitutively active mutant T286D, WT CaMKIV, CaMKIV kinase-inactive mutant K75M, or an empty vector used as control. 6 h post transfection, cells were transferred in serum free-media and incubated for additional 24 h. Cells were then serum-stimulated for 16 h. RBL1/p107 levels were analyzed from cell lysates by immunoblot. CamKII was detected using an anti-CaMKII antibody, while CamKI and CaMKIV were assessed using an anti-c-myc tag antibody. (**F**). RBL1/p107 levels were analyzed in cytoplasmic (C) and nuclear (N) fractions of A549 and MSTO-211H cells pre-incubated with the calpain inhibitor ALLN (100 µM) or a solvent control for 1 h and then incubated for 16 h with 100 µM ALLN, 12.5 µM AKTi VIII, 20 µM KN-93 alone or the indicated combination. Quantification was performed by densitometric analysis using the ImageJ program and expressed as % of control. All experiments were performed three times.

Because the inhibitor AKTi VIII might also affect the activity of the calcium-dependent protein kinase (CaMK)1α [38] and LY294002 and Wortmannin might differ in their abilities to interfere with Ca^2+^ levels [39], we hypothesized that the observed reduction in RBL1/p107 levels might be derived from the inhibition of the CaMKIα cascade rather than AKT. Thus, we investigated a potential role of CaMKIα signaling in modulating RBL1/p107, by exposing both A549 and MSTO-211H cells to the CaMK inhibitor KN-93 and to the CaMKK inhibitor ST-609. Notably, KN-93 but not ST-609 decreased RBL1/p107 protein levels as well as RBL1/p107 phosphorylation (Figure 4A,B) and led to a reduction in mRNA levels of RBL1/p107 of ~30% (Figure 4C). Since CaMKIs and CaMKIVs depend on CaMKK activity for their activation whereas CaMKIIs do not [22,23,24,40,41,42,43], these results point out a putative role for CaMKs and more likely CaMKIIs in modulating RBL1/p107 levels. Significantly, the effect of KN-93 on RBL1/p107 was specific as in fact KN-92, an inactive derivative of KN-93, had no effect in modulating RBL1/p107 levels (Figure 4D). Notably, the effect of KN-93 was specific for RBL1/p107 as it did not affect the levels of RBL2/p130 (Figure 4D), indicating that the CaMK signaling cascade might specifically regulate RBL1/p107 levels and not the closely related RBL2/p130.

To identify the putative CaMK responsible for KN-93-dependent effects on RBL1/p107 levels, we transiently overexpressed in A549 and MSTO-211H cells different members of the CaMK family, including wild type CaMKIα, CaMKIIα and CaMKIV and their respective kinase-inactive mutants (CaMKIα K49A, CaMKIIα K42R, CaMKIV K75M) as well as the constitutive active mutant of CaMKIIα (CaMKIIα T286D).

We selected CaMKIα because an AKTi VIII inhibitory activity on CaMKIα has been already described [38]. In addition, previously published work has reported that CaMKIα might play a role in regulating cell cycle and RB1/p105 activity [26]. On the other hand, we selected CaMKIIα based on the results obtained with the inhibitors KN-93 and STO-609 and on its known role as a cell cycle regulator [27,44]. Lastly, we chose CaMKIV because it might modulate the activity of the other CaMKs, thereby indirectly affecting cell cycle progression [45]. We reached considerable transient expression of all transfected constructs with the only exception of CaMKIIα T286D and CaMKIV K75M, which were expressed at low levels, as demonstrated by immunoblots with specific antibodies (Figure 4E). However, none of the expressed CaMKs affected RBL1/p107 levels, suggesting a complex and possibly redundant role for multiple CaMKs in modulating RBL1/p107 (Figure 4E).

Because KN-93 as AKTi VIII reduced the levels of RBL1/p107 and affected the relative amount of hyperphosphorylated RBL1/p107, we assessed the effect of these inhibitors on the subcellular distribution of RBL1/p107, as in fact RBL1/p107 cytoplasmic/nuclear distribution is dependent on the status of RBL1/p107 phosphorylation. As shown in Figure 4F, both inhibitors decreased the levels of cytoplasmic RBL1/p107 and relatively enhanced the levels of nuclear RBL1/p107 in cell fractions from A549 and MSTO-211H cells. This result reflects the observed reduction of RBL1/p107 levels and hypophosphorylation of RBL1/p107 in AKTi VIII- and KN-93-treated cells. Notably, in both cases, the reduction of cytosolic RBL1/p107 levels was counteracted by the combined inhibition of calpain while the increase of nuclear RBL1/p107 was enhanced in cells treated with the combination of AKTi VIII/ALLN or KN-93/ALNN when compared to cells treated with the inhibitors alone. These results suggest a complex regulation of RBL1/p107 levels/activity and cellular distribution involving the action of multiple CaMKs and calpain.

**Figure 5:**
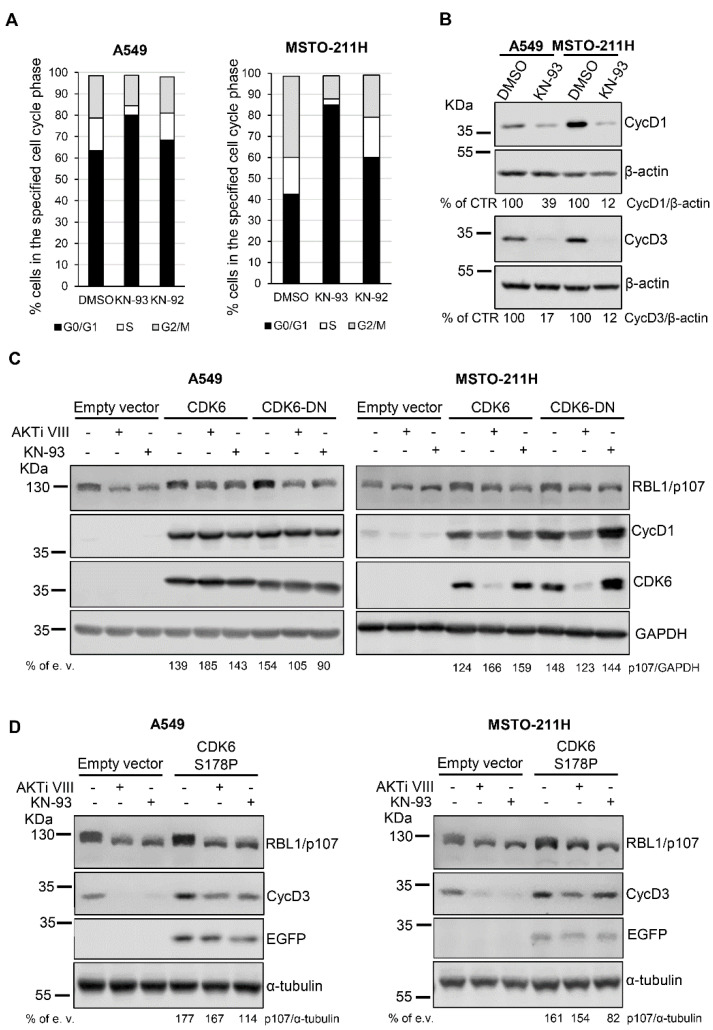
KN-93 effect on cell cycle of A549 and MSTO-211H cells. (**A**). The cell cycle was analyzed by FACS in A549 and MSTO-211H cells treated with 20 µM KN-93 or 20 µM KN-92 for 16 h. (**B**). Protein levels of cyclin D1 (CycD1) and cyclin D3 (CycD3) were assessed in A549 and MSTO-211H cells treated for 16 h with 20 µM KN-93. (**C**). A549 and MSTO-211H cells were co-transfected with plasmids coding for cyclin D1, CDK6-HA, CDK6-DN-HA or an empty vector as control and then treated with 12.5 µM AKTi VIII or 20 µM KN-93 for 16 h. RBL1/p107 levels were assessed by immunoblot. CDK6 was detected using an anti-HA antibody. (**D**). RBL1/p107 protein levels in A549 and MSTO-211H cells transduced with a lentivirus expressing CDK6 S178P or control lentivirus and transfected with plasmids coding for cyclin D3 or empty vector and then treated with 12.5 µM AKTi VIII or 20 µM KN-93 for 16 h. The expression of exogenous CDK6 S178P was monitored by detecting enhanced green fluorescent protein (EGFP), encoded by the bicistronic IRES pHAGE vector. Quantification was performed by densitometric analysis using the ImageJ program and expressed as % of CTR (B) or, for each treatment condition, as % empty vector (e. v.)-transfected respective control cells. All experiments were performed three times.

Because it has been previously reported that KN-93 might affect the cell cycle of several cell types [27] by possibly interfering with G1/S-specific cyclin-CDK complexes thereby promoting a G0/G1 arrest [26], we investigated the effect of KN-93 on the cell cycle of A549 and MSTO-211H cells. As shown in Figure 5A, KN-93 but not KN-92 led to an increase in the fraction of cells in the G0/G1 phase, suggesting a KN-93-evoked cell cycle arrest in G0/G1. In agreement, cells treated with KN-93 showed reduced levels of G1/S-specific cyclin D1 and cyclin D3 (Figure 5B). Notably, a similar effect on cell cycle, i.e., arrest in G0/G1, was previously demonstrated for the AKT inhibitor AKTi VIII in the same cellular models [18]. These results suggest that the effect of AKTi VIII and KN-93 on RBL1/p107 levels/phosphorylation might be associated with the inhibitory effect of the inhibitors on cell cycle progression. Accordingly, transient co-expression of cyclin D1 and CDK6 in A549 and MSTO-211H cells increased the basal levels of RBL1/p107 and partially reverted AKTi VIII and KN-93 actions on RBL1/p107 levels and phosphorylation (Figure 5C). The overexpression of cyclin D1 and of a dominant negative (DN) mutant of CDK6 mostly increased basal level of RBL1/p107, but did not compensate the inhibitory effect of AKTiVIII and KN-93 in A549 cells, whereas it partially compensated the effect of the two inhibitors in MSTO-211H cells, suggesting that in this cell line the forced expression of cyclin D1 might be enough to compensate the effects of the two inhibitors on RBL1/p107.

Similarly, the co-expression of cyclin D3 and of a constitutive active CDK6 mutant, CDK6 S178P [46], increased the basal level of RBL1/p107 and partially counteracted the effect of AKTi VIII and KN-93 on RBL1/p107 levels and phosphorylation in A549 cells (Figure 5D, left panel). In MSTO-211H cells, cyclin D3 and CDK6 S178P co-expression increased RBL1/p107 basal levels and partially compensated the effect of the AKTi VIII inhibitor (Figure 5D, right panel). These results suggest that the effects of AKTi VIII and KN-93 on RBL1/p107 levels and phosphorylation might be indirect and linked to reduced activity of G1/S cyclins/CDK complexes associated with cell cycle arrest in G0/G1 phase (Figure 5D).

## 4. Discussion

The three members of the RB family, RB1, RBL1/p107 and RBL2/p130, are key regulators of the cell cycle [47] and their dysregulation is strongly associated with tumor initiation and progression [1,48]. RB proteins restrain the cell cycle and inhibit the G1/S phase transition by interacting with the E2F transcription factors or forming the DREAM complex [2,17]. RB1/p105 binds and inhibits the transcriptional activators E2F1, E2F2 and E2F3, whereas RBL1/p107 and RBL2/p130 recruit the transcriptional repressors E2F4 and E2F5 to the nucleus. Binding of RB proteins to the E2F transcription factors is modulated by RB protein phosphorylation. Upon phosphorylation mediated by cyclin-CDK complexes, E2F factors are released with the consequent expression of genes regulating cell cycle progression. In addition to regulating the cell cycle, there is growing evidence suggesting that RB proteins might modulate other processes whose dysregulation is associated with tumorigenesis, such as chromosomal stability, cell senescence, apoptosis, and cell differentiation [4,49,50,51]. Thus, it is pivotal to better understand how RB protein levels/activities are modulated and the role that oncogenic pathways play in this process.

RBL1/p107 is phosphorylated by the cyclinD-CDK4/CDK6 complex [9,34] and its phosphorylation status is dependent on the dynamic equilibrium between the activity of cyclinD-CDK4/CDK6 complexes and the action of phosphatase 2A (PP2A) [34,52,53,54,55,56]. In spite of many indications supporting the notion that RBL1/p107 is mainly regulated on a transcriptional level [57], there are also evidences demonstrating that RBL1/p107 levels are modulated post-translationally. Indeed, RBL1/p107 protein degradation has been shown to occur in both a calpain- and proteasome-dependent manner [36,37]. Notably, Sengupta et al. identified a C-terminal sequence in RBL1/p107 whose phosphorylation by CDK4/CDK6 prevents proteasomal-dependent degradation of RBL1/p107 [58]. Here, we made the novel observation that in lung cancer and mesothelioma cells, RBL1/p107 levels and phosphorylation are modulated by a complex network of signaling pathways including CaMKs and calpain, the activity of which is Ca^2+^-dependent [21,59,60].

Because recent work from our laboratories demonstrated that RBL2/p130 is a direct target of AKT and AKT-dependent phosphorylation of RBL2/p130 is crucial in regulating RBL2/p130 stability [18], we initially sought to determine whether the AKT signaling pathway might play a similar role in regulating the stability/activity of the closely related RBL1/p107. The results of initial experiments with the AKT inhibitor AKTi VIII suggested a role of AKT in modulating RBL1/p107 levels/activity in lung cancer and mesothelioma cells, as in fact AKT inhibition affected RBL1/p107 levels as well as phosphorylation. However, the results of additional experiments ruled against a direct major role of AKT in regulating RBL1/p107 levels in these models. First, AKT activation by cell stimulation with different growth factors, including EGF, IGF-2, insulin (data not shown) and TGF-beta (data not shown), both in early as well as in chronic exposure regimens, did not affect RBL1/p107 levels, suggesting that RBL1/p107 stability is not strictly dependent on signals triggered by growth factors, whose action in regulating RBL1/p107 protein levels is still controversial [61,62]. Accordingly, RBL1/p107 levels were not significantly affected by additional growth factor-evoked signaling pathways, such as the ERK1/2 and the p38 signaling cascades. Second, the potential role of AKT in modulating RBL1/p107 was further challenged by the observation that additional PI3K inhibitors, such as LY294002 and Wortmannin, were inconsistent in reducing RBL1/p107 levels. More importantly, AKT1 depletion by siRNA approaches or exogenous expression of constitutively active AKT1 and AKT2 proteins had only minor effects in modulating RBL1/p107 levels/phosphorylation, suggesting that the mechanisms regulating RBL1/p107 stability are not directly dependent on AKT activation and considerably differ from the regulation of RBL2/p130 action.

Because the AKT inhibitor AKTi VIII might also inhibit CaMKIα [38] and LY294002, but not wortmannin, might affect intracellular Ca^2+^ levels [39], we then hypothesized that the effect of these inhibitors on RBL1/p107 protein levels might have actually worked by modulating Ca^2+^-dependent networks and CaMKs signaling. Indeed, cell exposure to the CaMK inhibitor KN-93, but not the inactive derivative KN-92, reduced RBL1/p107 phosphorylation and levels supporting a previously uncharacterized role of this family of protein kinases in modulating RBL1/p107 expression.

Several studies have previously demonstrated a role for Ca^2+^/calmodulin-dependent pathways, particularly the multifunctional protein family of CaMKs, in cell cycle regulation [26,27]. CaMKI is an important modulator of the G1 phase, whereas CaMKII plays an important role in regulating the G2/M and the metaphase/anaphase transitions [27]. In addition, there are many reports demonstrating that the CaMK inhibitor KN-93 might arrest the cell cycle and lead to G1 block [27]. Reports suggest that CaMKs inhibition might modulate the cell cycle by lowering cyclin D1 levels [63,64], reducing the activity of CDK2 and CDK4 [26,63], affecting the levels of p21 [44] or p27 [63] and negatively modulating the phosphorylation of RB1/p105 [44,63,64]. Our results suggest that CaMKs might affect RBL1/p107 function at multiple levels, as in fact cell exposure to KN-93 reduced both levels and phosphorylation of RBL1/p107 protein. Significantly, RBL2/p130 levels were not affected by CaMK inhibition, demonstrating a different regulatory mechanism for the two related RB proteins and that CaMKs might specifically regulate RBL1/p107 but not RBL2/p130.

In order to identify the specific CaMK responsible for the regulation of RBL1/p107 levels, we transiently expressed several CaMK proteins, including the CaMKI K49A mutant, which was previously reported to control RB1/p105 phosphorylation [26]. However, we were not able to identify a single CaMK activity directly affecting RBL1/p107 levels, suggesting a more complex regulation of RBL1/p107 levels, which might rely on the action of multiple redundant CaMKs.

**Figure 6:**
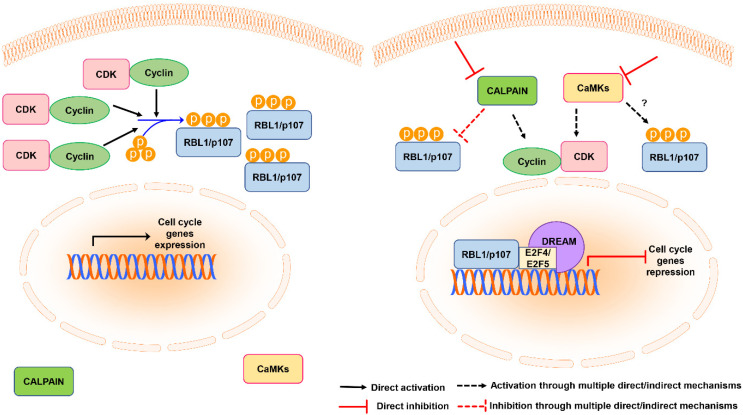
Schematic representation of the model of RBL1/p107 regulation by calpain and CaMKs. In cycling cells RBL1/p107 is phosphorylated by cyclin-CDK complexes, promoting RBL1/p107 cytoplasmic localization (**left** panel). Calpain inhibition or CaMK inhibition promote RBL1/p107 dephosphorylation and nuclear accumulation by directly or indirectly affecting its stability or the activity/levels of cyclins and CDKs. RBL1/p107 nuclear accumulation might increase the localization of transcriptional repressors at the promoters of genes controlling cell cycle progression, contributing to cell cycle arrest (**right** panel).

AKTi VIII and KN-93 inhibitors not only decreased the total levels of RBL1/p107, but also increased the levels of nuclear RBL1/p107, which is consistent with a reduction in RBL1/p107 phosphorylation. Interestingly, the inhibition of the calcium-dependent endopeptidase calpain counteracted the reduction in the cytoplasmic levels caused by AKTi VIII or KN-93 and increased the fraction of nuclear RBL1/p107, suggesting that another Ca^2+^-regulated protein might contribute to the regulation of RBL1/p107 protein levels. Previously published data have indicated a possible role for calpain in controlling RBL1/p107 protein degradation [36,37], thereby suggesting that the effect of CaMK inhibition on RBL1/p107 levels might be connected to calpain-dependent protein degradation. In addition, several studies indicated that calpain activity can modulate the cell cycle by affecting cyclins levels and CDK activity and promoting RB1/p105 hyperphosphorylation [65]. Since RBL1/p107 activity, including the regulation of gene expression and CDK activity or the modulation of chromatin packing, might also rely on RBL1/p107 subcellular localization [5,66], these data suggest that calpain might modulate RBL1/p107 functions by interfering with its nuclear-cytoplasmic distribution. The nuclear function of RBL1/p107 is not fully elucidated. However, it has been suggested that RBL1/p107 can bind to the repressor E2F4, forming DREAM-like complexes [2] and might contribute to the repression of both G1/S and G2/M genes [16]. An increase in nuclear and hypophosphorylated RBL1/p107 might contribute to enhance RBL1/p107 binding to transcriptional repressors and their localization at promoters of G1/S genes or G2/M genes (Figure 6).

We observed that KN93 induced a cell cycle block and enriched the cells in the G0/G1 phase associated with a reduction in cyclin D1 and cyclin D3 levels. These results are in agreement with data reported in other cellular models [27] and suggest a possible scenario where the observed reduction in the total levels of RBL1/p107 and increase in its hypo-phosphorylated form might be in part due to cell cycle arrest in G0/G1, since in G0/G1 the levels and phosphorylation of RBL1/p107 decrease [67,68,69]. Accordingly, when we overexpressed cyclin D1 and CDK6 or cyclin D3 and a constitutively active mutant of CDK6, the effect of both AKTi VIII and KN93 on RBL1/p107 levels was partially reversed. Collectively, these data suggest that the effect of CaMK inhibition on RBL1/p107 could be partially secondary to the induction of cell cycle arrest in the G0/G1 phase. In turn, RBL1/p107 dephosphorylation and nuclear accumulation might contribute to the cell cycle arrest induced upon CaMK inhibition by repressing genes that modulate cell cycle progression (Figure 6).

## 5. Conclusions

In summary, in the present work we demonstrated that in lung cancer and mesothelioma cells, RBL1/p107 levels and phosphorylation are modulated by a complex network of functional interactions involving Ca^2+^-dependent signaling, CaMKs, calpain and their ability to directly or indirectly regulate the cell cycle, pointing out a substantial difference with the mechanisms regulating RBL2/p130 stability. Importantly, the identification of novel regulators of the tumor suppression function of RB proteins might open new strategies for therapy.

## Data Availability

The data presented in this study are available in this article and in supplemental material.

## Supplementary Materials

The following are available online at https://www.mdpi.com/article/10.3390/cancers13195025/s1. Table S1: Primers used in the study. The uncropped blots are shown in supplementary materials.
