# Supplementary material for: RBL1/p107 Expression Levels Are Modulated by Multiple Signaling Pathways
Source: Cancers (Basel). 2021 Oct 8;13(19):5025. doi: 10.3390/cancers13195025 (PMC8507926; doi:10.3390/cancers13195025)
Supplement: Supplementary file 1 [file cancers-13-05025-s001.zip › cancers-1328012-supplementary.pdf]

**Primer name**	**Primer sequence**
CaMKIα fwd	AATTCACCATGGCAATGCTGGGGGCAGTGGAAGGC
CaMKIα 1stNcoI mut fwd	CTTGCAGCACCCTTGGATTGC
CaMKIα 1stNcoI mut rev	GCAATCCAAGGGTGCTGCAAG
CaMKIα 2ndNcoI mut fwd	CGGCGAGCCACGGGAGCTGC
CaMKIα 2ndNcoI mut rev	GCAGCTCCCGTGGCTCGCCG
CaMKIα rev	CTCGCGGCCGCGTGAGCTGGTGGGGCAGTGTGGG
CaMKIα K49A fwd	GCTGGTGGCCATCGCATGCATTGCCAAGG
CaMKIα K49A rev	CCTTGGCAATGCATGCGATGGCCACCAGC
CaMKIIα fwd	AATTCACCATGGCCACCATCACCTGCACC
CaMKIIα rev	CTCGCGGCCGCTCAGTGGGGCAGGACGGAGGGCG
CaMKIIα T286D fwd	CACAGACAGGAGGACGTGGACTGCCTGAAG
CaMKIIα T286D rev	CTTCAGGCAGTCCACGTCCTCCTGTCTGTG
CaMKIIα K42R fwd	GGAGTATGCTGCCAGAATCATCAACACAAAG
CaMKIIα K42R rev	CTTTGTGTTGATGATTCTGGCAGCATACTCC
CaMKIV fwd	AATTCACCATGGCAATGCTCAAAGTCACGGTGCCC
CaMKIV NcoI mut fwd	CCAGCAGCAGTCATGGCAGCATCC
CaMKIV NcoI mut rev	GGATGCTGCCATGACTGCTGCTGG
CaMKIV rev	CTCGCGGCCGCGTGTACTCTGGCAGGATC
CaMKIV K75M fwd	TTATGCTCTCATGGTGTTAAAG
CaMKIV K75M rev	CTTTAACACCATGAGAGCATAA
RBL1/p107 fwd	GCAACTACAGCCTAGAGGGAG
RBL1/p107 rev	TGATACCCTTTCCAACCGTGG
β-actin fwd	CAGGGCGTGATGGTGGGC
β-actin rev	CTCGGTCAGCAGCACGG
CDK6 fwd	GCAGGCACCATGGAGAAGGACG
CDK6 S178P fwd	AGATGGCTCTAACC**C**CAGTGGTCGTCACGC
CDK6 S178P rev	GCGTGACGACCACTGGGGTTAGAGCCATCT
CDK6 rev	AGACTCGAGCGGCCGCCACTGTGC

Supplemental Table S1. Primers used in the study.

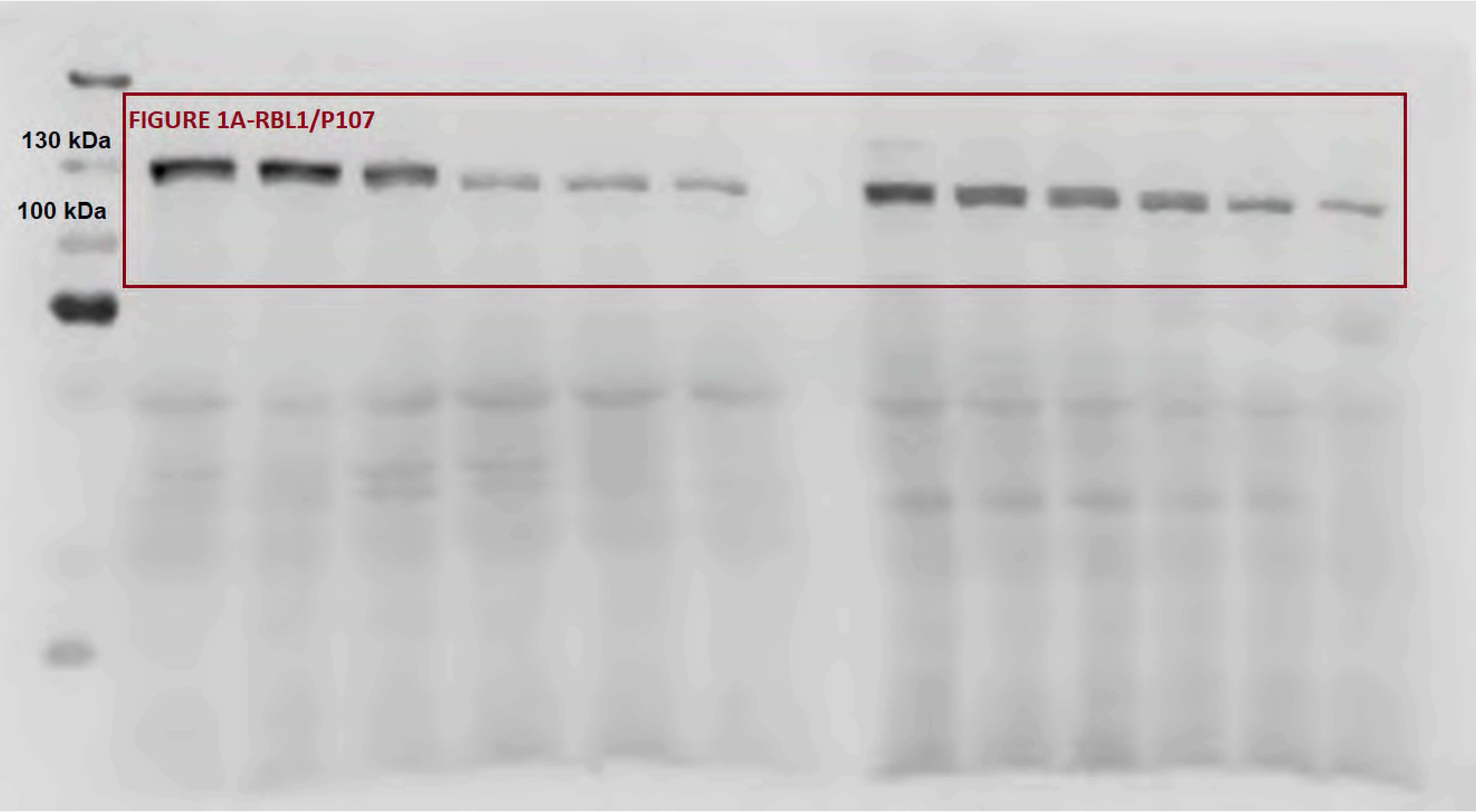

FIGURE 1A-RBL1/P107
130 kDa
100 kDa

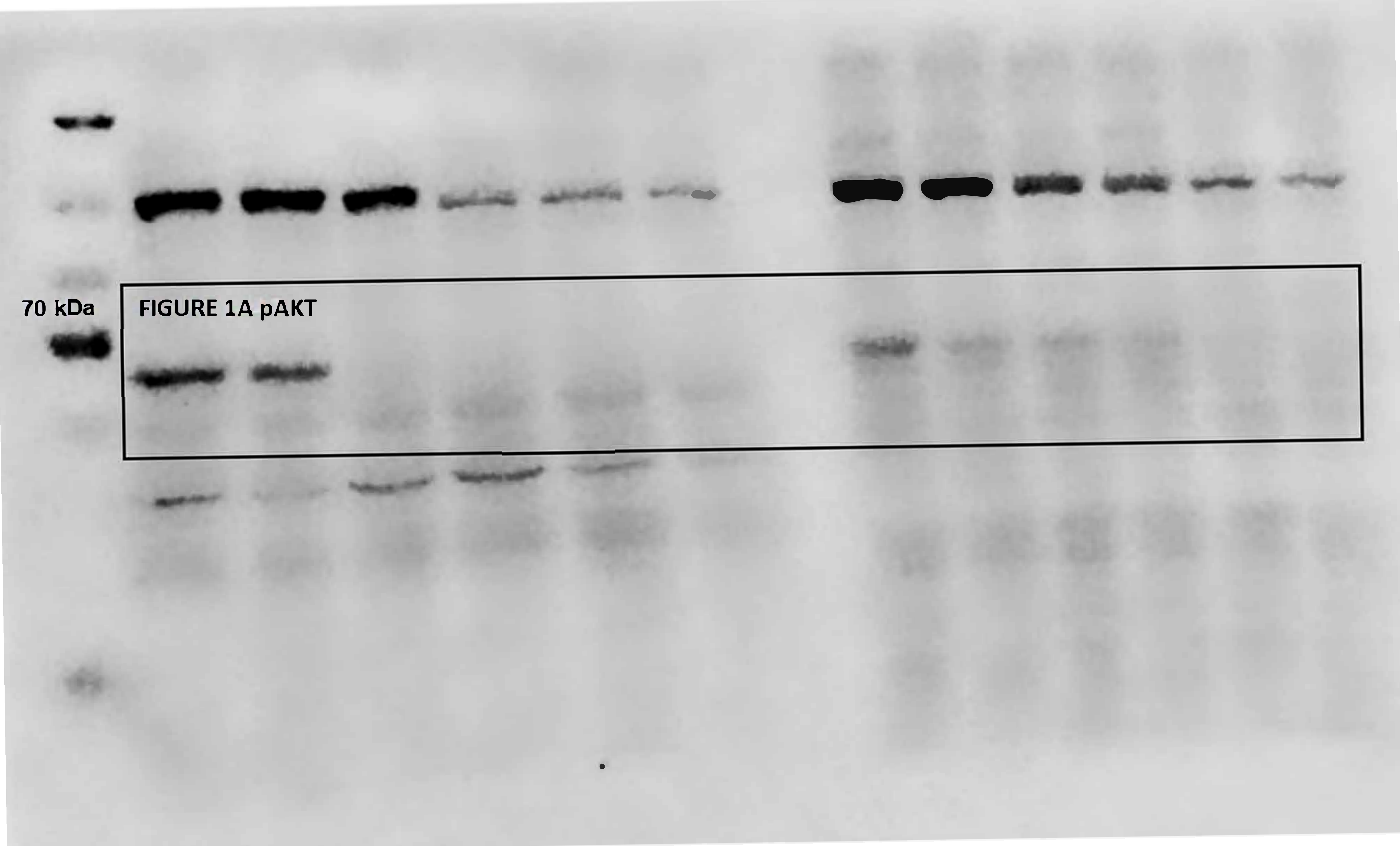

70 kDa
FIGURE 1A pAKT

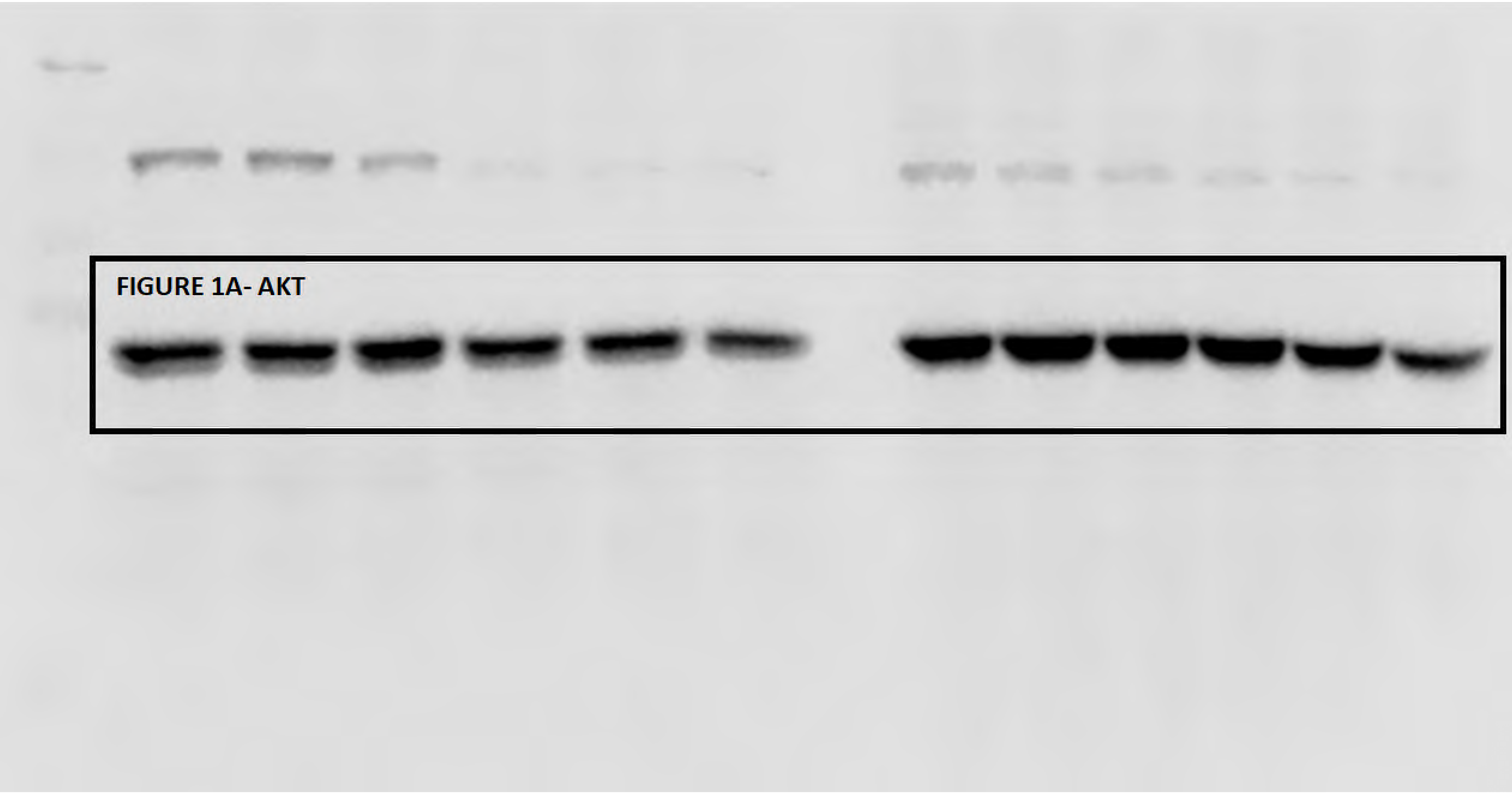
FIGURE 1A- AKT

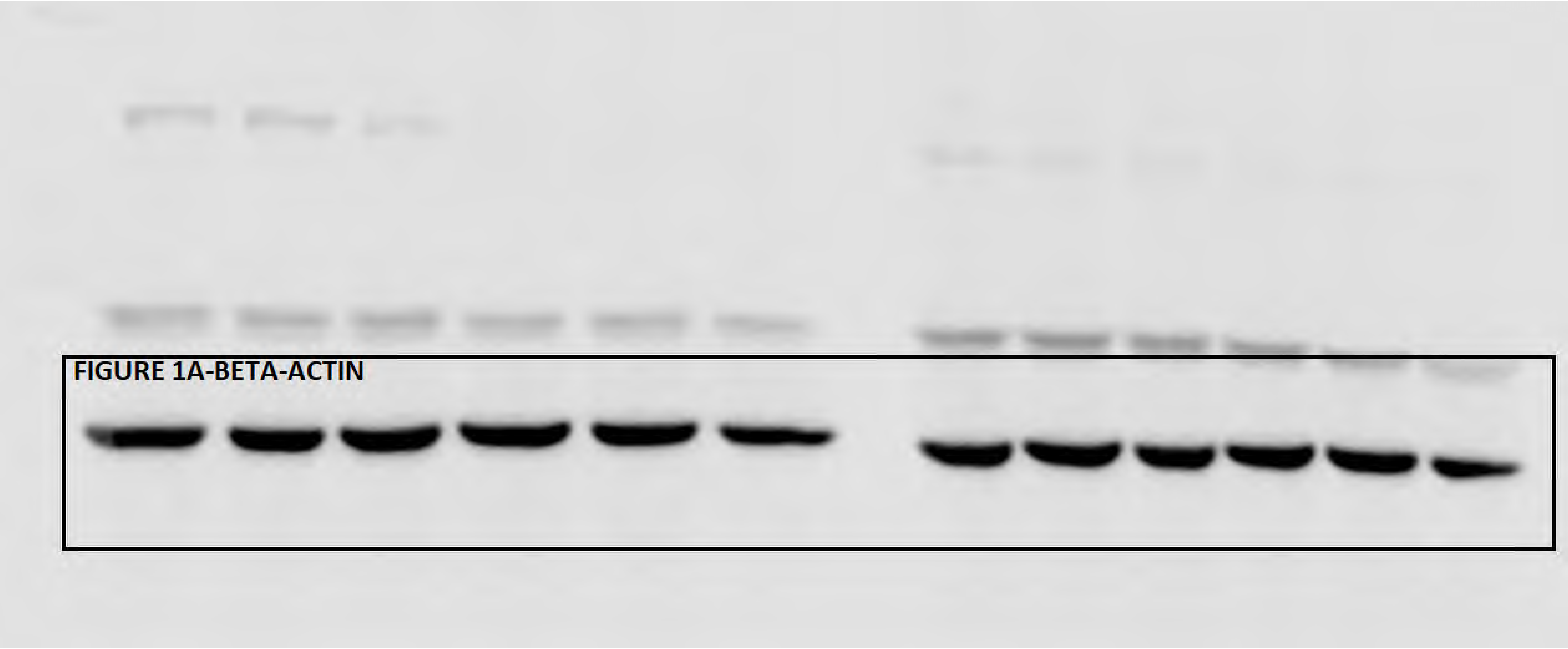

FIGURE 1A-BETA-ACTIN

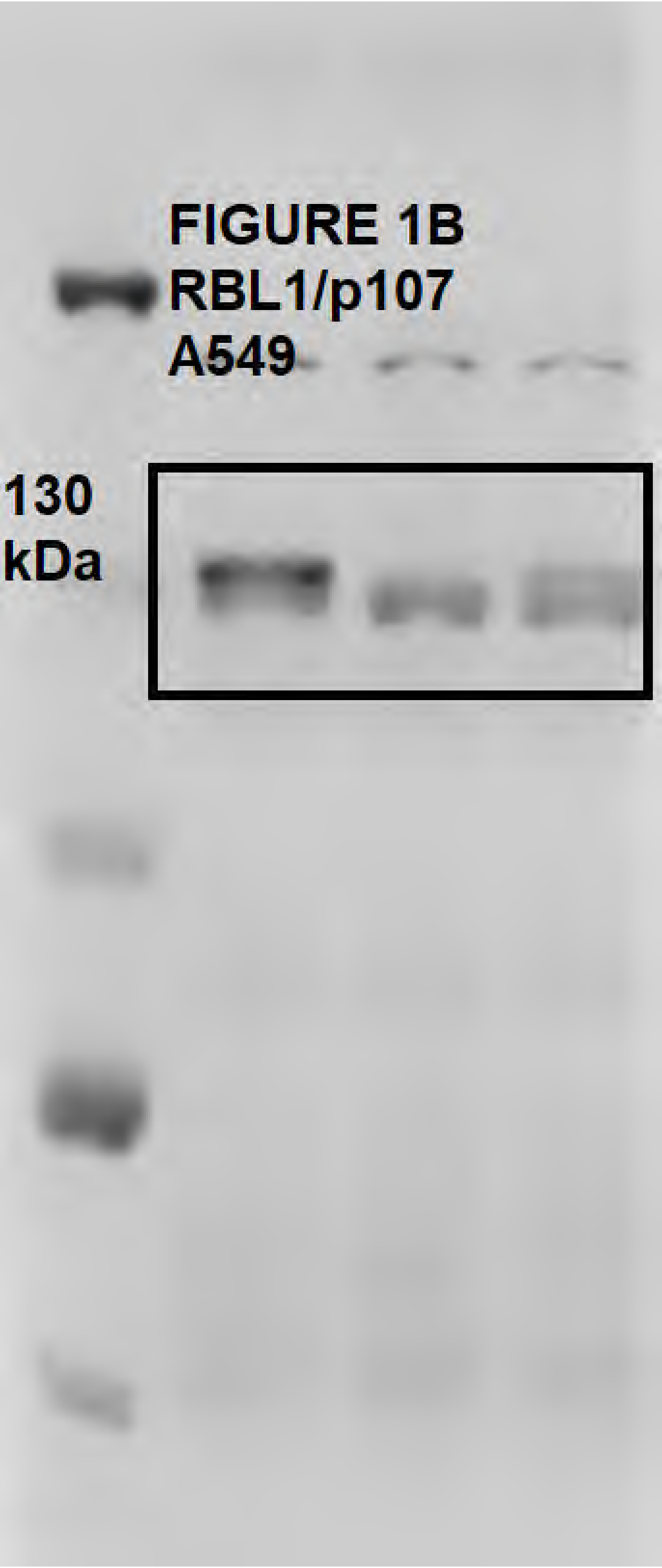
FIGURE 1B
RBL1/p107
A549
130
kDa

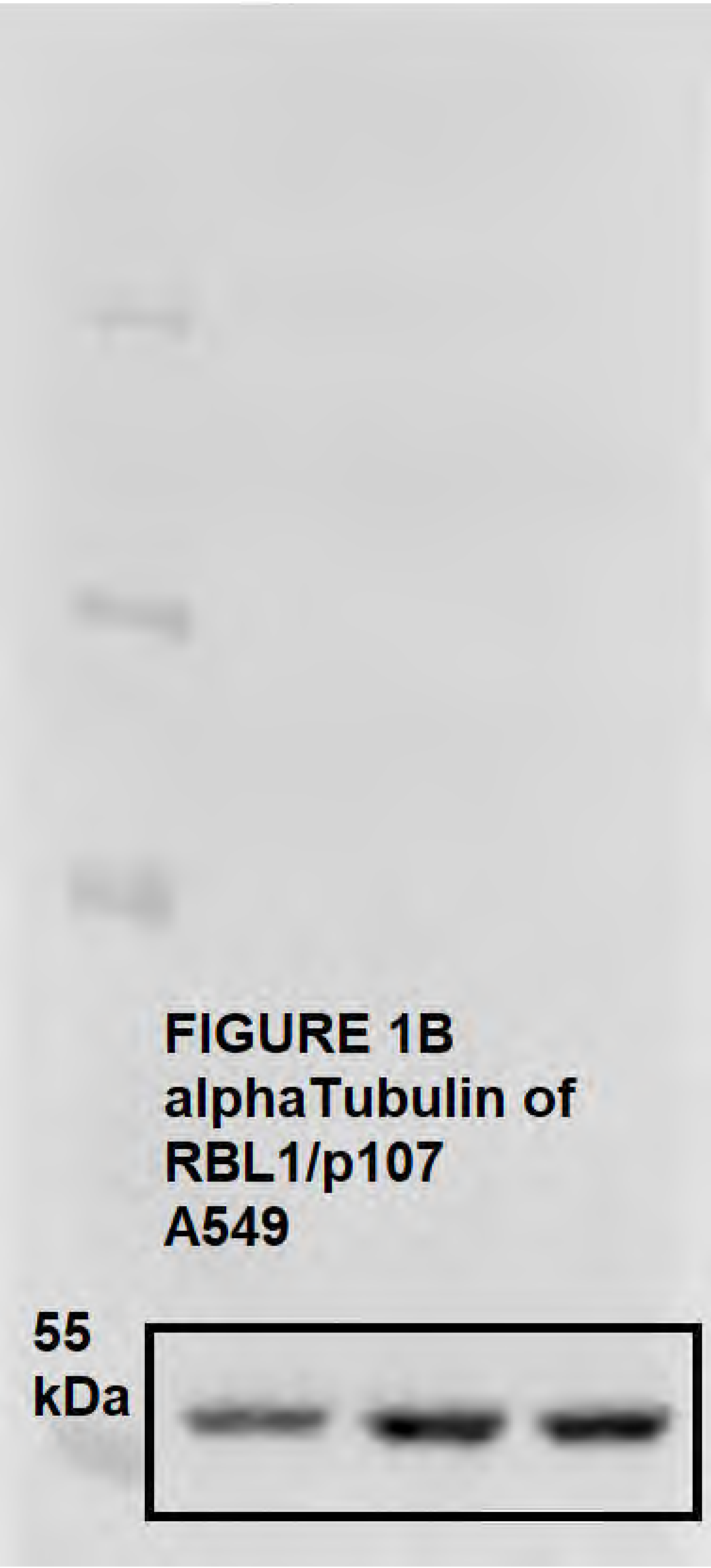

FIGURE 1B
alphaTubulin of
RBL1/p107
A549

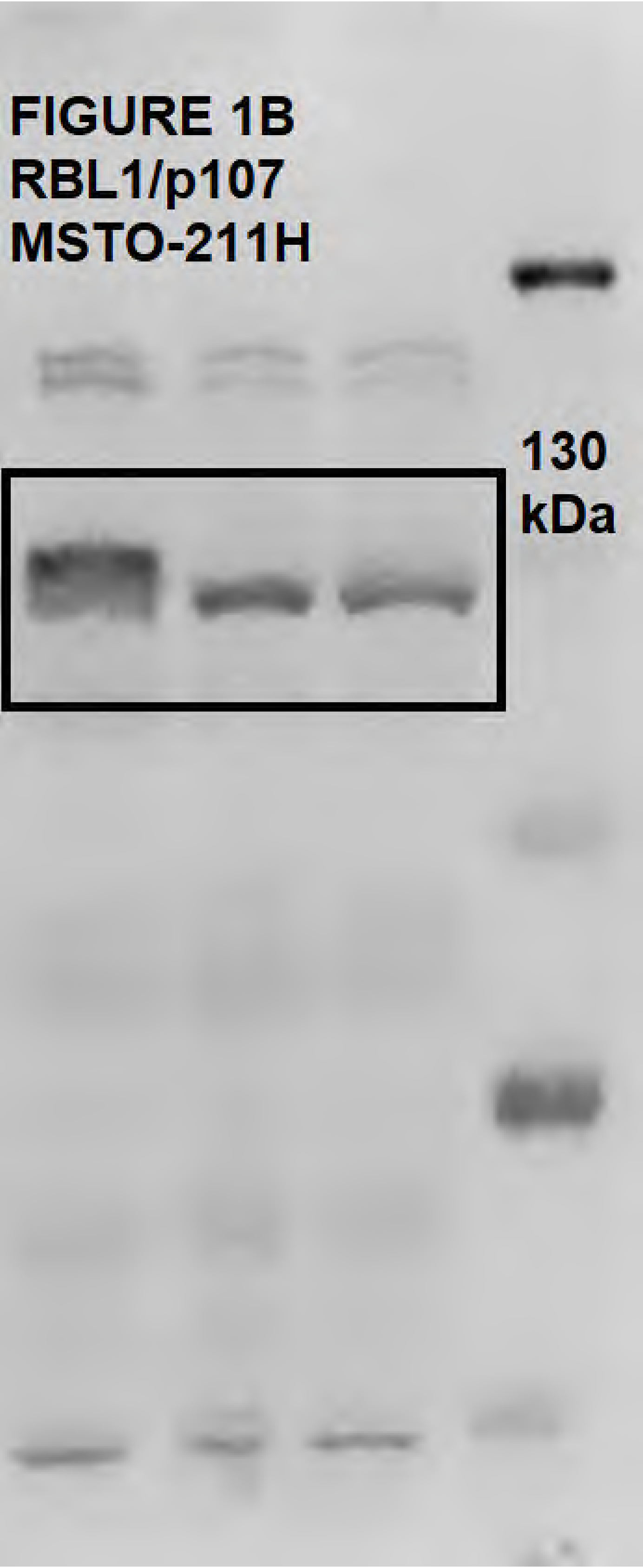
FIGURE 1B
RBL1/p107
MSTO-211H
130
kDa

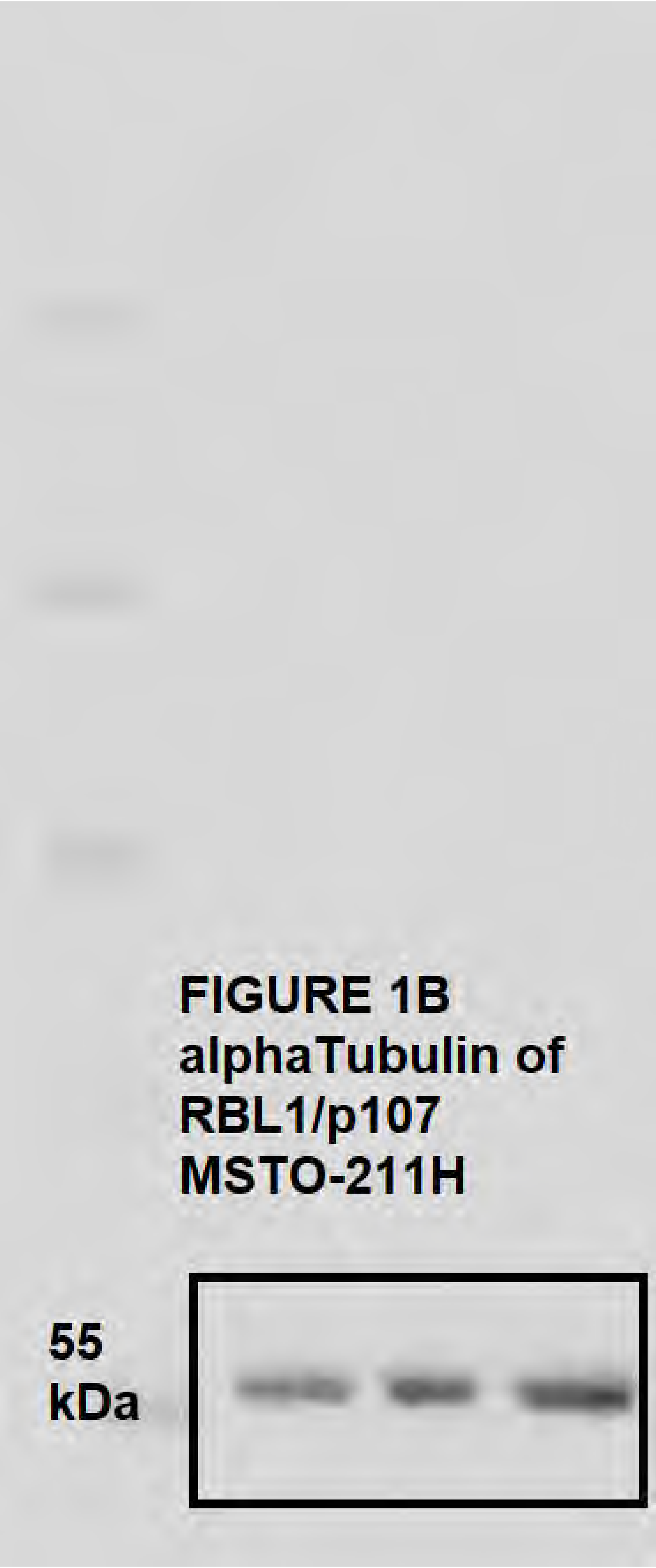

FIGURE 1B
alphaTubulin of
RBL1/p107
MSTO-211H

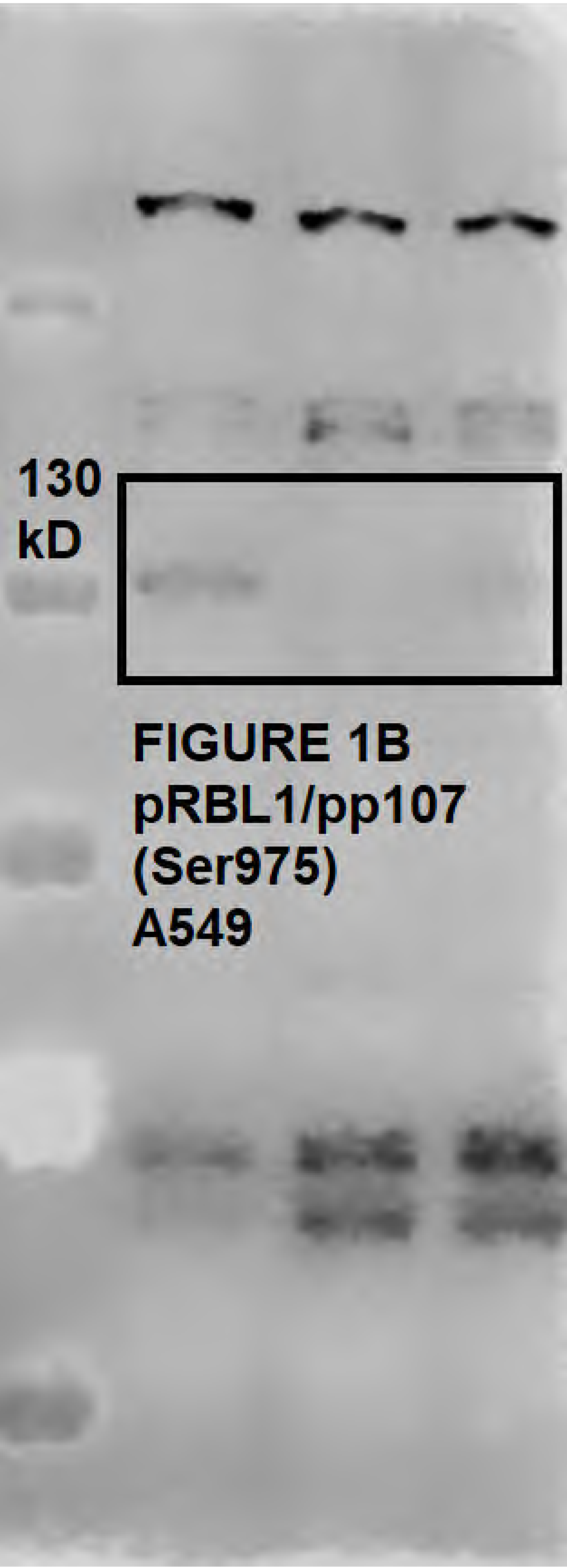

FIGURE 1B
pRBL1/pp107
(Ser975)
A549

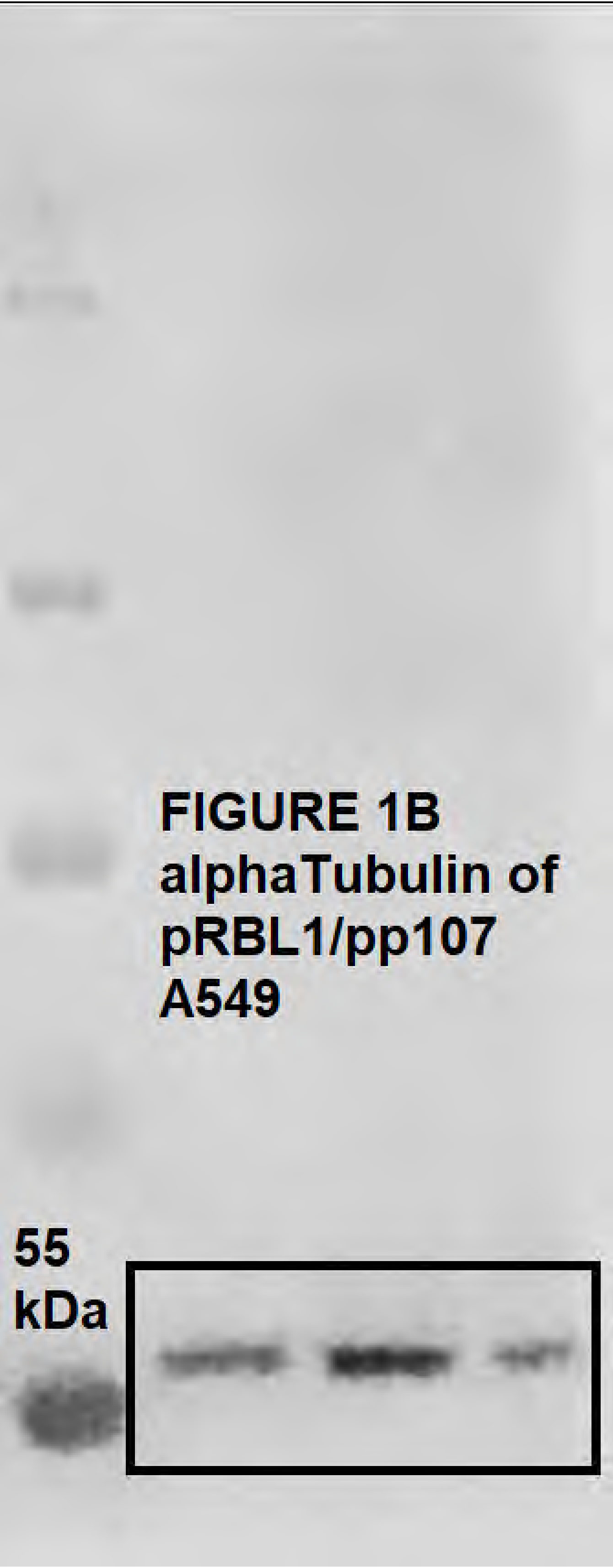

FIGURE 1B
alphaTubulin of
pRBL1/pp107
A549

FIGURE 1B
pRBL1/pp107
MSTO-211H

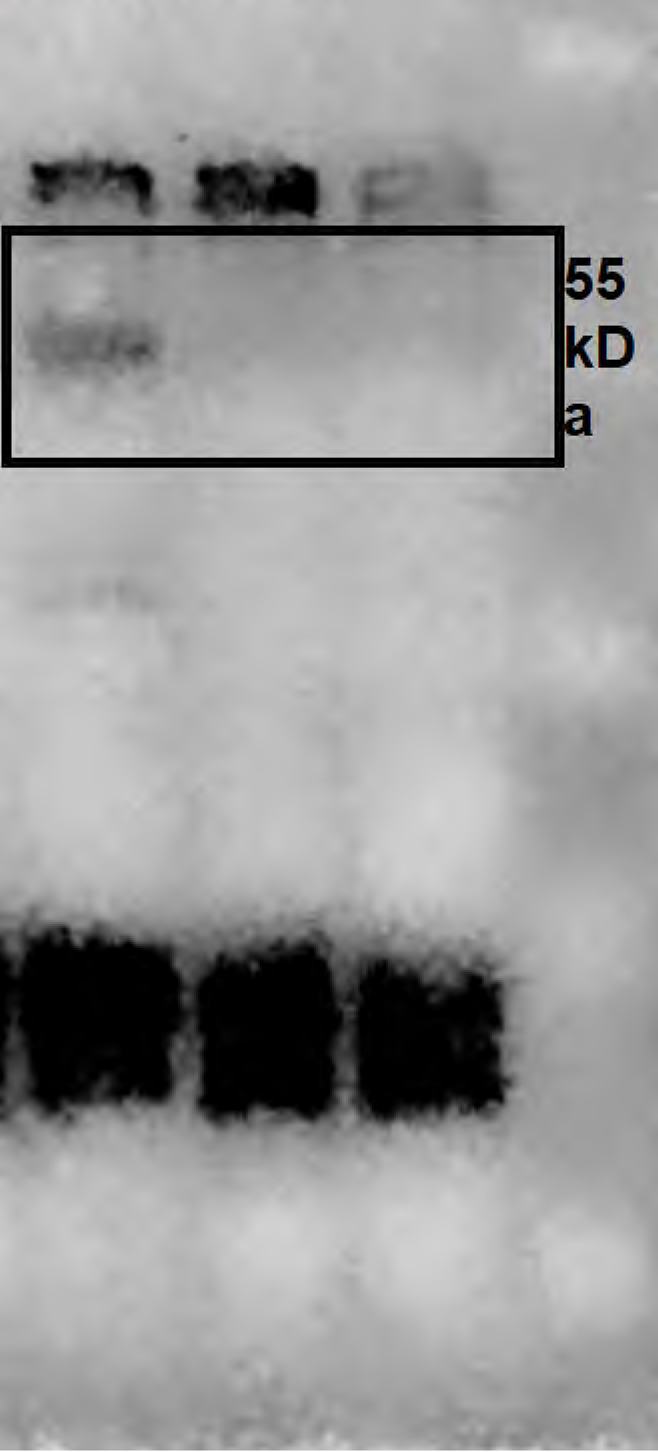

FIGURE 1B
alphaTubulin of
pRBL1/pp107
MSTO-211H

55
kDa

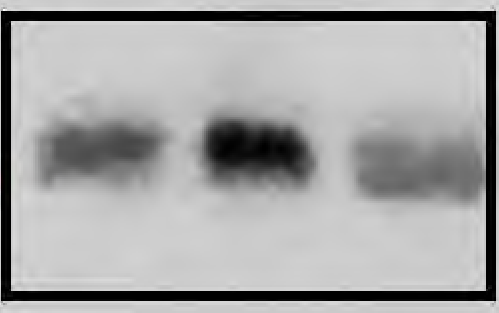

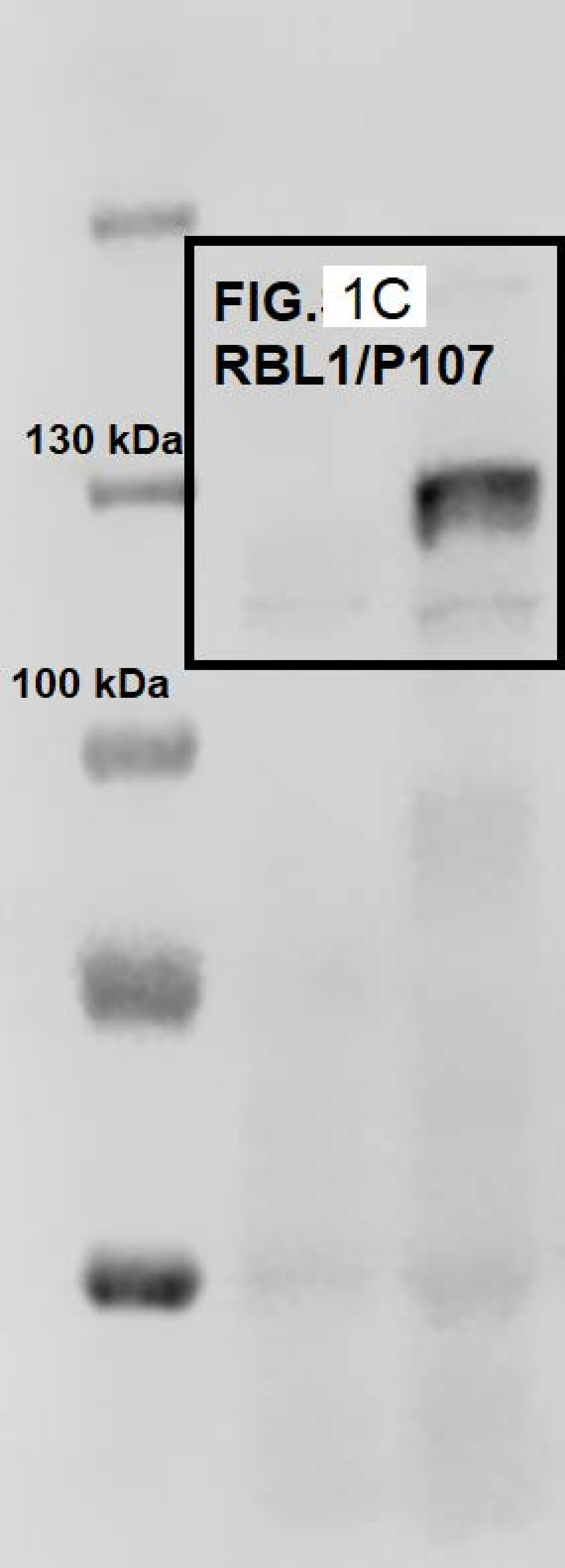
FIG. 1C
RBL1/P107
130 kDa
100 kDa

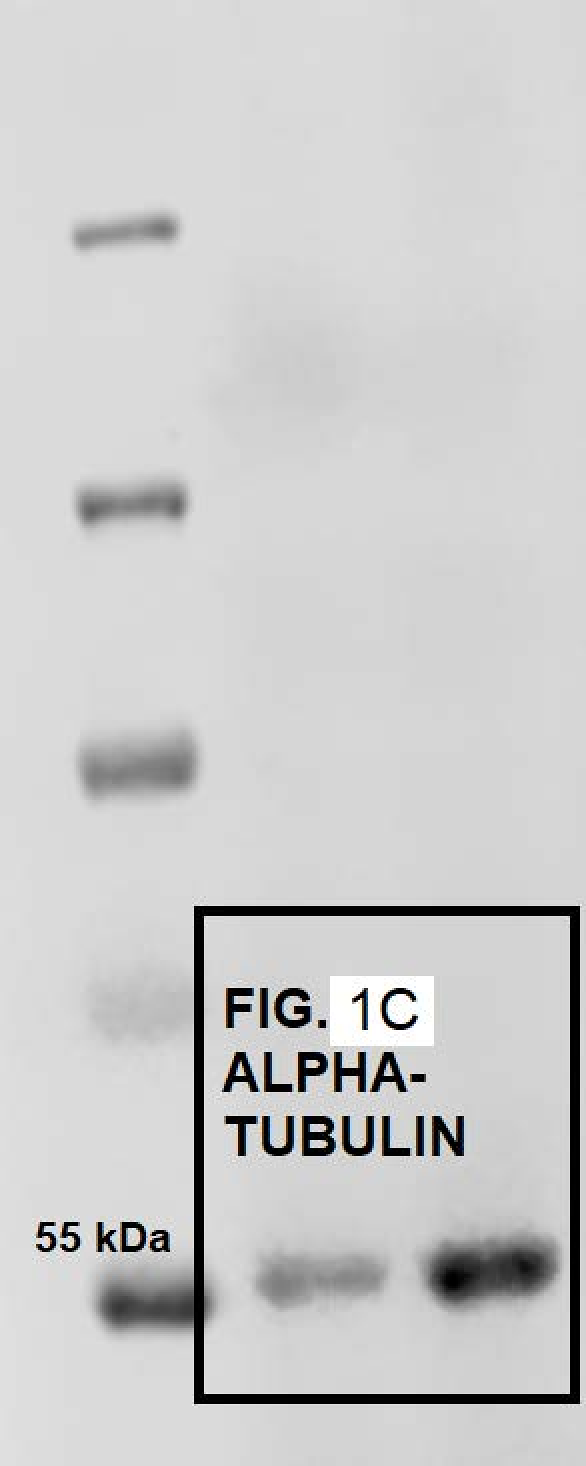
FIG. 1C
ALPHA-
TUBULIN
55 kDa

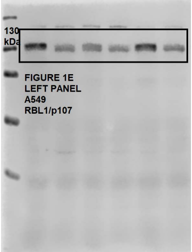
130
kDa
FIGURE 1E
LEFT PANEL
A549
RBL1/p107

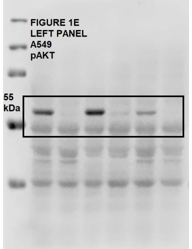
FIGURE 1E
LEFT PANEL
A549
pAKT
55
kDa

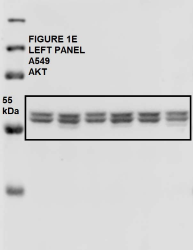
FIGURE 1E
LEFT PANEL
A549
AKT
55
kDa

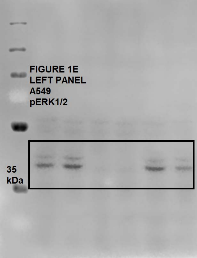
FIGURE 1E
LEFT PANEL
A549
pERK1/2
35
kDa

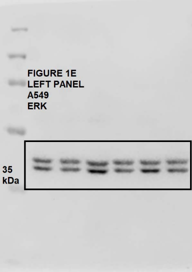
FIGURE 1E
LEFT PANEL
A549
ERK
35
kDa

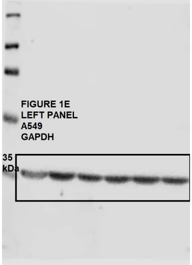
FIGURE 1E
LEFT PANEL
A549
GAPDH
35
kDa

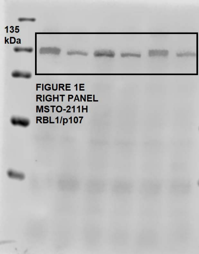

135
kDa
FIGURE 1E
RIGHT PANEL
MSTO-211H
RBL1/p107

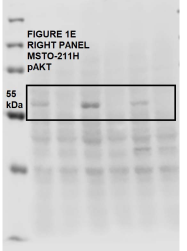
FIGURE 1E
RIGHT PANEL
MSTO-211H
pAKT
55
kDa

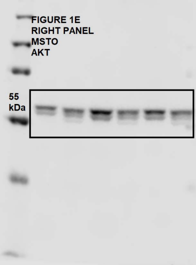
FIGURE 1E
RIGHT PANEL
MSTO
AKT
55
kDa

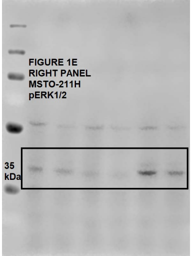
FIGURE 1E
RIGHT PANEL
MSTO-211H
pERK1/2
35
kDa

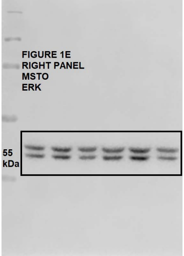

FIGURE 1E
RIGHT PANEL
MSTO
ERK
55
kDa

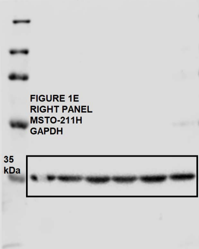
FIGURE 1E
RIGHT PANEL
MSTO-211H
GAPDH
35
kDa

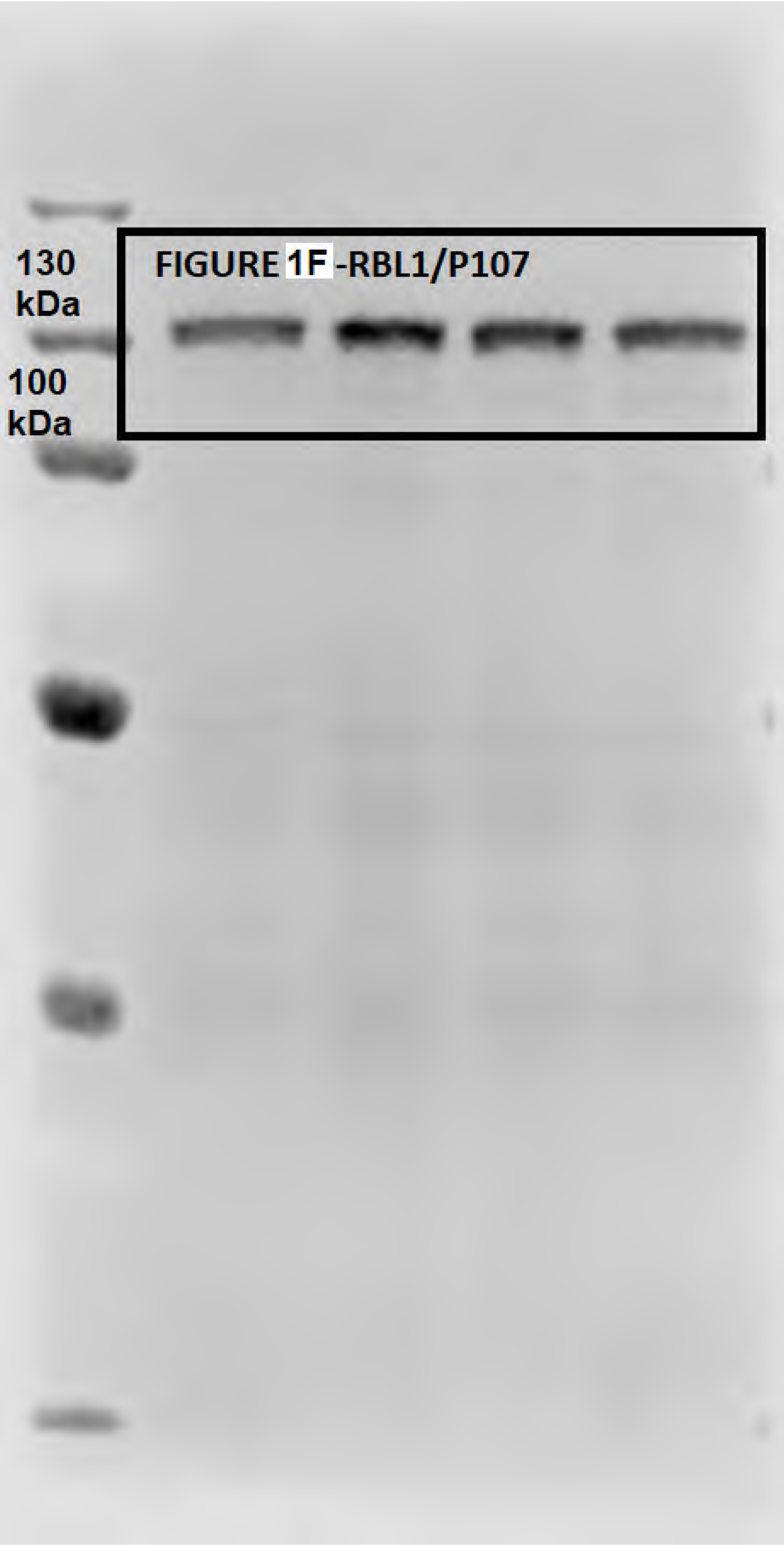
130 kDa
100 kDa
FIGURE 1F -RBL1/P107

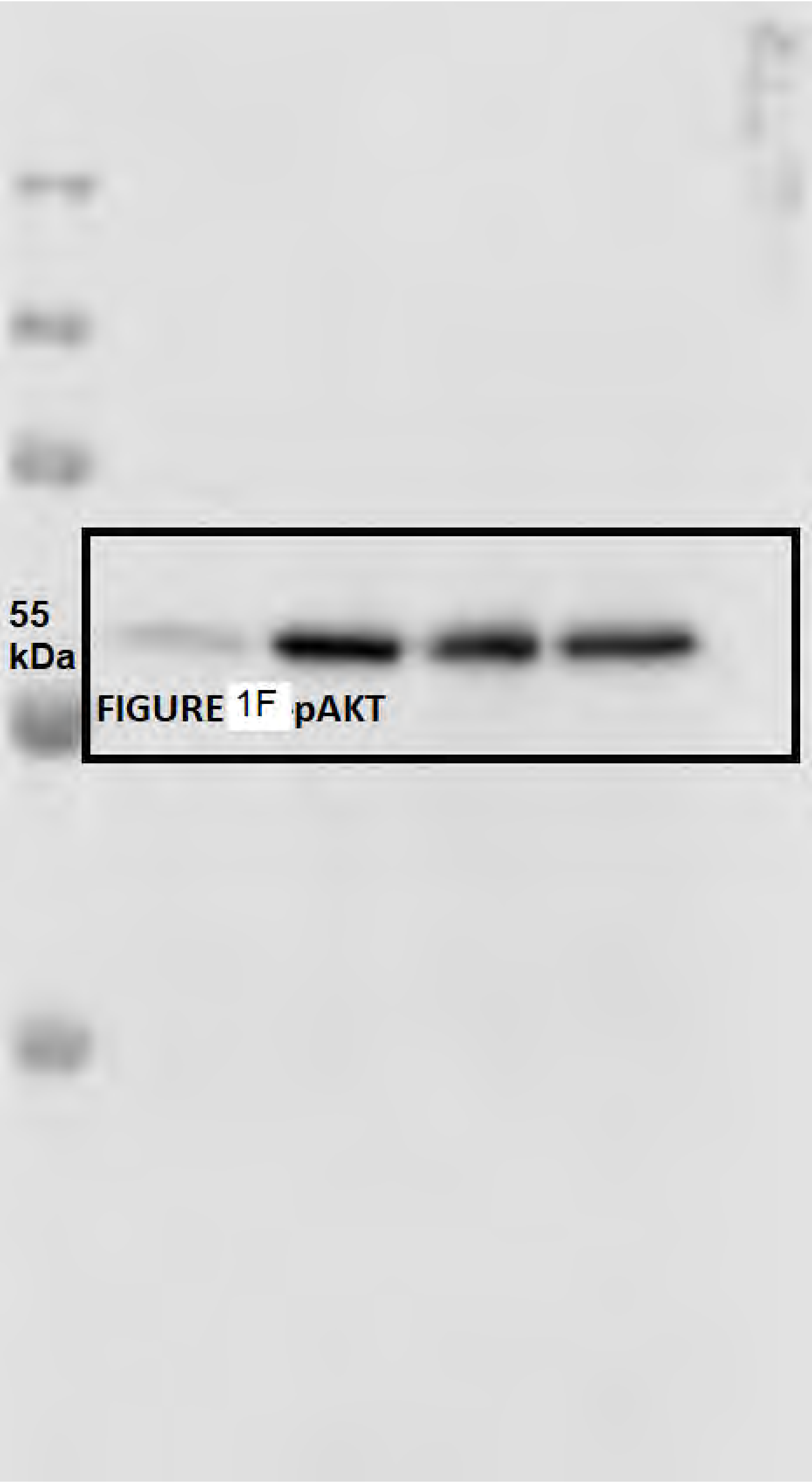
55
kDa
FIGURE 1F pAKT

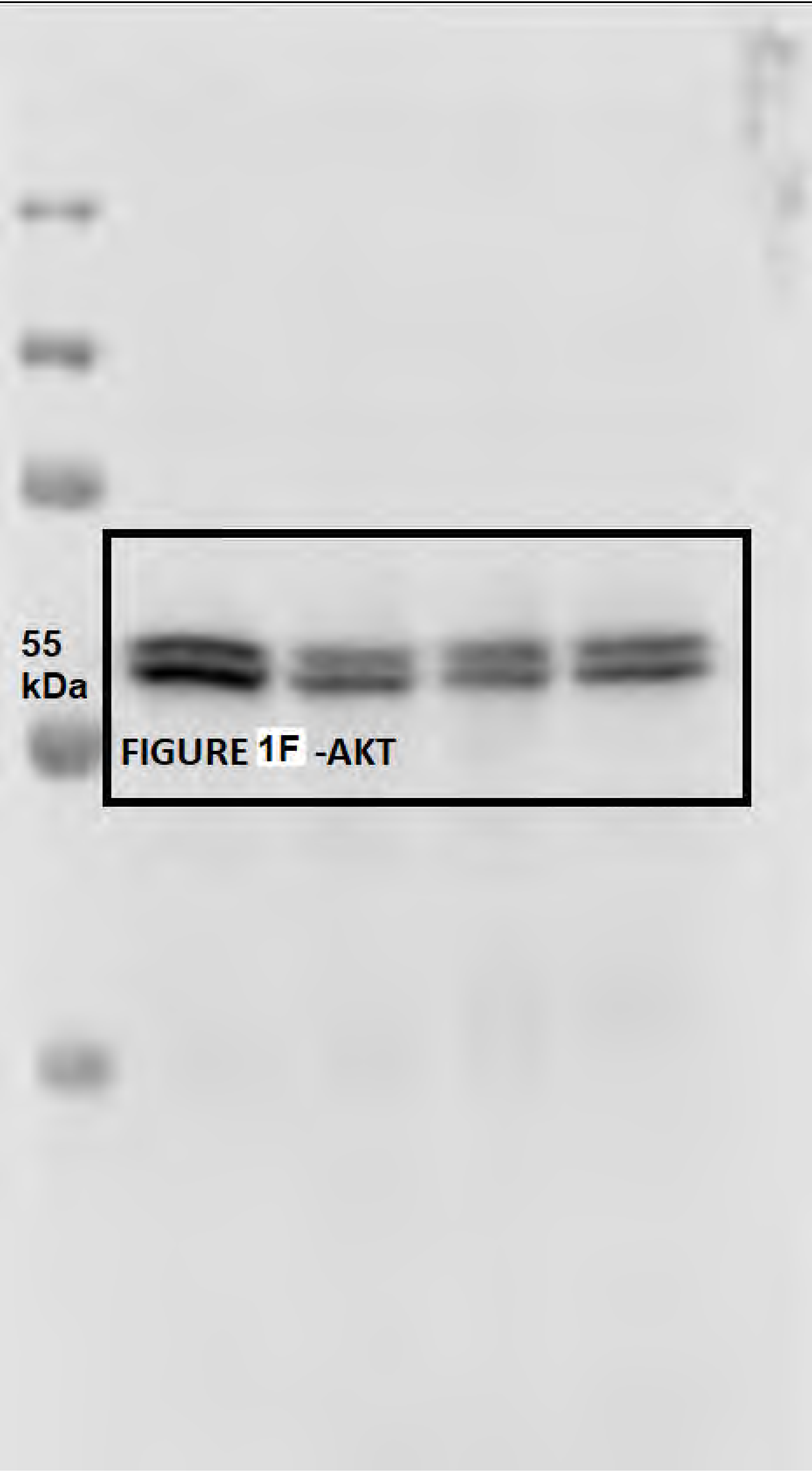
55 kDa
FIGURE 1F -AKT

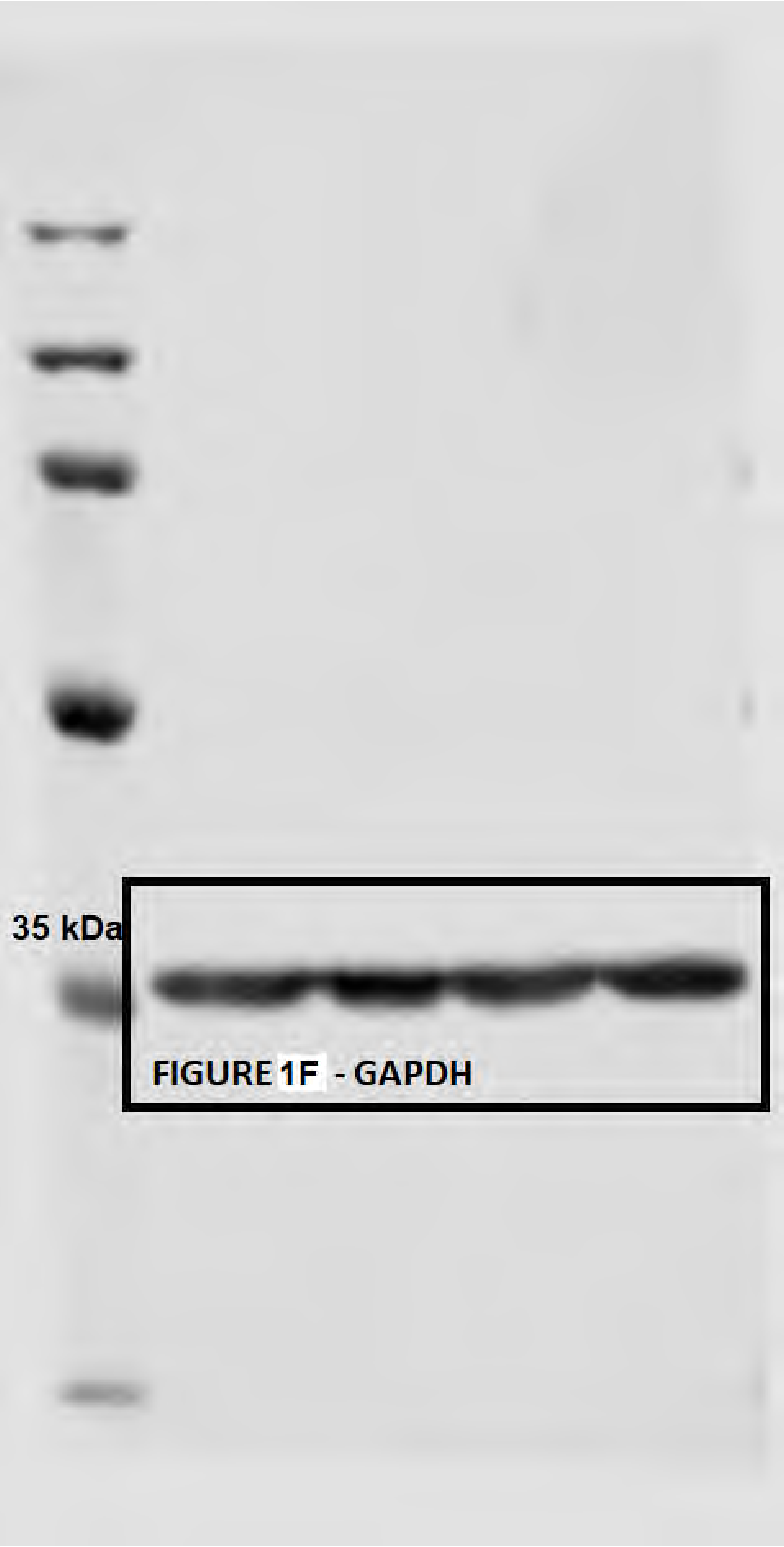
35 kDa
FIGURE 1F - GAPDH

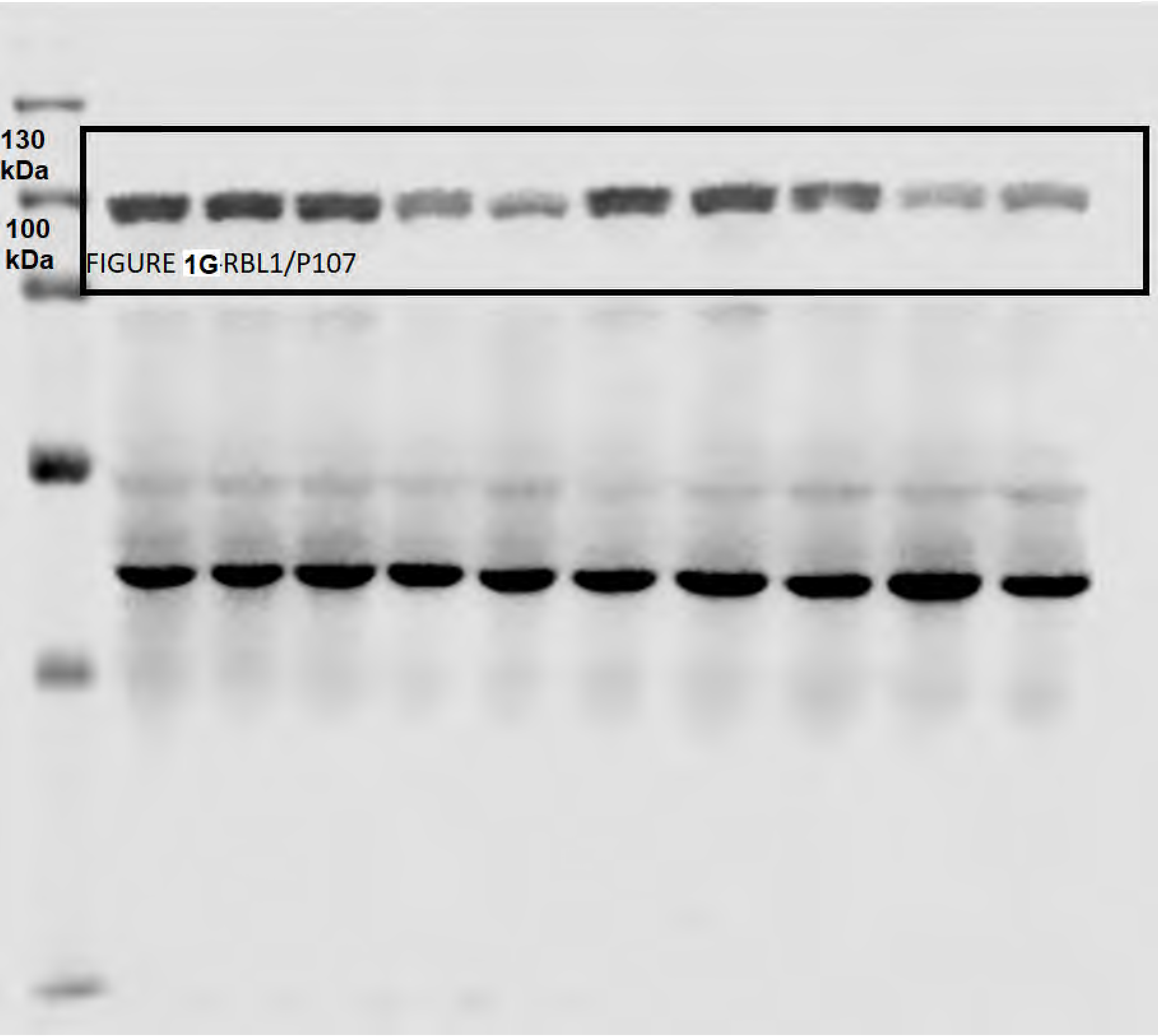
130 kDa
100 kDa
FIGURE 1G RBL1/P107

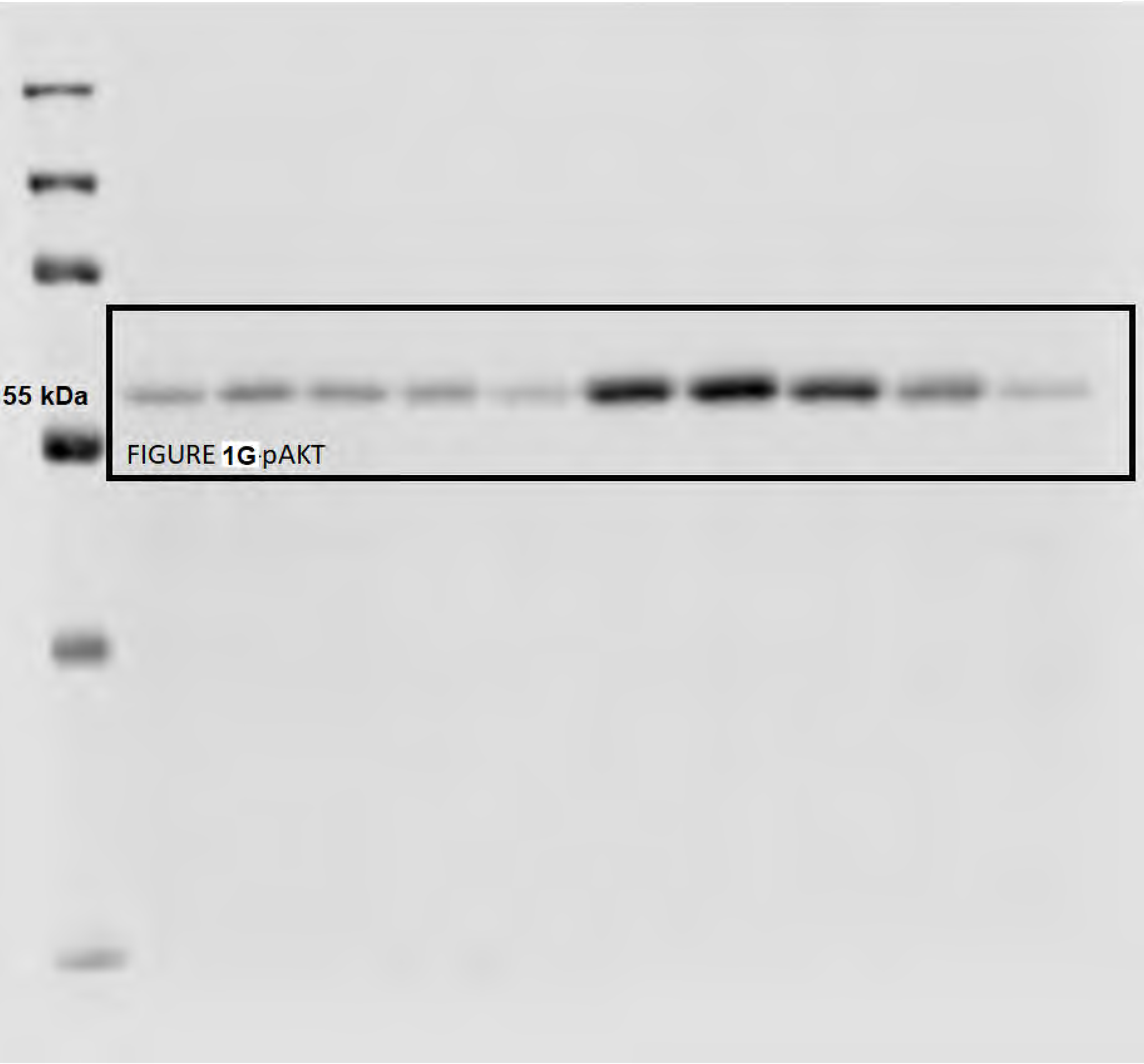
55 kDa
FIGURE 1G pAKT

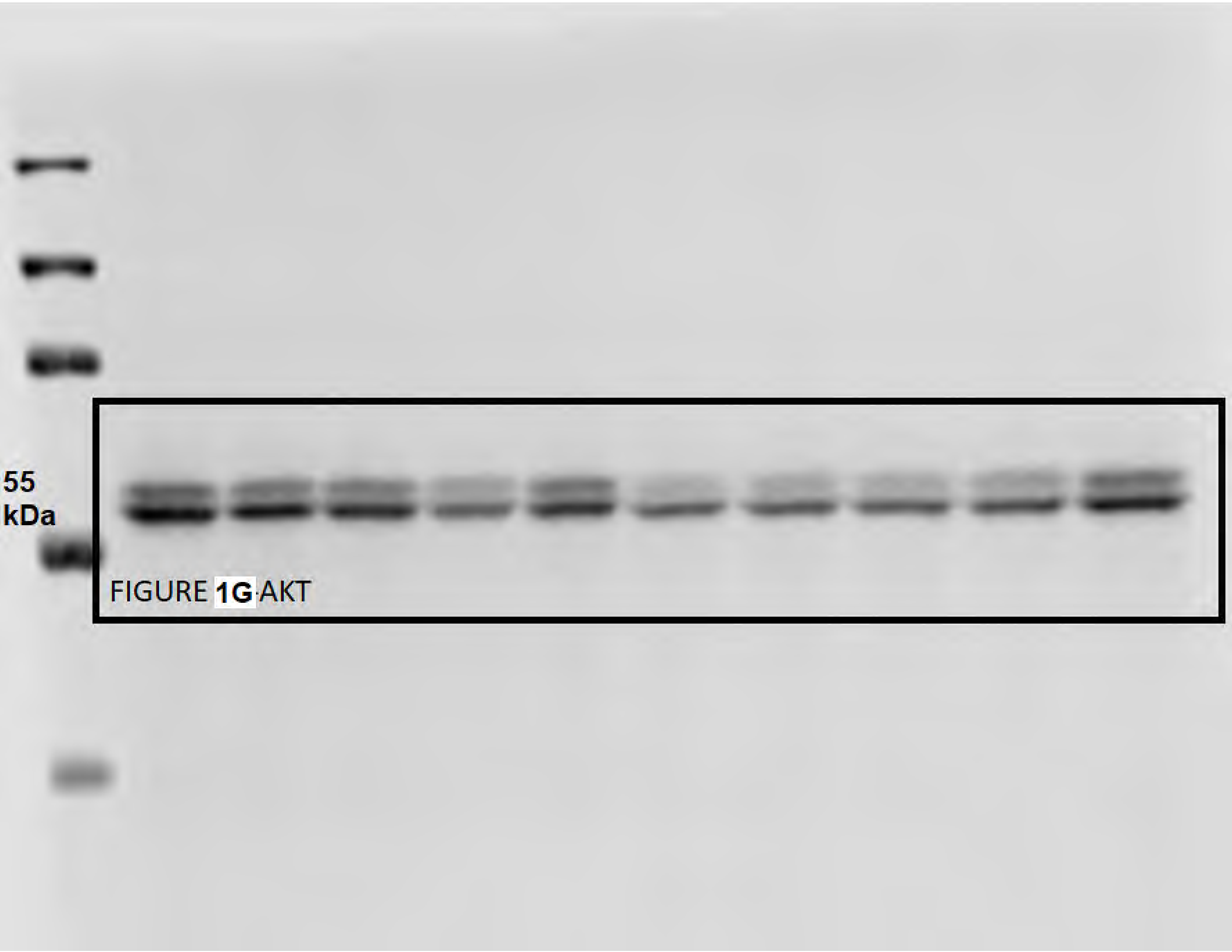
55
kDa
FIGURE 1G AKT

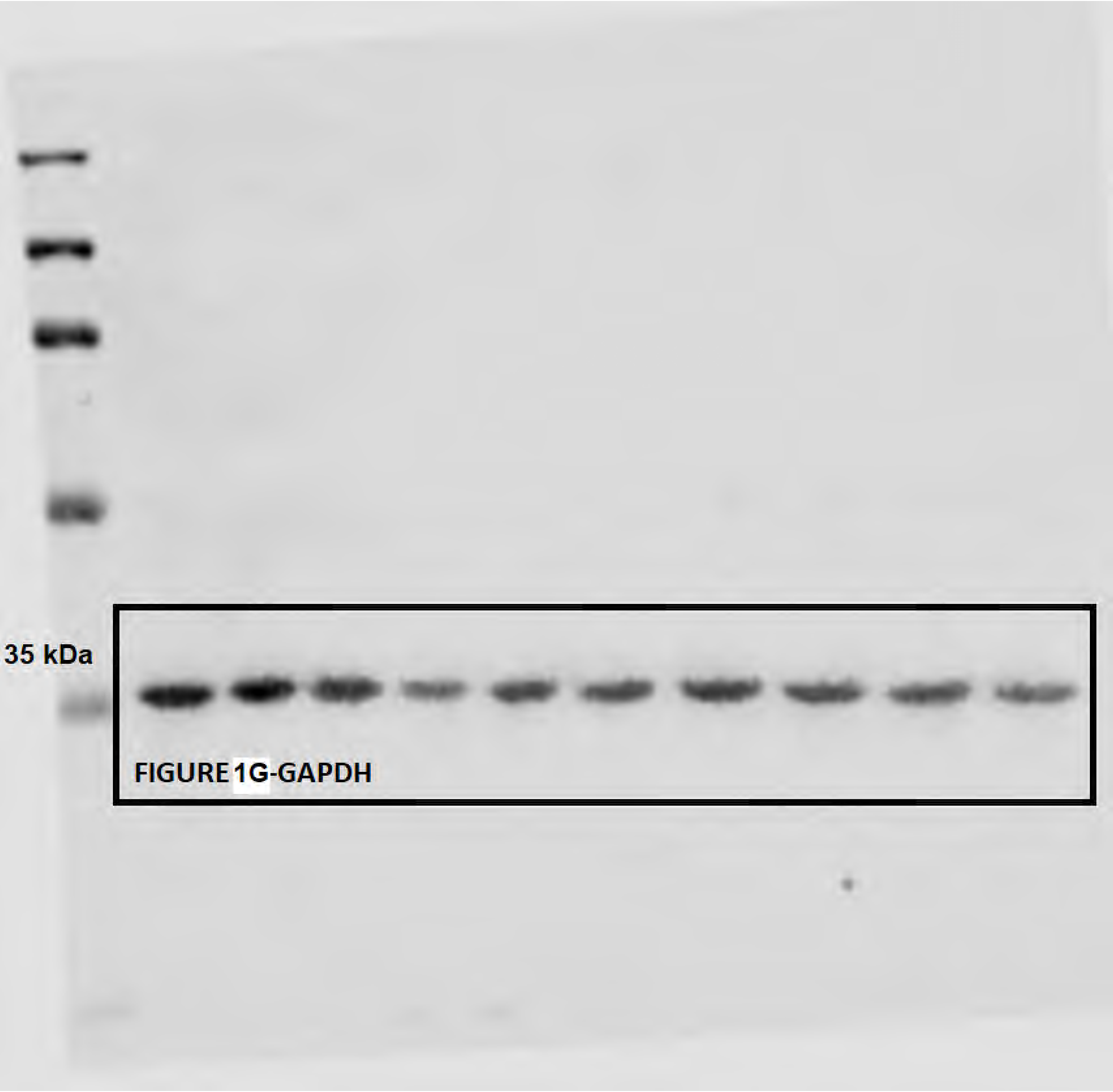
35 kDa
FIGURE 1G-GAPDH

FIGURE 2A-LEFT PANEL-RBL1/p107

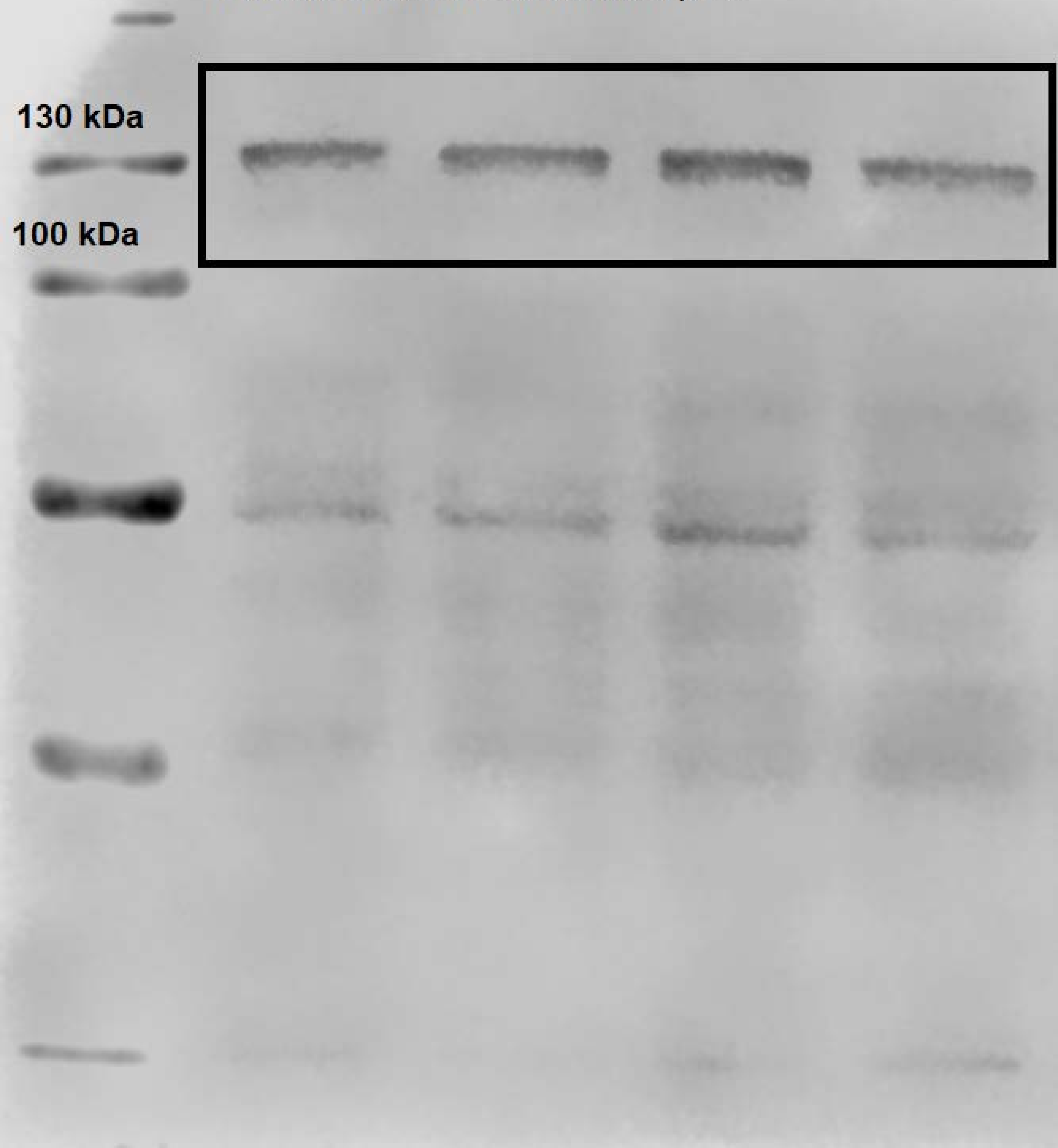

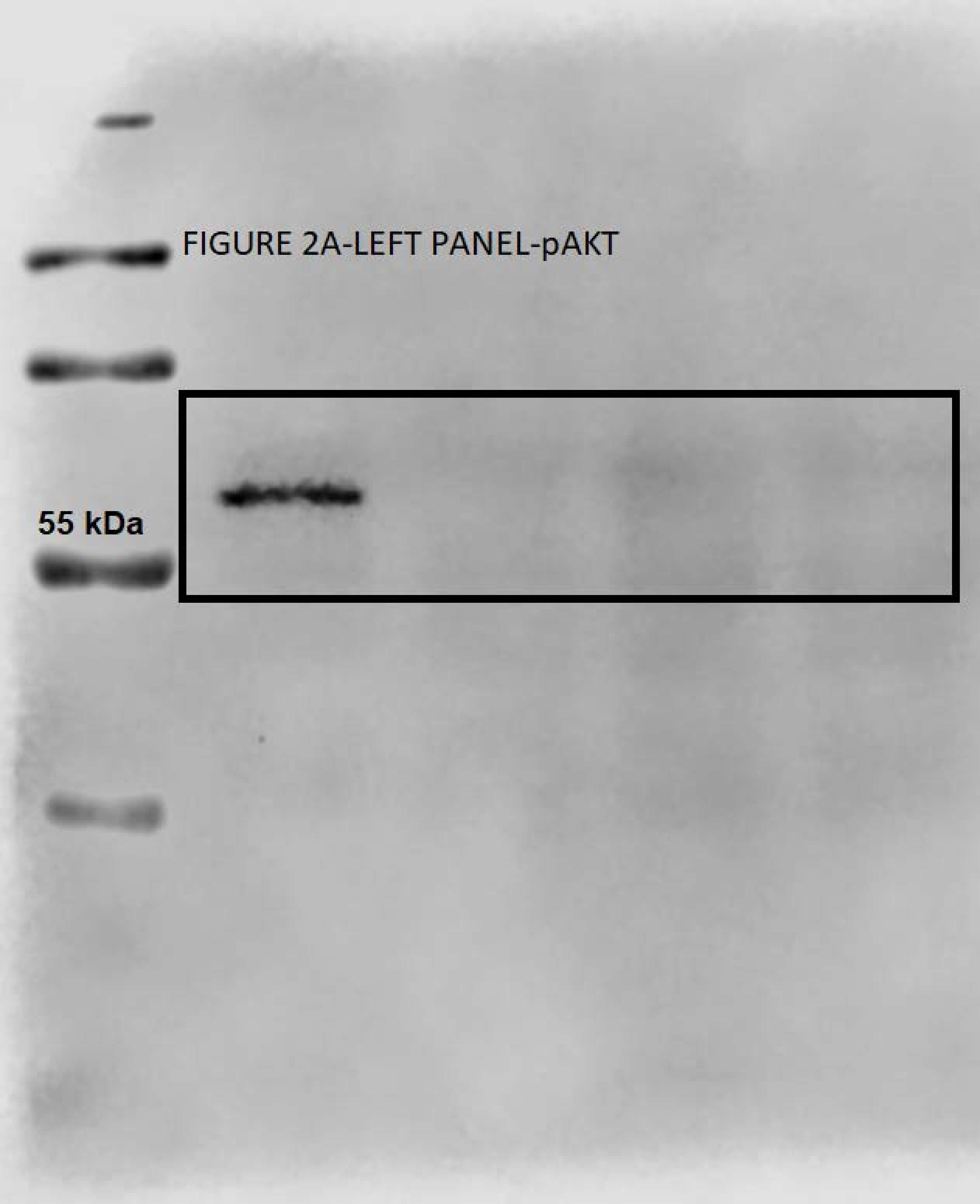
FIGURE 2A-LEFT PANEL-pAKT
55 kDa

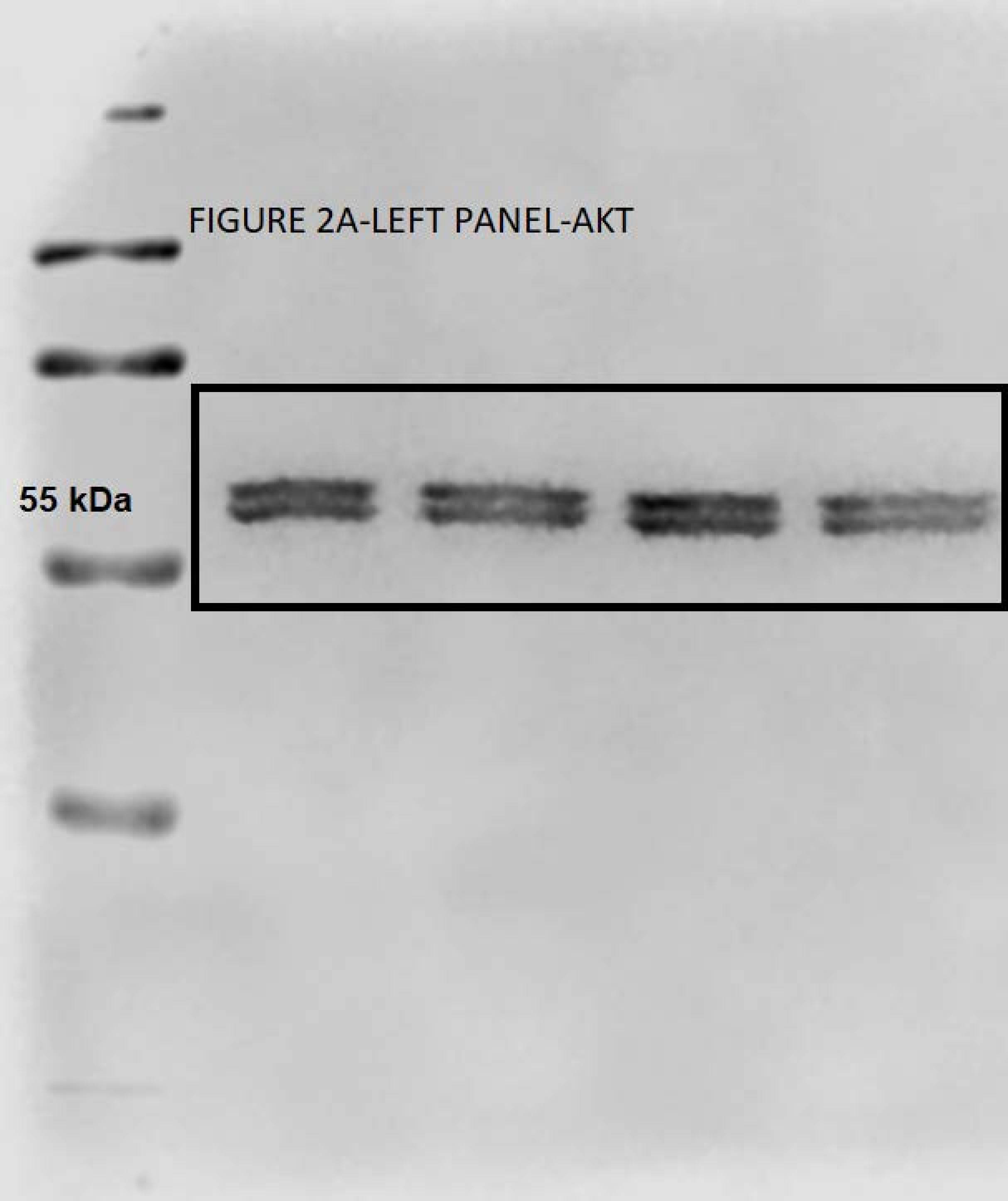
FIGURE 2A-LEFT PANEL-AKT
55 kDa

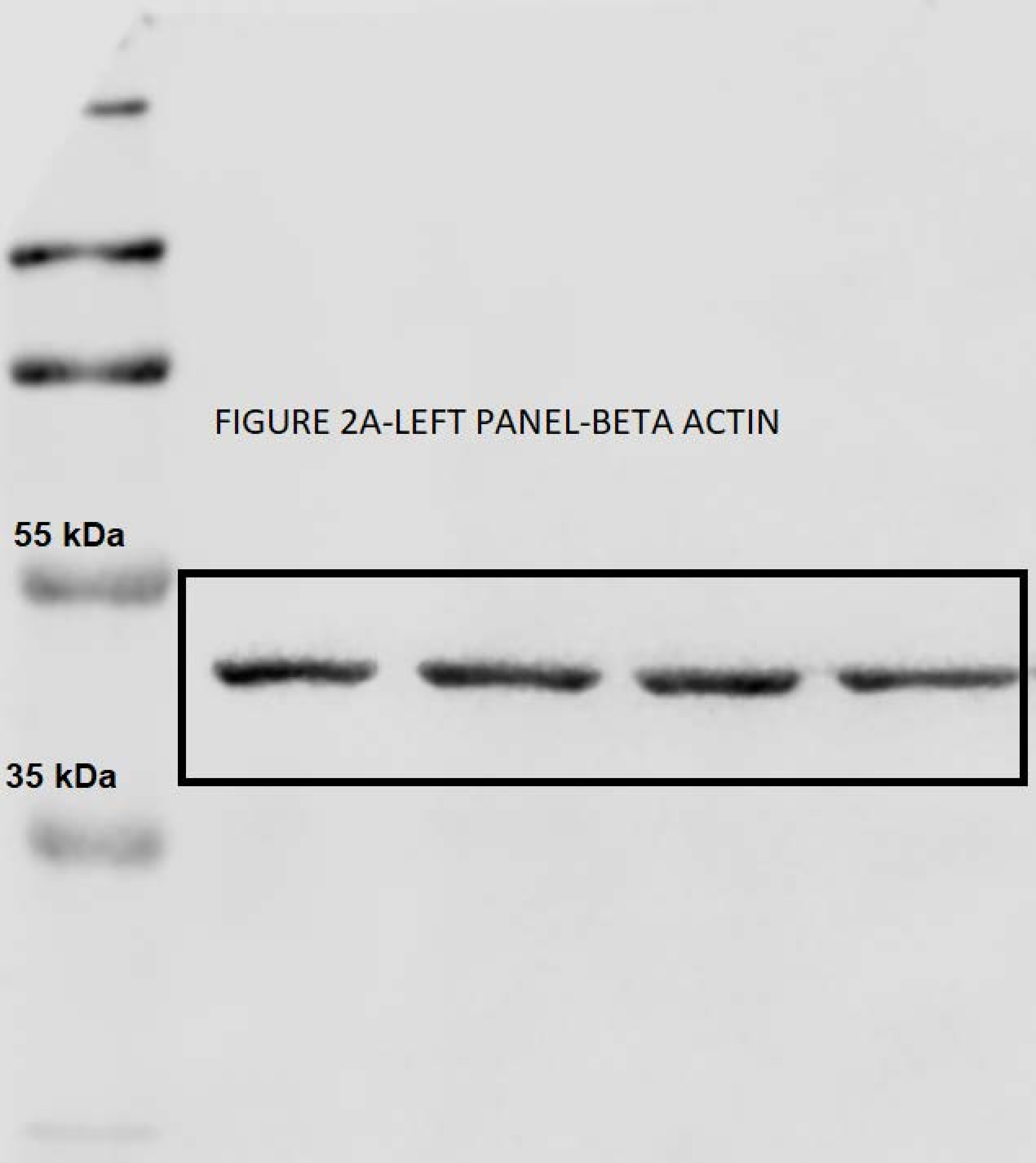

FIGURE 2A-LEFT PANEL-BETA ACTIN

FIGURE 2A-RIGHT PANEL-RBL1/p107

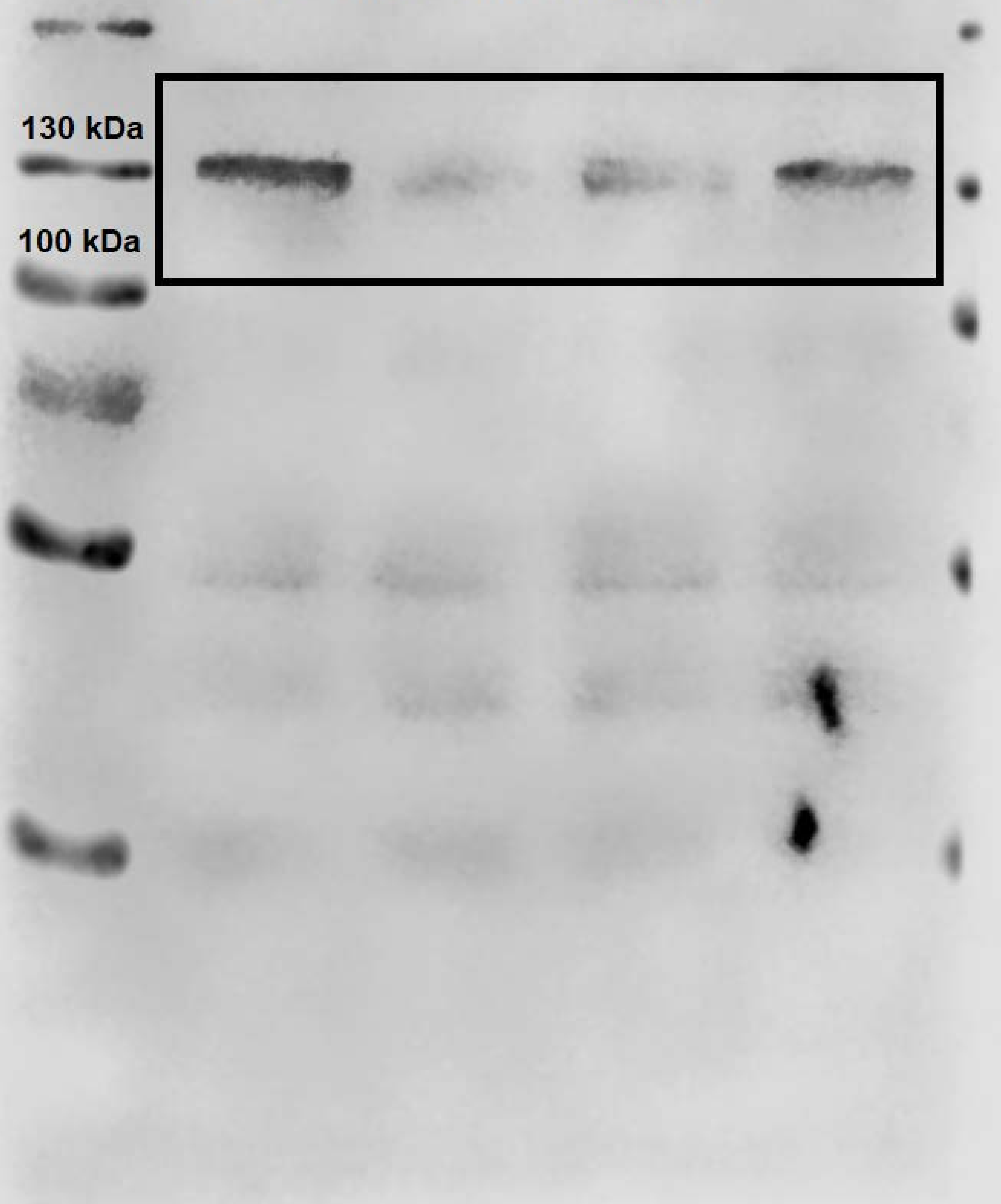

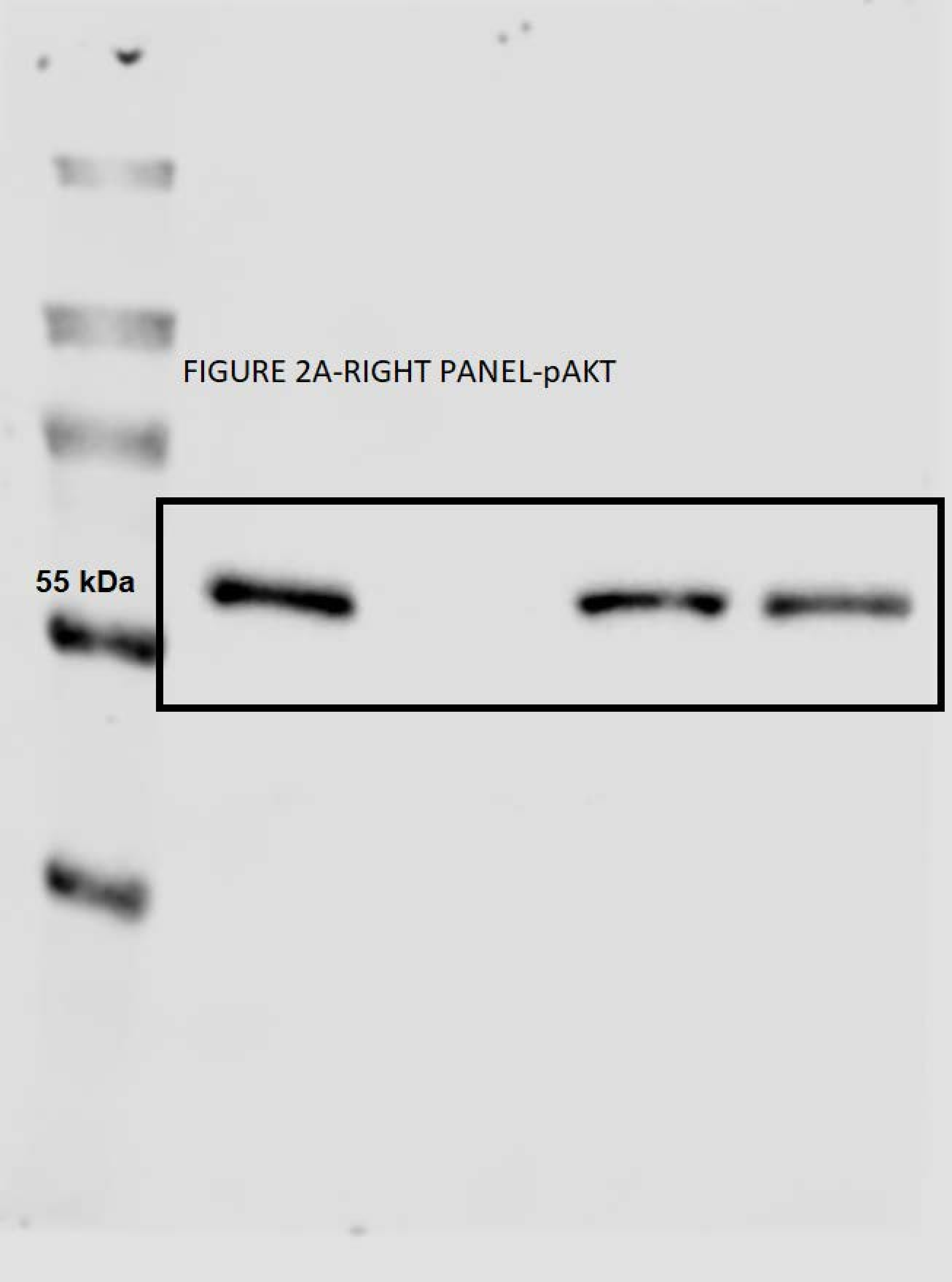
FIGURE 2A-RIGHT PANEL-pAKT
55 kDa

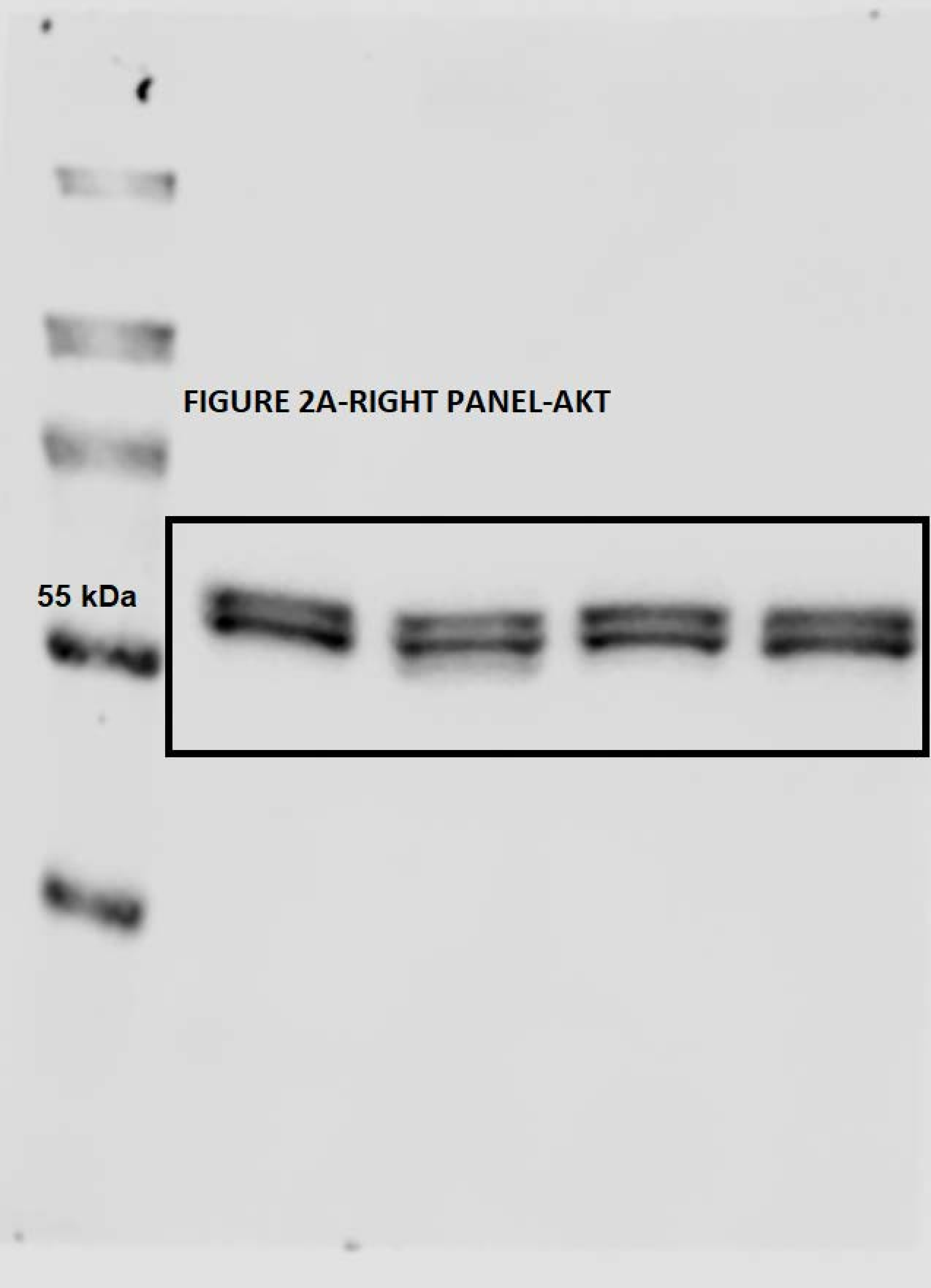
FIGURE 2A-RIGHT PANEL-AKT
55 kDa

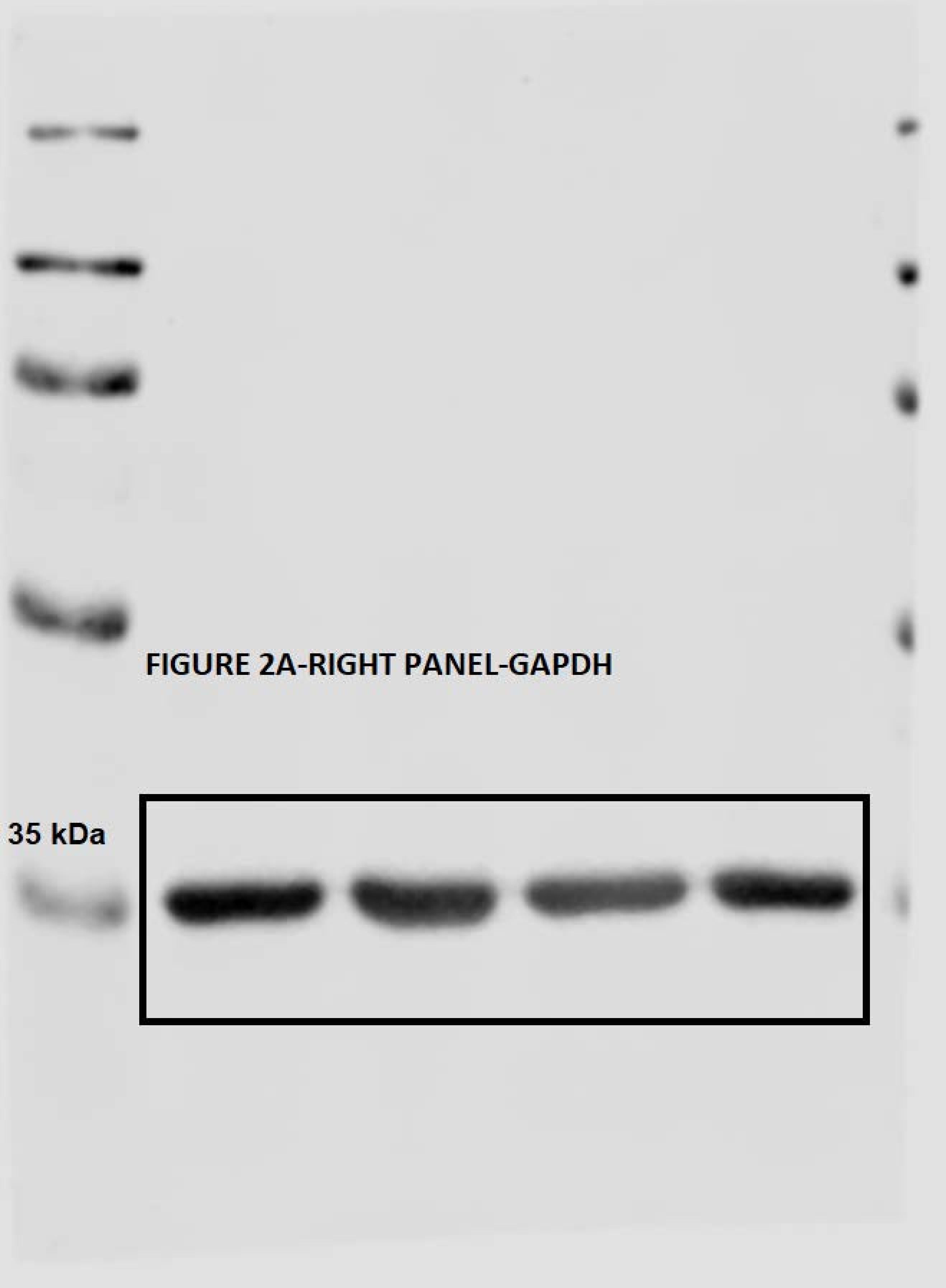
FIGURE 2A-RIGHT PANEL-GAPDH
35 kDa

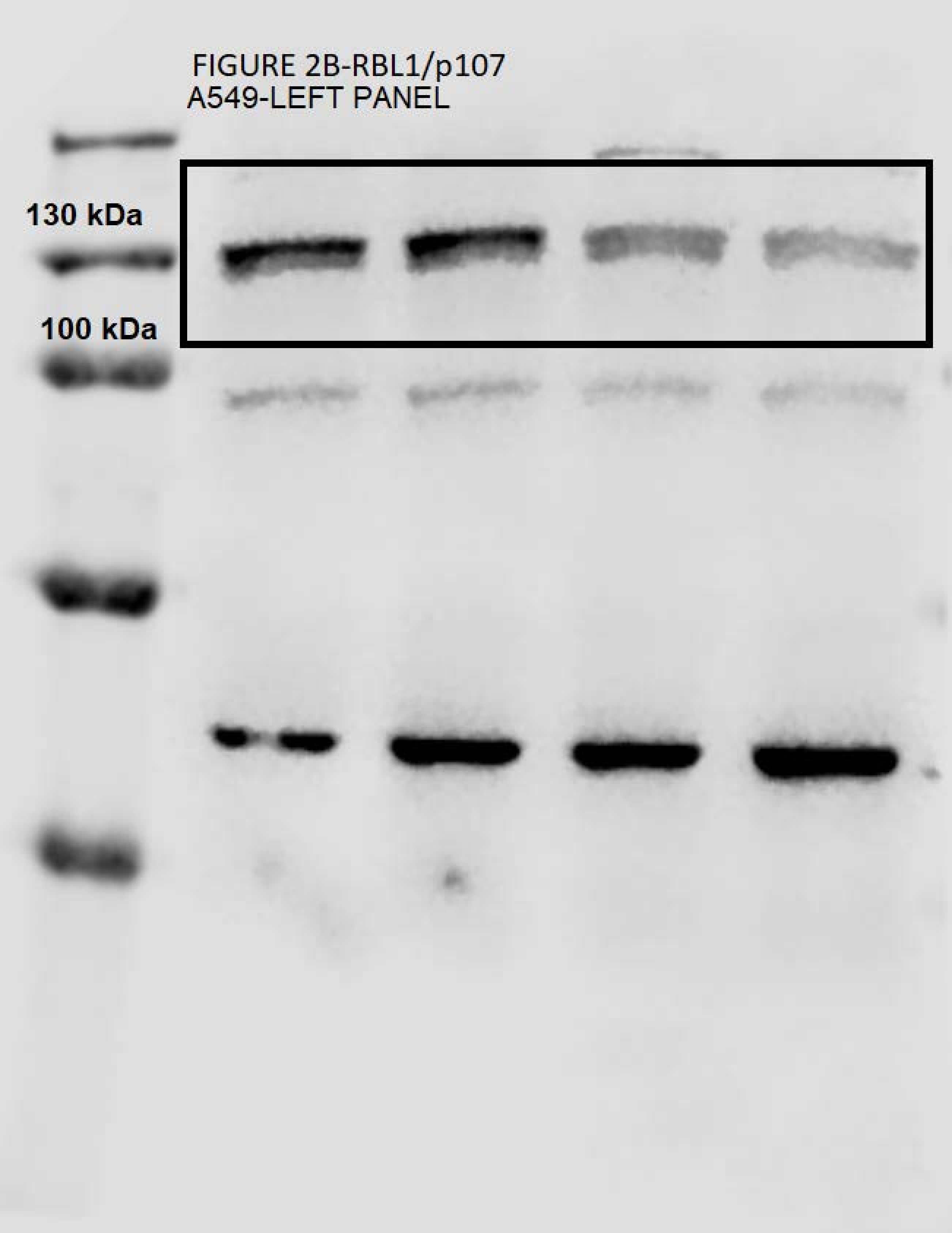
FIGURE 2B-RBL1/p107
A549-LEFT PANEL
130 kDa
100 kDa

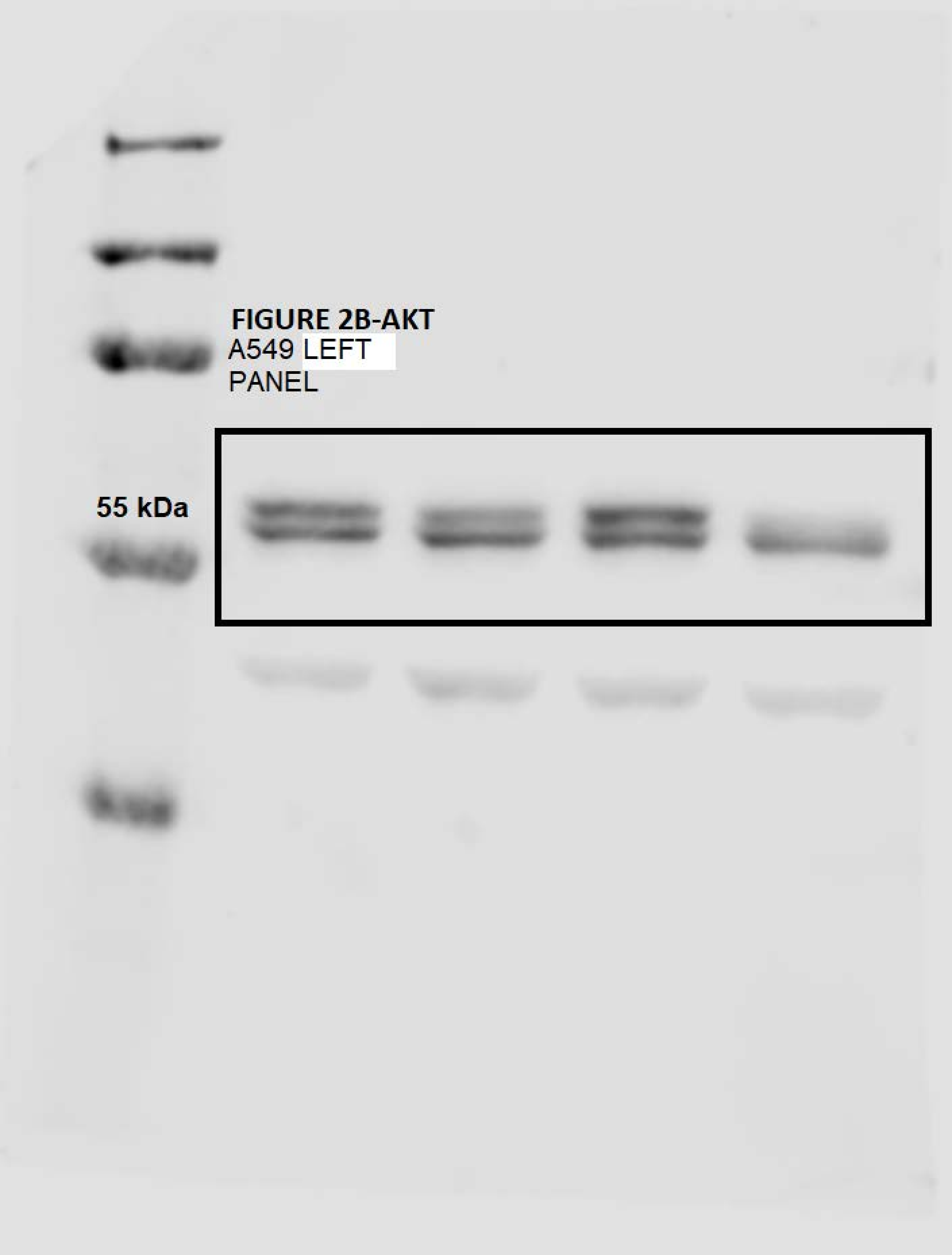
FIGURE 2B-AKT
A549 LEFT
PANEL
55 kDa

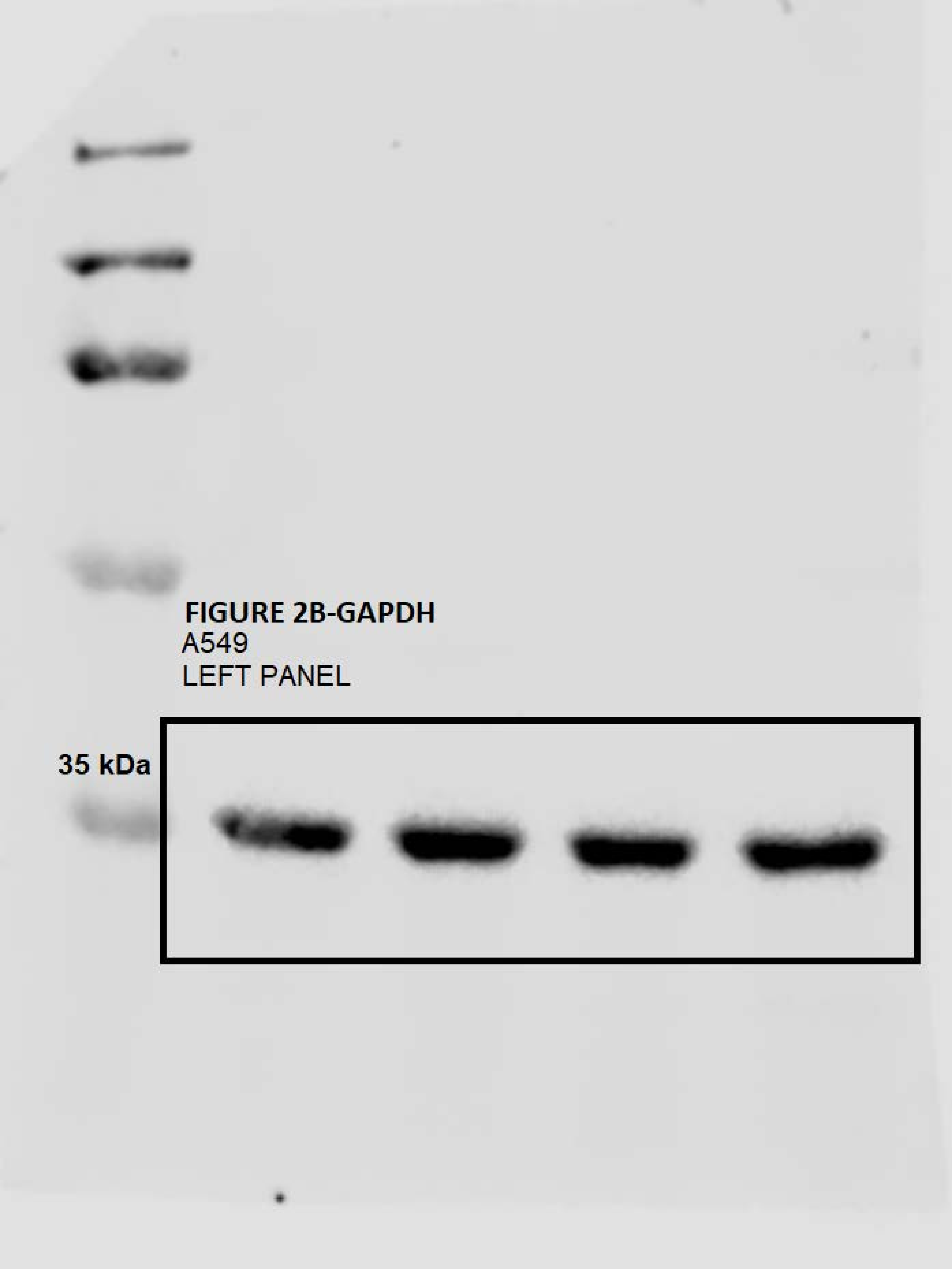
FIGURE 2B-GAPDH
A549
LEFT PANEL
35 kDa

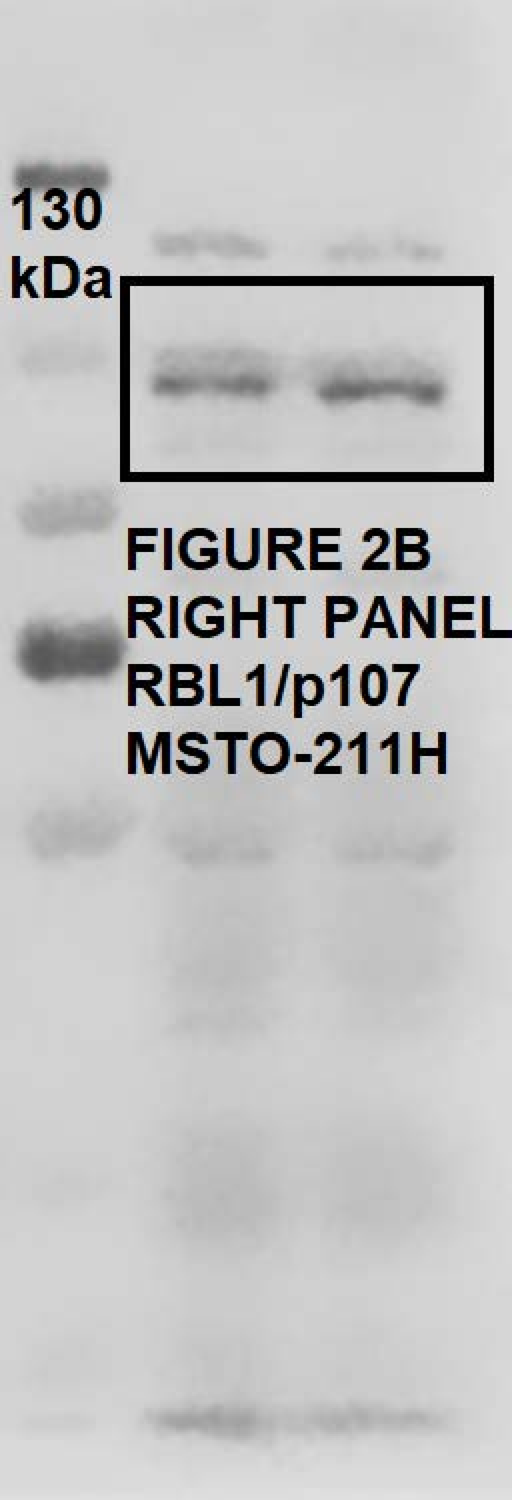
130
kDa
FIGURE 2B
RIGHT PANEL
RBL1/p107
MSTO-211H

FIGURE 2B
RIGHT
PANEL
AKT
MSTO-211H

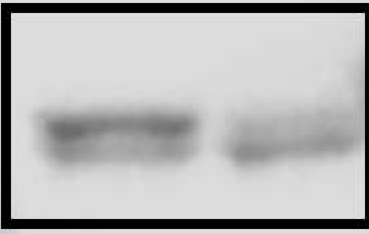

55
kDa

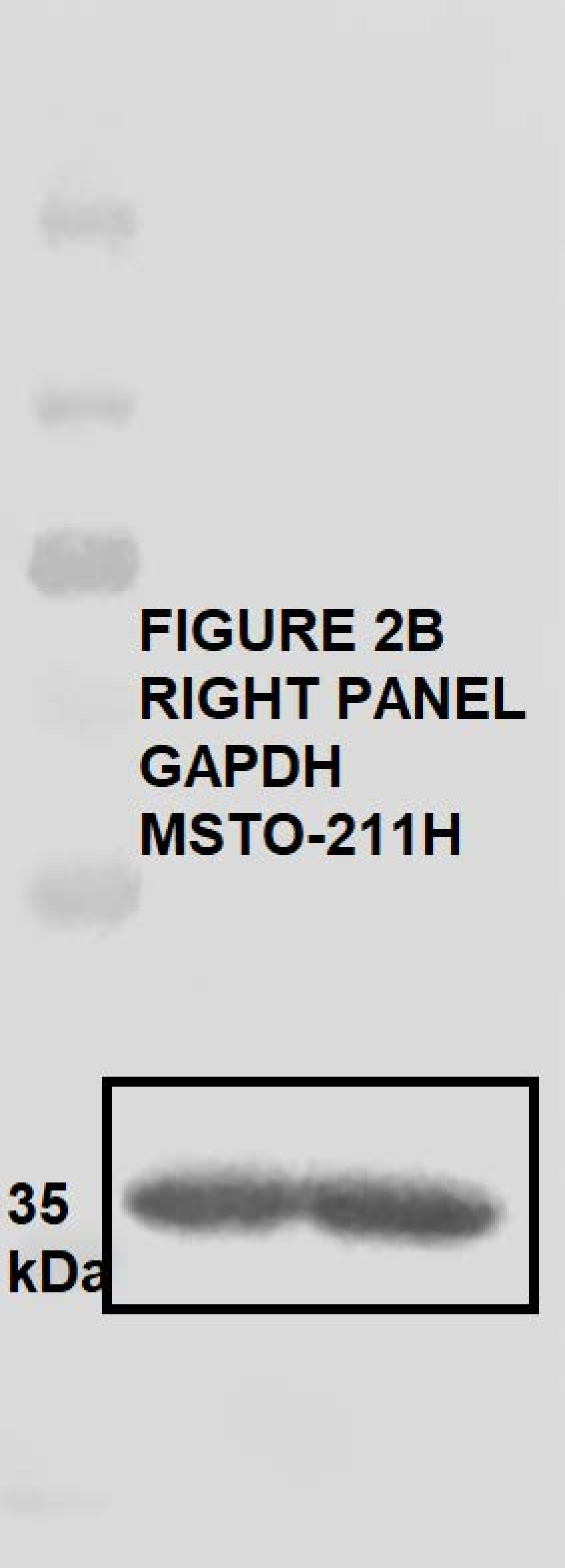
FIGURE 2B
RIGHT PANEL
GAPDH
MSTO-211H
35
kDa

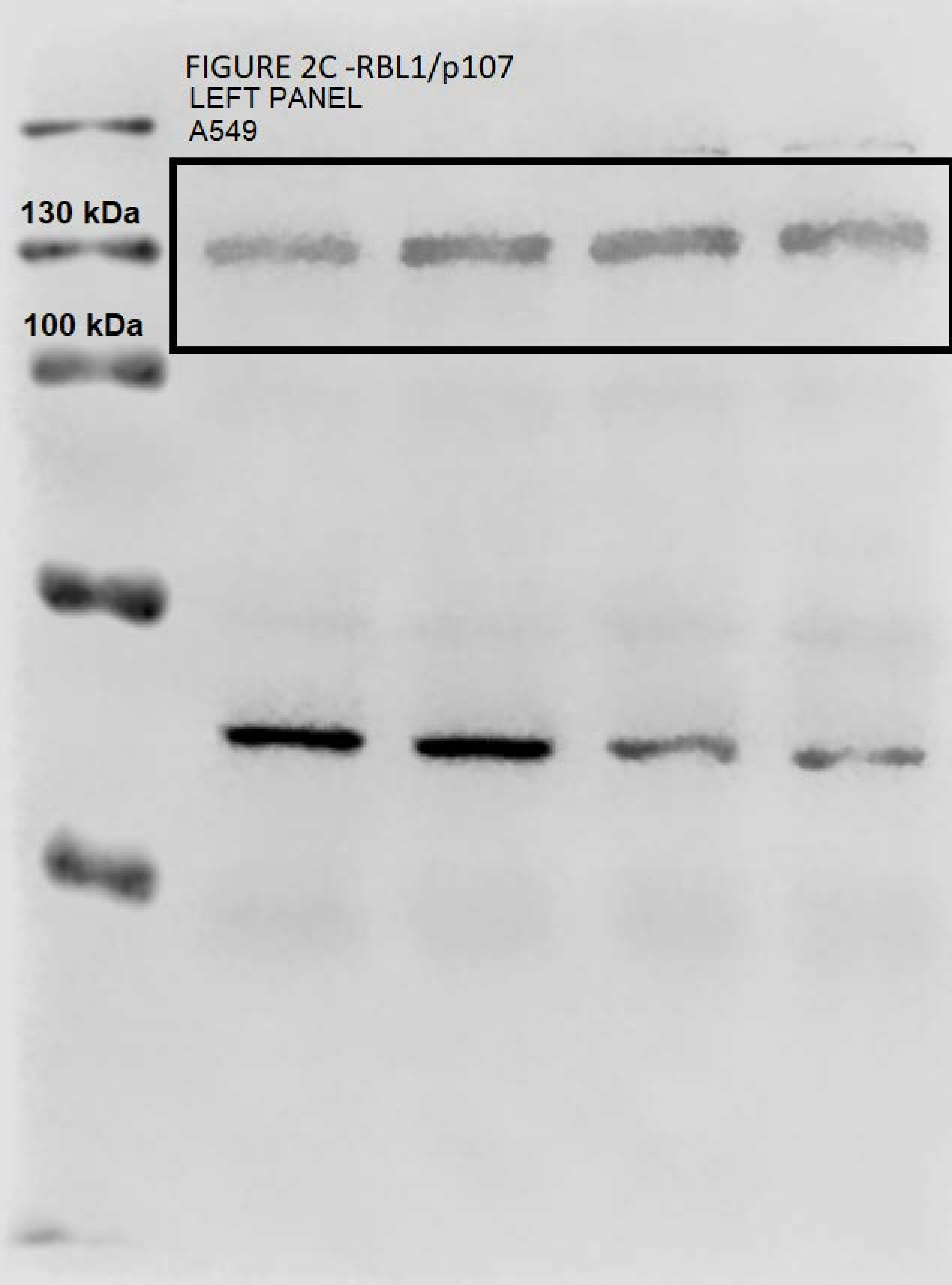
FIGURE 2C -RBL1/p107
LEFT PANEL
A549
130 kDa
100 kDa

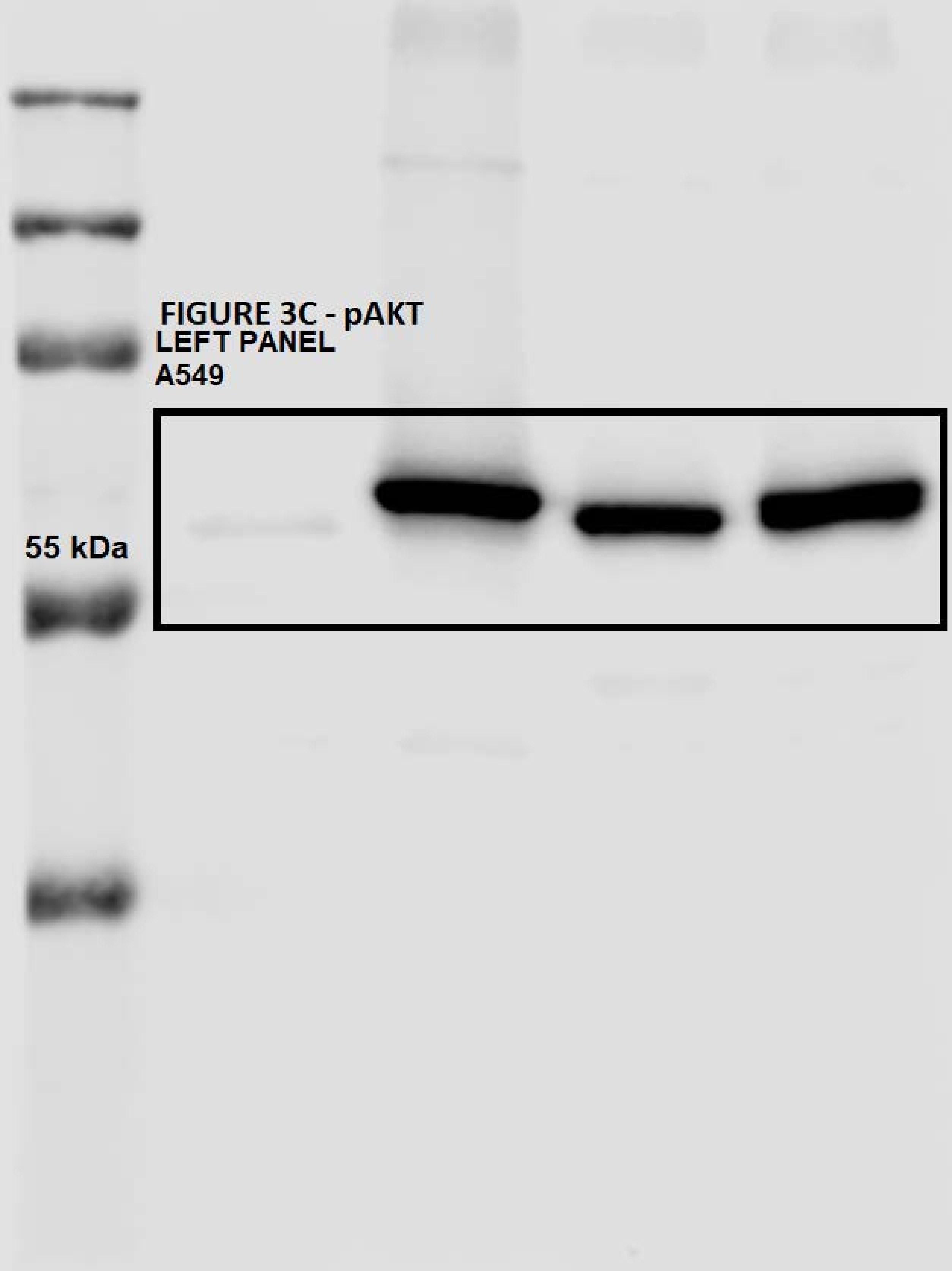
FIGURE 3C - pAKT
LEFT PANEL
A549
55 kDa

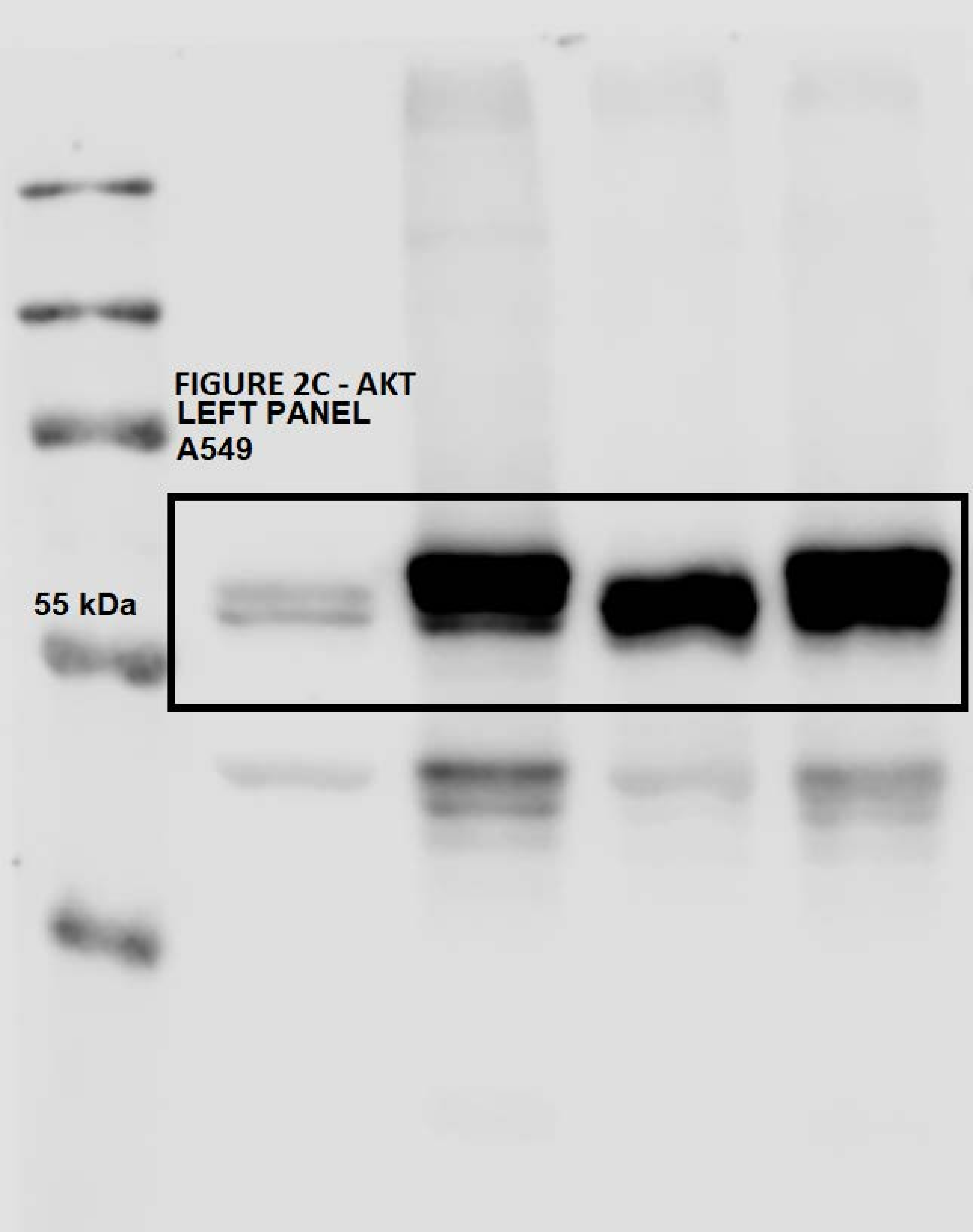
FIGURE 2C - AKT
LEFT PANEL
A549
55 kDa

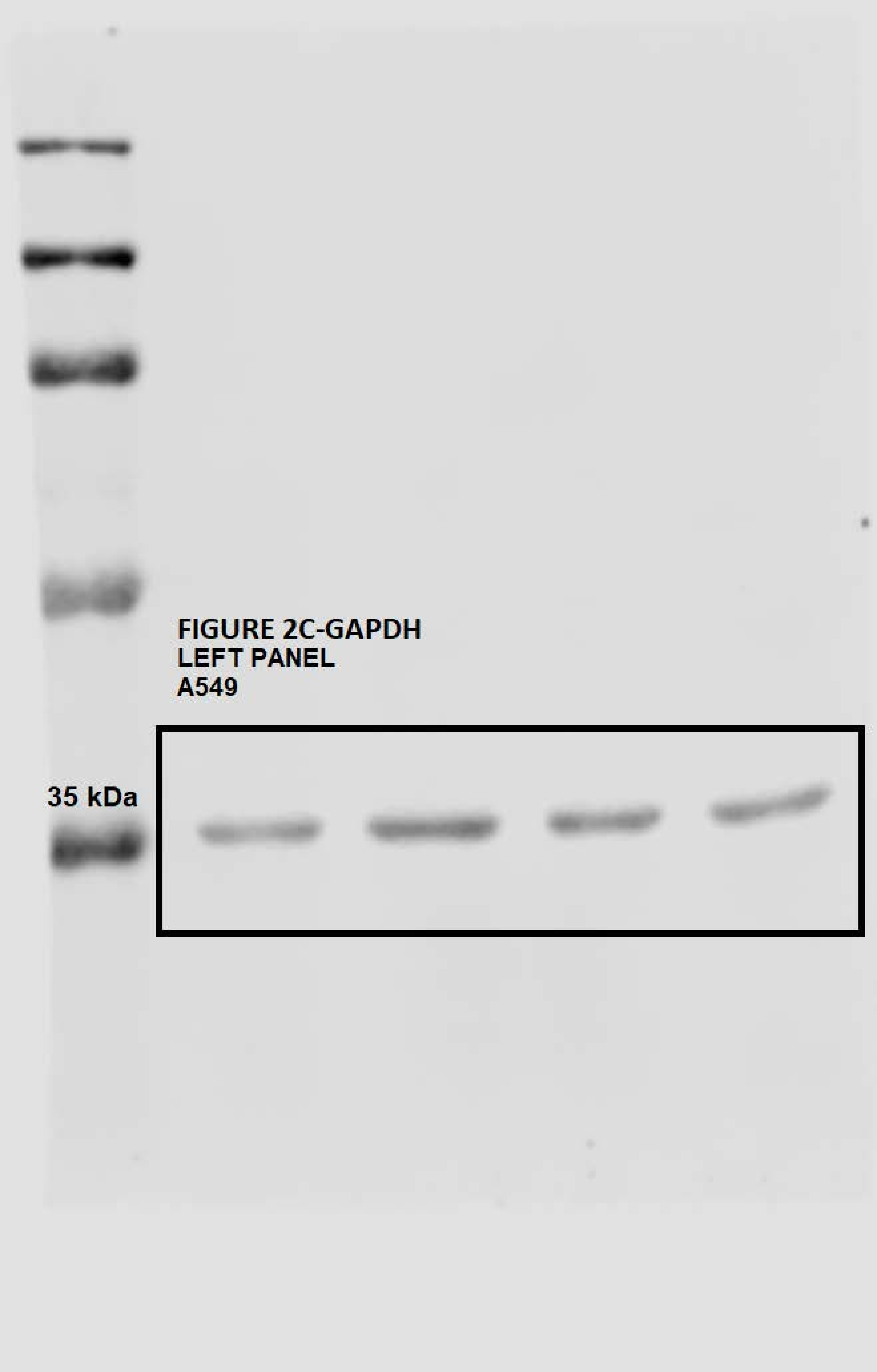
FIGURE 2C-GAPDH
LEFT PANEL
A549
35 kDa

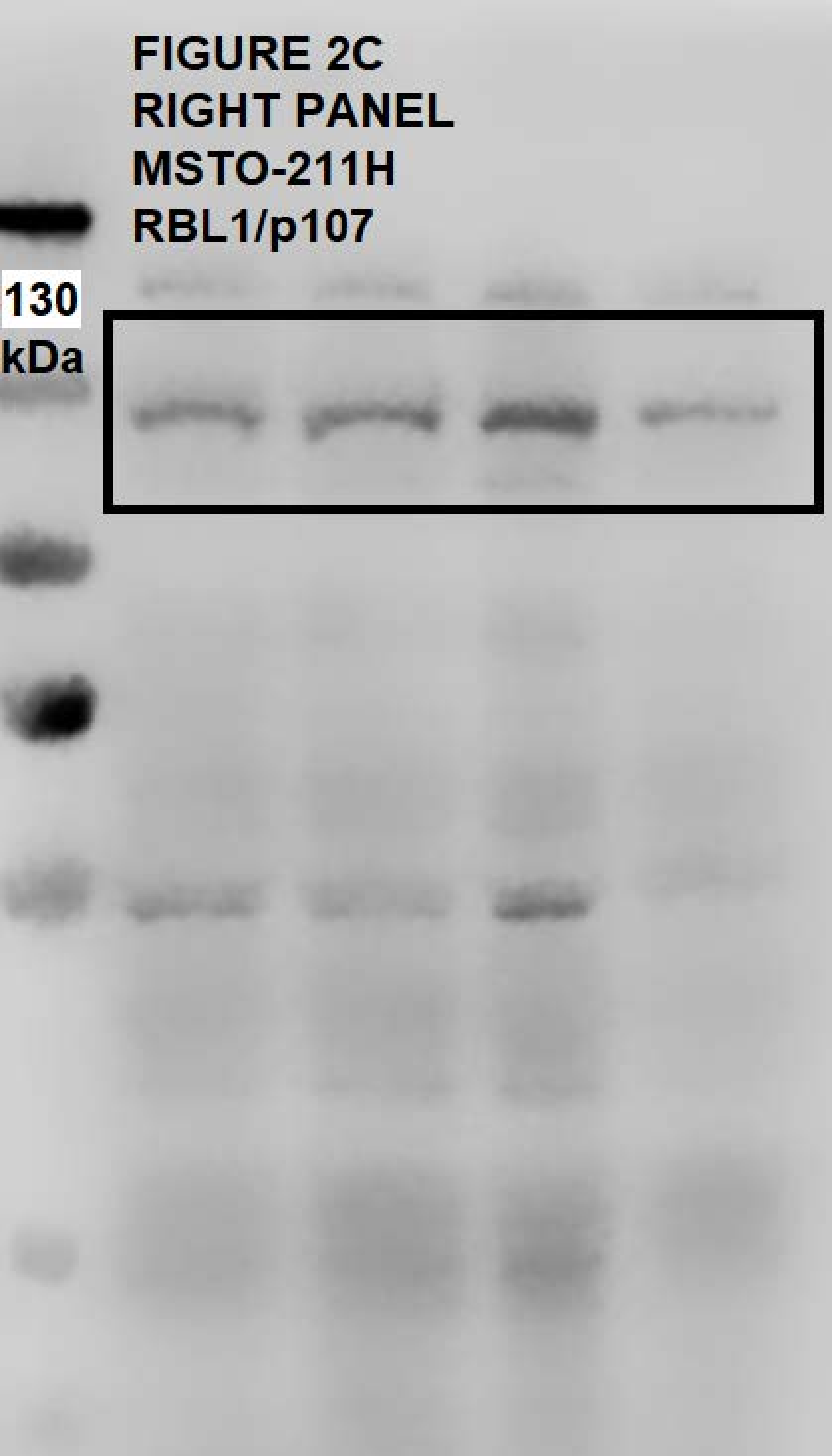
FIGURE 2C
RIGHT PANEL
MSTO-211H
RBL1/p107
130
kDa

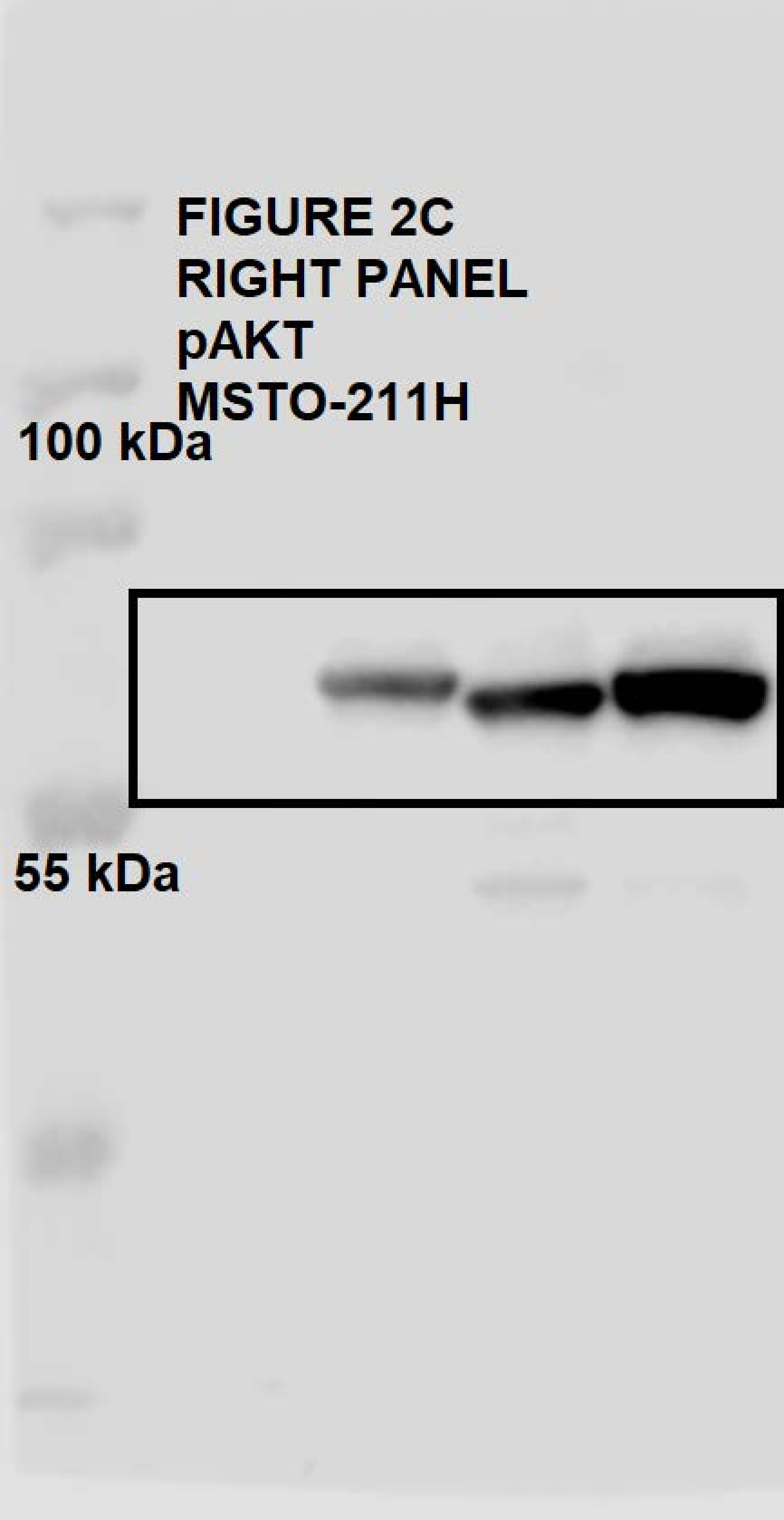
FIGURE 2C
RIGHT PANEL
pAKT
MSTO-211H
100 kDa
55 kDa

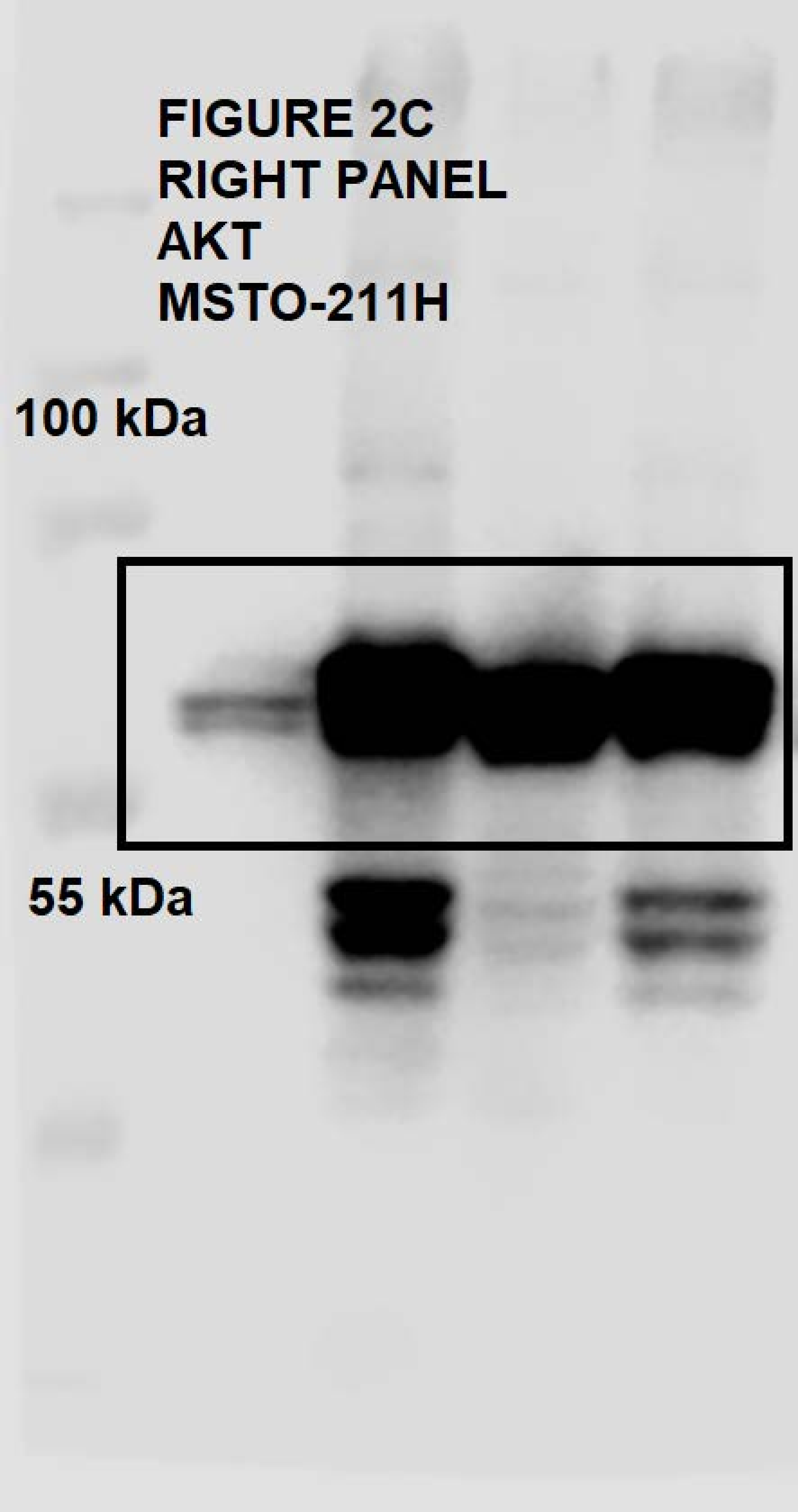
FIGURE 2C
RIGHT PANEL
AKT
MSTO-211H
100 kDa
55 kDa

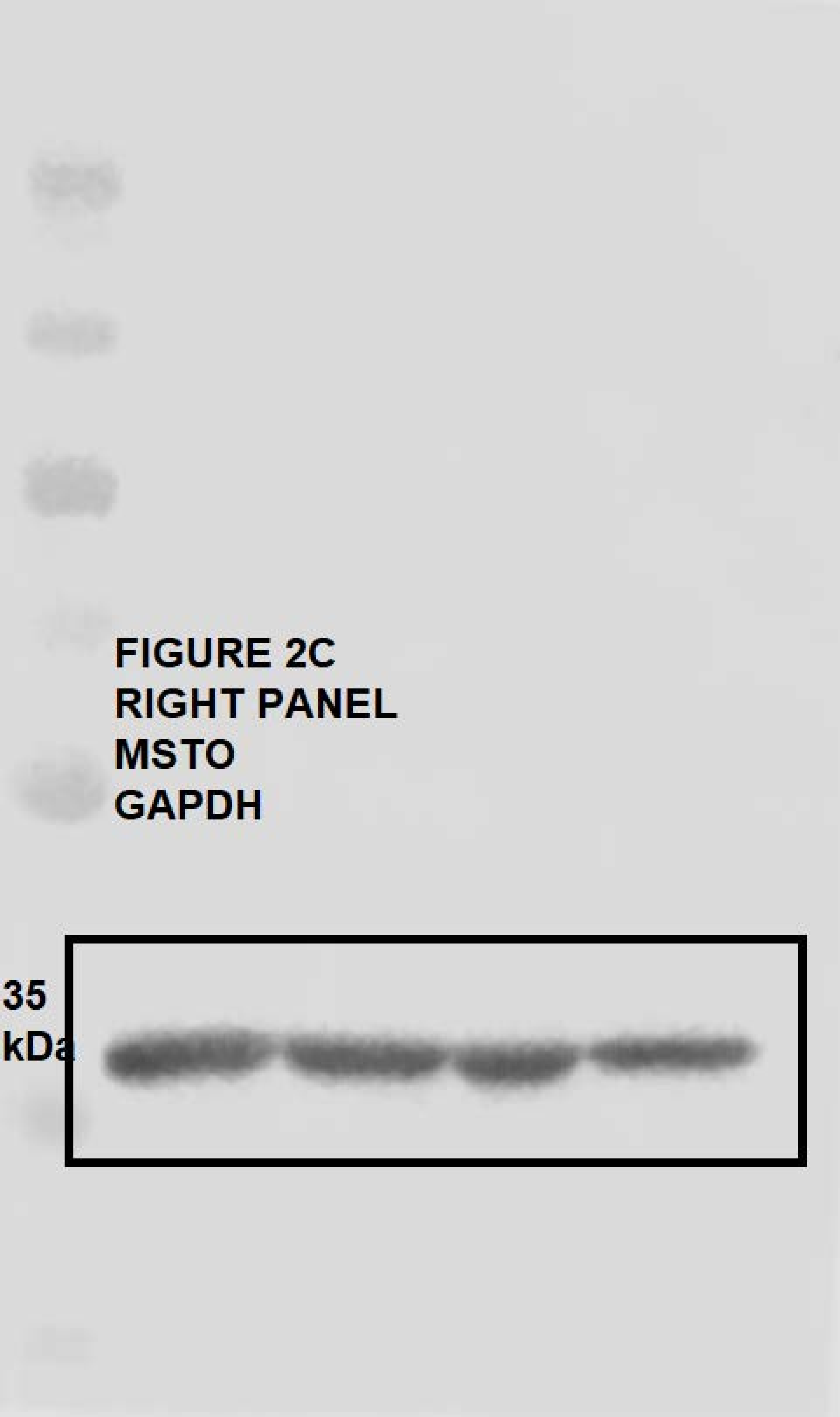
FIGURE 2C
RIGHT PANEL
MSTO
GAPDH
35
kDa

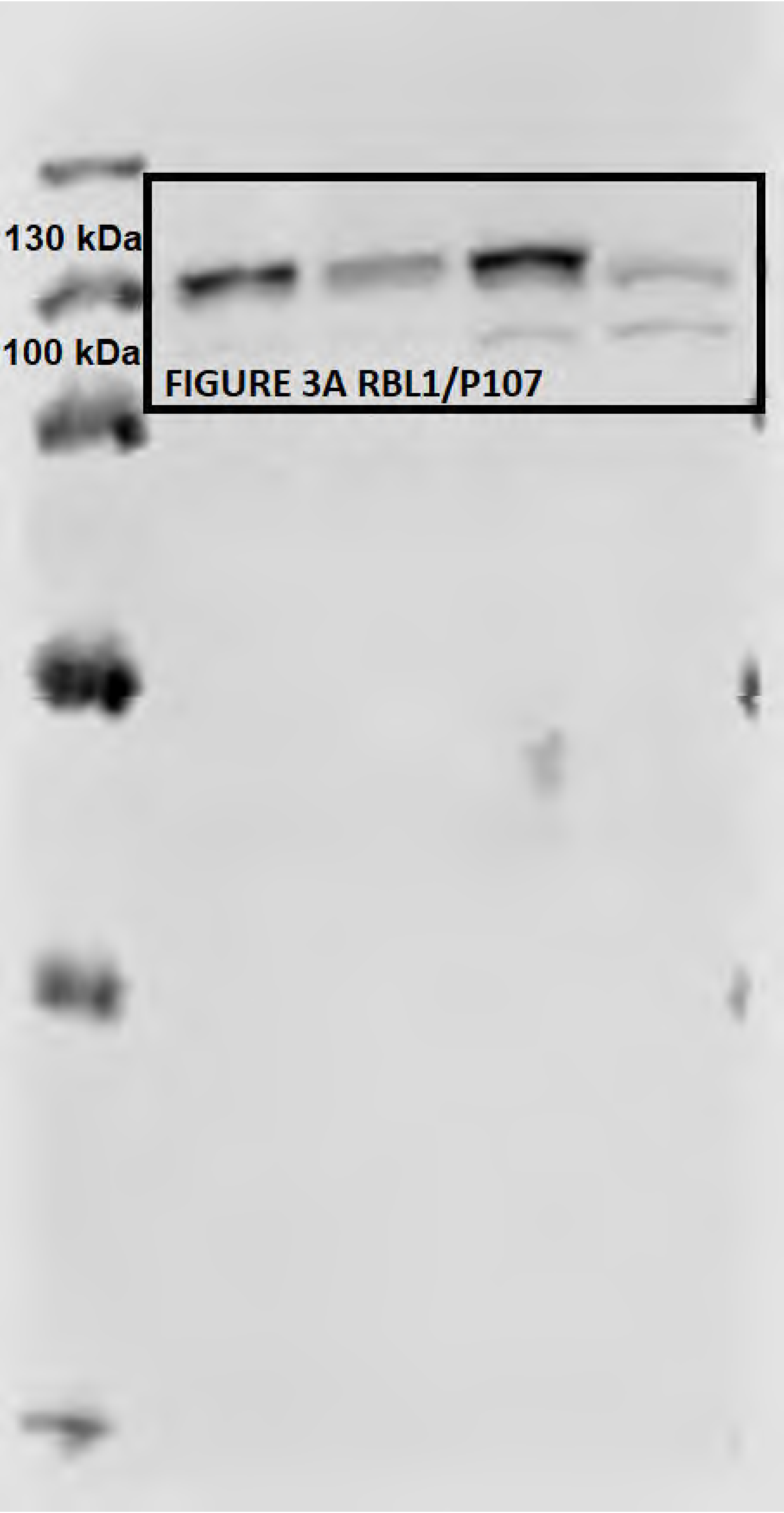
130 kDa
100 kDa
FIGURE 3A RBL1/P107

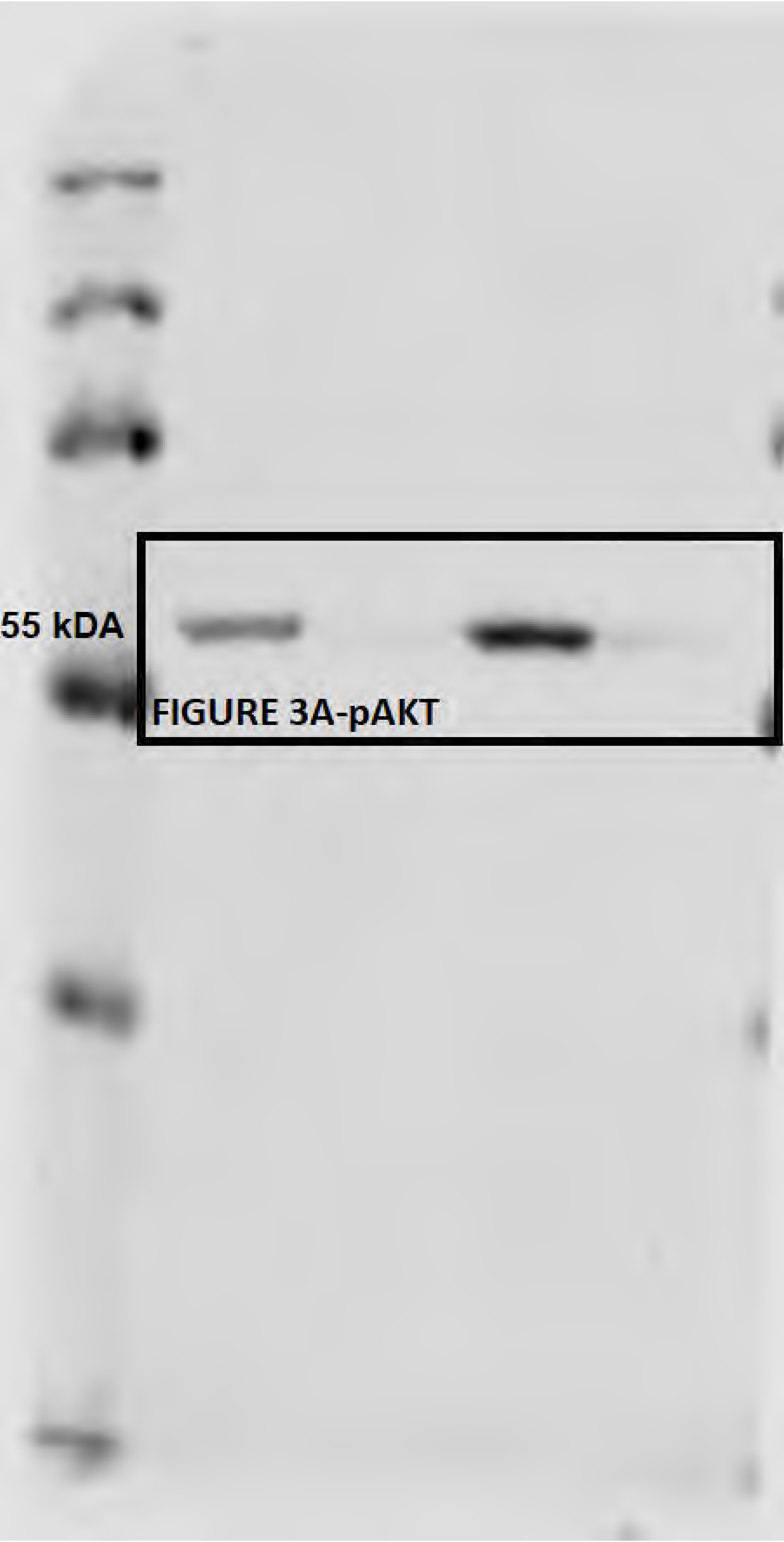
55 kDA
FIGURE 3A-pAKT

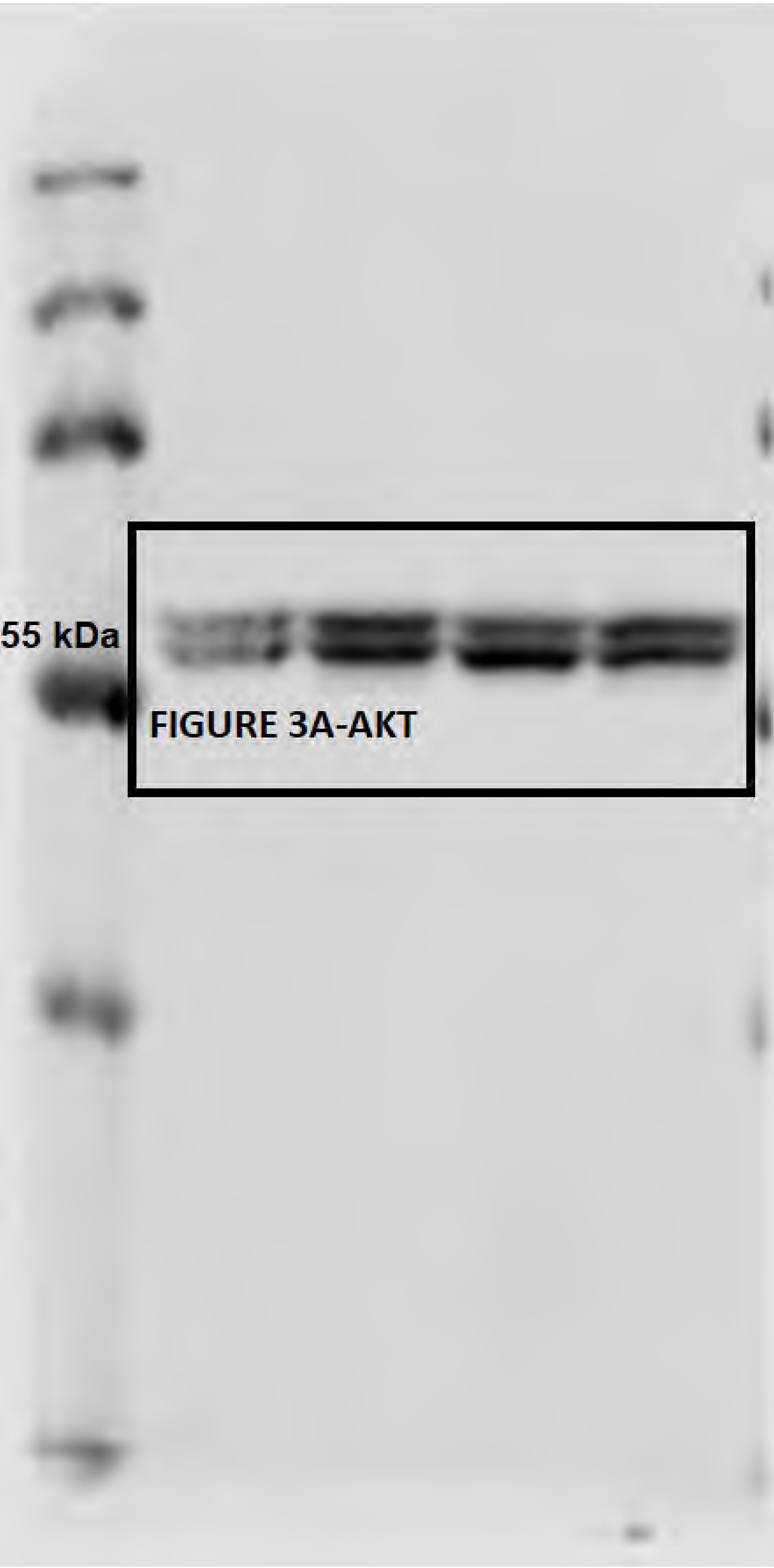
55 kDa
FIGURE 3A-AKT

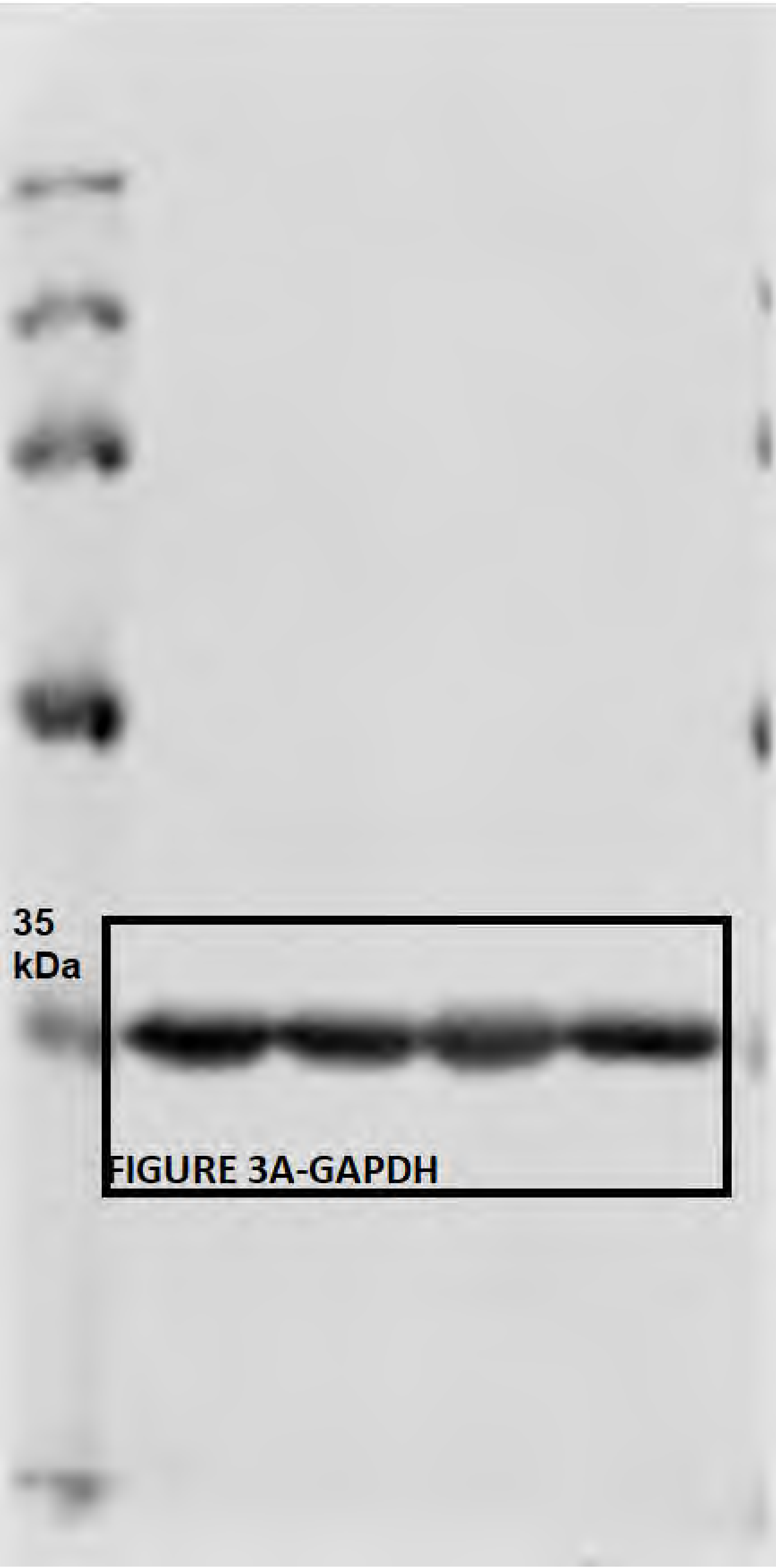
35
kDa
FIGURE 3A-GAPDH

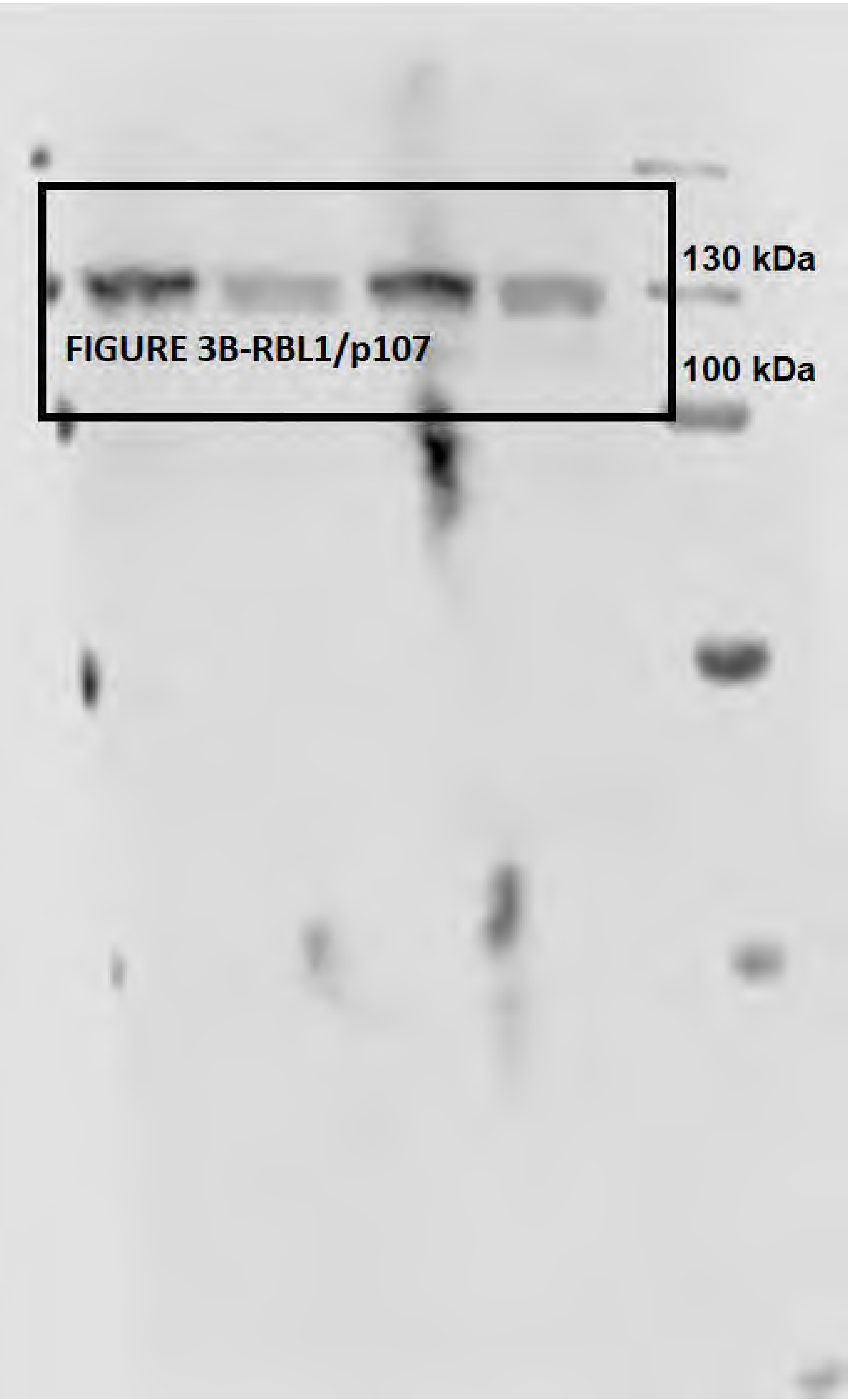
130 kDa
FIGURE 3B-RBL1/p107
100 kDa

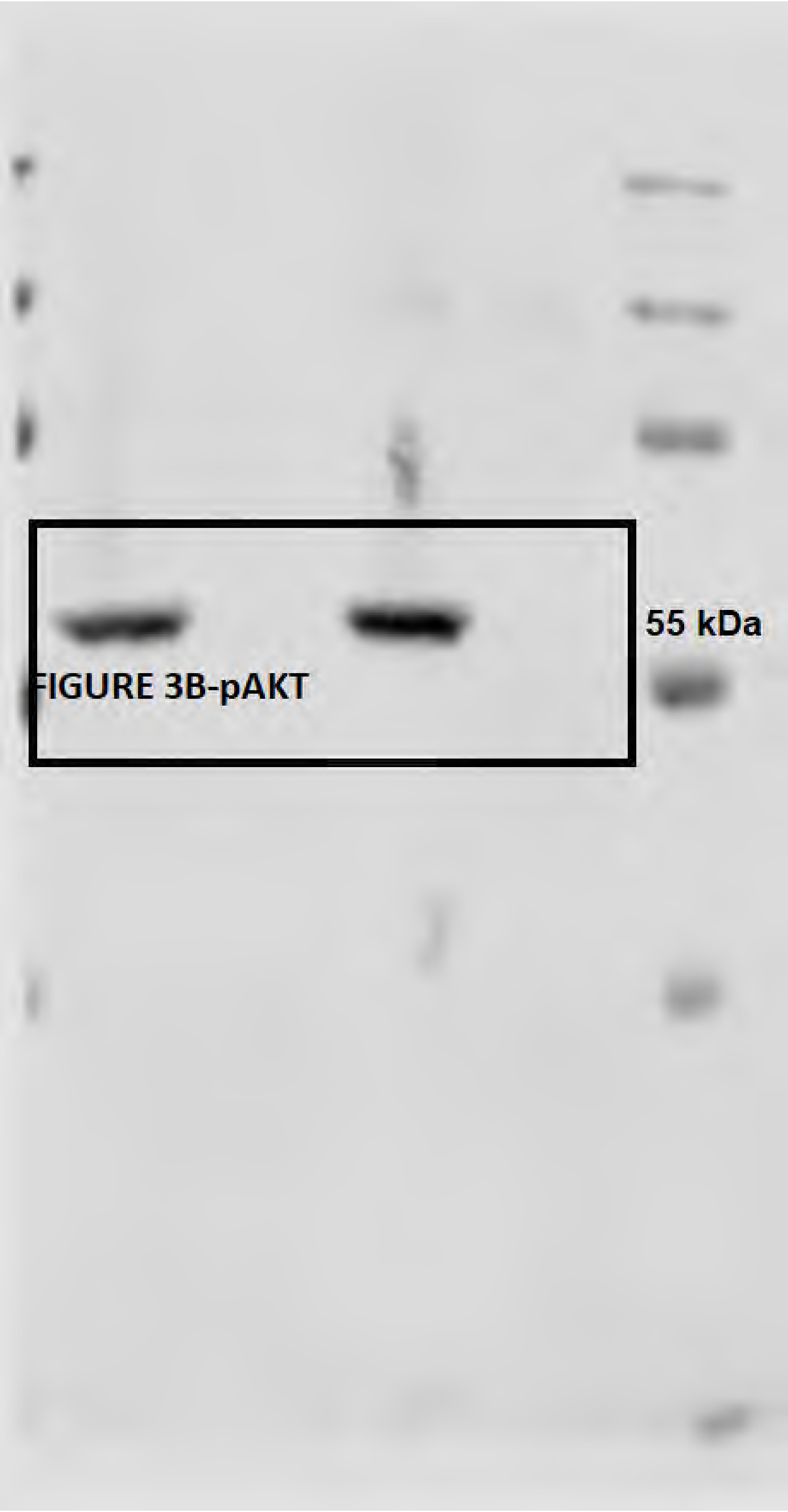

55 kDa
FIGURE 3B-pAKT

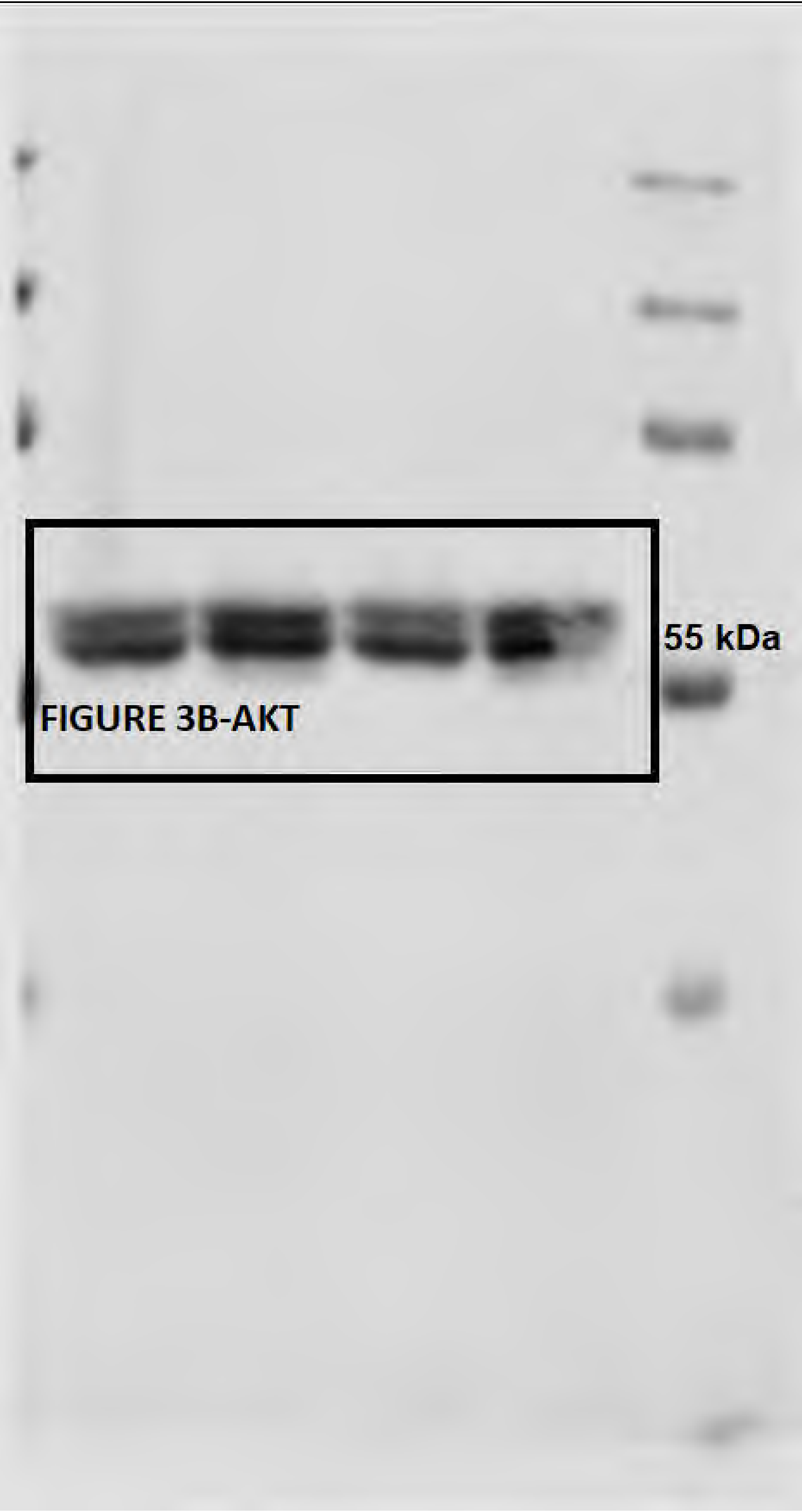
55 kDa
FIGURE 3B-AKT

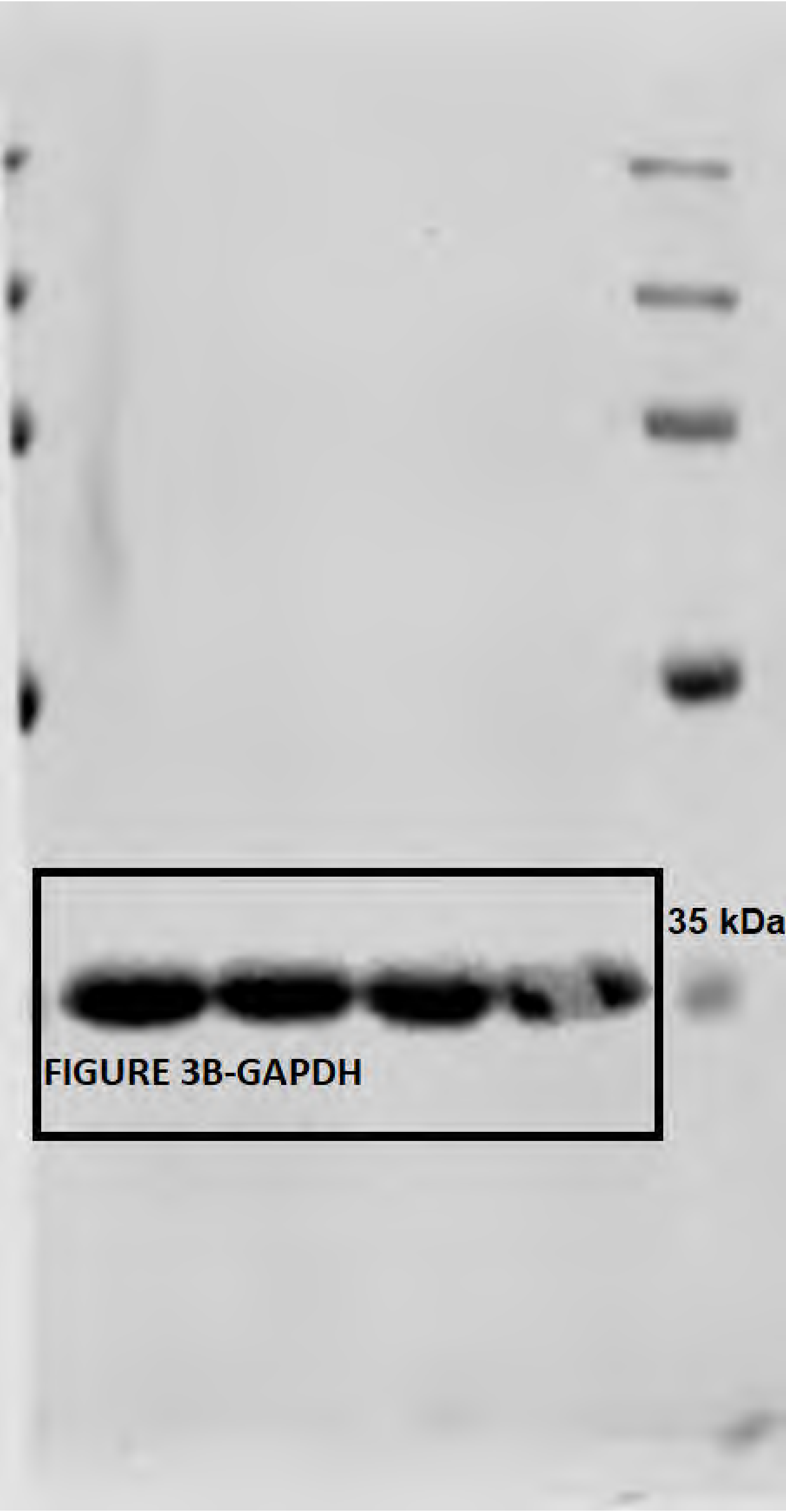
35 kDa
FIGURE 3B-GAPDH

FIGURE 3C
LEFT PANEL
A549
RBL1/p107

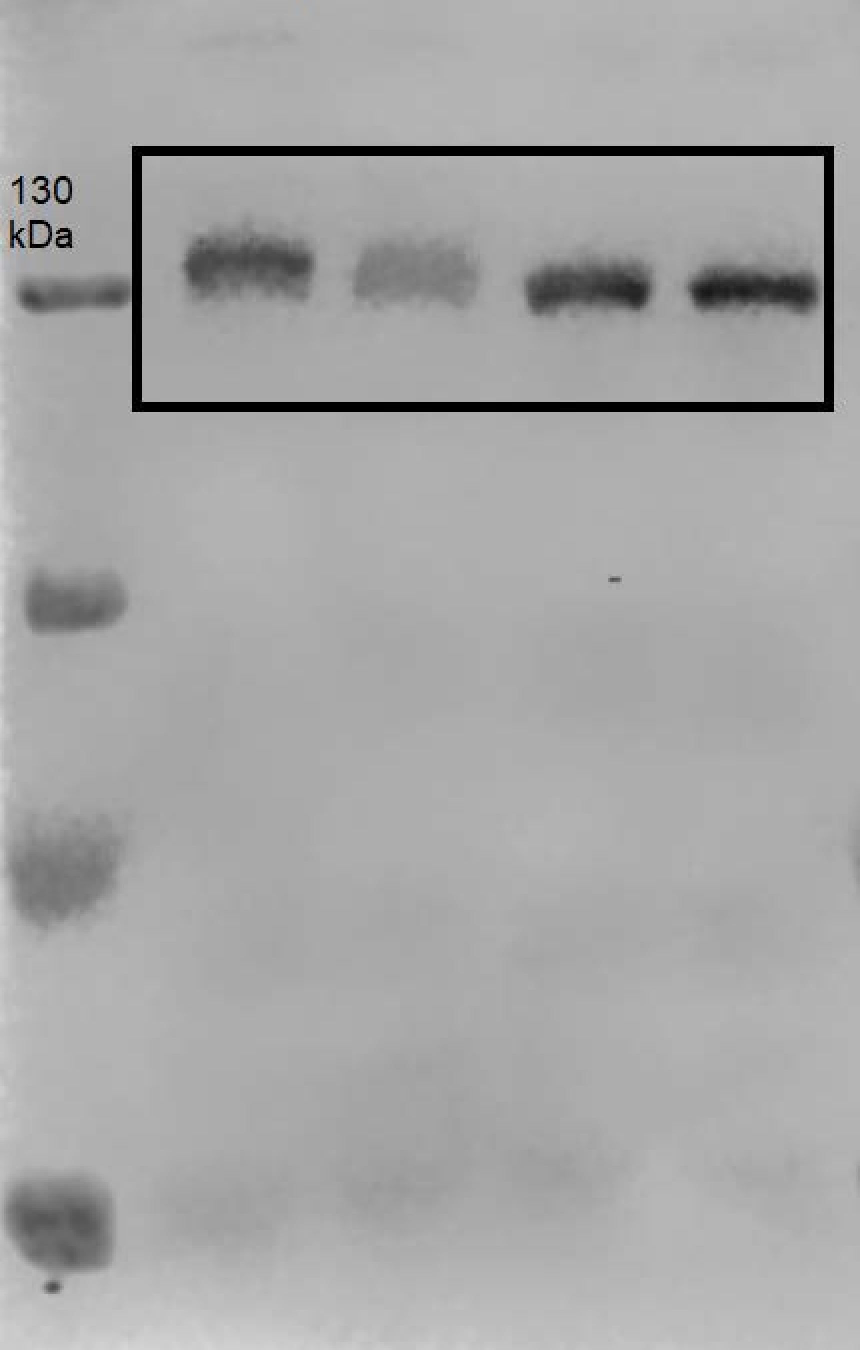

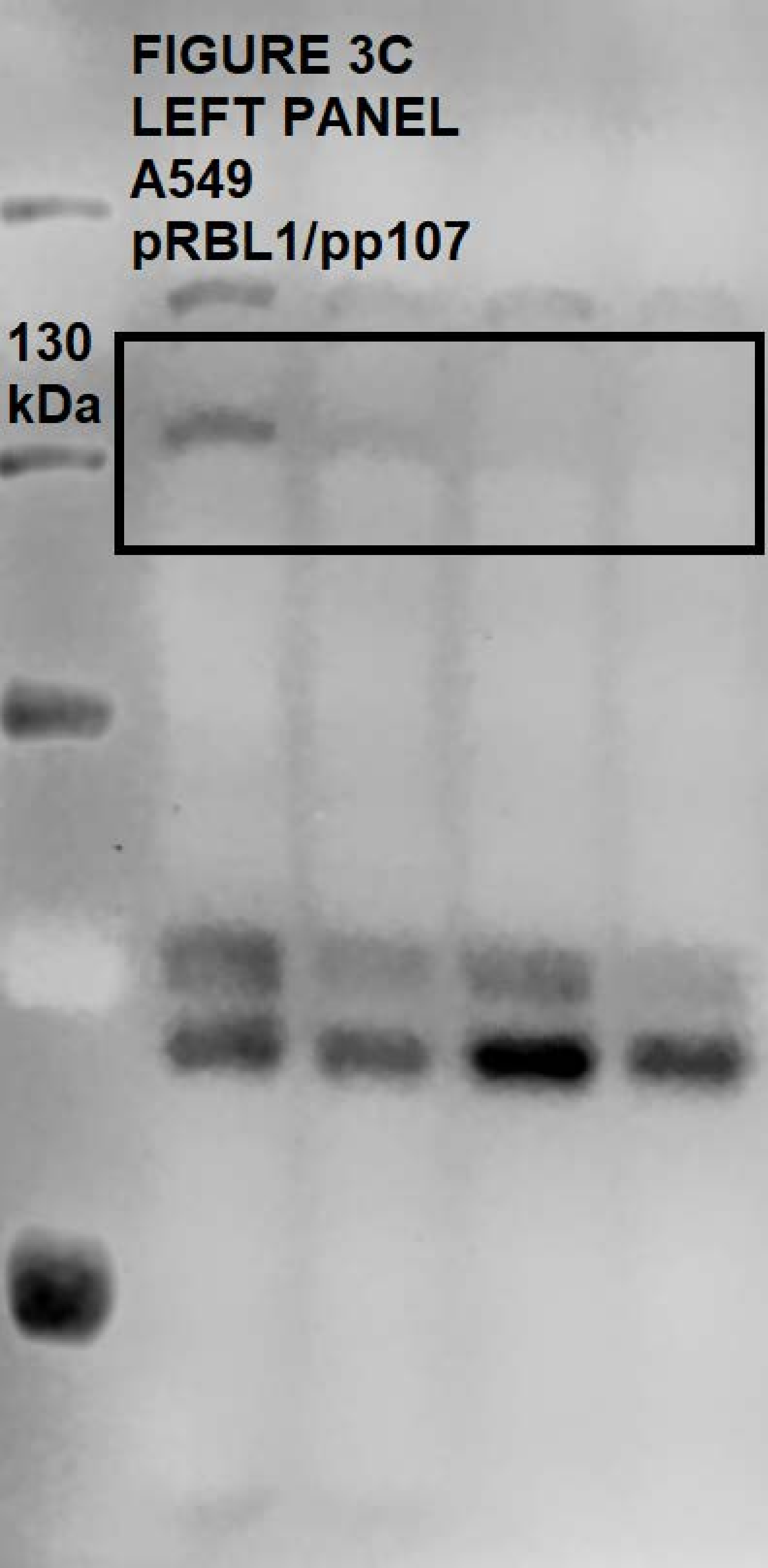
FIGURE 3C
LEFT PANEL
A549
pRBL1/pp107
130
kDa

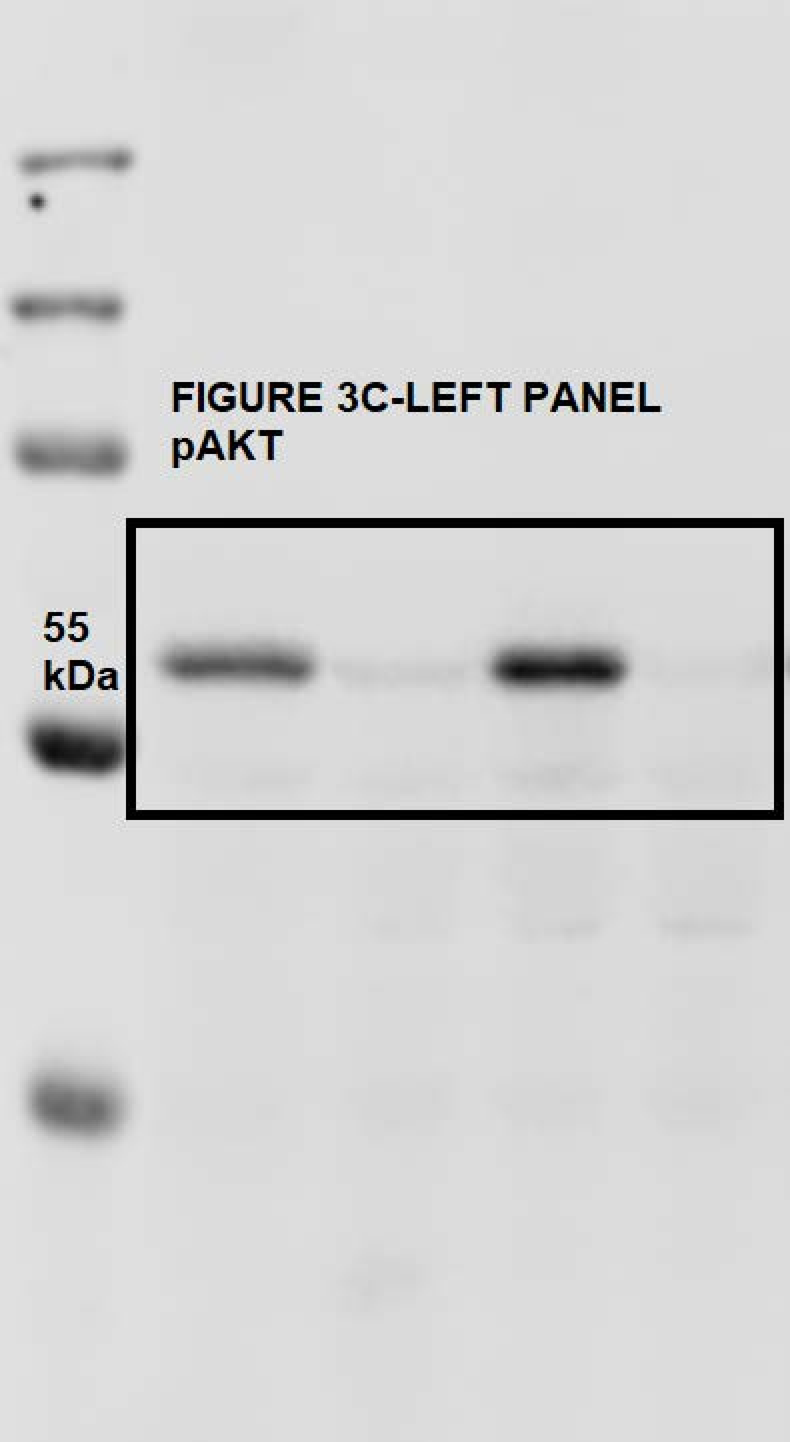
FIGURE 3C-LEFT PANEL
pAKT
55
kDa

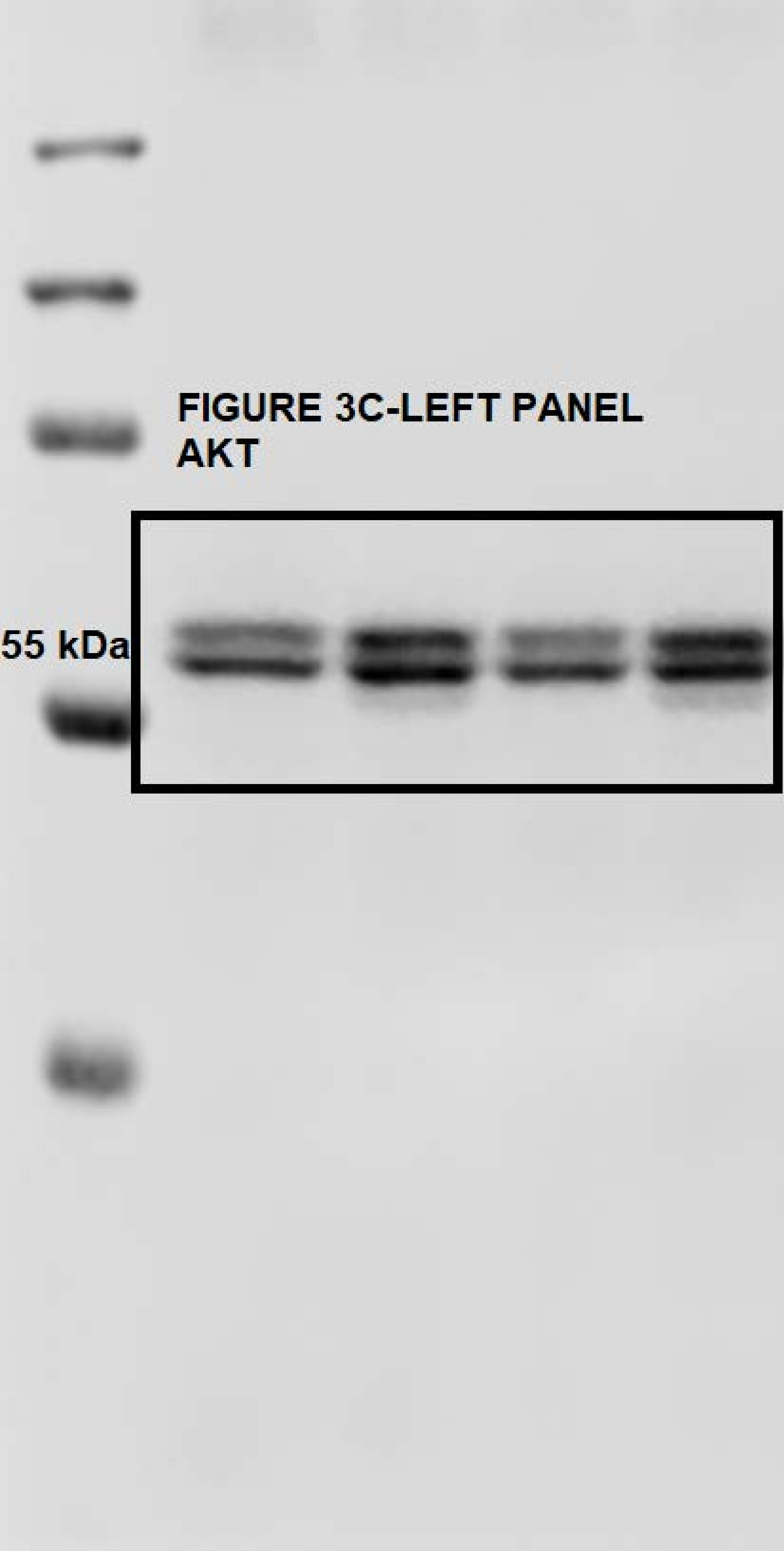

FIGURE 3C-LEFT PANEL
AKT

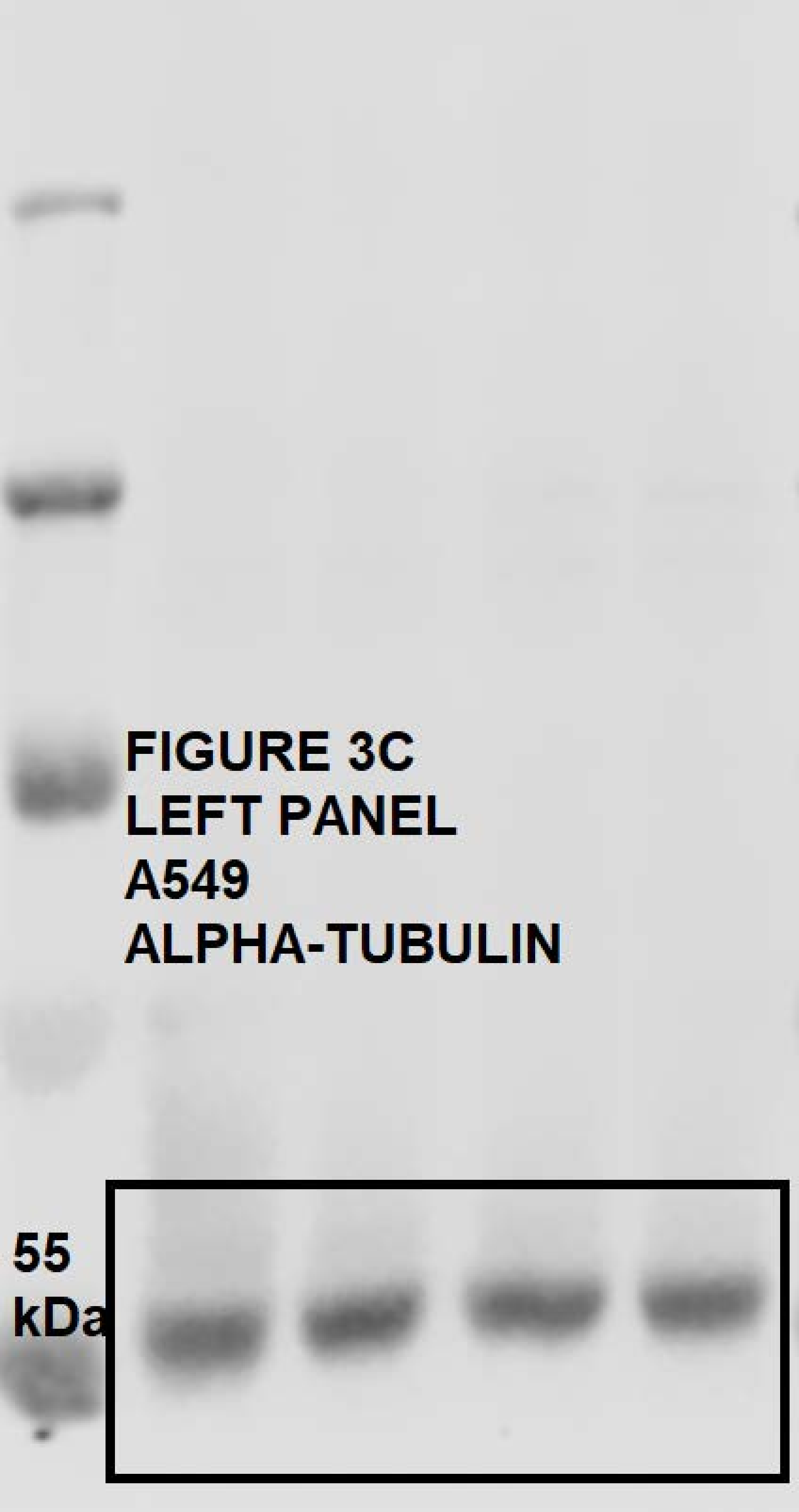
FIGURE 3C
LEFT PANEL
A549
ALPHA-TUBULIN
55
kDa

FIGURE 3C
RIGHT PANEL
MSTO-211H
RBL1/p107

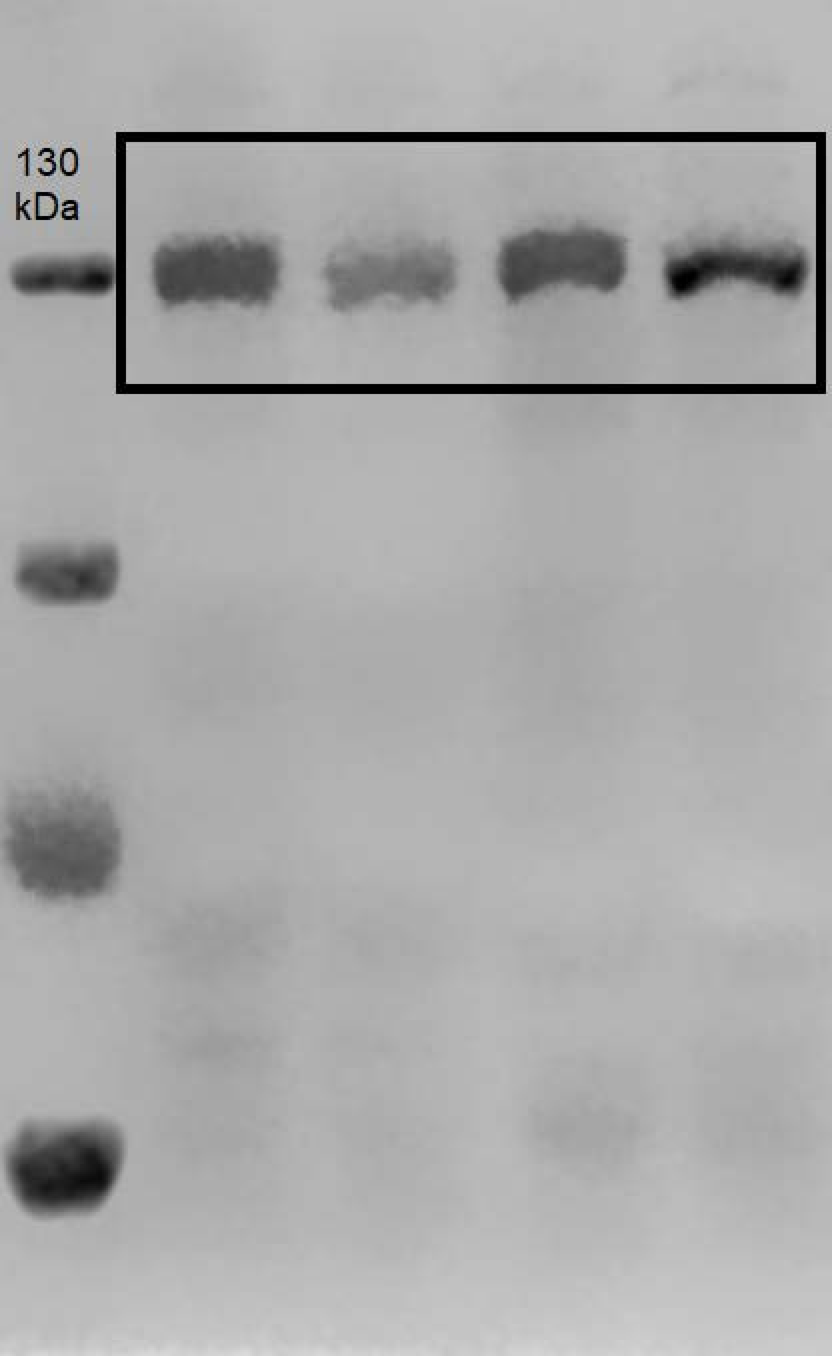

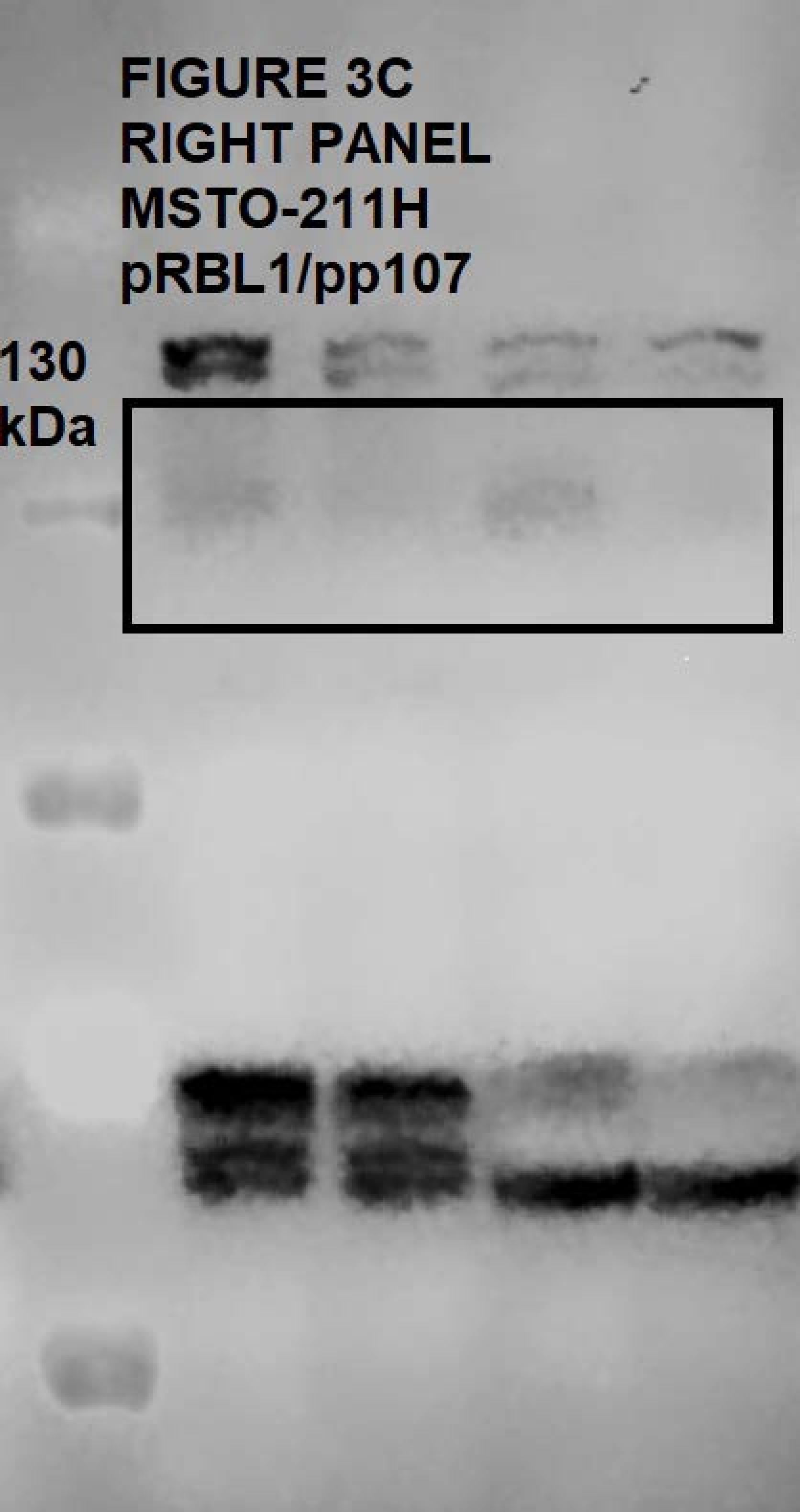
FIGURE 3C
RIGHT PANEL
MSTO-211H
pRBL1/pp107
130
kDa

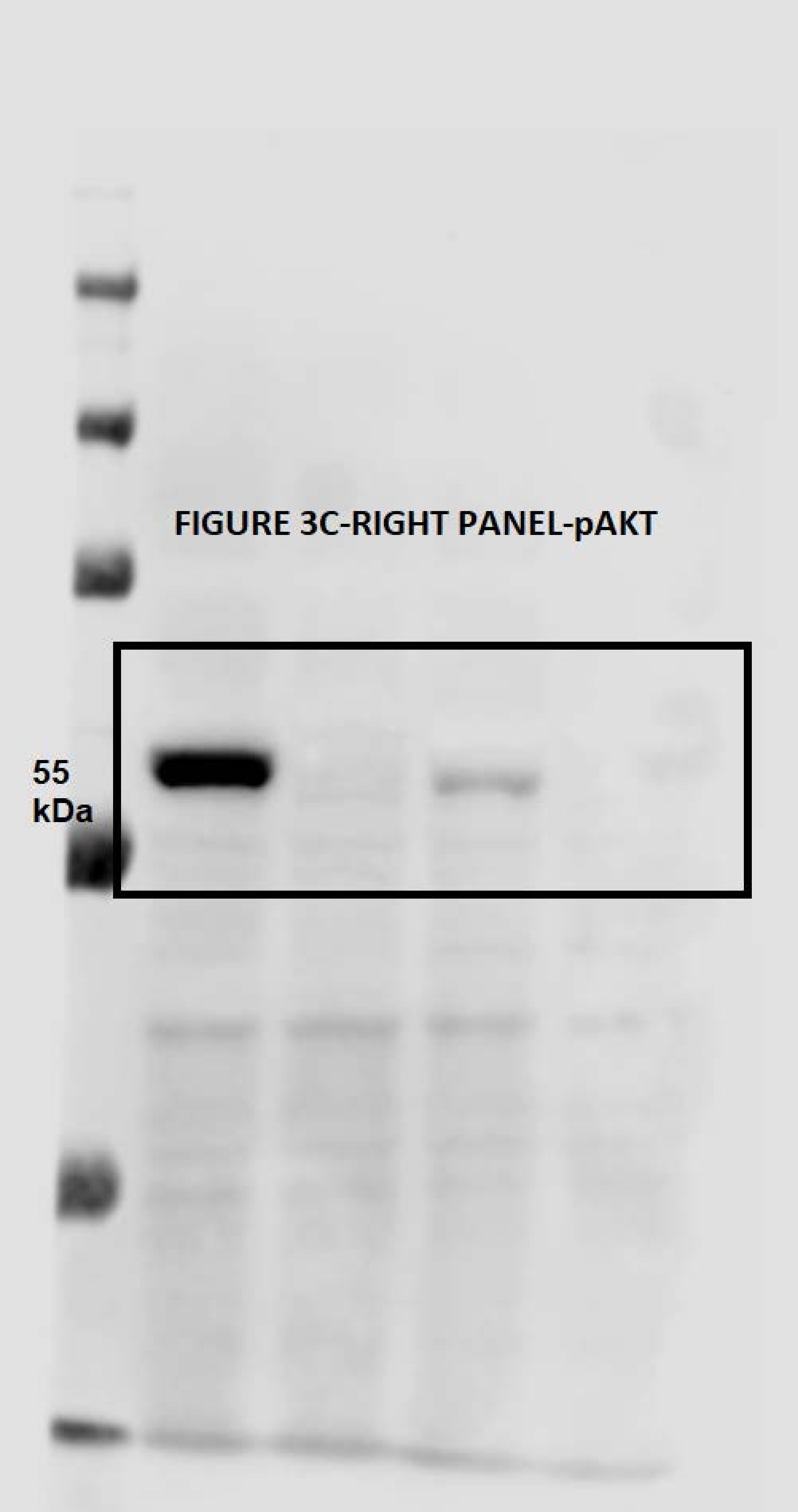
FIGURE 3C-RIGHT PANEL-pAKT
55
kDa

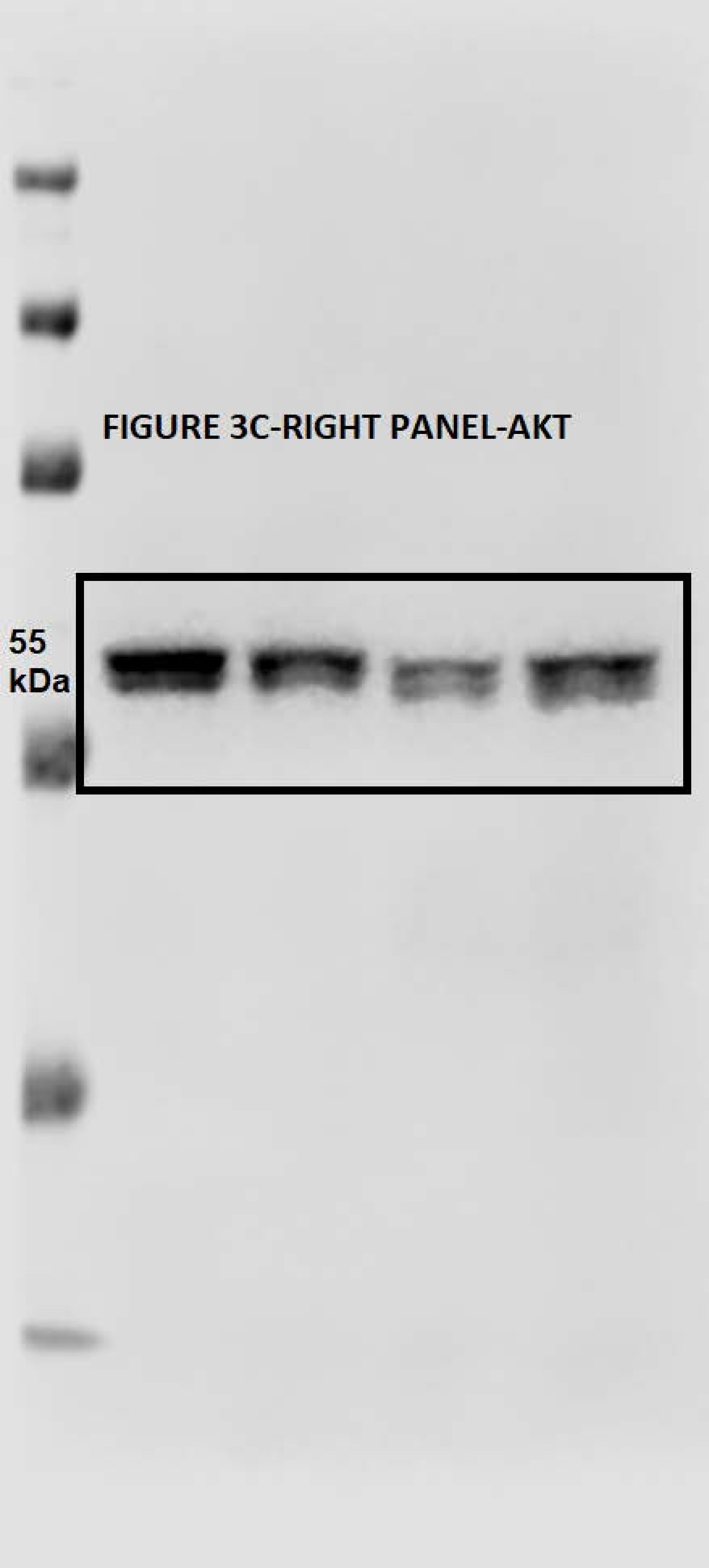
FIGURE 3C-RIGHT PANEL-AKT
55
kDa

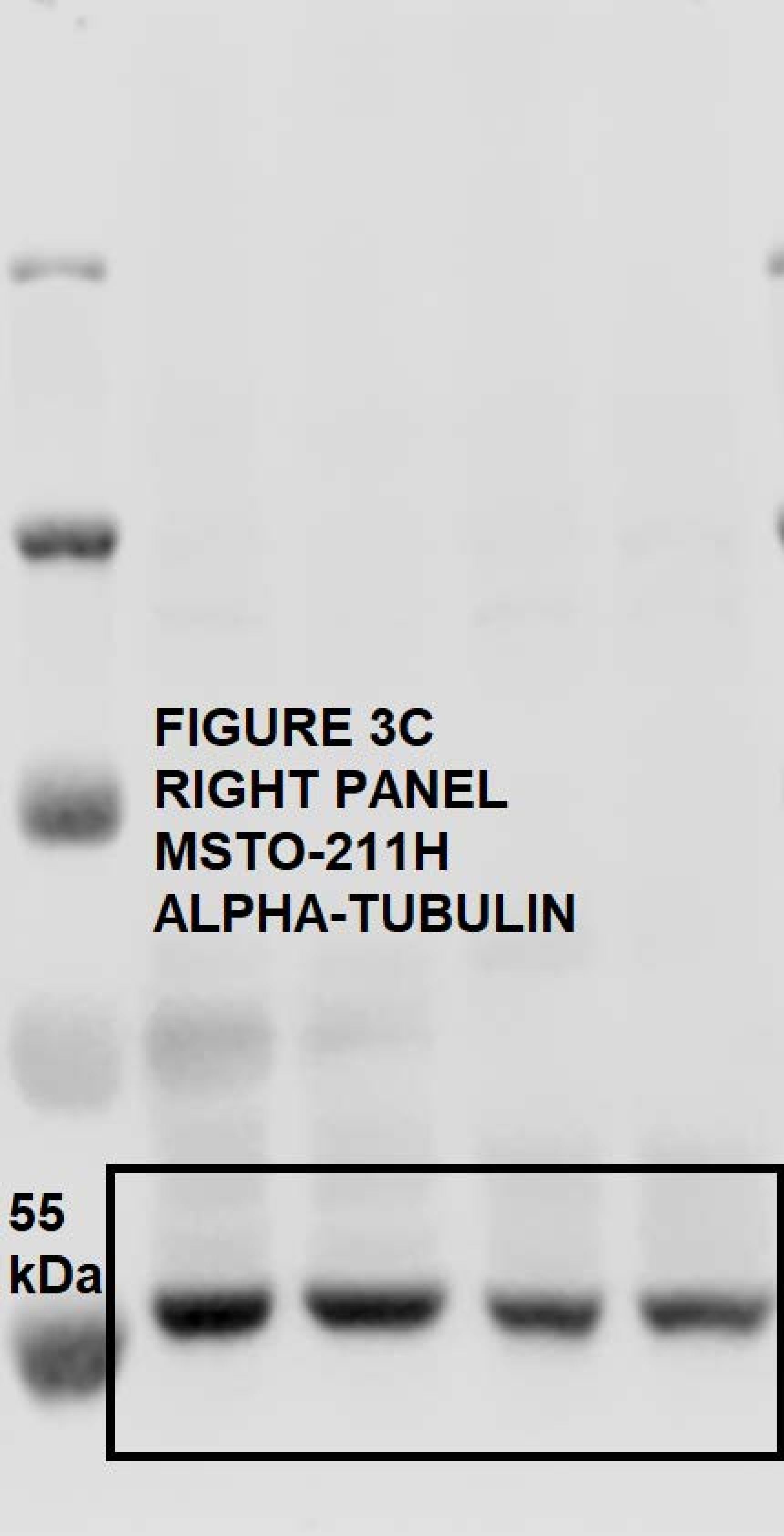
FIGURE 3C
RIGHT PANEL
MSTO-211H
ALPHA-TUBULIN
55
kDa

FIGURE 4A-RBL1/p107

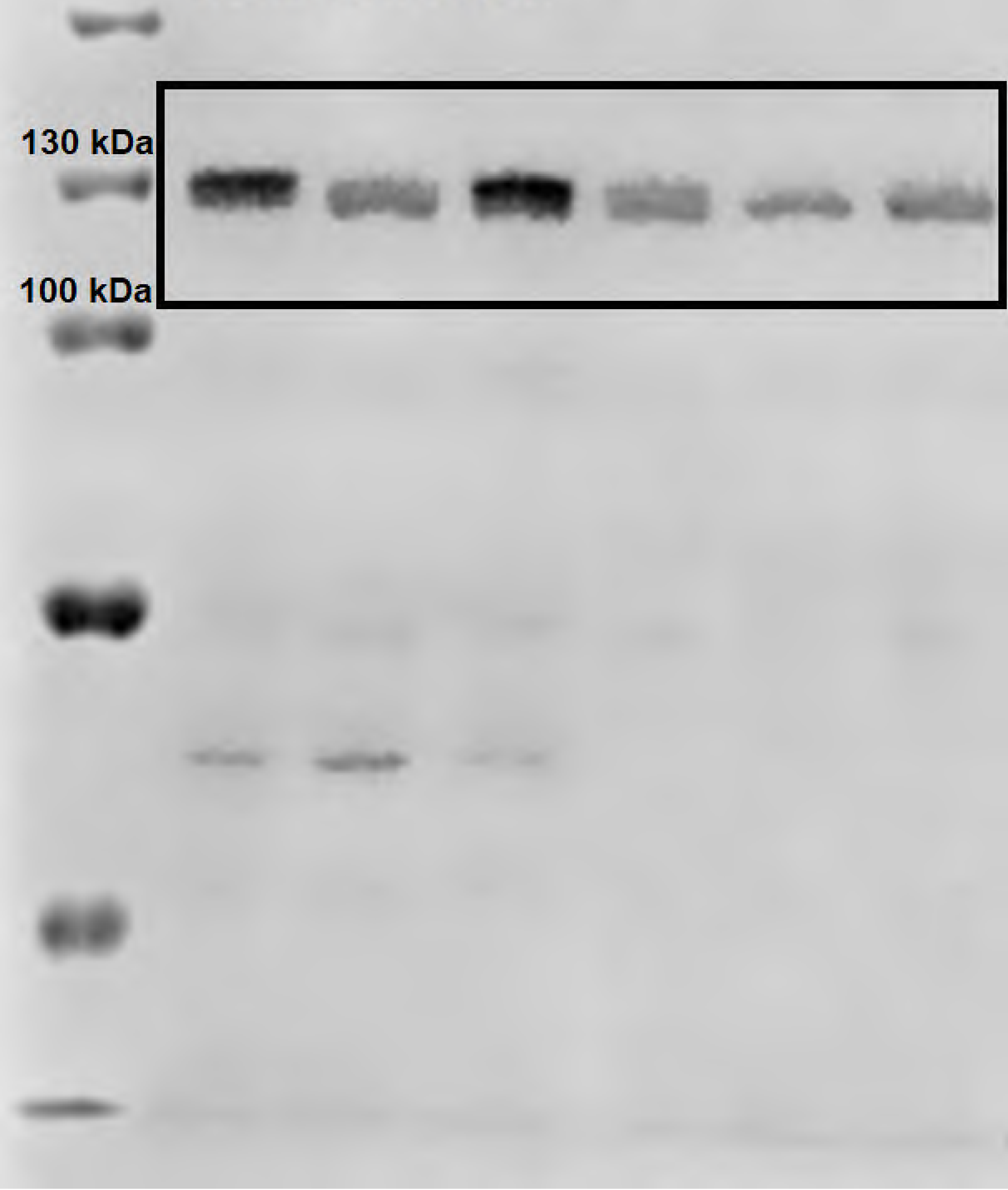

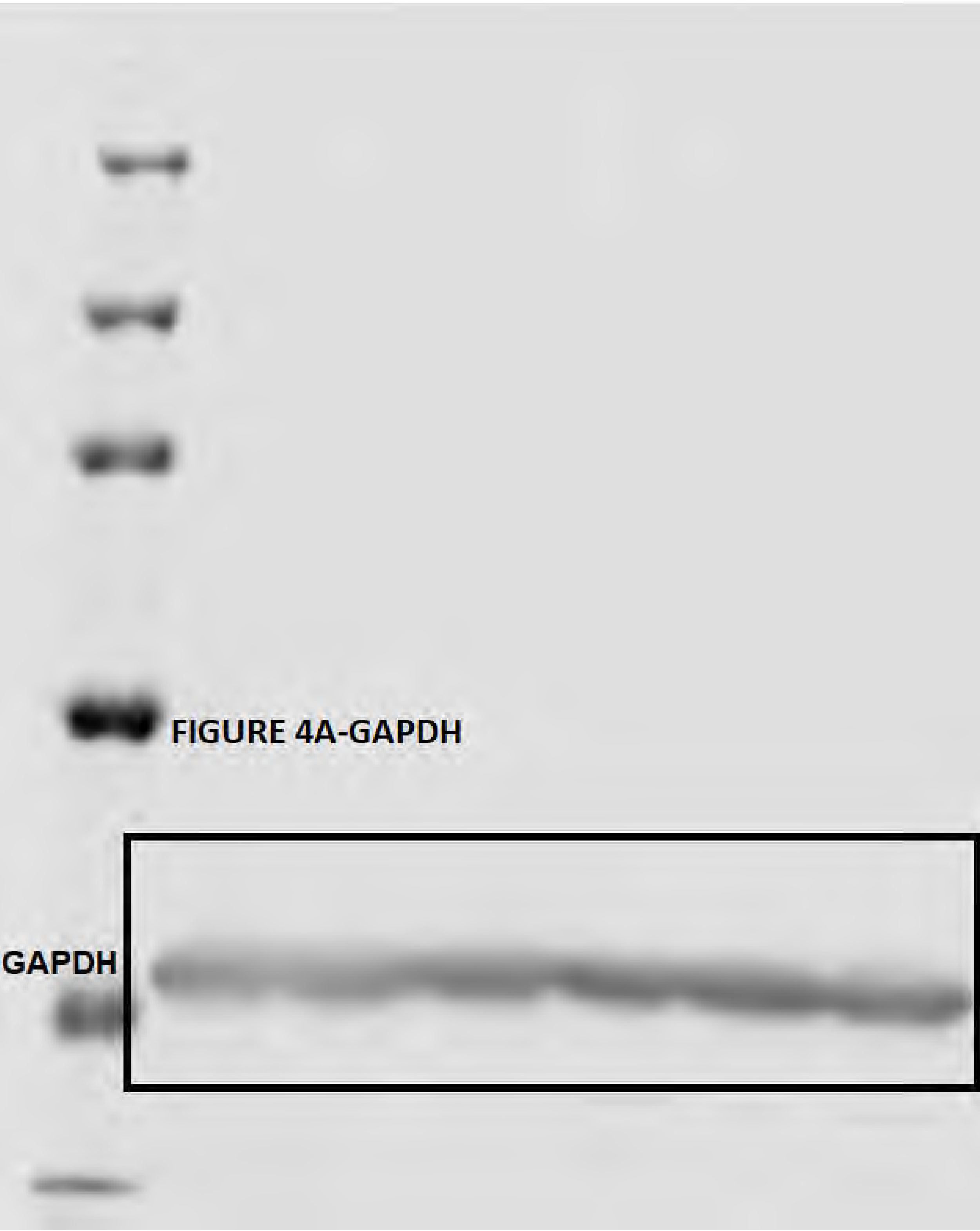
FIGURE 4A-GAPDH
GAPDH

FIGURE 4B
A549
RBL1/p107

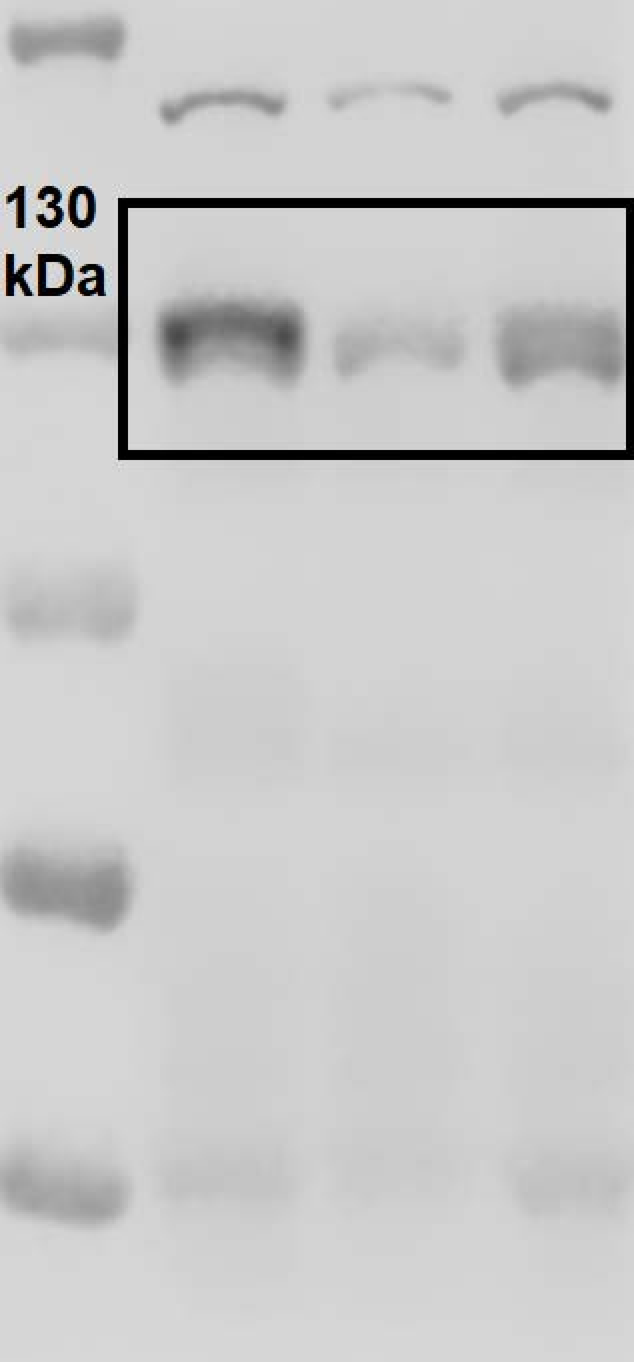

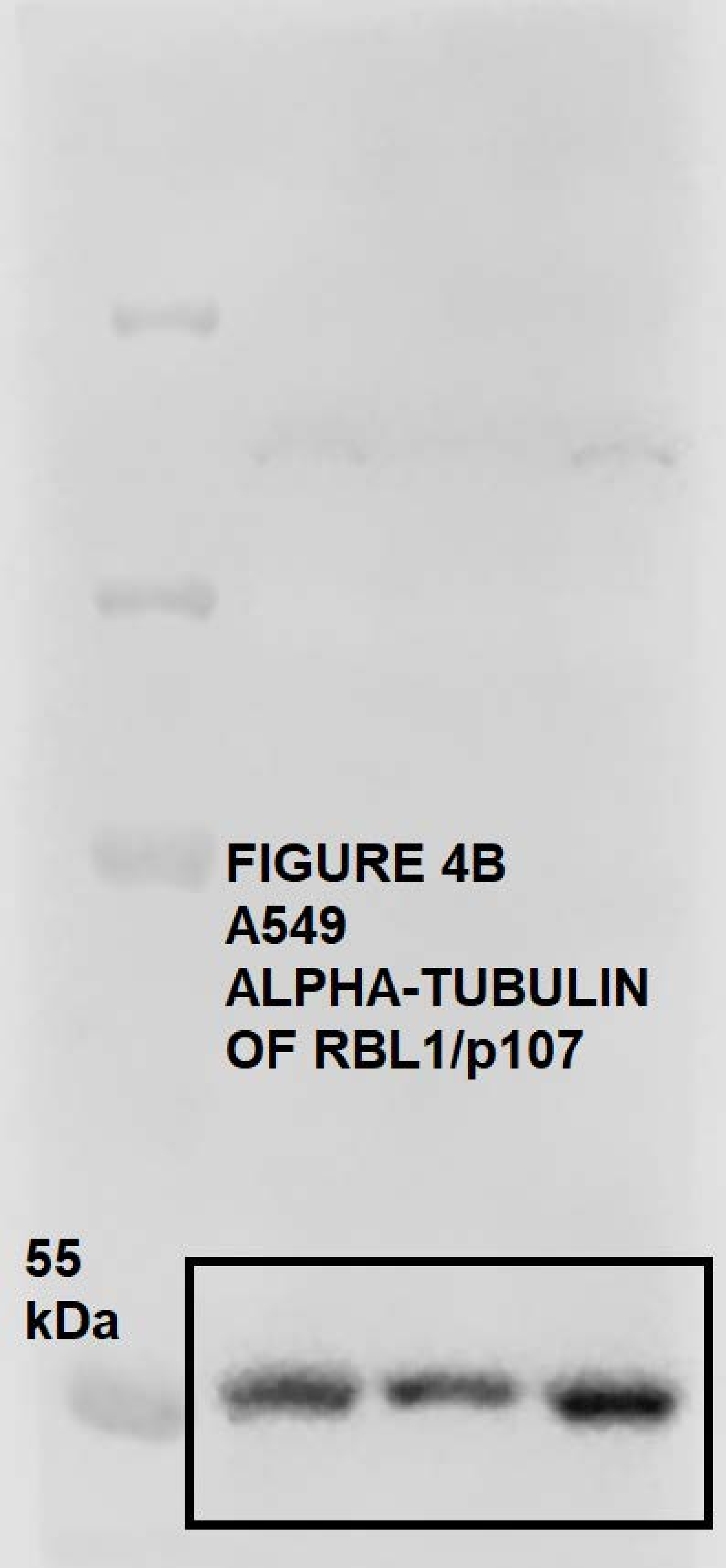
FIGURE 4B
A549
ALPHA-TUBULIN
OF RBL1/p107
55
kDa

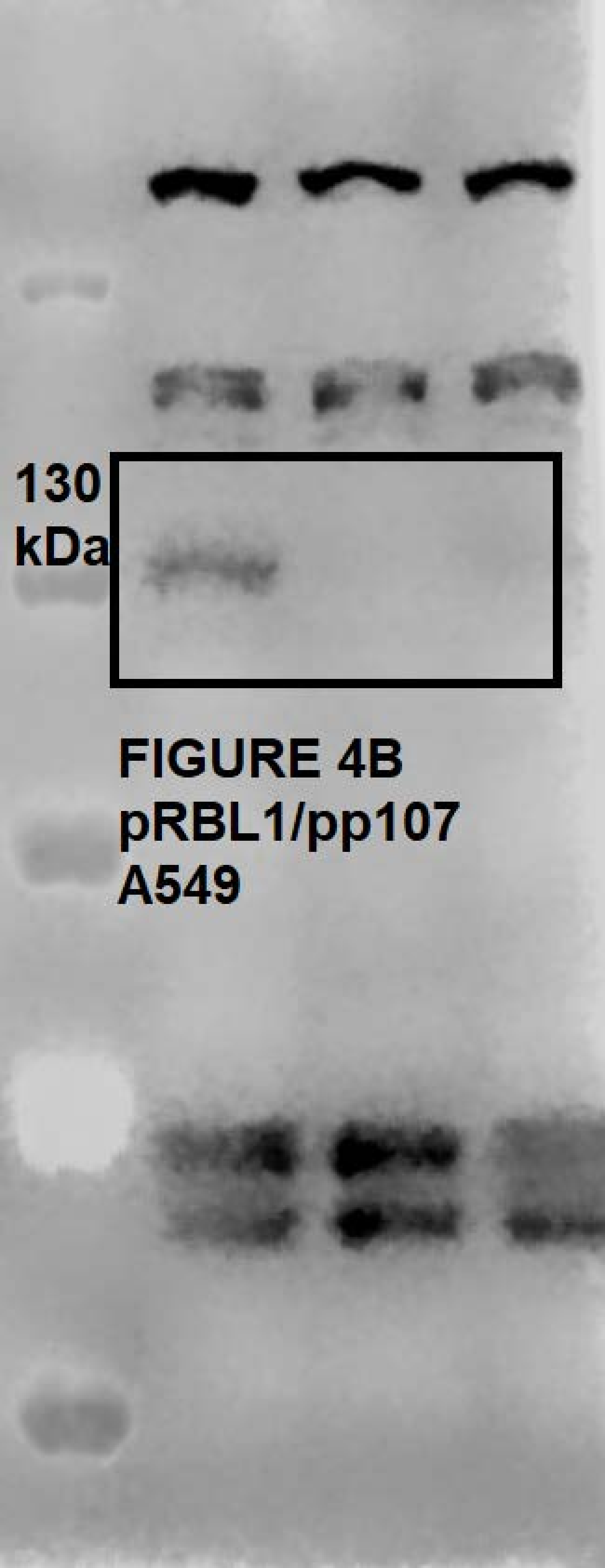

FIGURE 4B
pRBL1/pp107
A549

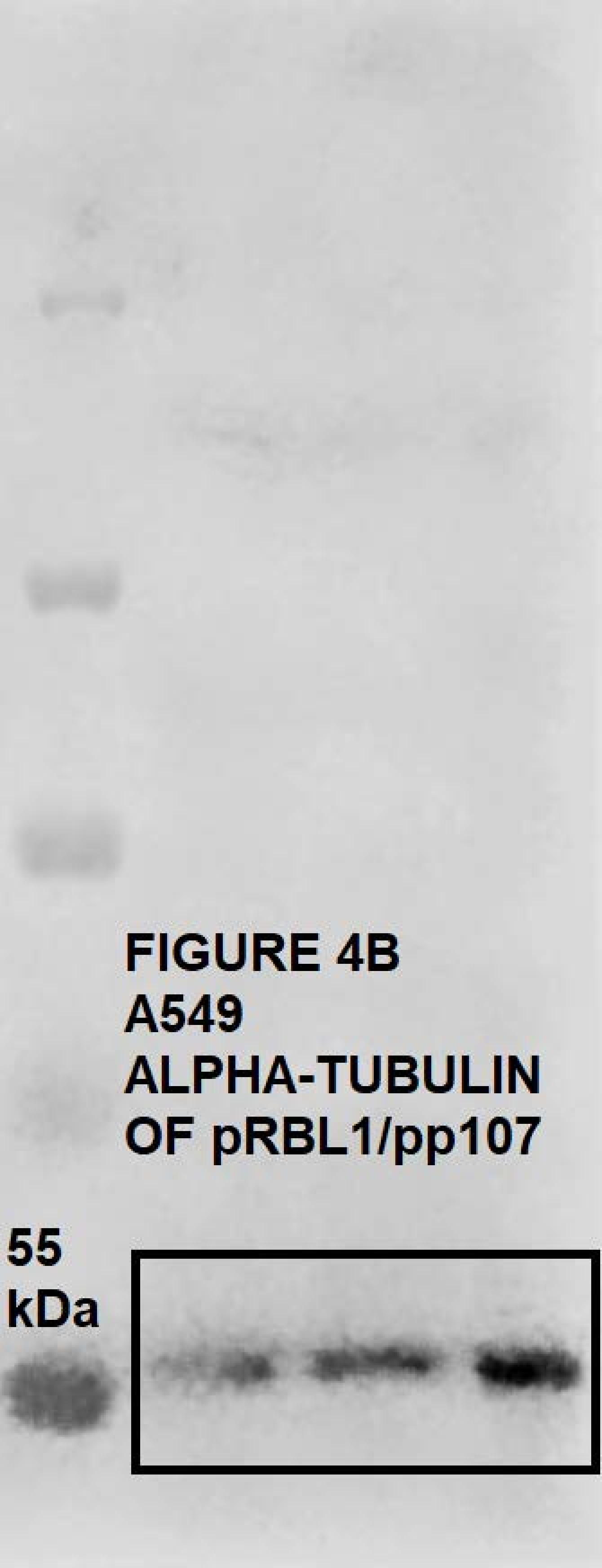
FIGURE 4B
A549
ALPHA-TUBULIN
OF pRBL1/pp107
55
kDa

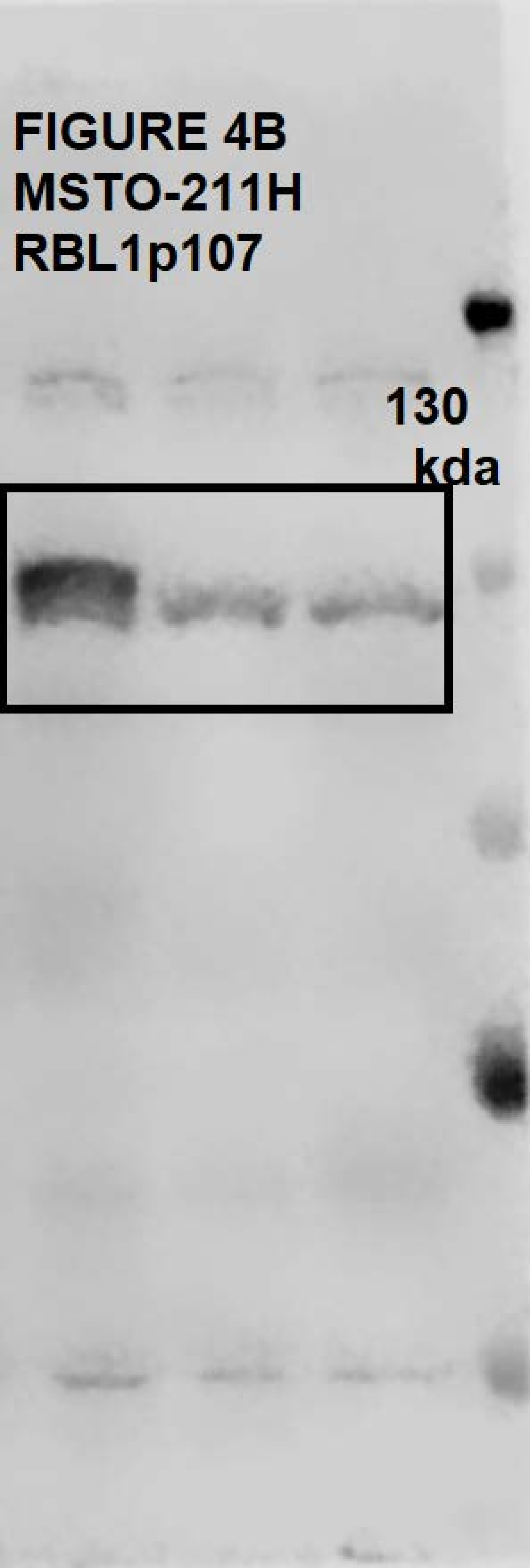
FIGURE 4B
MSTO-211H
RBL1p107
130
kda

FIGURE 4B
MSTO-211H
ALPHA-TUBULIN
OF RBL1/p107

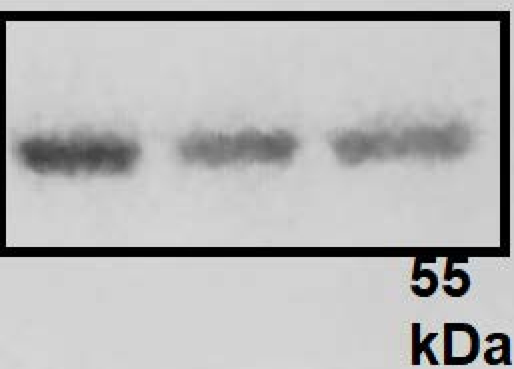

55
kDa

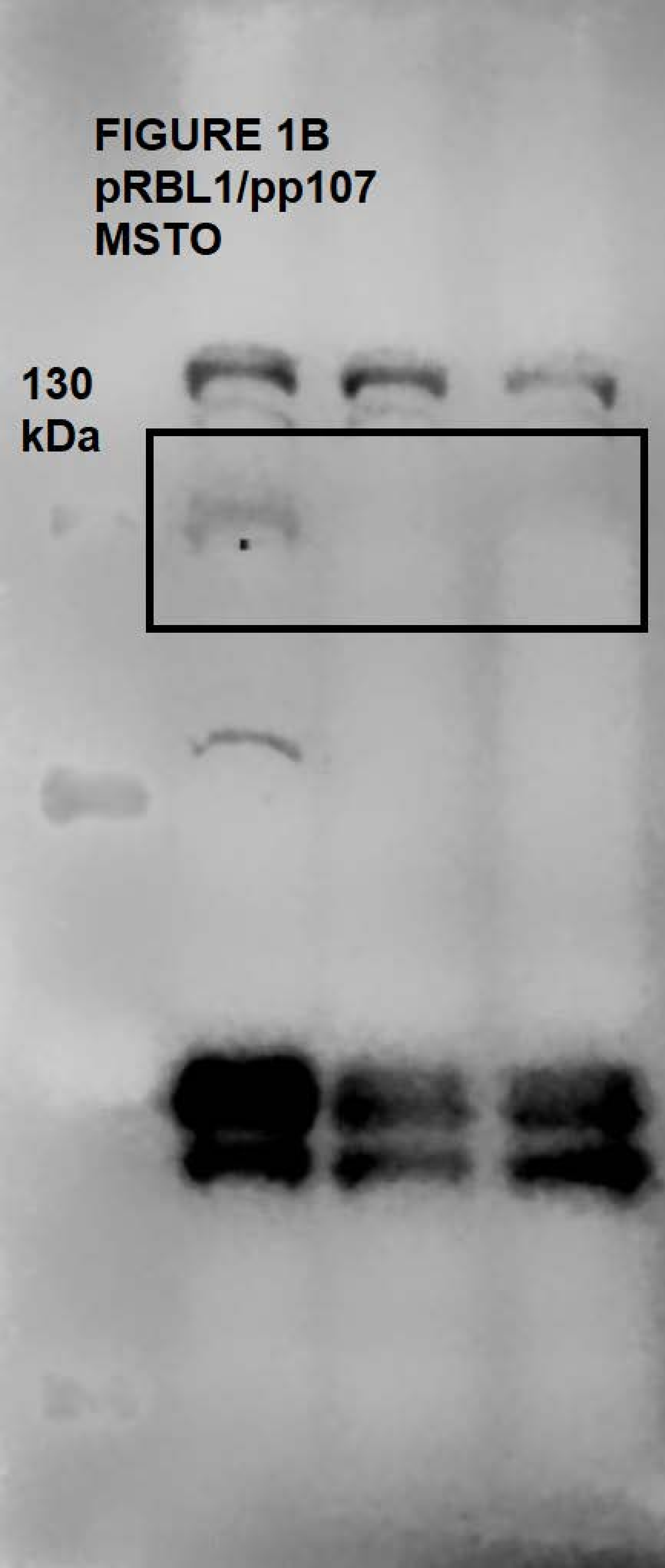
FIGURE 1B
pRBL1/pp107
MSTO
130
kDa

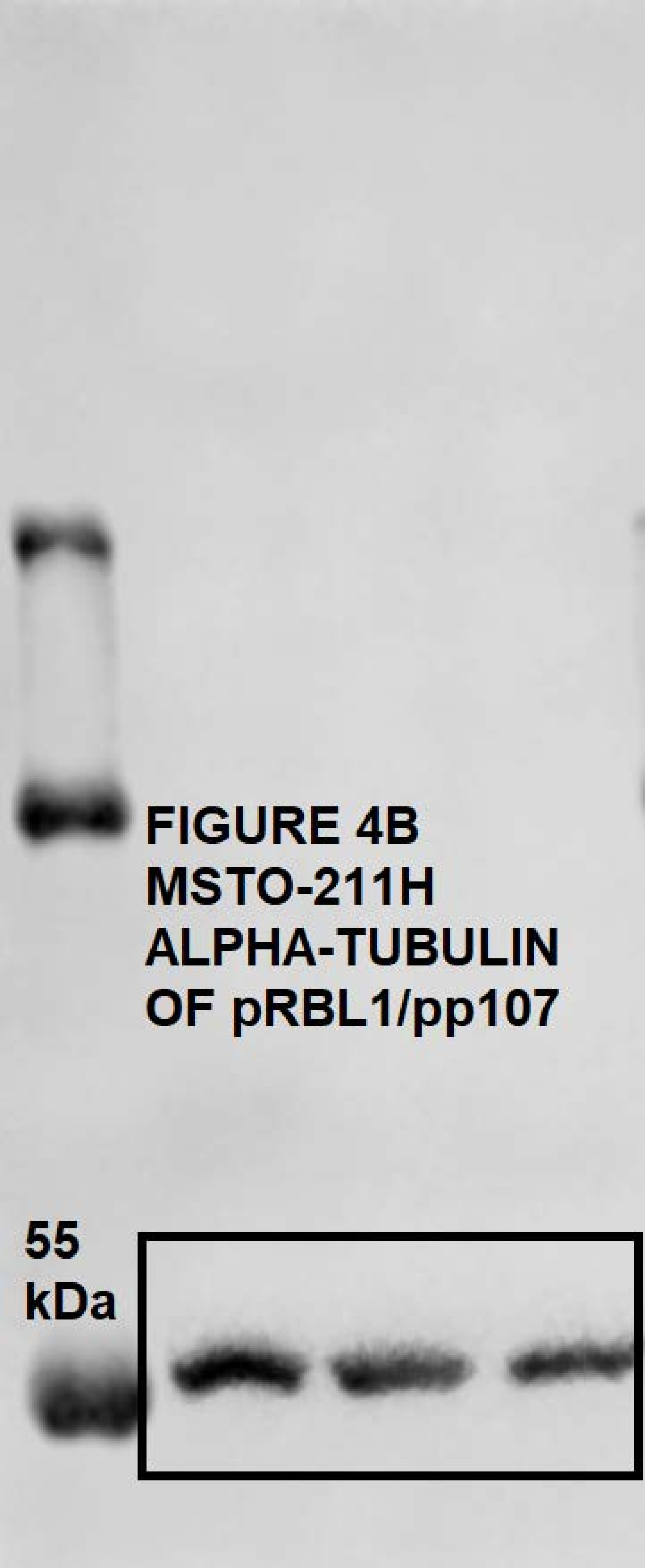
FIGURE 4B
MSTO-211H
ALPHA-TUBULIN
OF pRBL1/pp107
55
kDa

FIGURE 4D LEFT PANEL

RBL1/p107

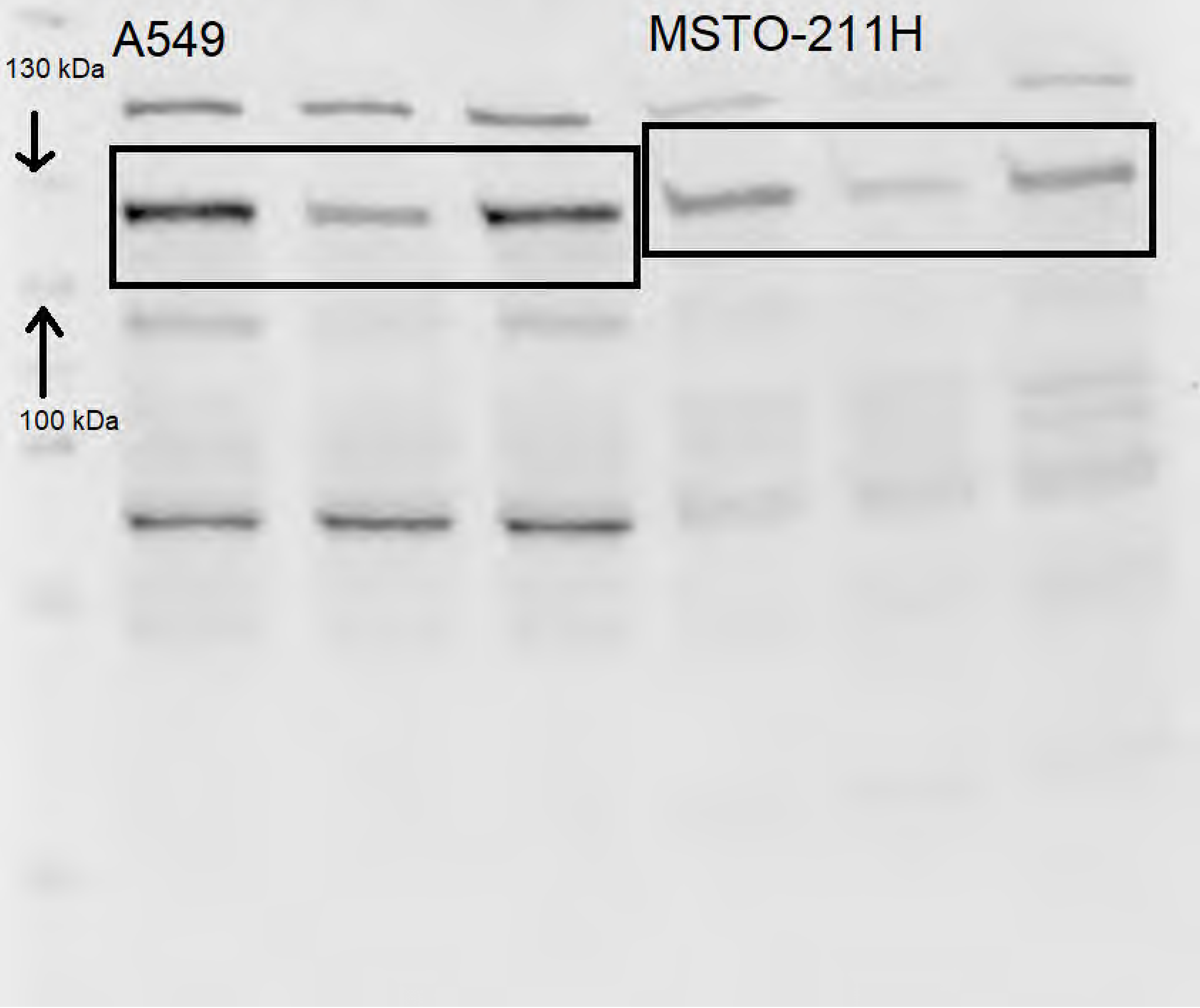

FIGURE 4D LEFT PANEL
ACTIN

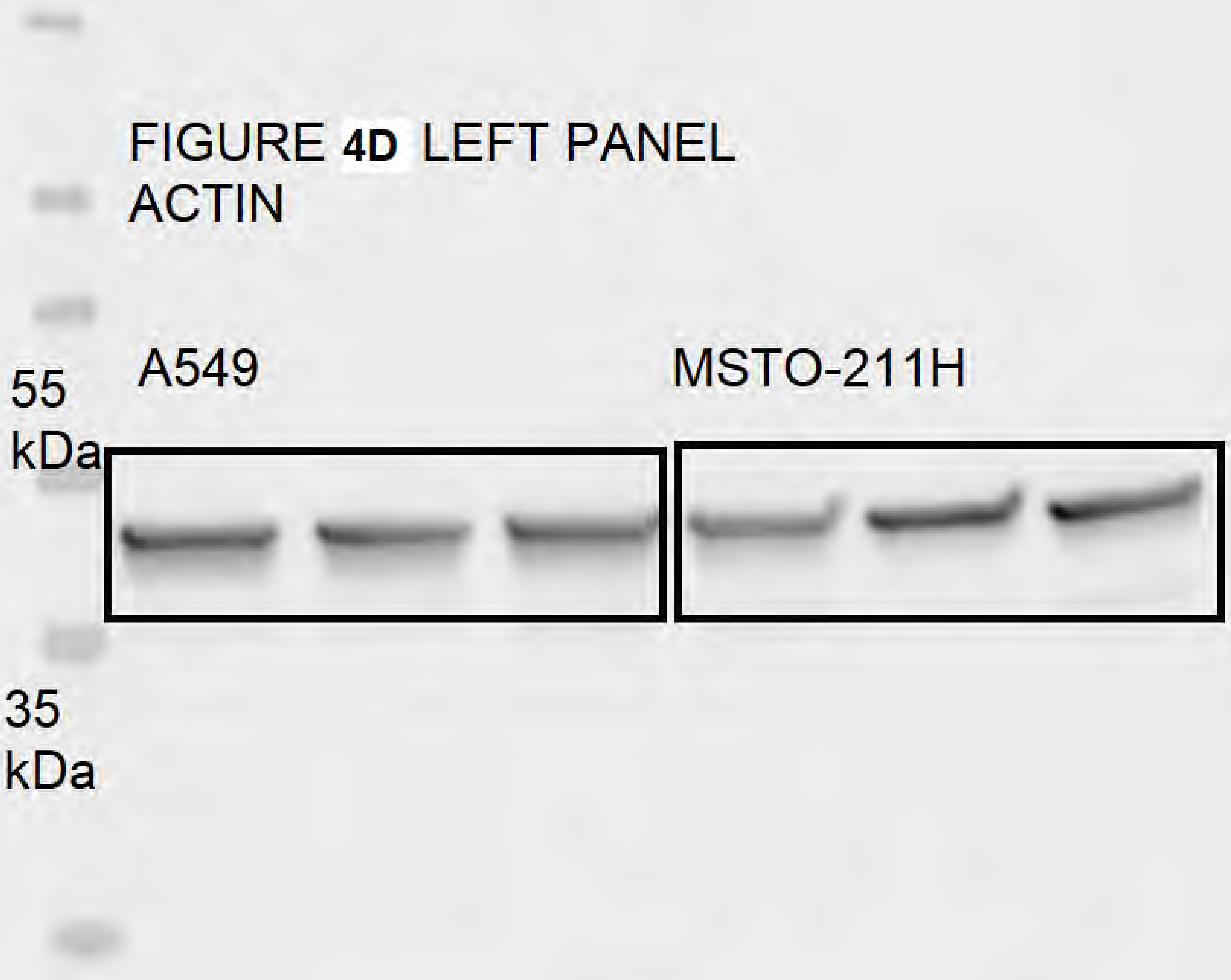

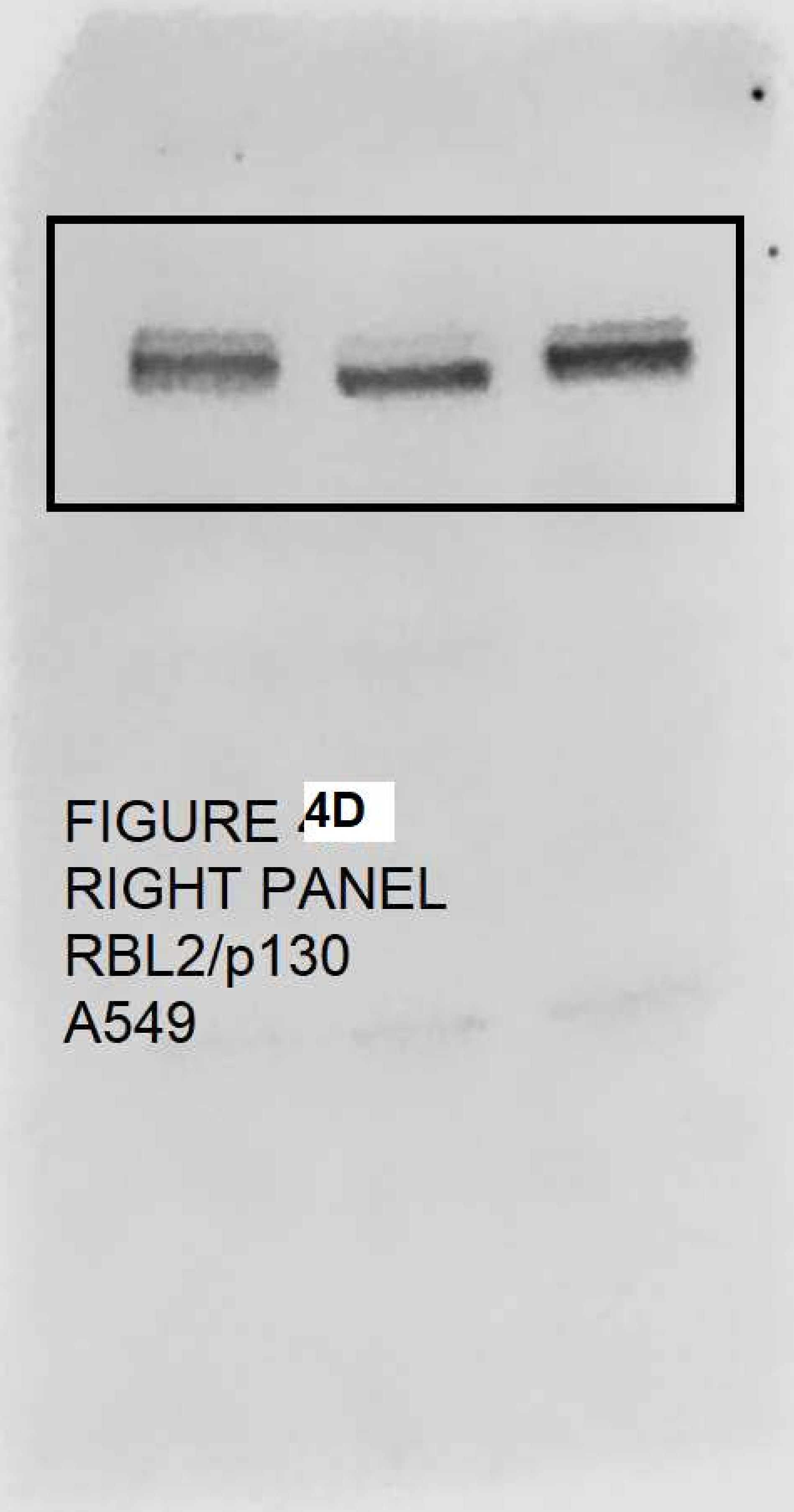
FIGURE 4D
RIGHT PANEL
RBL2/p130
A549

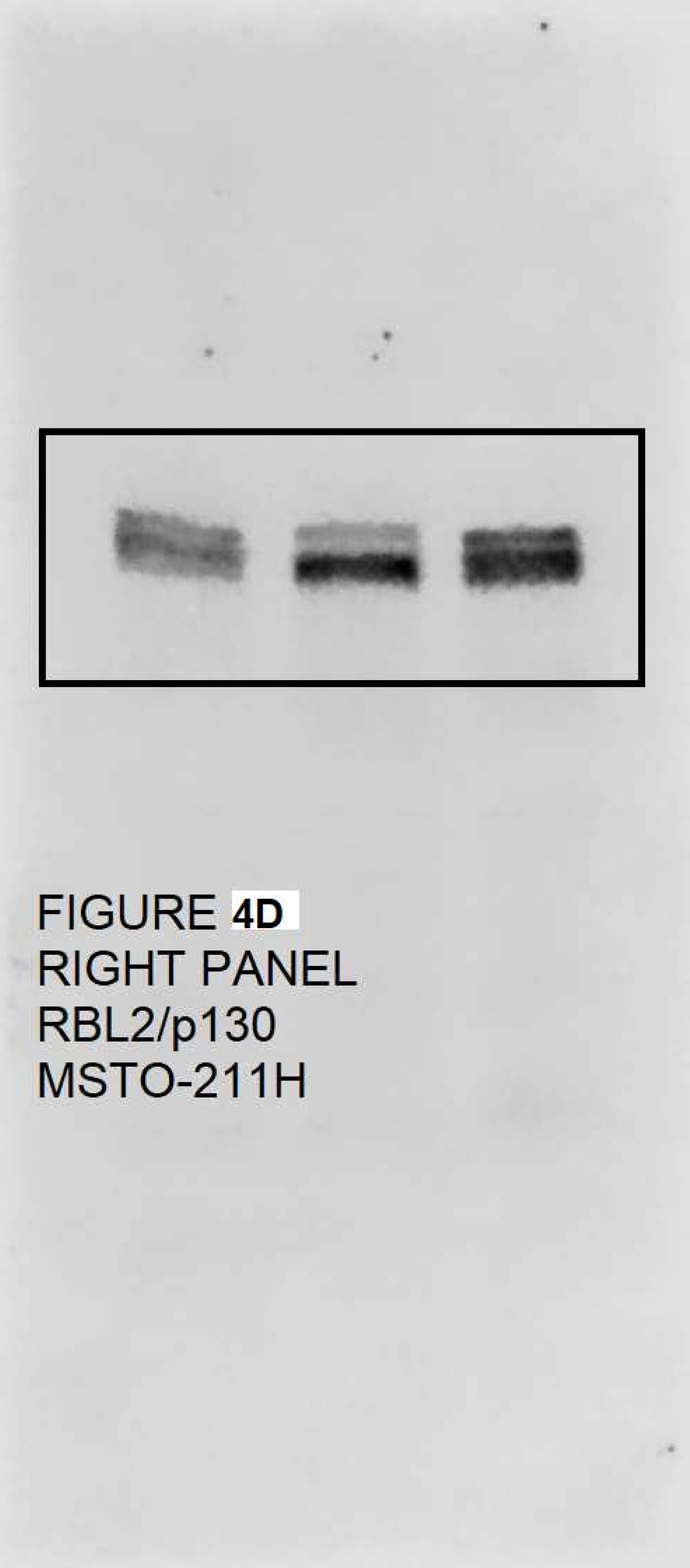
FIGURE 4D
RIGHT PANEL
RBL2/p130
MSTO-211H

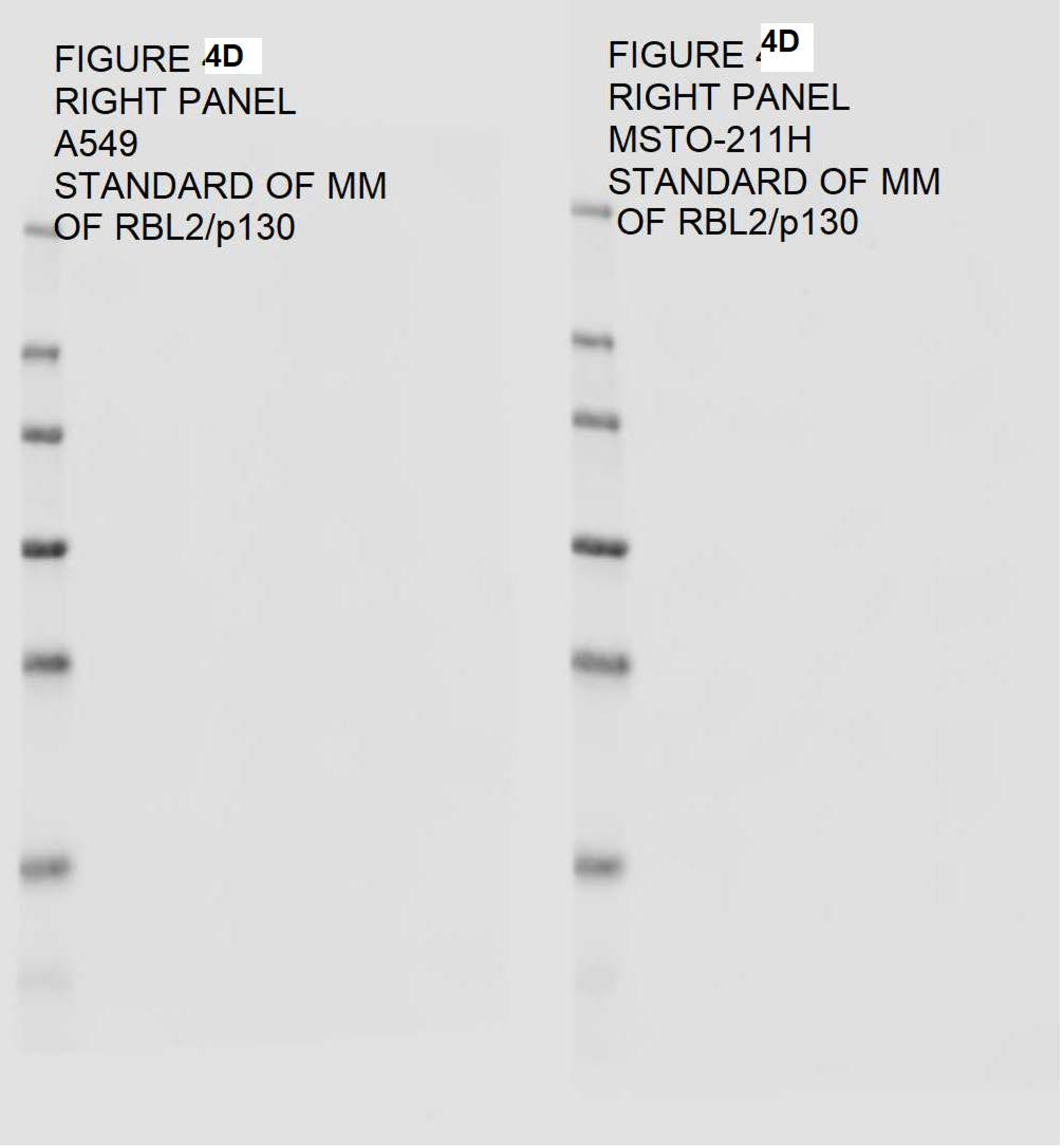
FIGURE 4D
RIGHT PANEL
A549
STANDARD OF MM
OF RBL2/p130
FIGURE 4D
RIGHT PANEL
MSTO-211H
STANDARD OF MM
OF RBL2/p130

FIGURE 4D
RIGHT PANEL
MERGE OF RBL2/p130 AND OF THE STANDARD OF MM

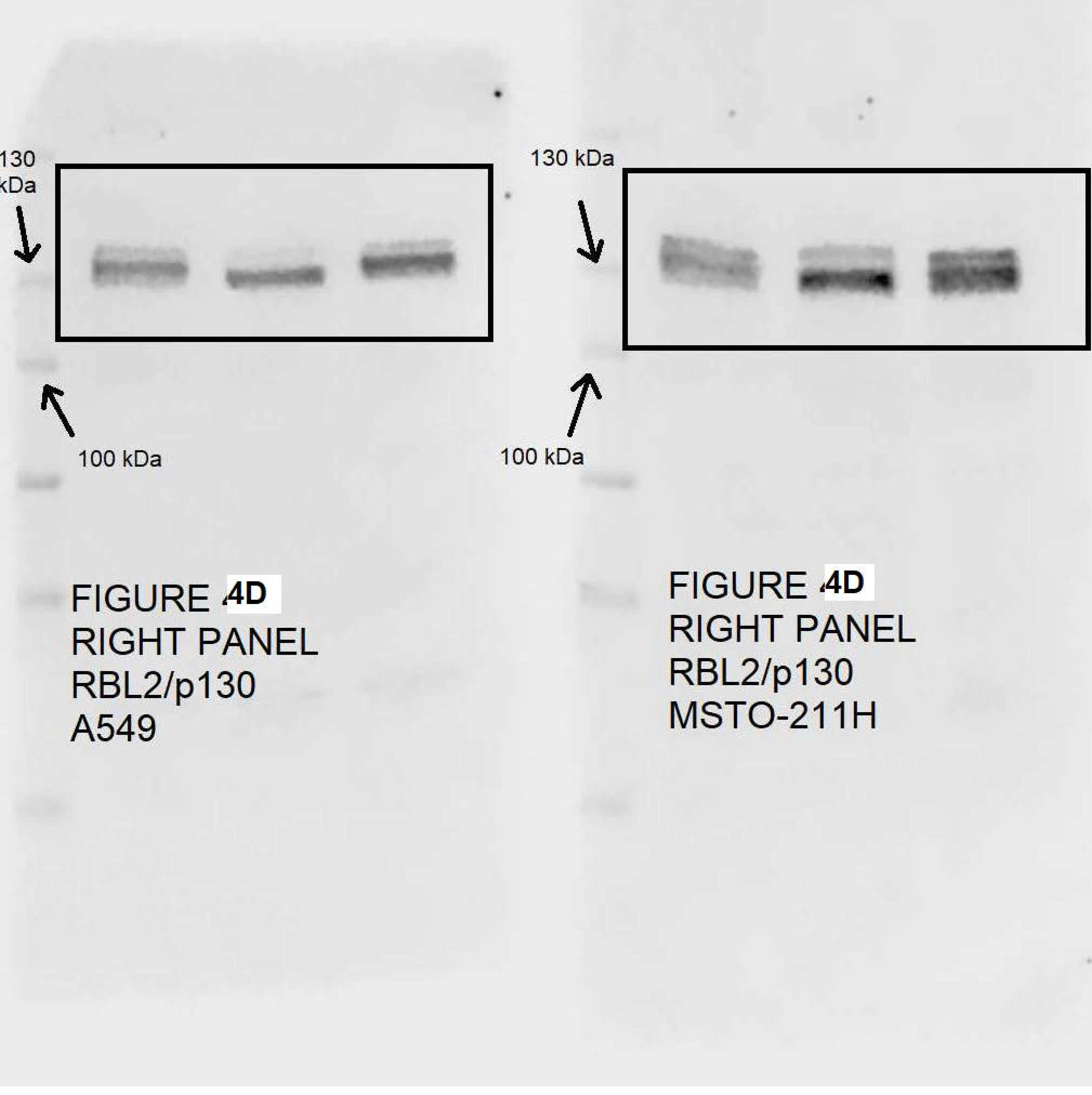

FIGURE 4D
RIGTH PANEL
BETA-ACTIN
A549

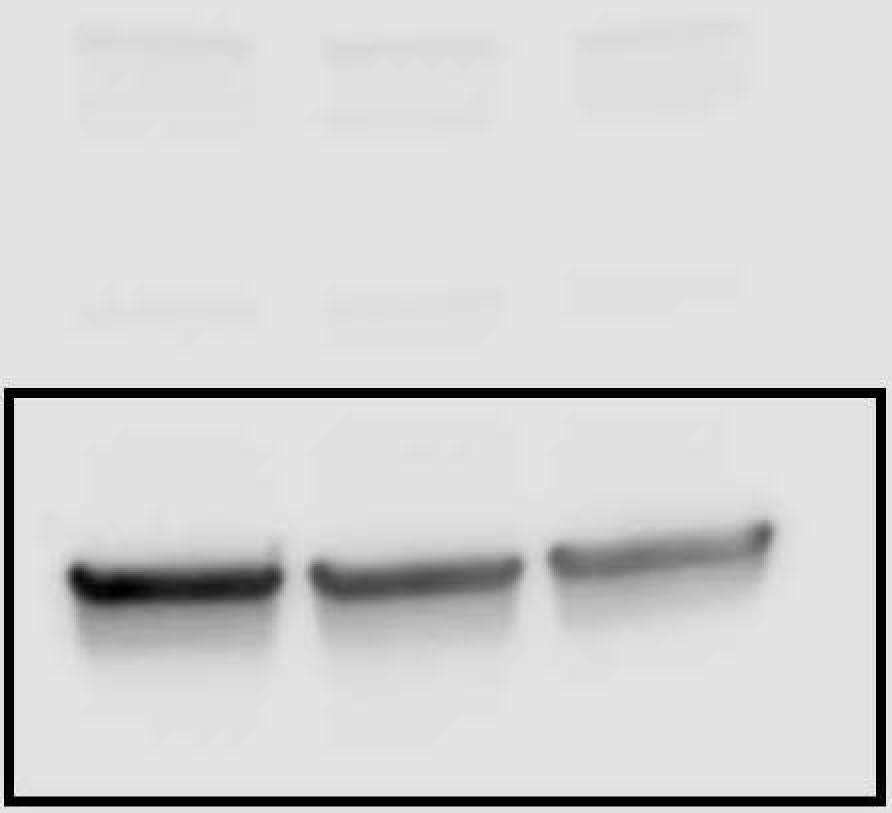

FIGURE 4D
RIGHT PANEL
BETA-ACTIN
MSTO-211H

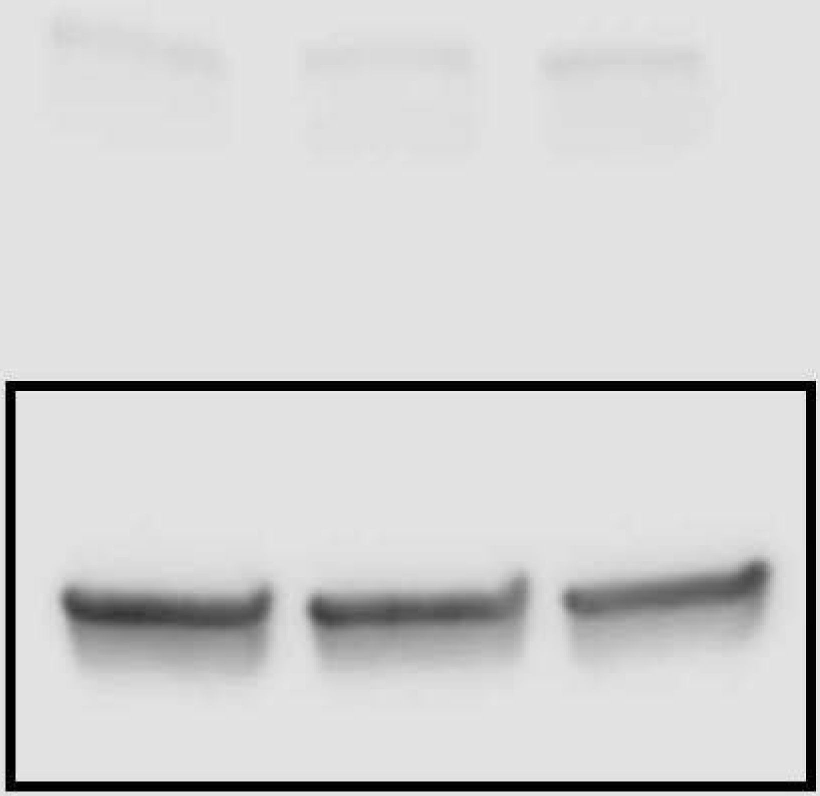

FIGURE 4D
RIGHT PANEL
A549
STANDARD OF MM OF
BETA-ACTIN

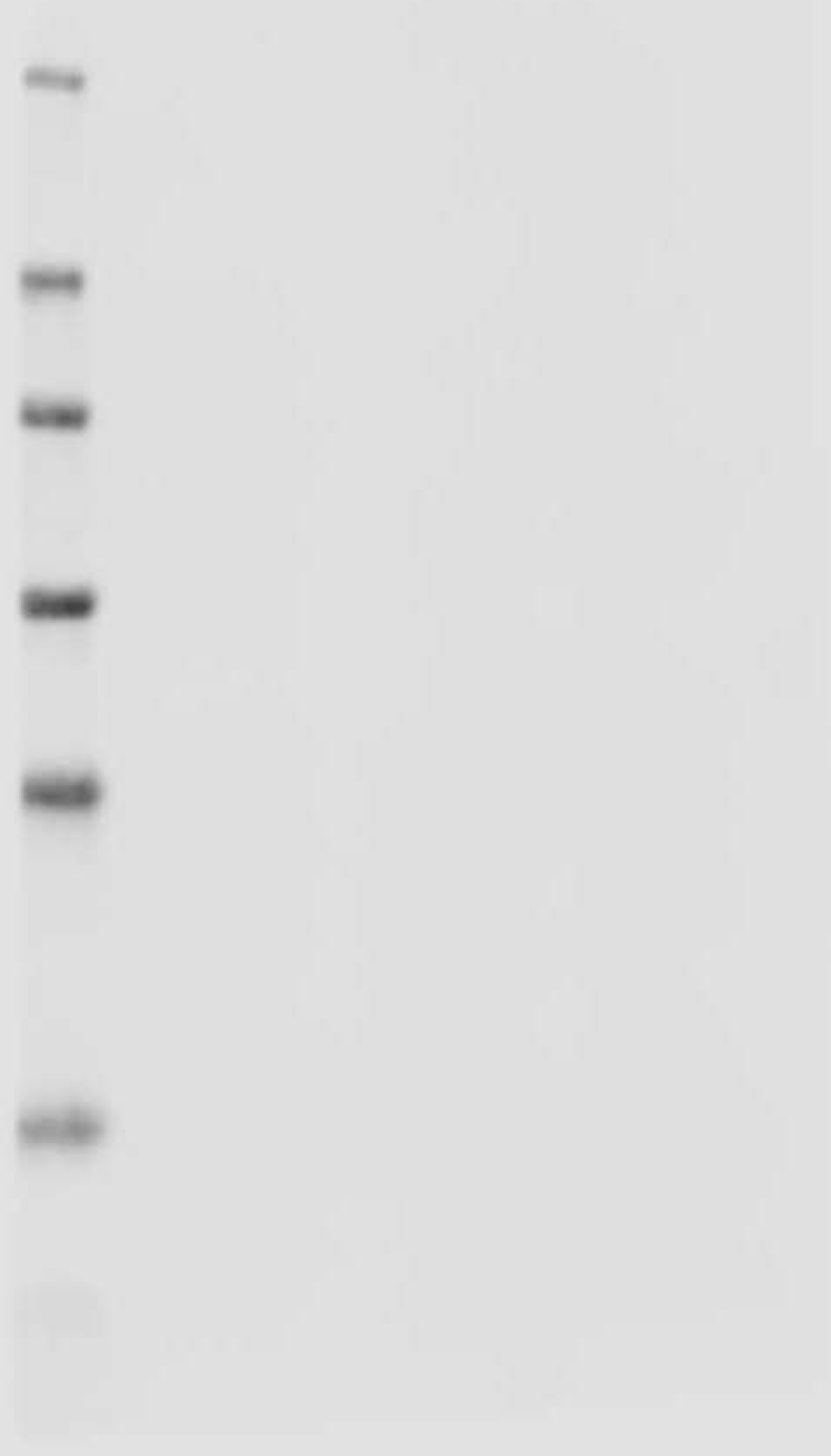

FIGURE 4D
RIGHT PANEL
MSTO-211H
STANDARD OF MM OF
BETA-ACTIN

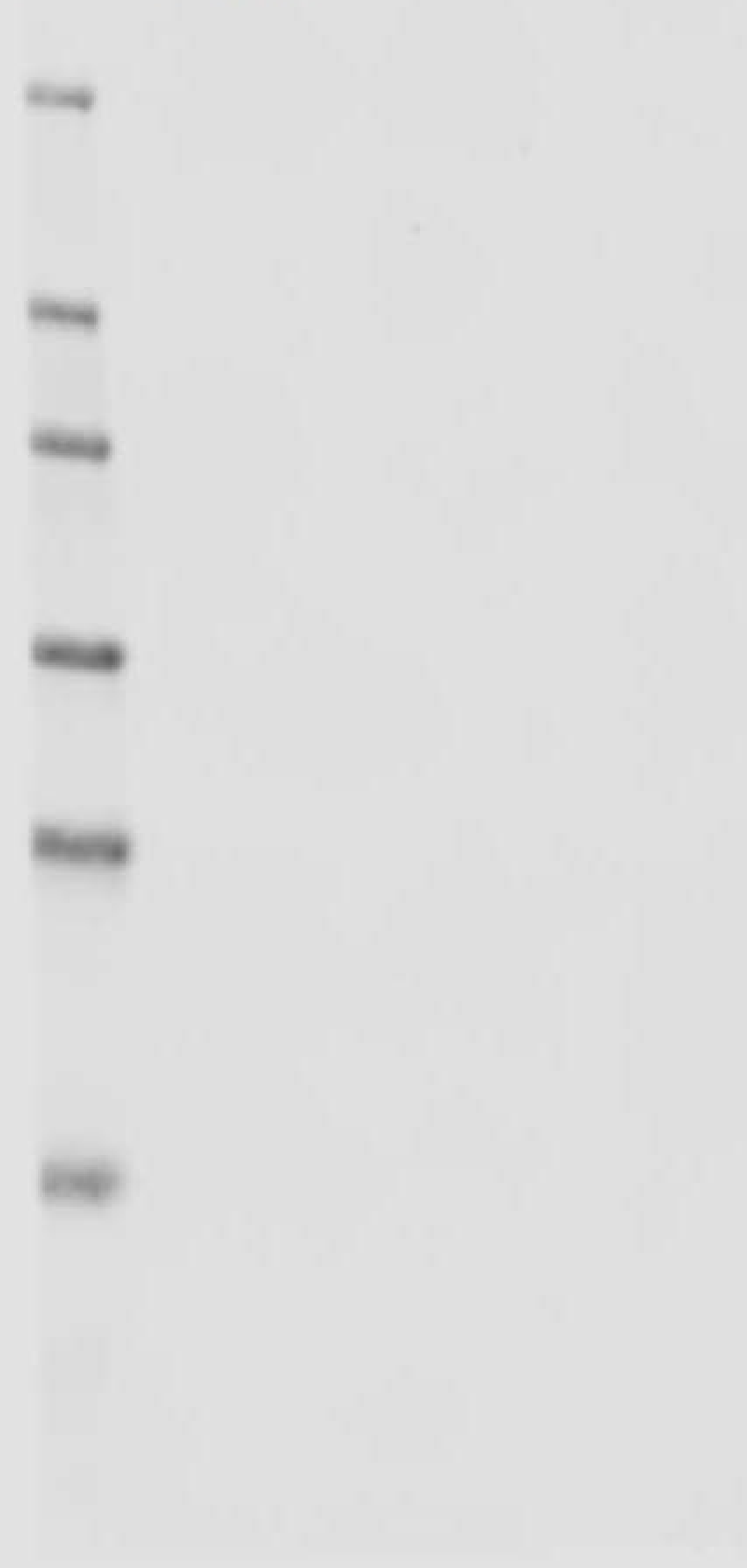

FIGURE 4D
RIGHT PANEL
MERGE OF BETA-ACTIN AND THE STANDARD OF MOLECULAR MASS

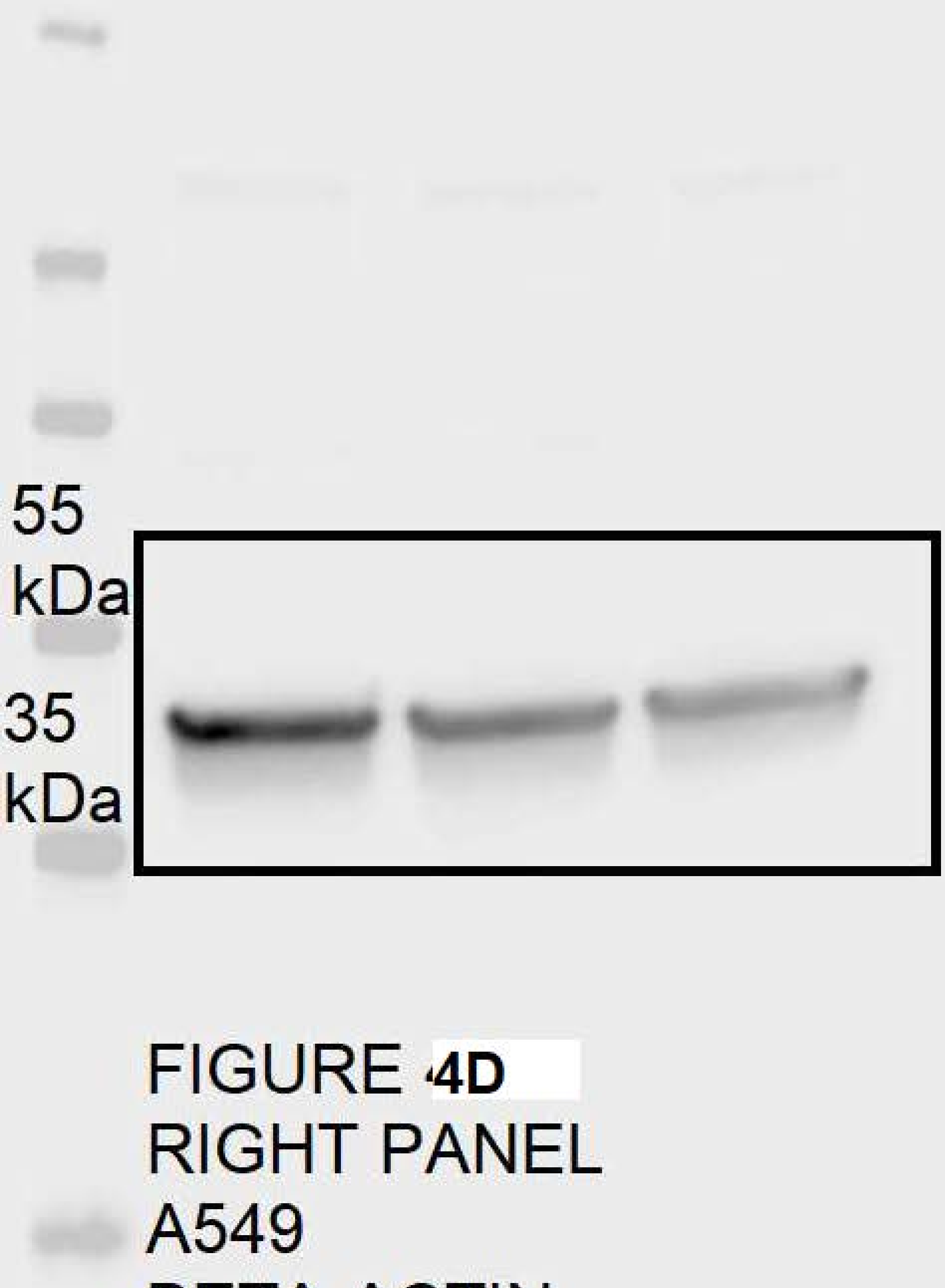

FIGURE 4D
RIGHT PANEL
A549
BETA-ACTIN

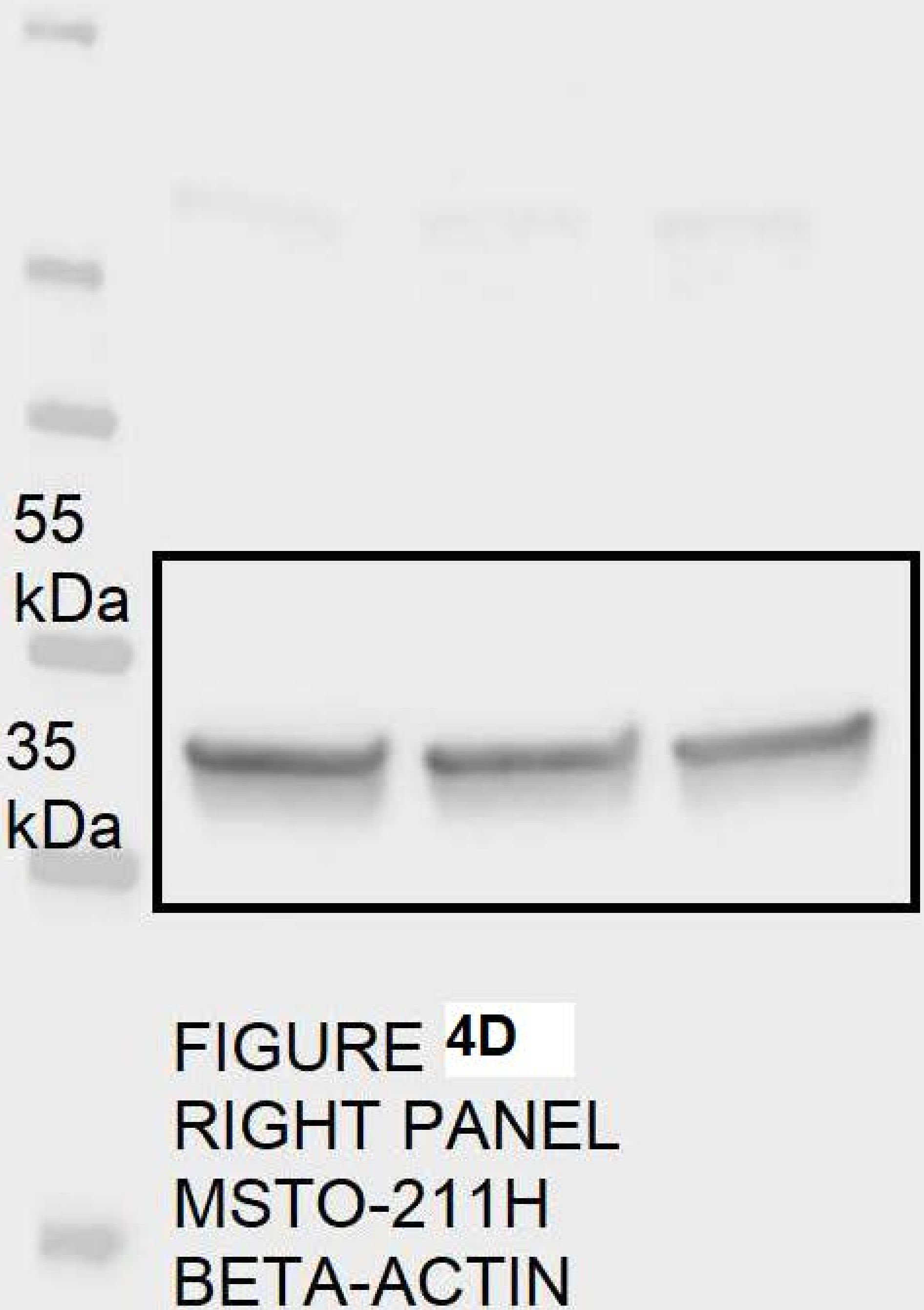

FIGURE 4D
RIGHT PANEL
MSTO-211H
BETA-ACTIN

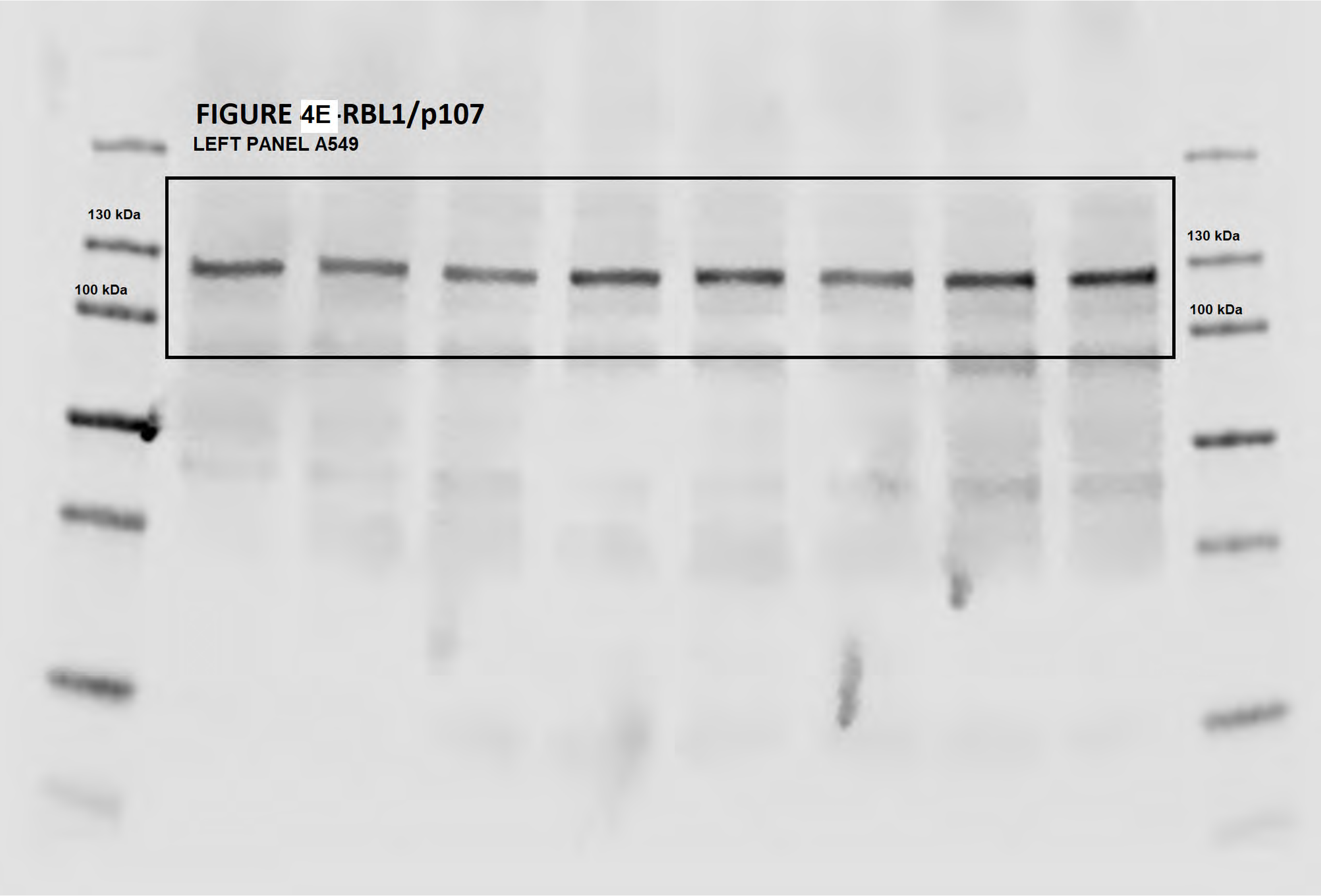
FIGURE 4E RBL1/p107
LEFT PANEL A549
130 kDa
100 kDa
130 kDa
100 kDa

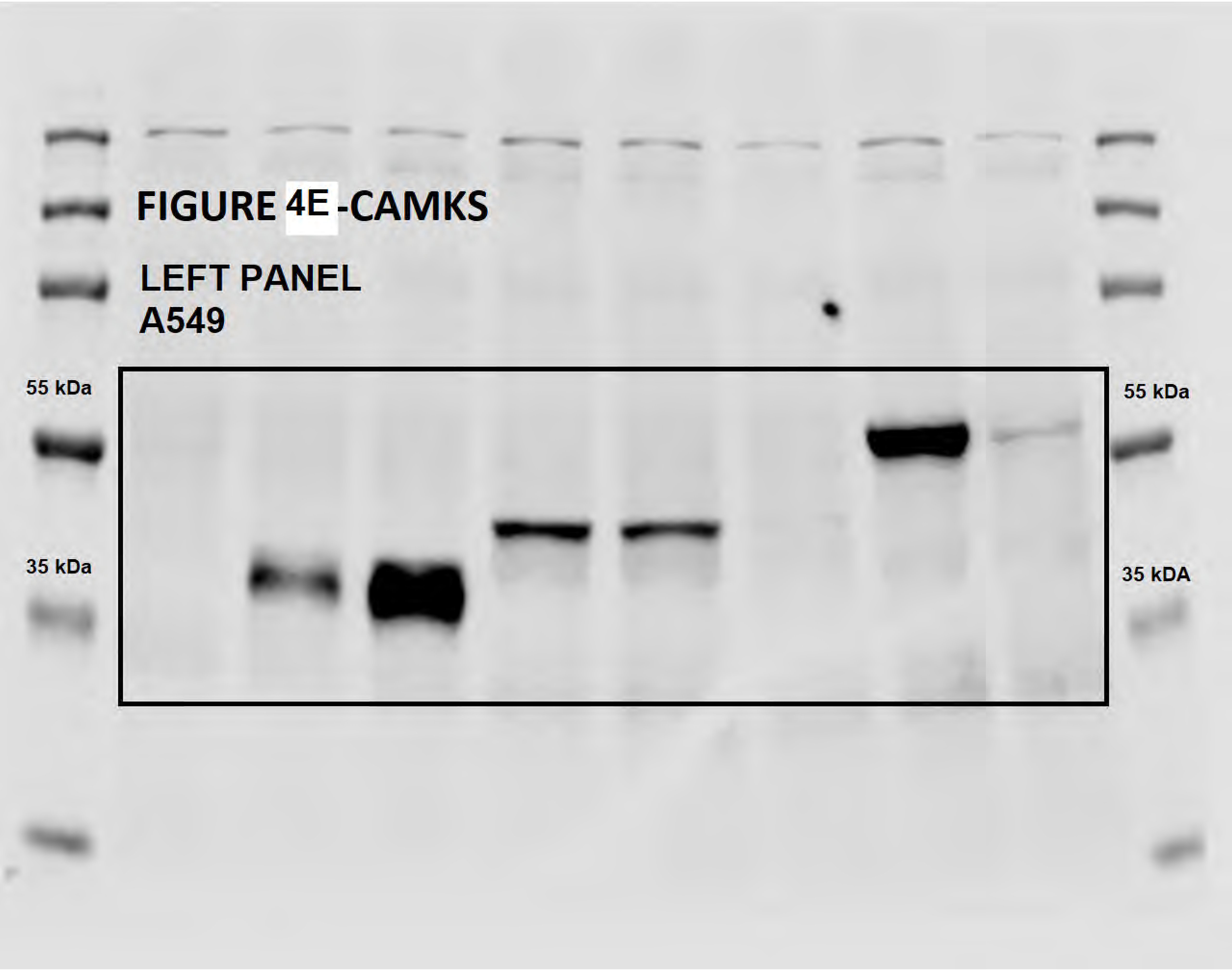
FIGURE 4E -CAMKS
LEFT PANEL
A549
55 kDa
35 kDa
55 kDa
35 kDA

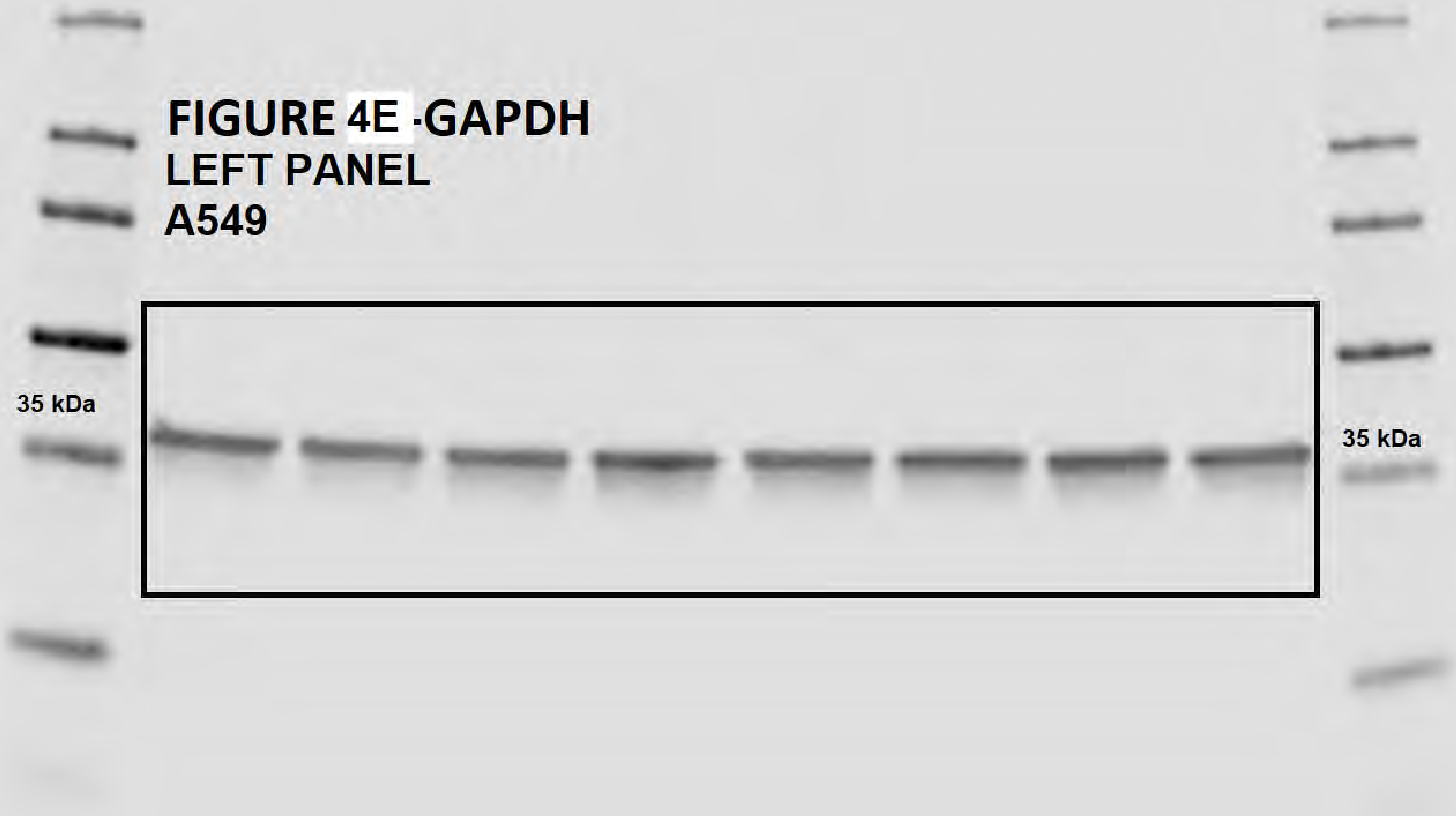
FIGURE 4E -GAPDH
LEFT PANEL
A549
35 kDa
35 kDa

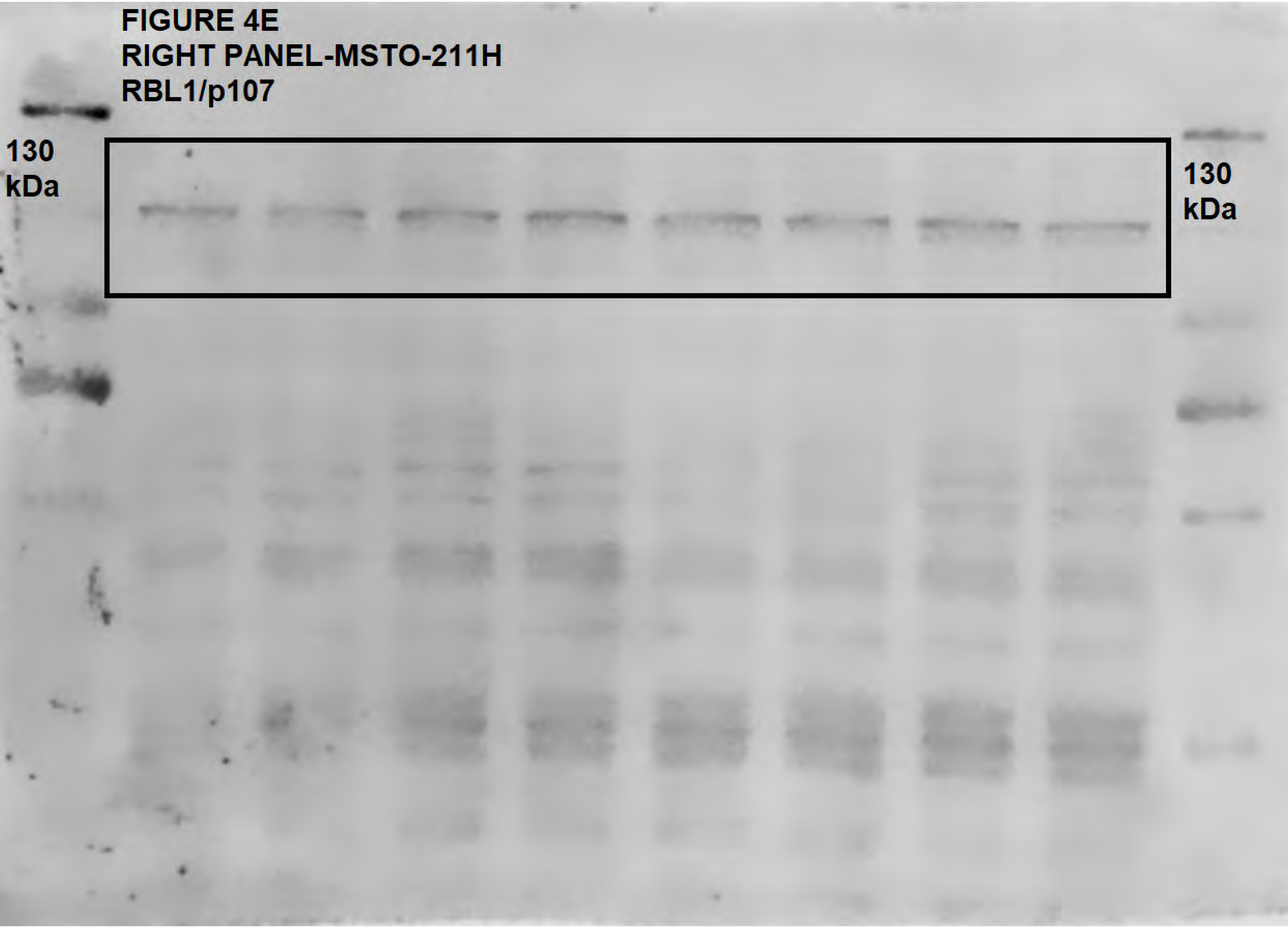
FIGURE 4E
RIGHT PANEL-MSTO-211H
RBL1/p107
130 kDa
130 kDa

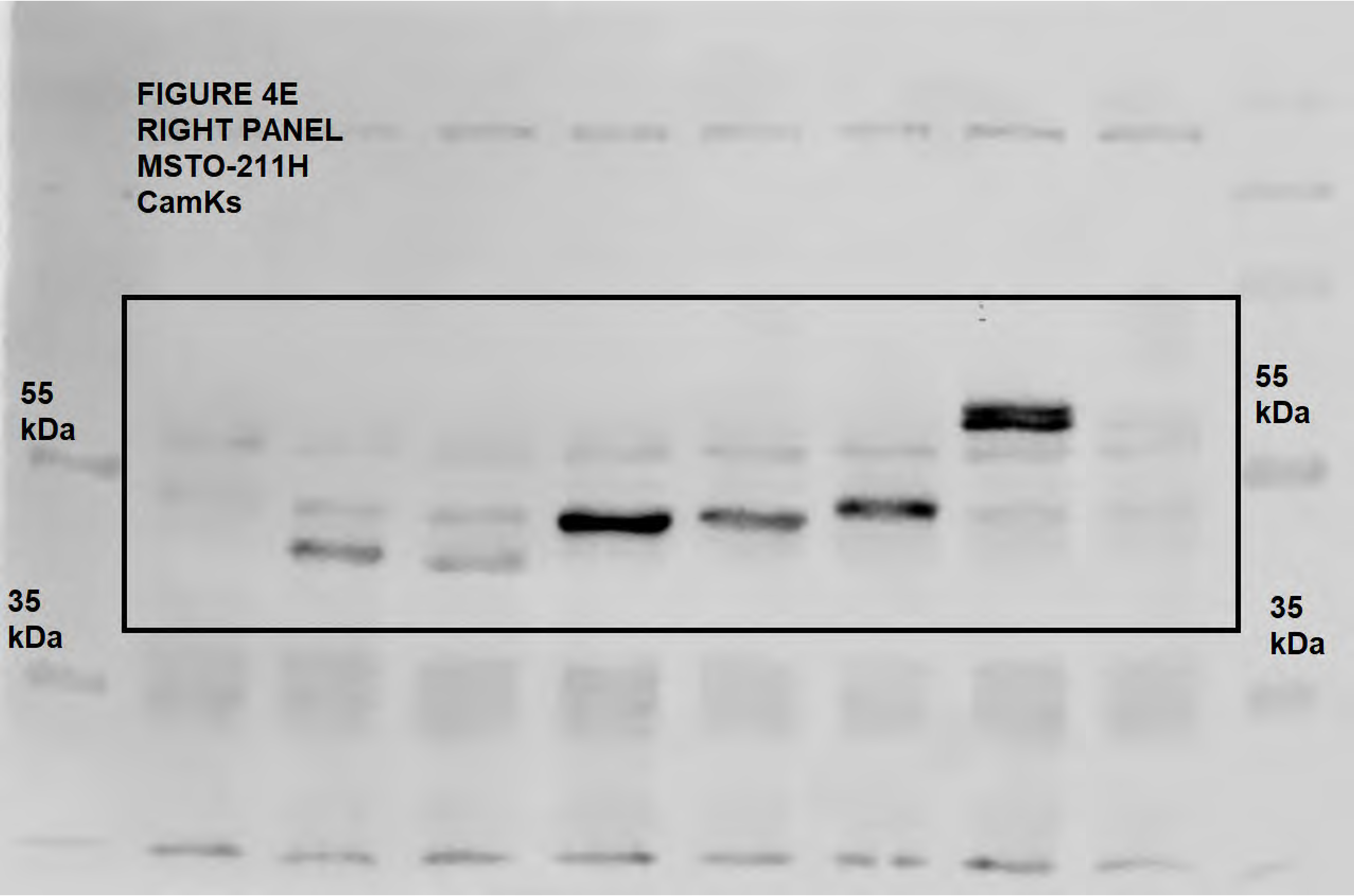
FIGURE 4E
RIGHT PANEL
MSTO-211H
CamKs
55 kDa
35 kDa
55 kDa
35 kDa

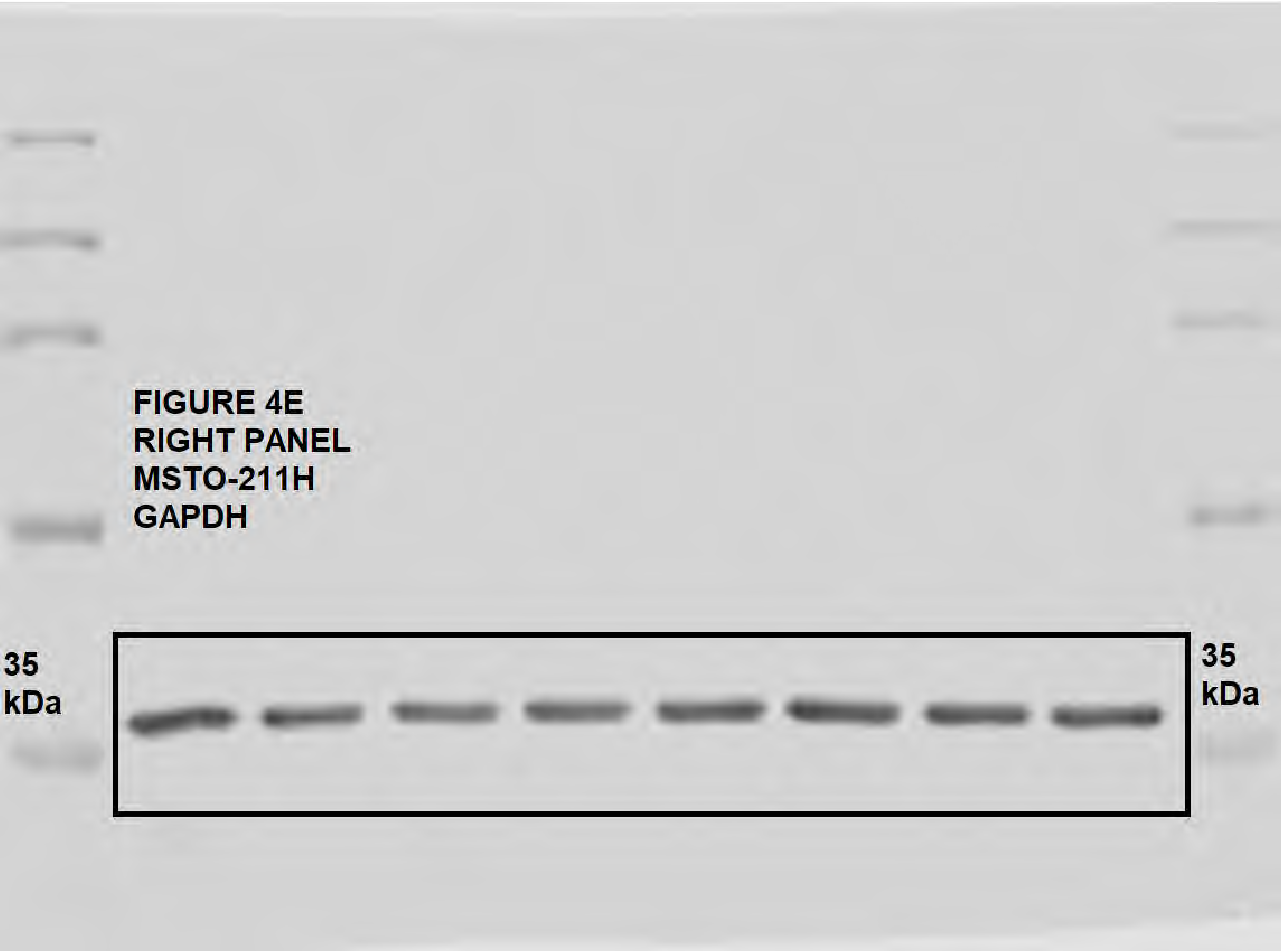
FIGURE 4E
RIGHT PANEL
MSTO-211H
GAPDH
35 kDa
35 kDa

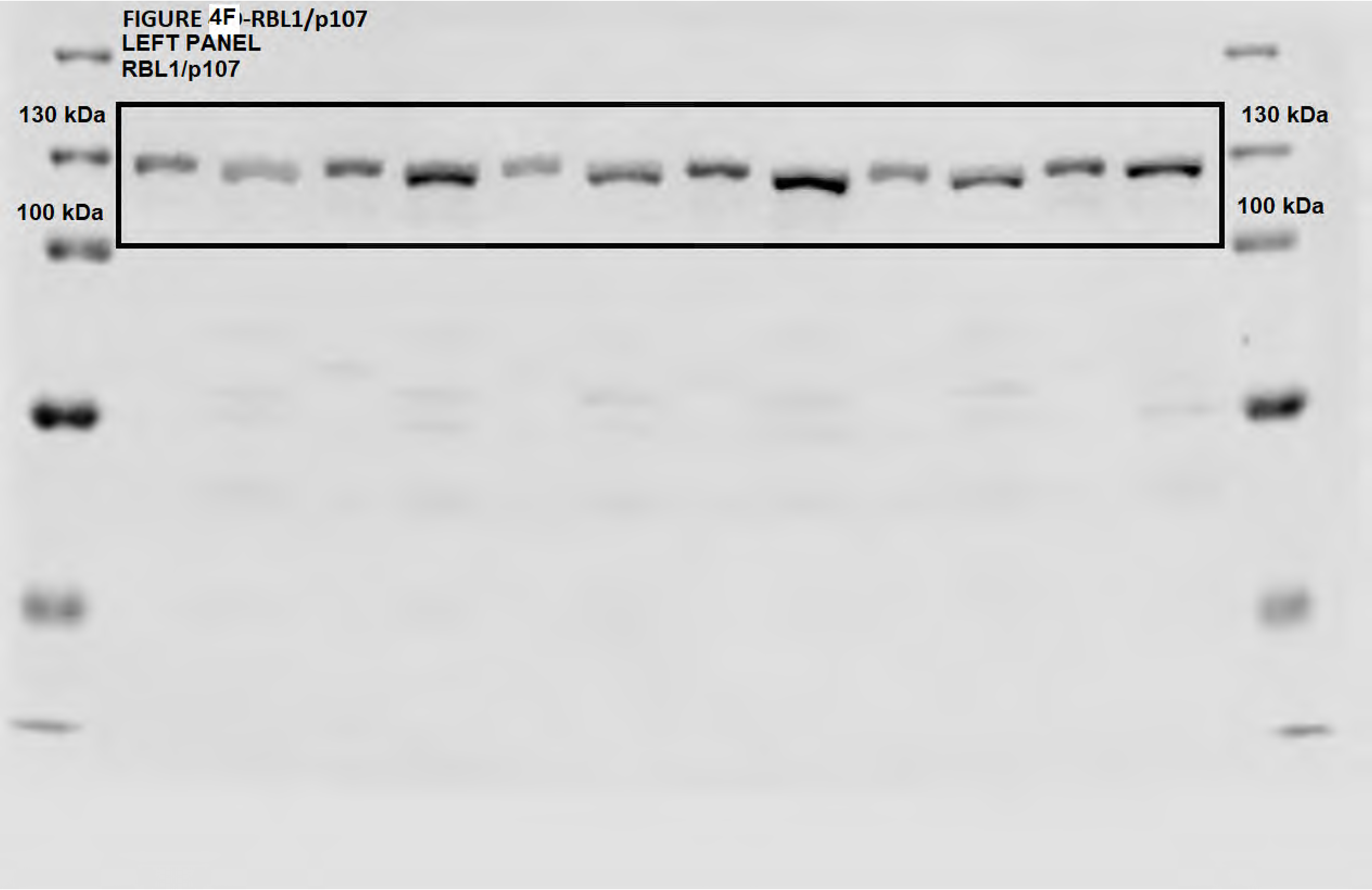
FIGURE 4F-RBL1/p107
LEFT PANEL
RBL1/p107
130 kDa
100 kDa
130 kDa
100 kDa

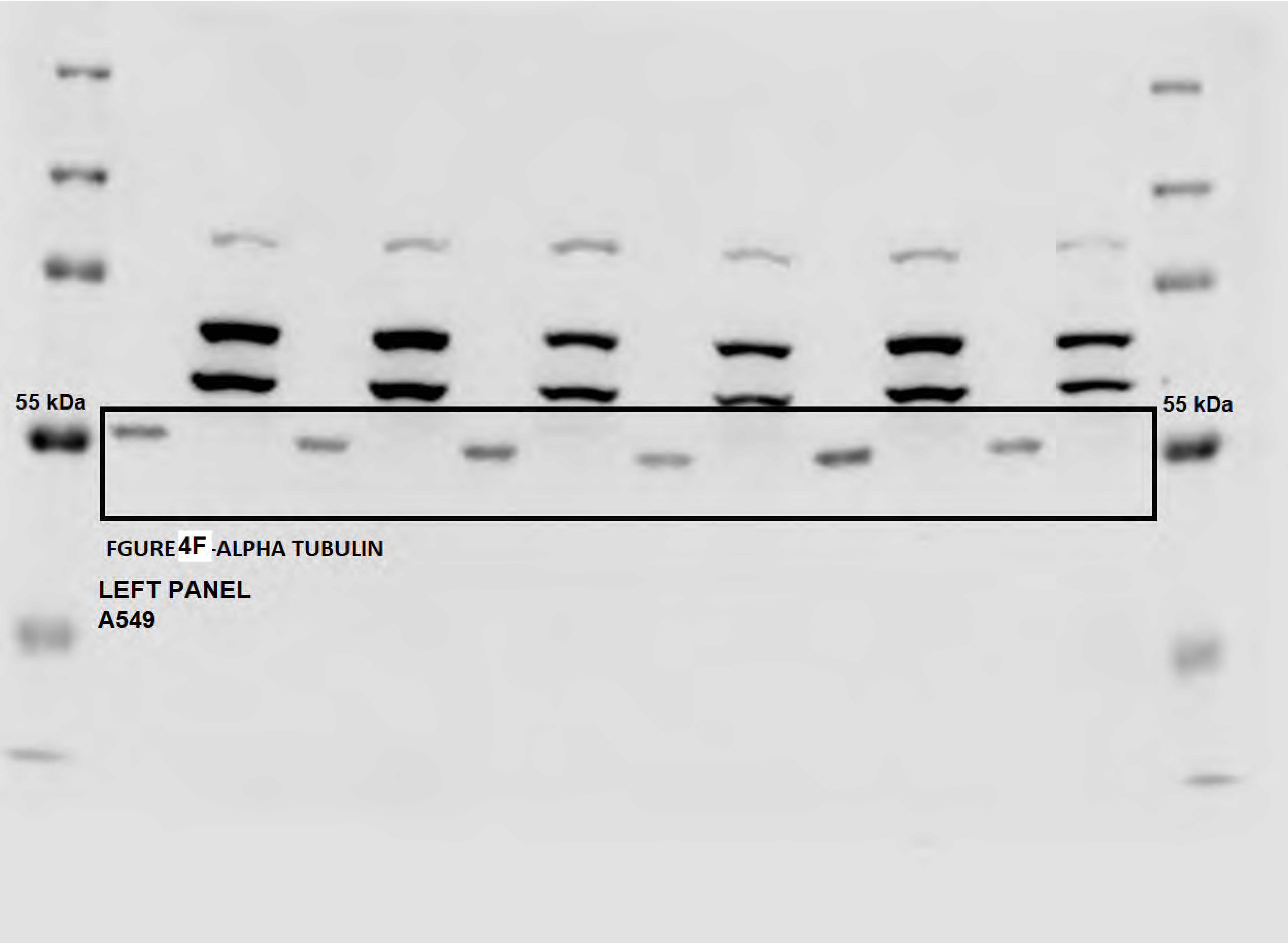
55 kDa
55 kDa
FGURE 4F -ALPHA TUBULIN
LEFT PANEL
A549

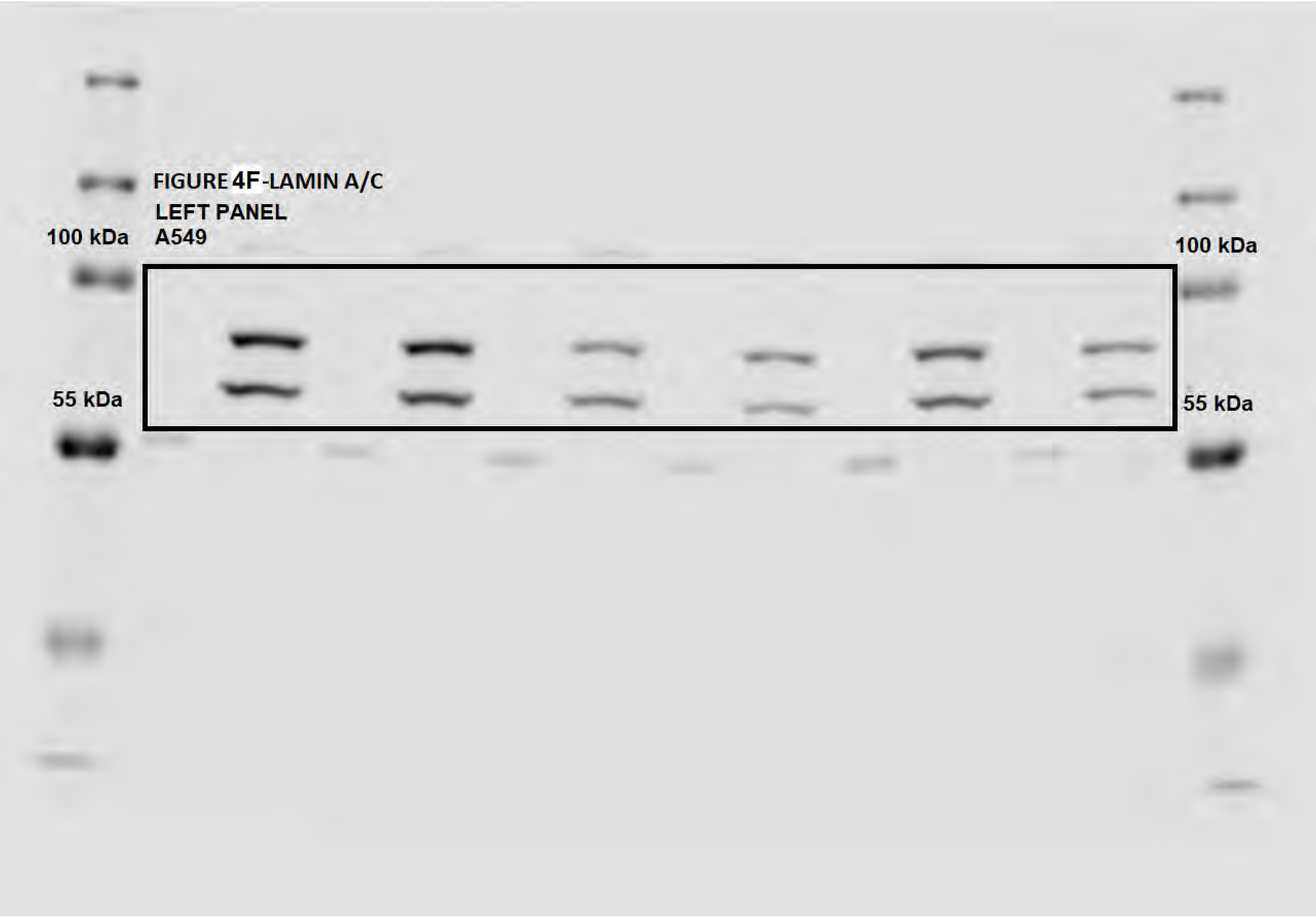
FIGURE 4F-LAMIN A/C
LEFT PANEL
A549
100 kDa
55 kDa
100 kDa
55 kDa

FIGURE 4F
RIGHT PANEL
MSTO-211H - RBL1/p107

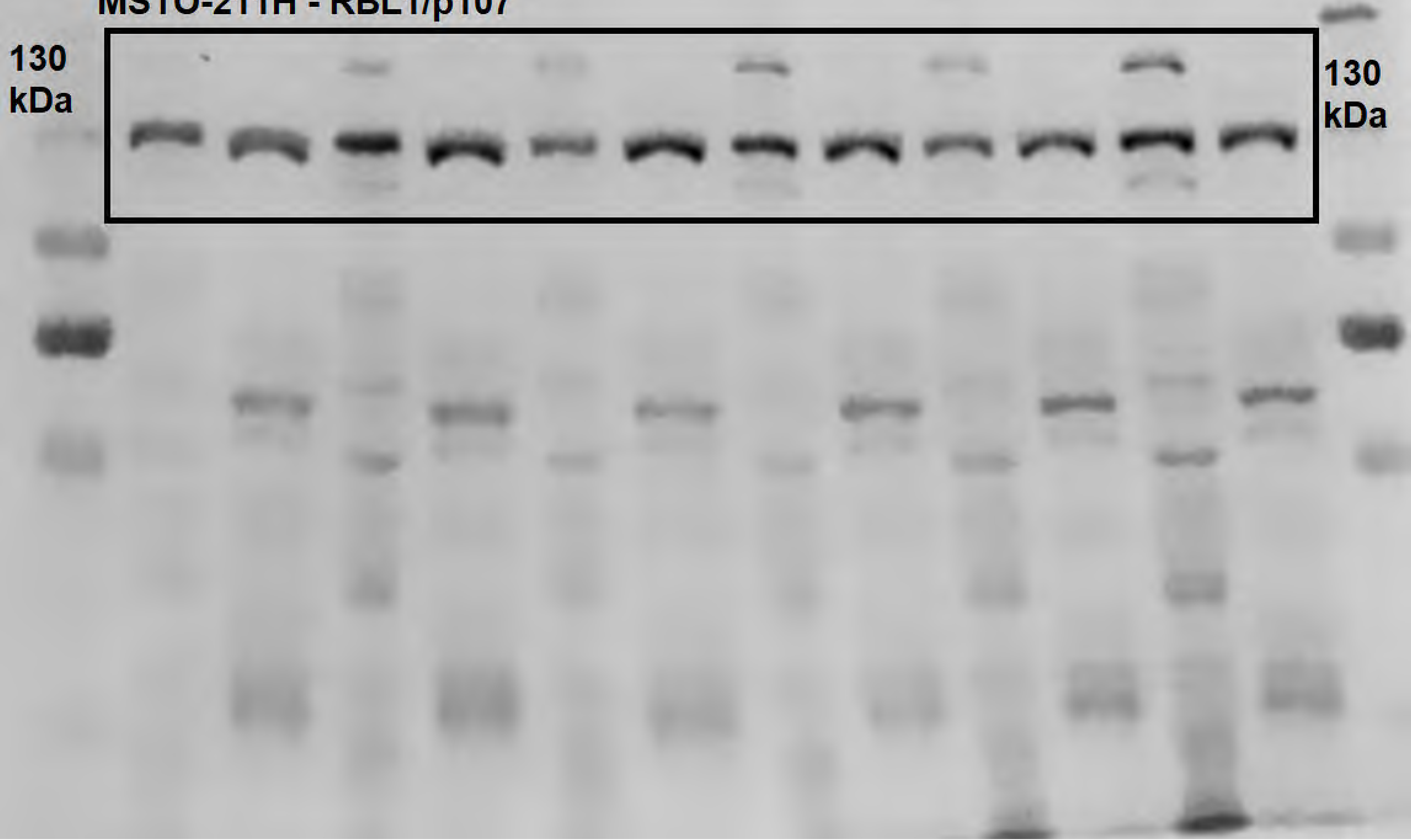

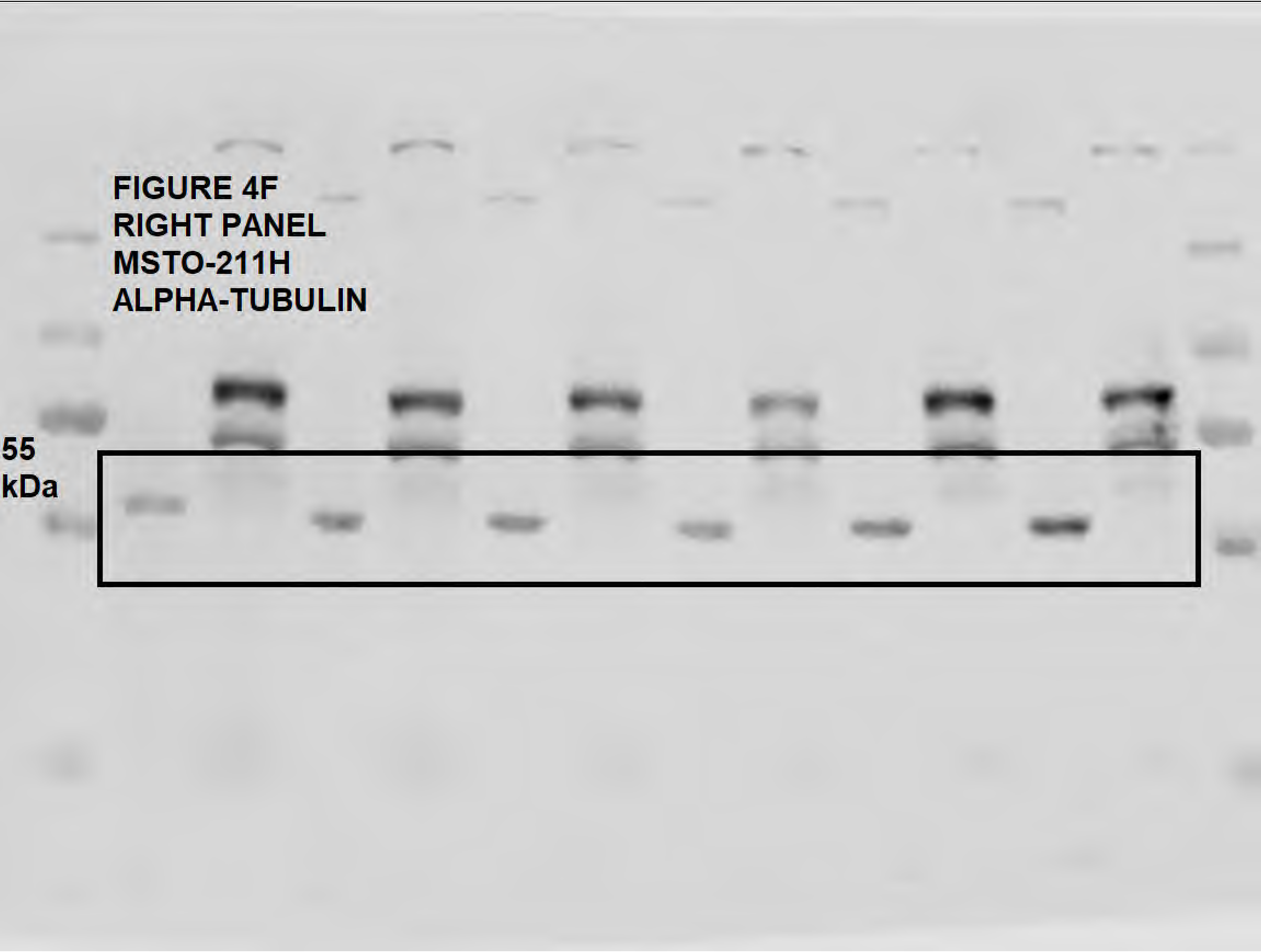
FIGURE 4F
RIGHT PANEL
MSTO-211H
ALPHA-TUBULIN
55
kDa

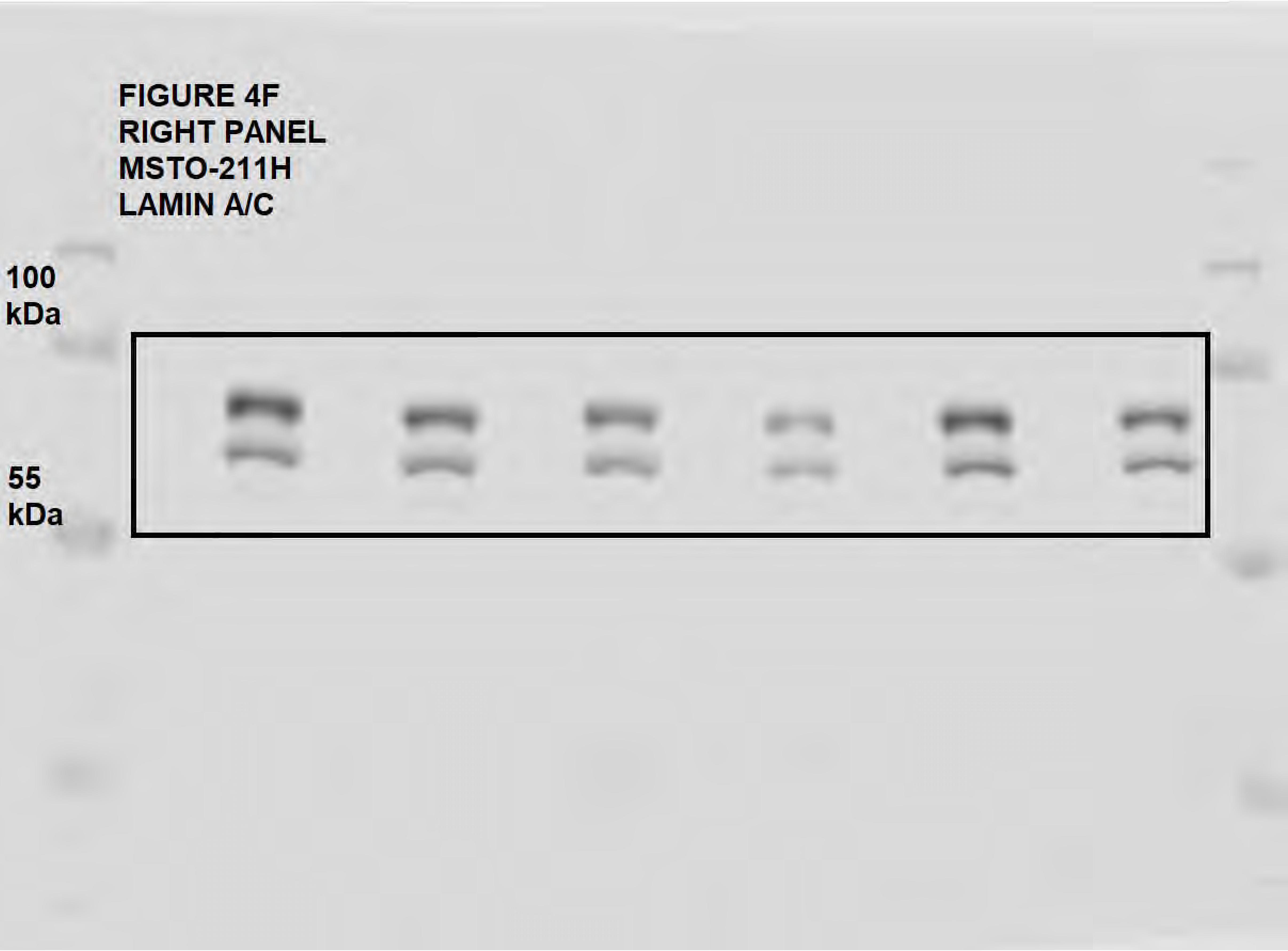
FIGURE 4F
RIGHT PANEL
MSTO-211H
LAMIN A/C
100
kDa
55
kDa

FIGURE 5B
CycD1

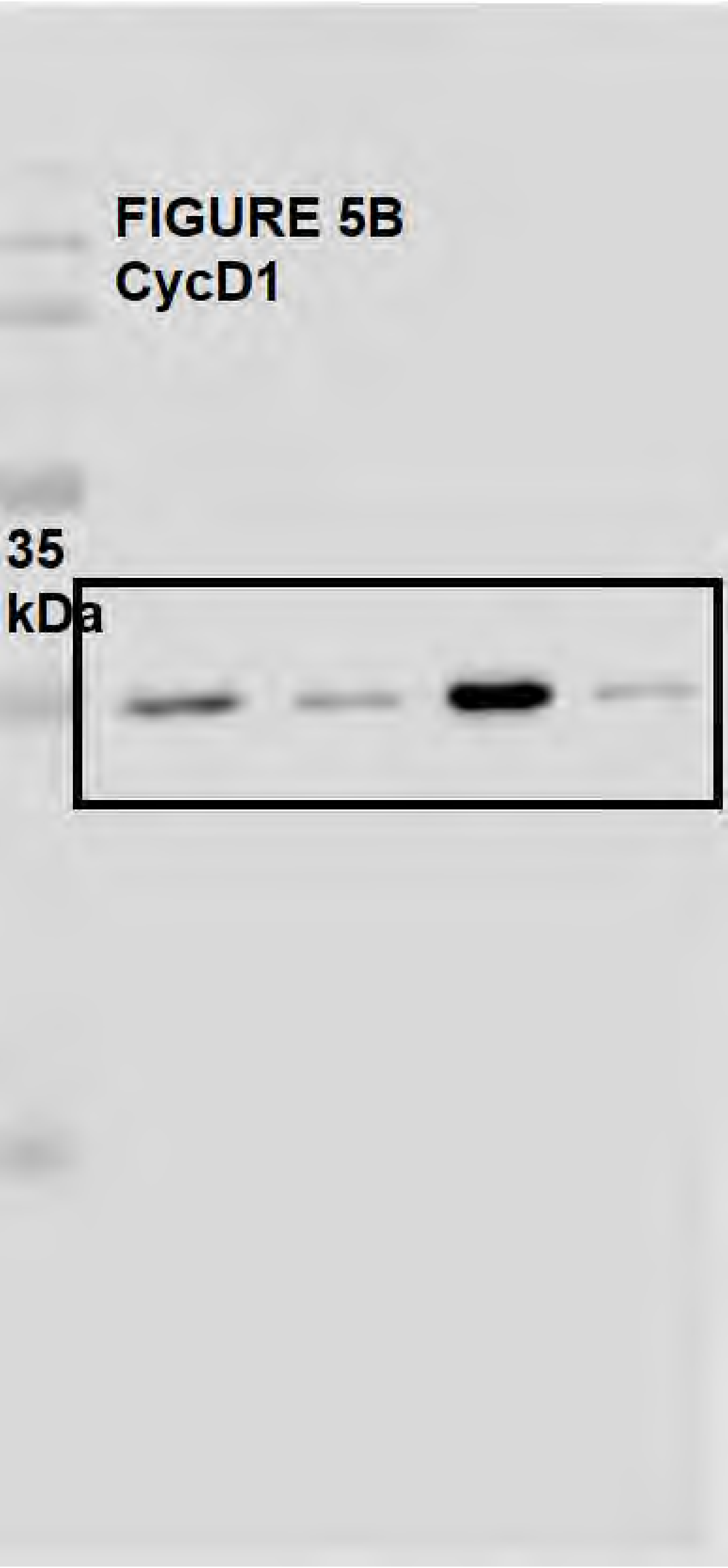

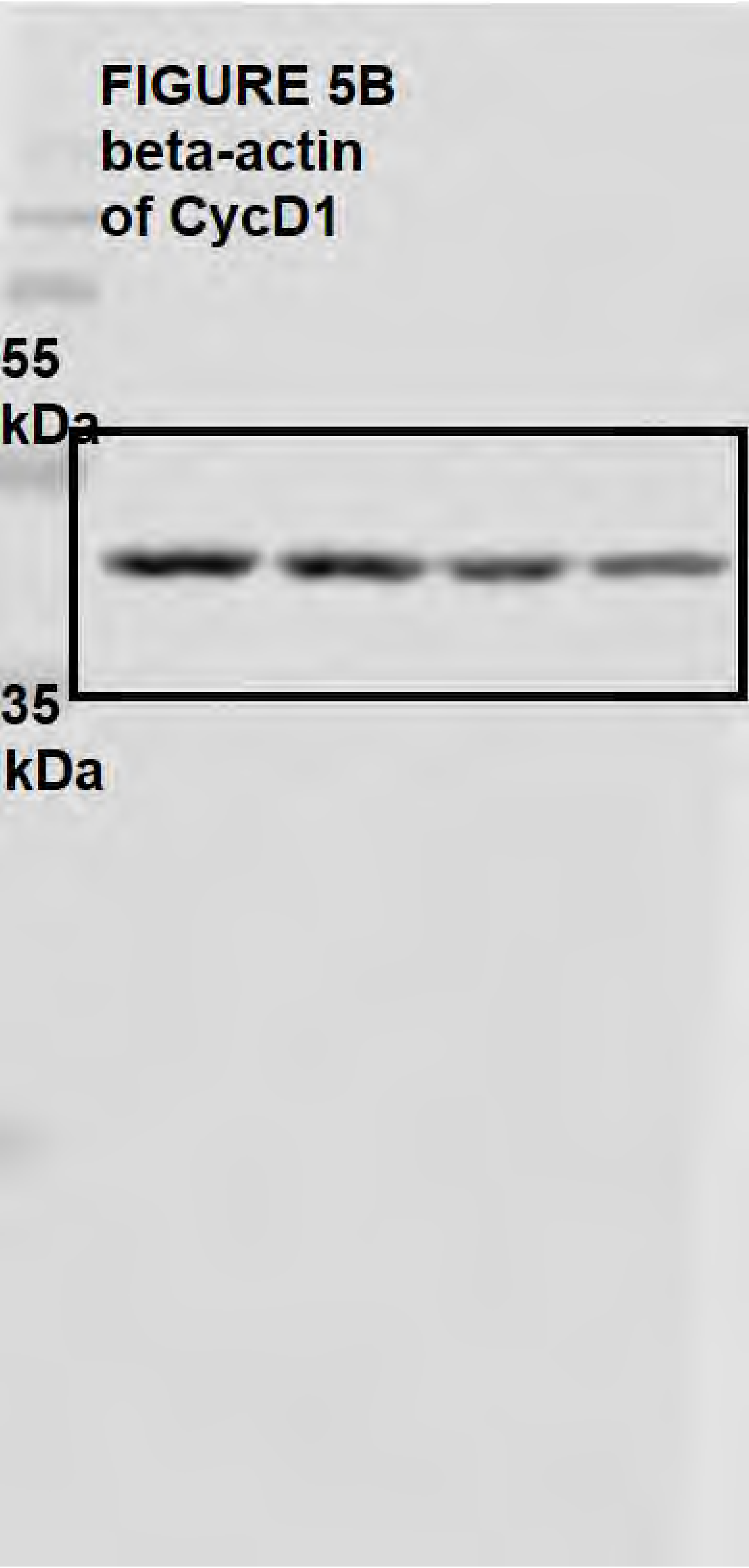

FIGURE 5B
beta-actin
of CycD1

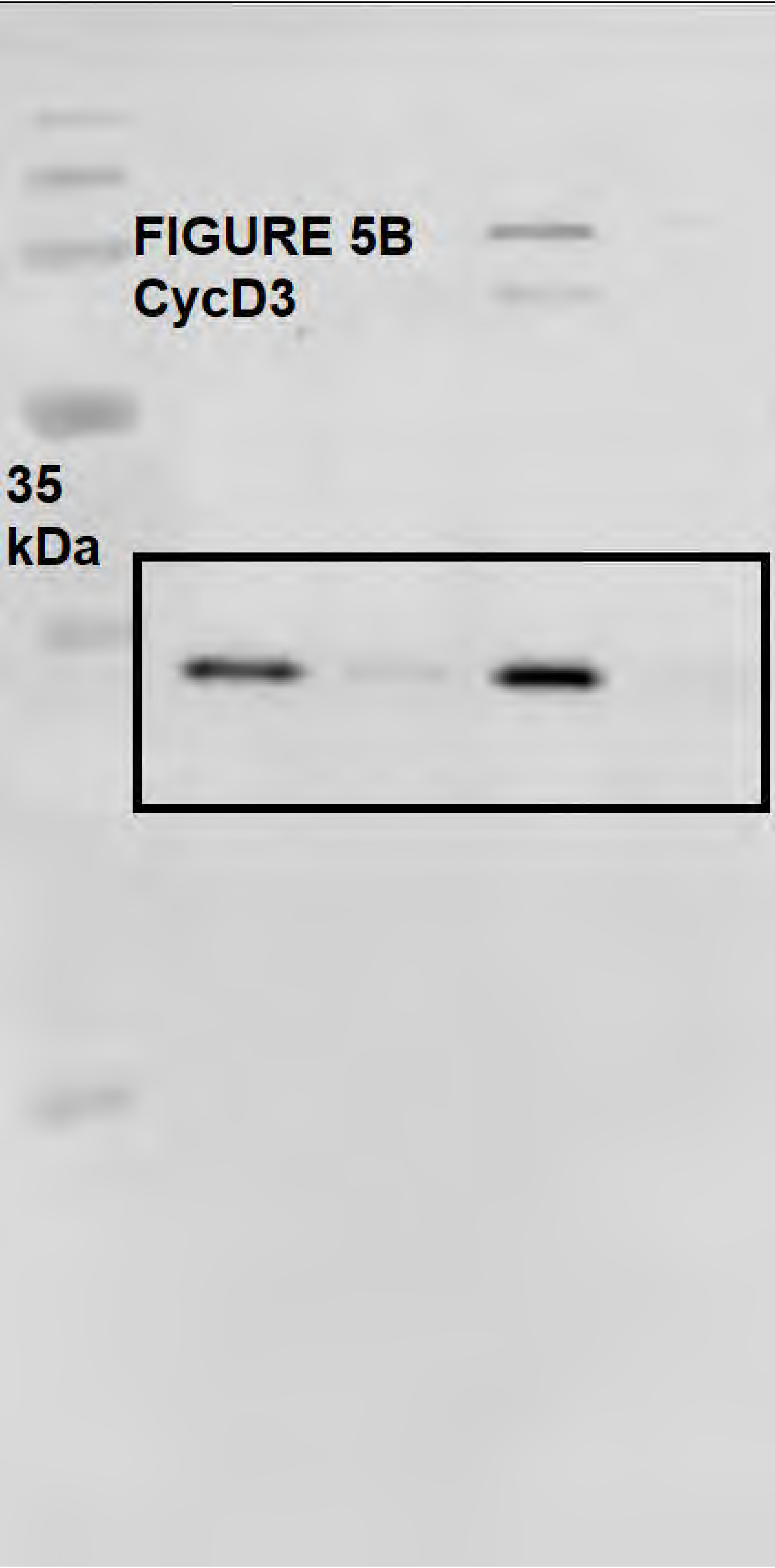

FIGURE 5B
CycD3

FIGURE 5B
beta-actin of
CycD3

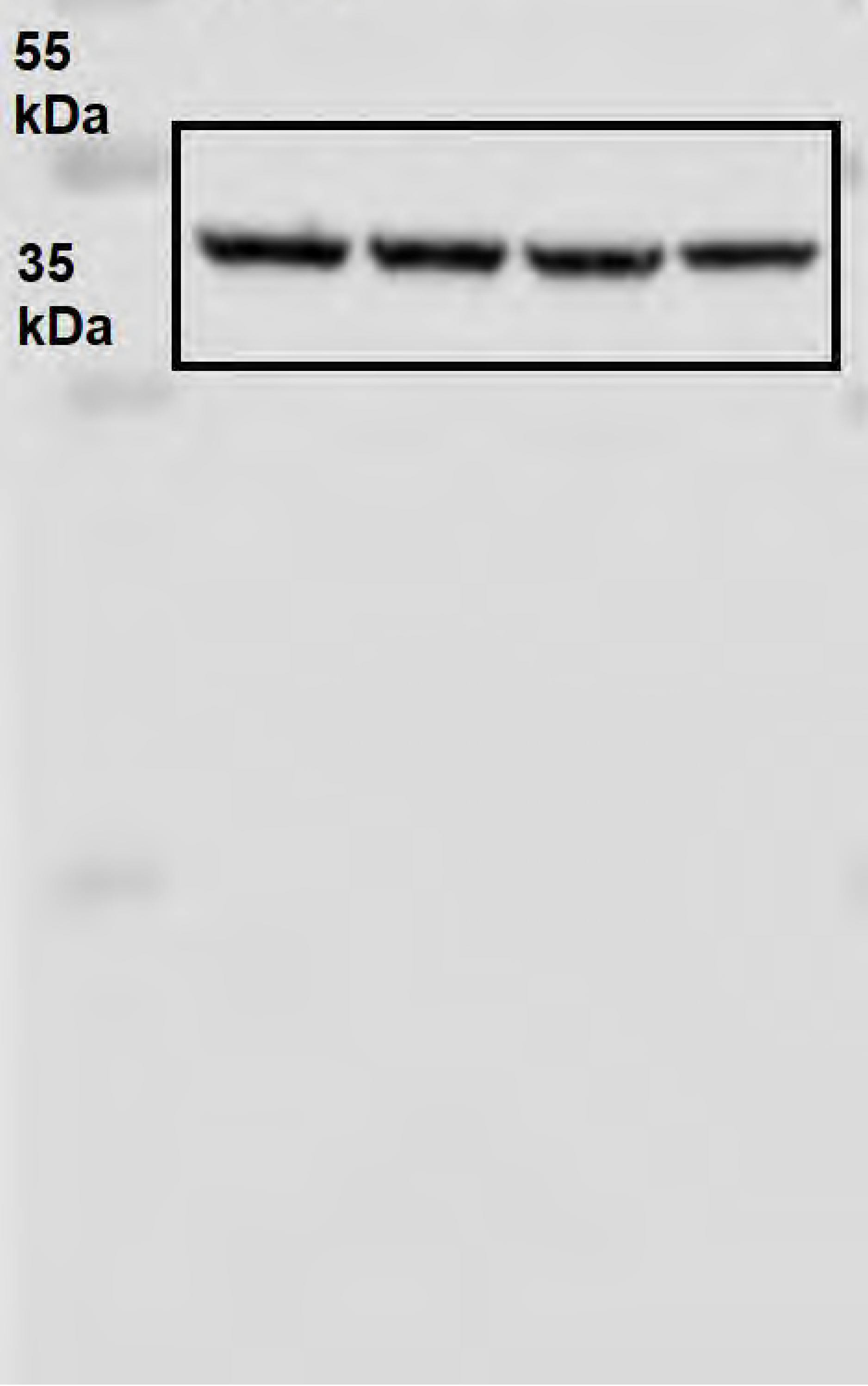

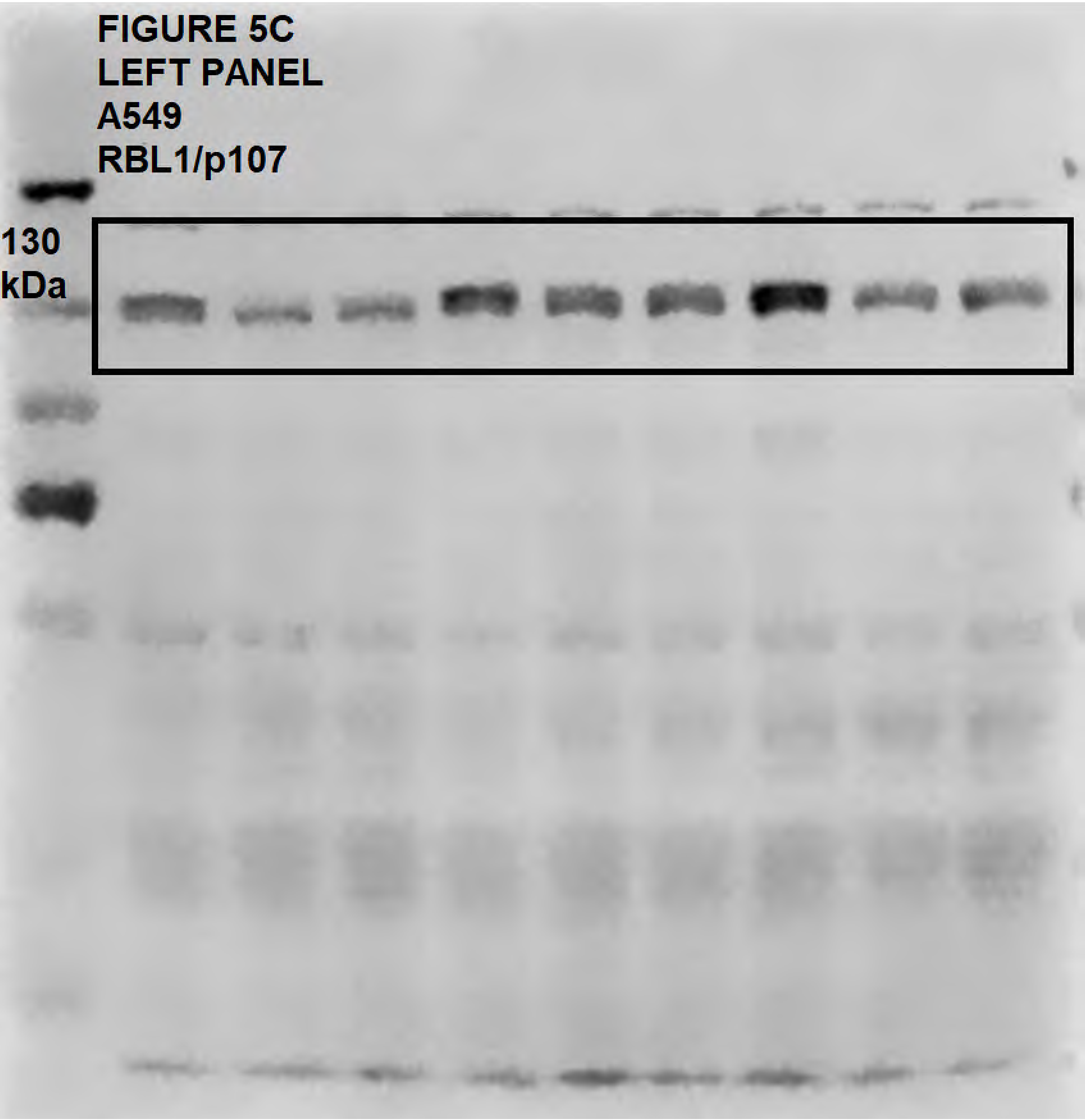
FIGURE 5C
LEFT PANEL
A549
RBL1/p107
130
kDa

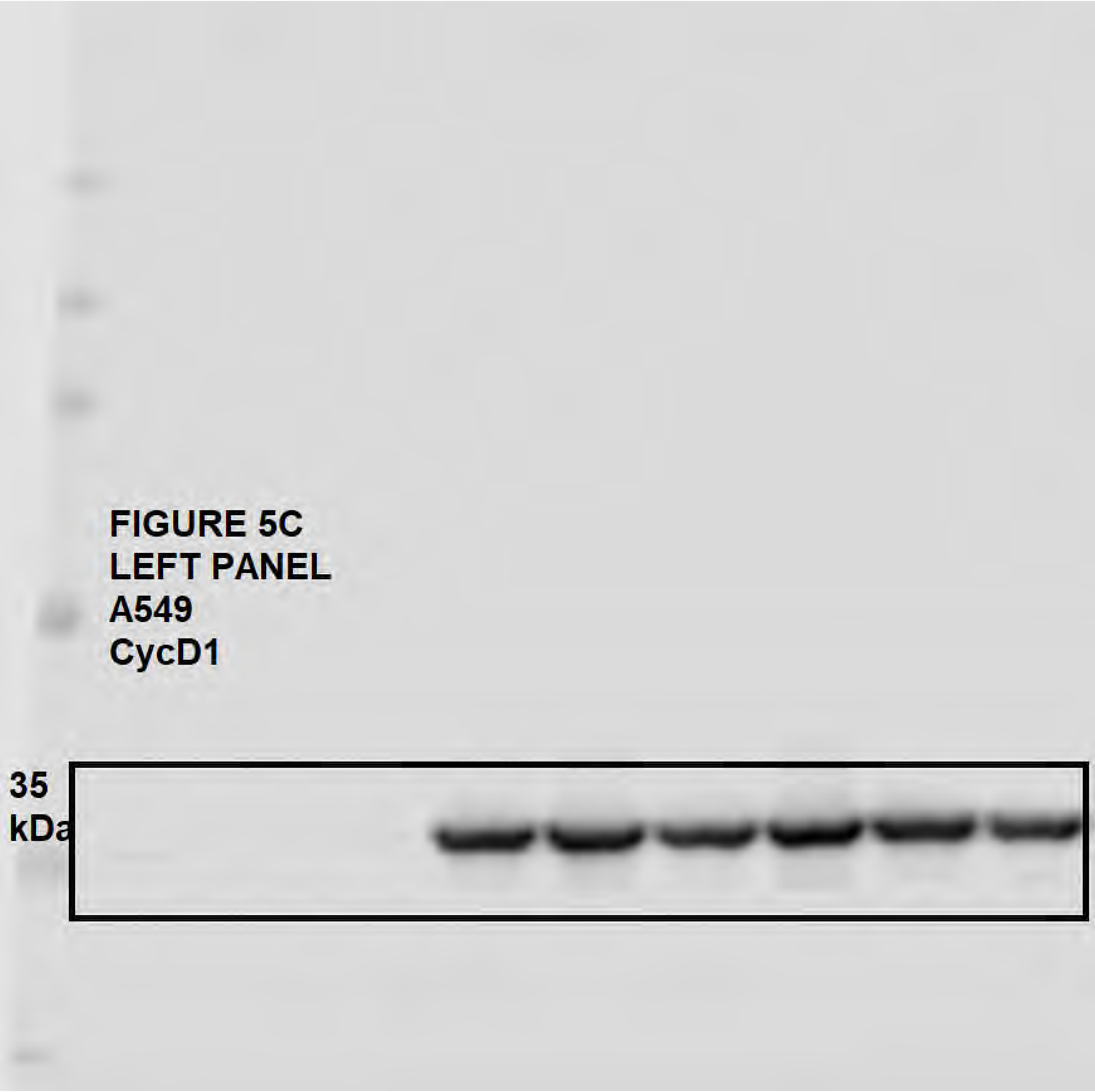
FIGURE 5C
LEFT PANEL
A549
CycD1
35
kDa

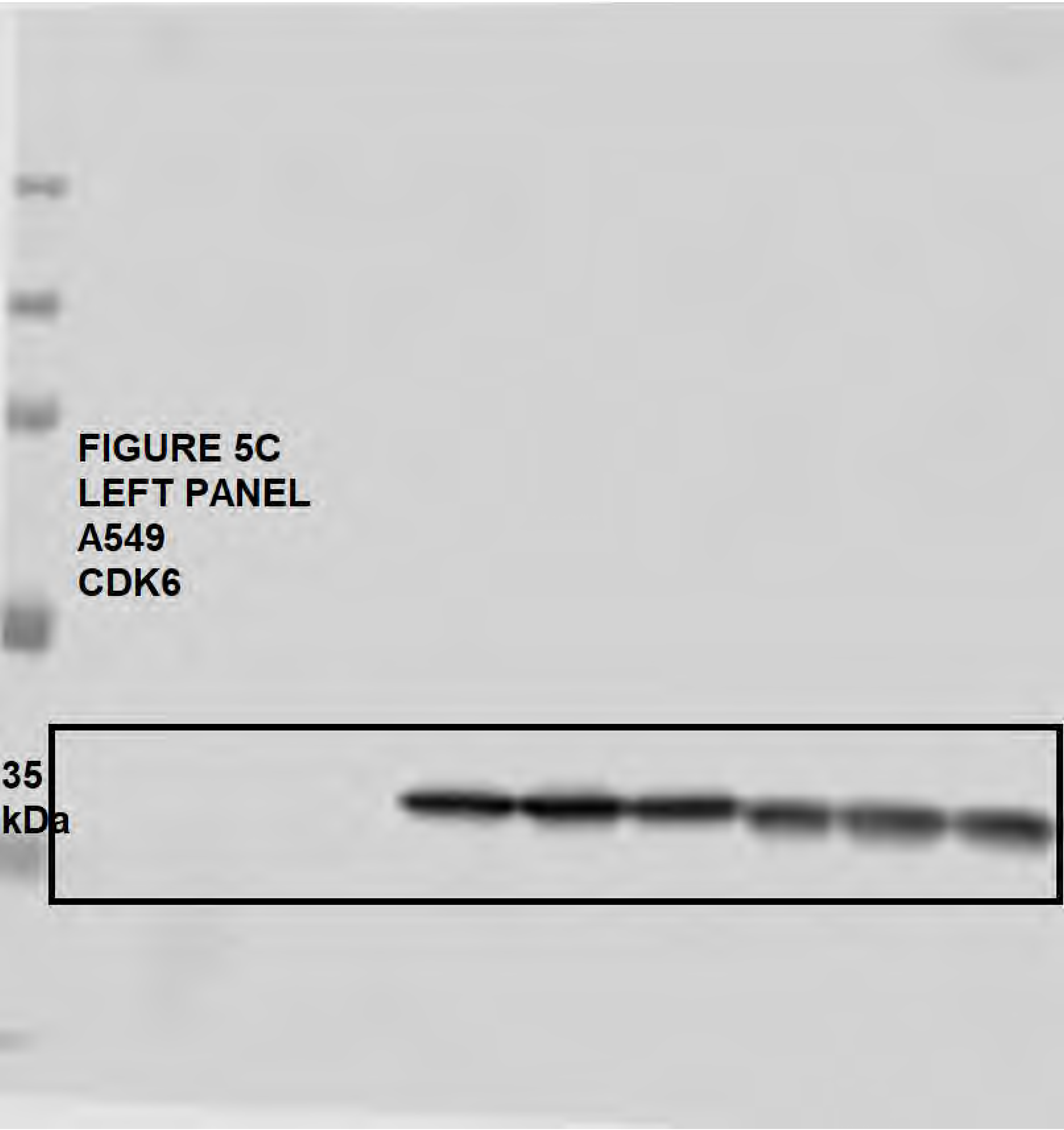
FIGURE 5C
LEFT PANEL
A549
CDK6
35
kDa

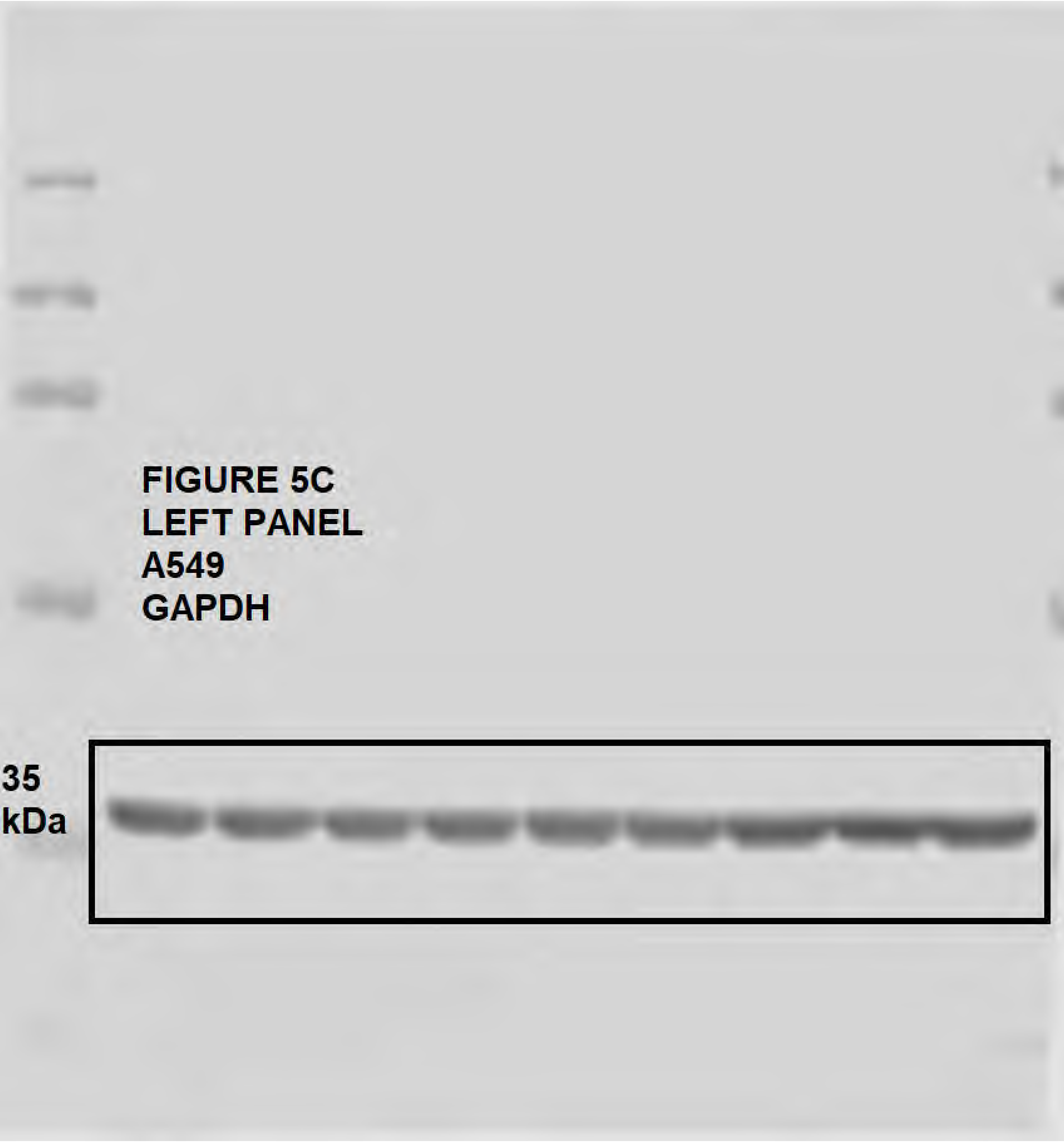
FIGURE 5C
LEFT PANEL
A549
GAPDH
35
kDa

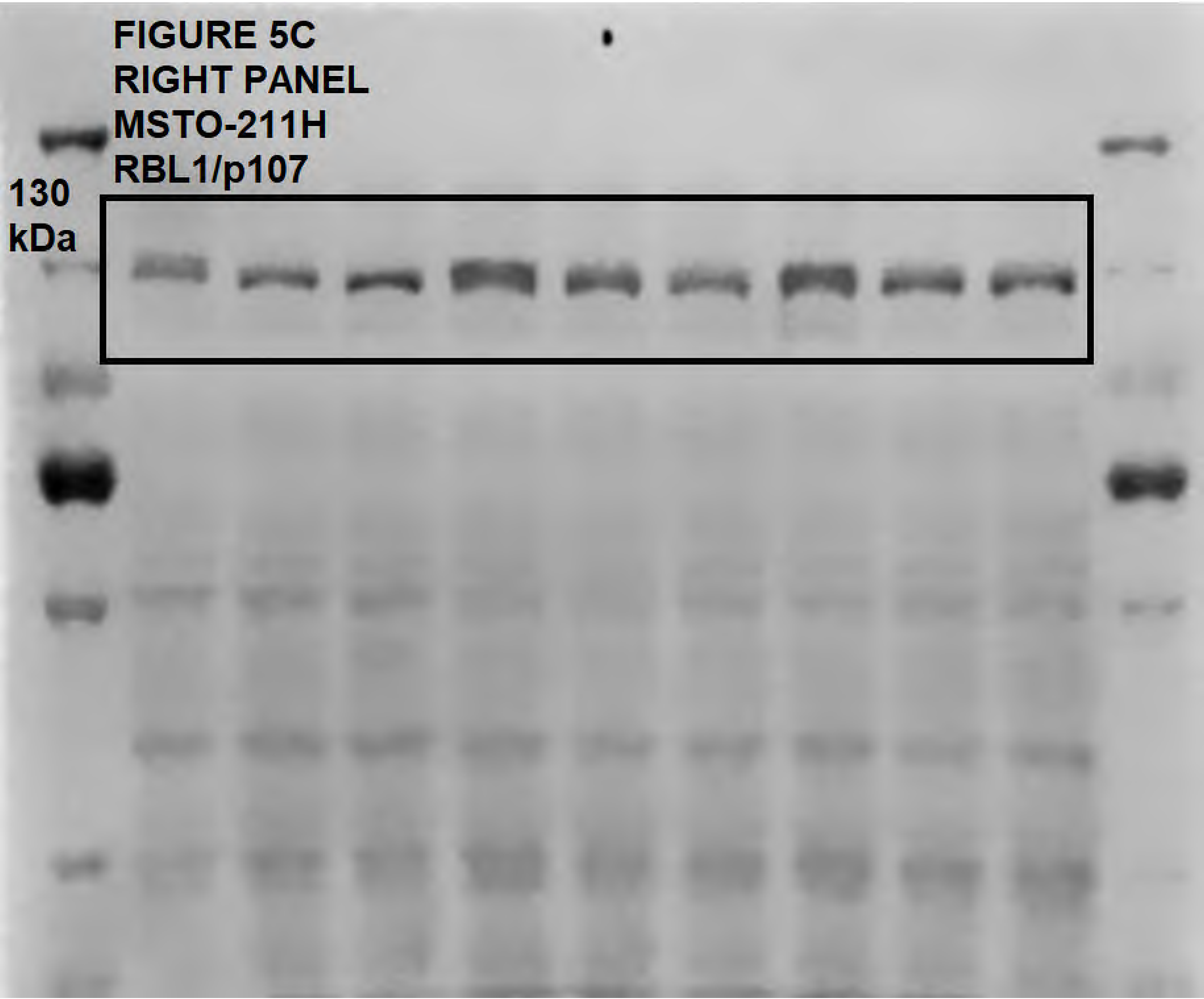
FIGURE 5C
RIGHT PANEL
MSTO-211H
RBL1/p107
130
kDa

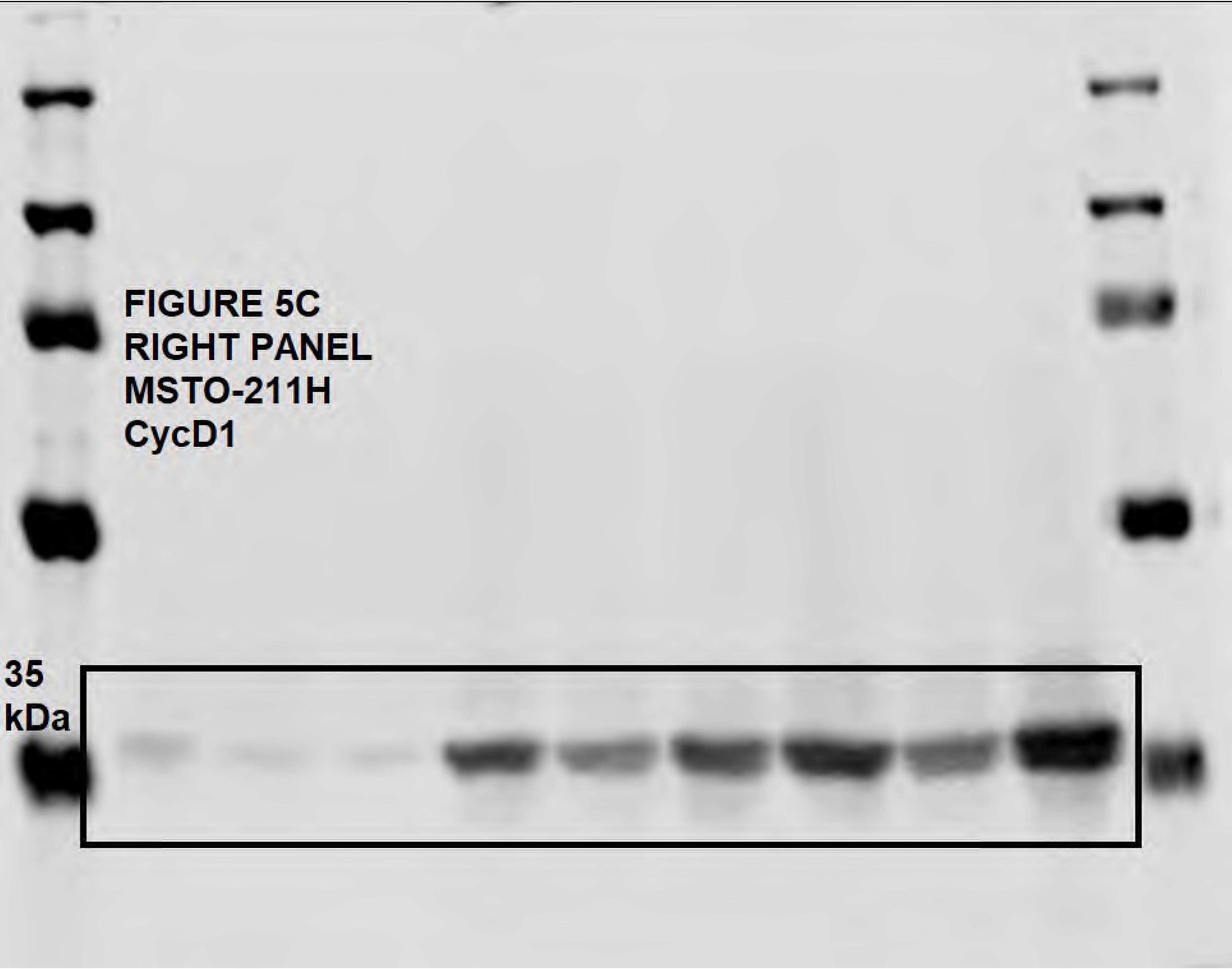
FIGURE 5C
RIGHT PANEL
MSTO-211H
CycD1
35
kDa

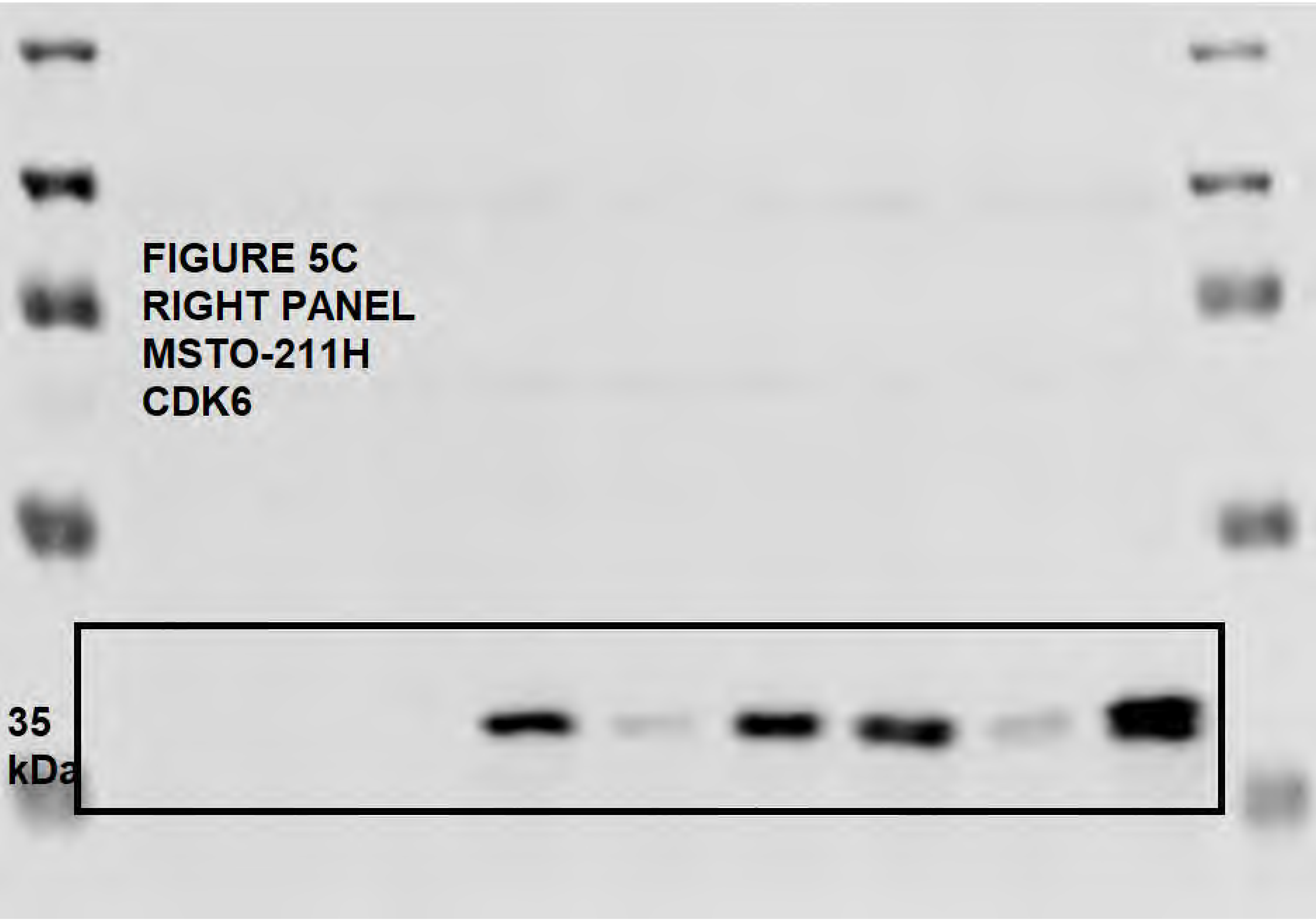
FIGURE 5C
RIGHT PANEL
MSTO-211H
CDK6
35
kDa

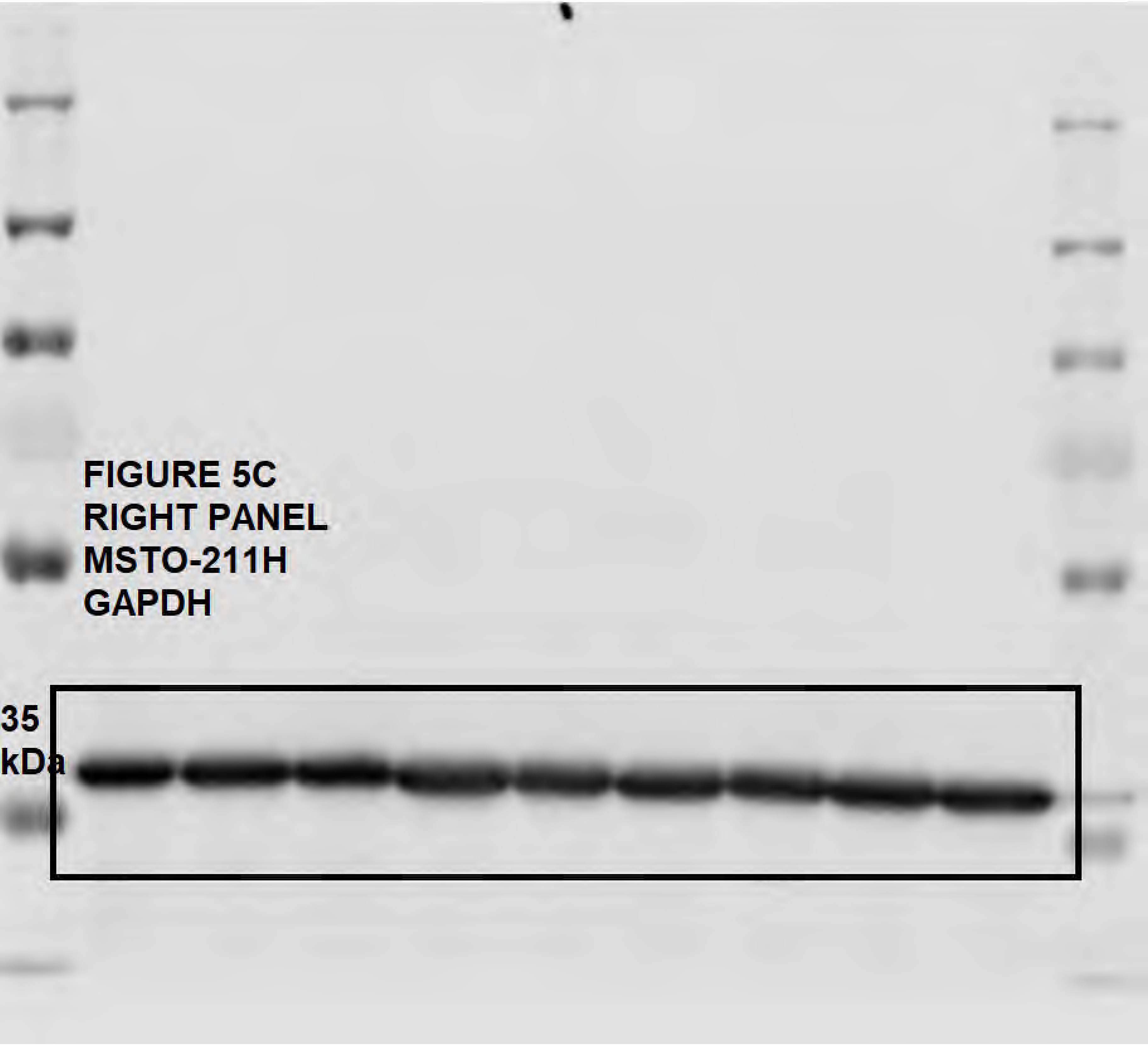
FIGURE 5C
RIGHT PANEL
MSTO-211H
GAPDH
35
kDa

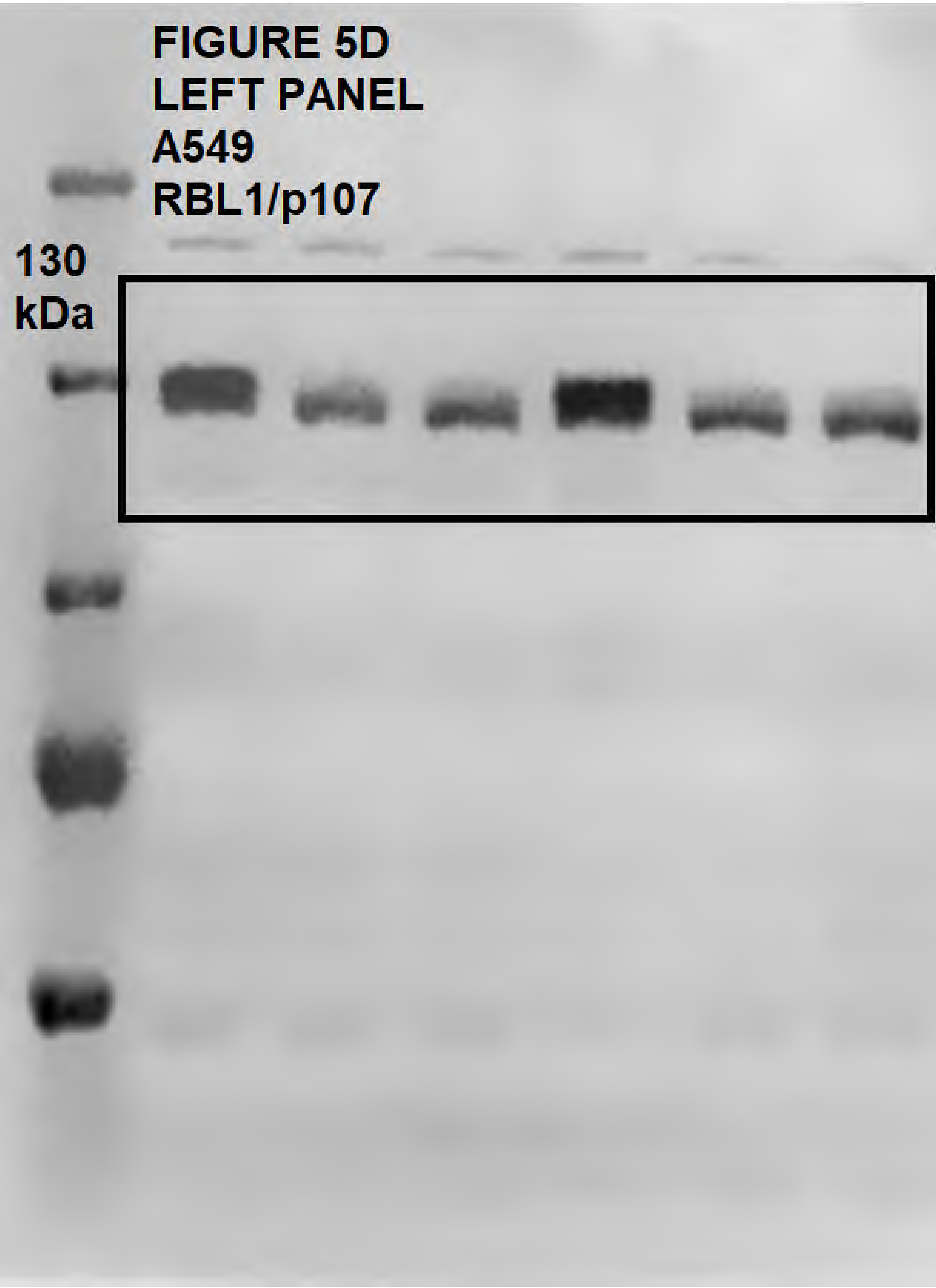
FIGURE 5D
LEFT PANEL
A549
RBL1/p107
130
kDa

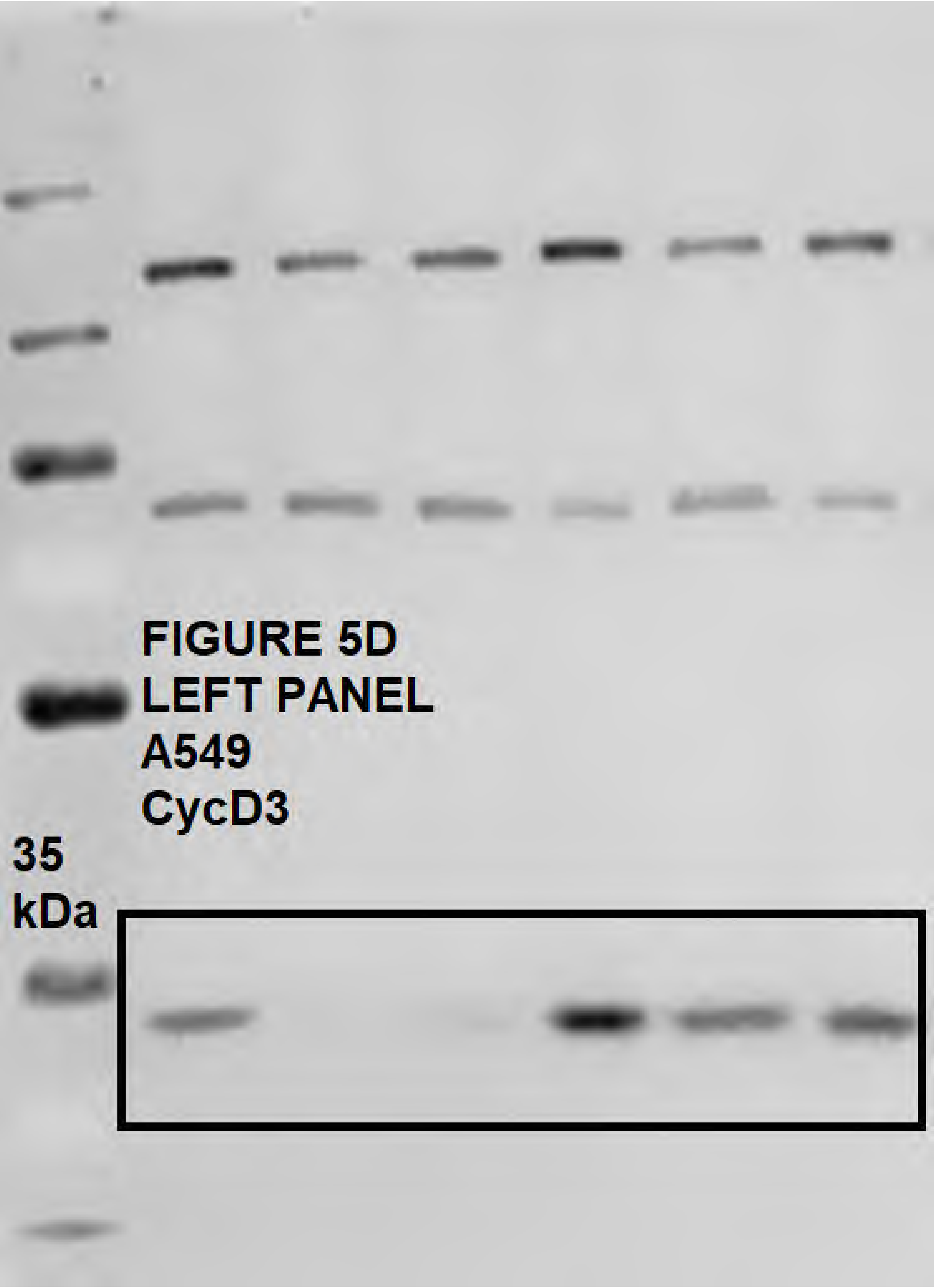
FIGURE 5D
LEFT PANEL
A549
CycD3
35
kDa

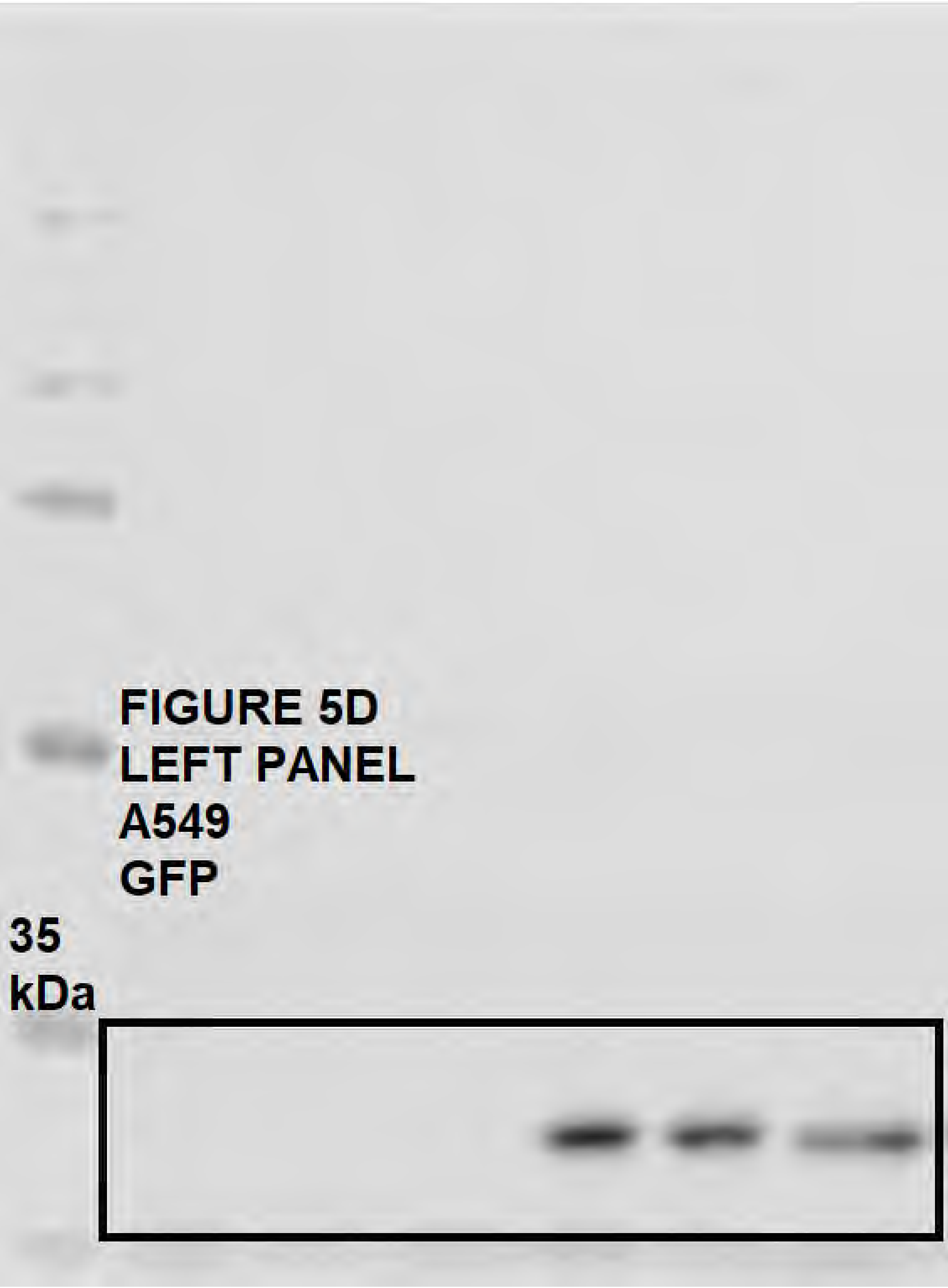
FIGURE 5D
LEFT PANEL
A549
GFP
35
kDa

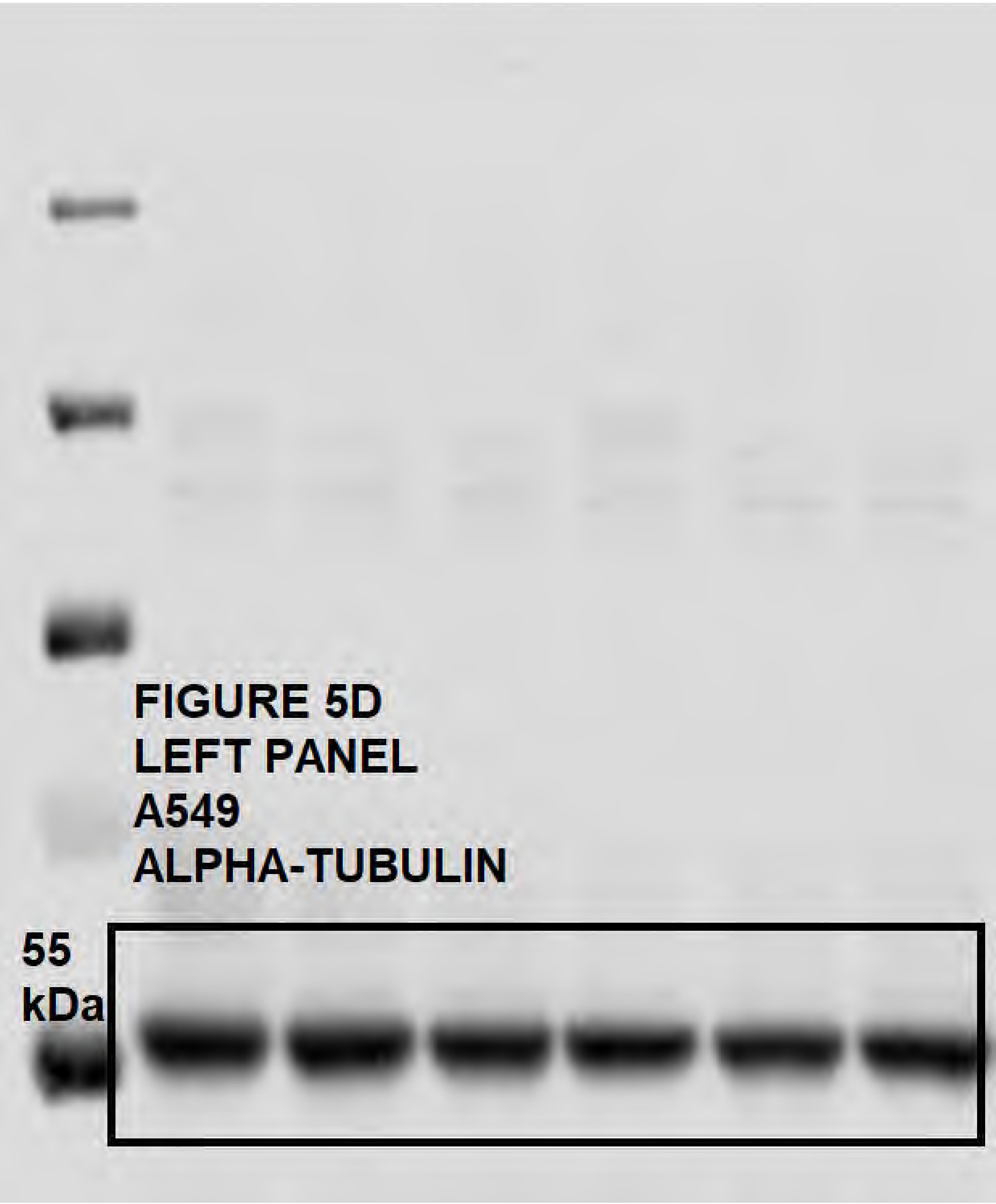
FIGURE 5D
LEFT PANEL
A549
ALPHA-TUBULIN
55
kDa

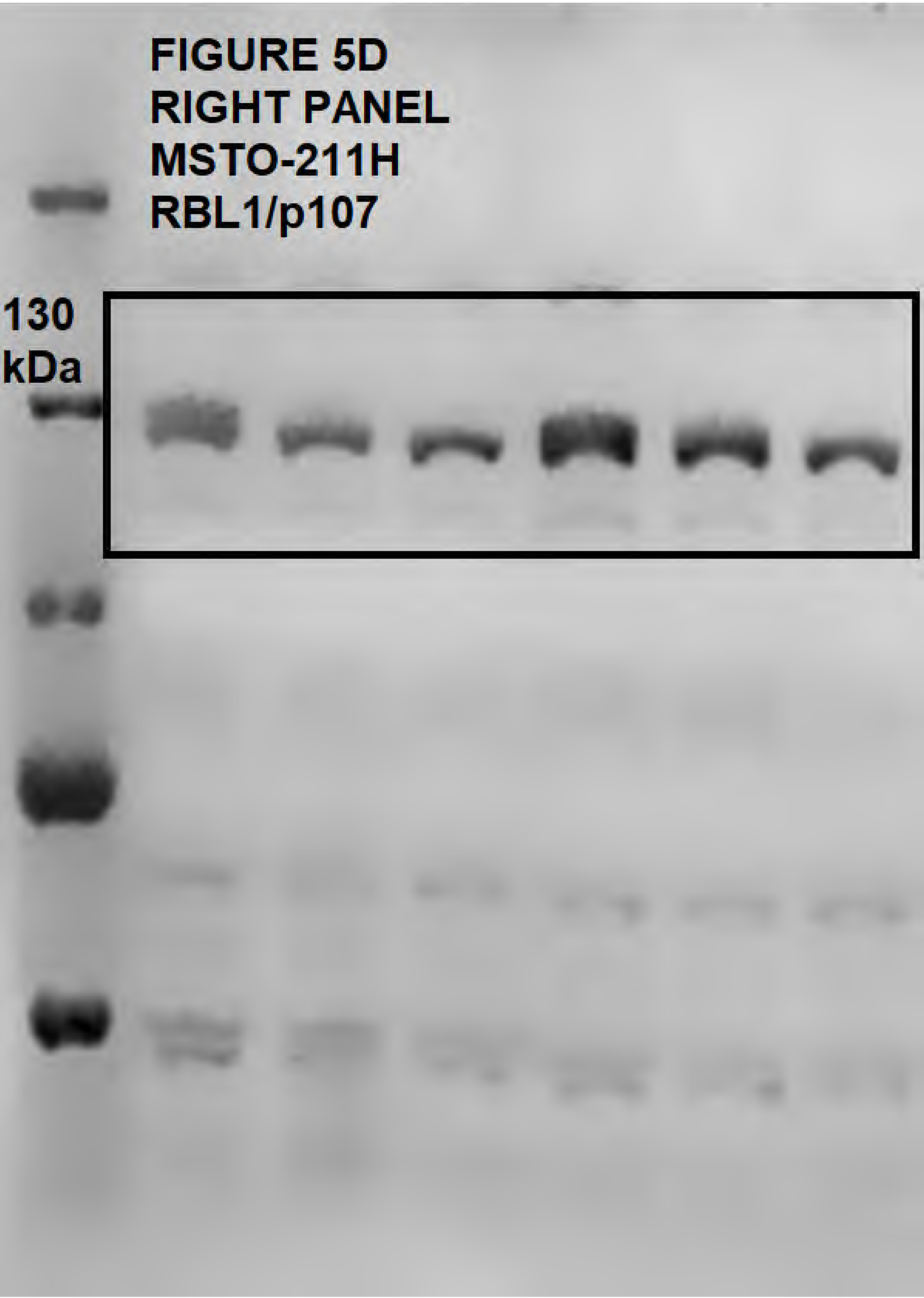
FIGURE 5D
RIGHT PANEL
MSTO-211H
RBL1/p107
130
kDa

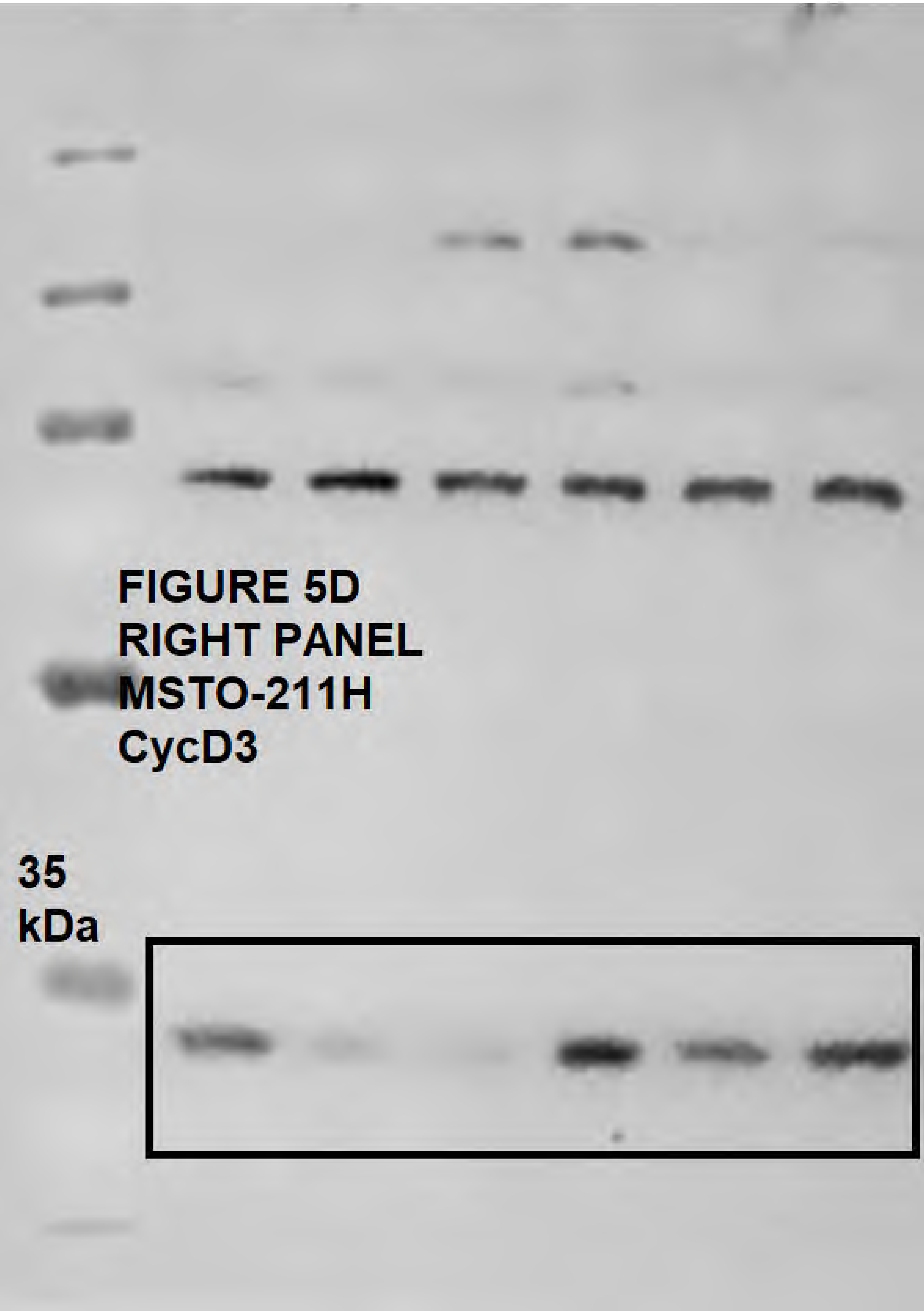
FIGURE 5D
RIGHT PANEL
MSTO-211H
CycD3
35
kDa

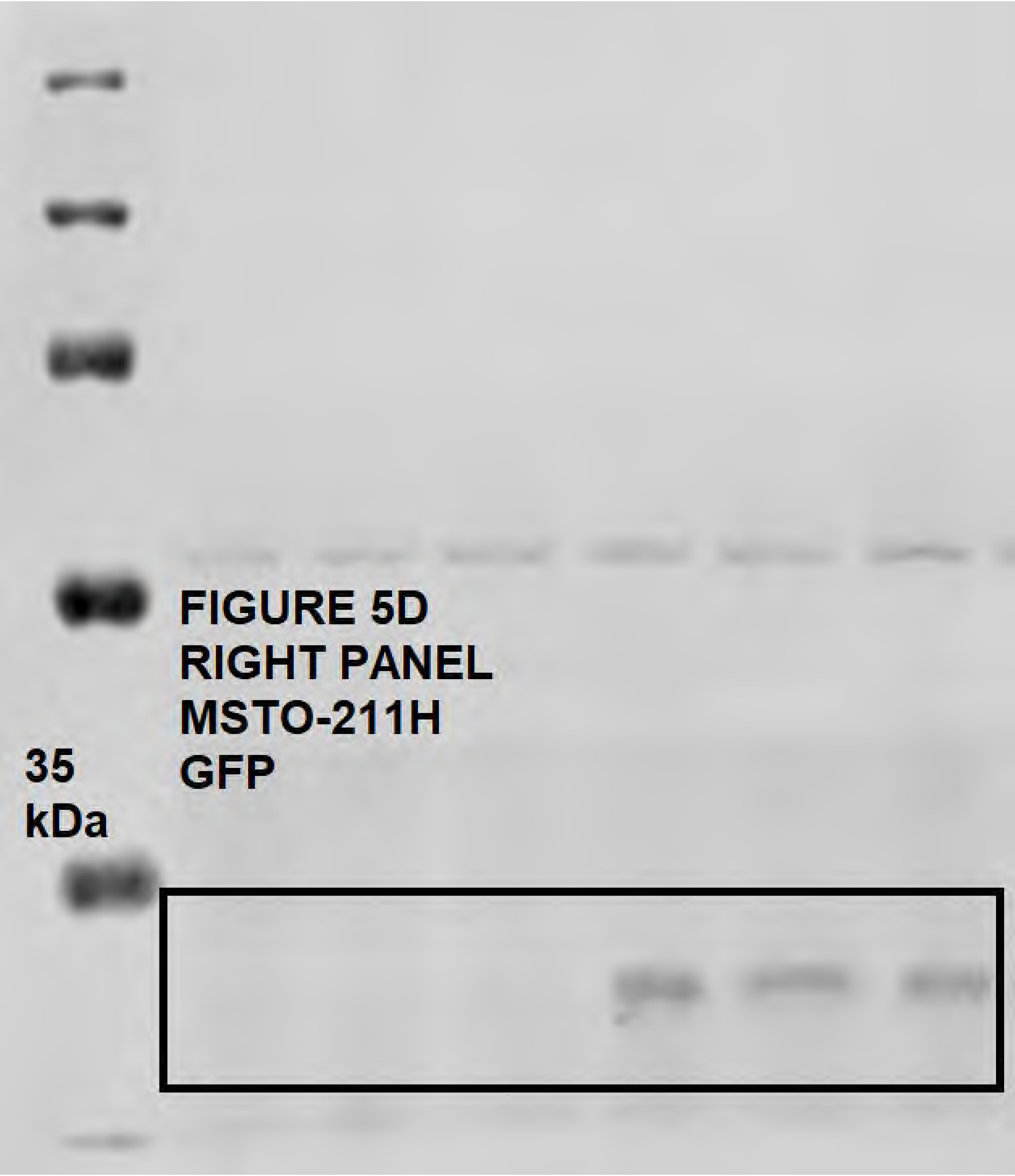
FIGURE 5D
RIGHT PANEL
MSTO-211H
GFP
35
kDa

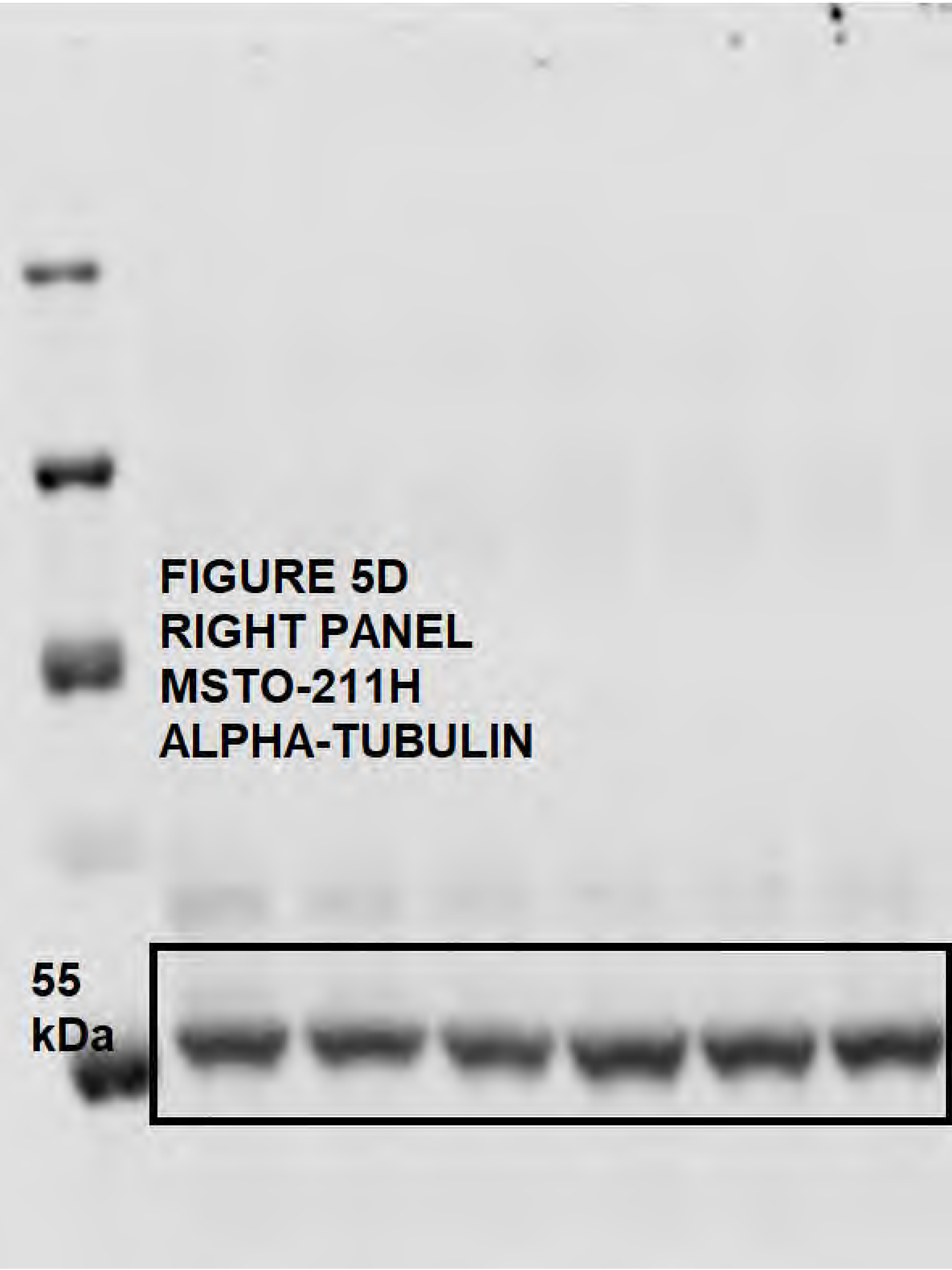
FIGURE 5D
RIGHT PANEL
MSTO-211H
ALPHA-TUBULIN
55
kDa
